# Author Correction: Hidden impacts of ocean warming and acidification on biological responses of marine animals revealed through meta-analysis

**DOI:** 10.1038/s41467-024-53492-y

**Published:** 2024-10-23

**Authors:** Katharina Alter, Juliette Jacquemont, Joachim Claudet, María E. Lattuca, María E. Barrantes, Stefano Marras, Patricio H. Manríquez, Claudio P. González, Daniel A. Fernández, Myron A. Peck, Carlo Cattano, Marco Milazzo, Felix C. Mark, Paolo Domenici

**Affiliations:** 1 Royal Netherlands Institute for Sea Research, Department of Coastal Systems, P.O. Box 59, 1790 AB Den Burg, The Netherlands; 2 School of Aquatic and Fishery Sciences, University of Washington, 1122 NE Boat St, 98195 Seattle, WA USA; 3 National Center for Scientific Research, PSL Université Paris, CRIOBE, CNRS-EPHE-UPVD, Maison de l’Océan, 195 rue Saint-Jacques, 75005 Paris, France; 4 Centro Austral de Investigaciones Científicas (CADIC-CONICET), Bernardo Houssay 200, V9410CAB Ushuaia, Argentina; 5 Universidad Nacional de Tierra del Fuego, Antártida e Islas del Atlántico Sur; Instituto de Ciencias Polares, Ambiente y Recursos Naturales (UNTDF - ICPA), Fuegia Basket 251, V9410BXE Ushuaia, Argentina; 6 CNR-IAS, Consiglio Nazionale delle Ricerche, Instituto per lo studio degli Impatti Antropici e Sostenibilità in ambiente marino. Località Sa Mardini, 09170 Torregrande, Oristano Italy; 7 Centro de Estudios Avanzados en Zonas Áridas (CEAZA), Coquimbo, Chile; 8 Laboratorio de Ecología y Conducta de la Ontogenia Temprana (LECOT), Coquimbo, Chile; 9 Wageningen University, Department of Animal Sciences, Marine Animal Ecology Group, De Elst 1, 6708 WD Wageningen, The Netherlands; 10 NBFC, National Biodiversity Future Center, Palermo, Italy; 11 Department of Integrative Marine Ecology, Stazione Zoologica Anton Dohrn (SZN), Lungomare Cristoforo Colombo, I-90149 Palermo, Italy; 12 Dipartimento di Scienze della Terra e del Mare (DiSTeM), Università di Palermo, Via Archirafi 20, I-90123 Palermo, Italy; 13 Section of Integrative Ecophysiology, Alfred Wegener Institute Helmholtz Centre for Polar and Marine Research, Am Handelshafen 12, Bremerhaven, 27570 Germany; 14 CNR-IBF, Area di Ricerca San Cataldo, Via G. Moruzzi N°1, 56124 Pisa, Italy

**Keywords:** Climate-change ecology, Ecophysiology, Behavioural ecology, Biodiversity

Correction to: *Nature Communications* 10.1038/s41467-024-47064-3, published online 3 April 2024

The original version of this Article contained an error in Fig. 5a, in which the tiles for the fish were incorrectly ordered. The correct version of Fig. 5 is:
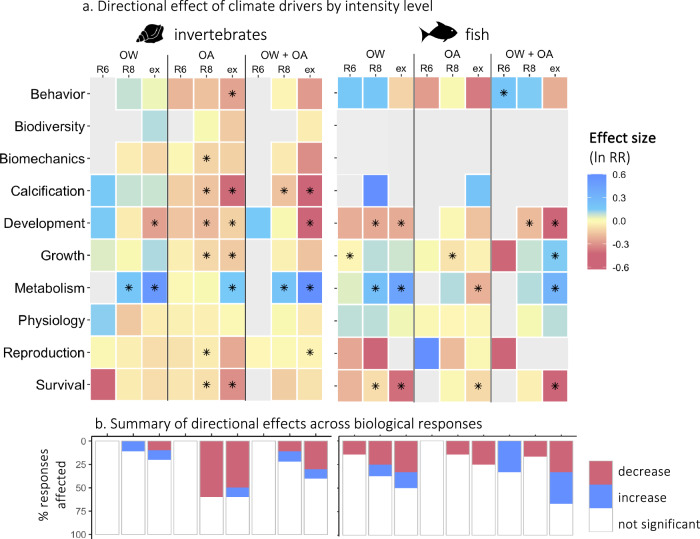


which replaces the previous incorrect version:
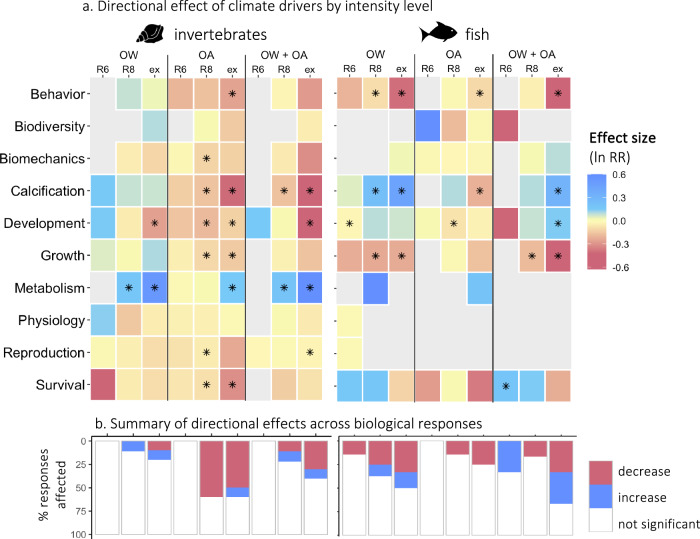


This has been corrected in both the PDF and HTML versions of the Article.

