# Supplementary material for: Hidden impacts of ocean warming and acidification on biological responses of marine animals revealed through meta-analysis
Source: Nat Commun. 2024 Apr 3;15:2885. doi: 10.1038/s41467-024-47064-3 (PMC10991405; doi:10.1038/s41467-024-47064-3)
Supplement: Supplementary file 1 — Supplementary Information [file 41467_2024_47064_MOESM1_ESM.pdf]

Supplementary material

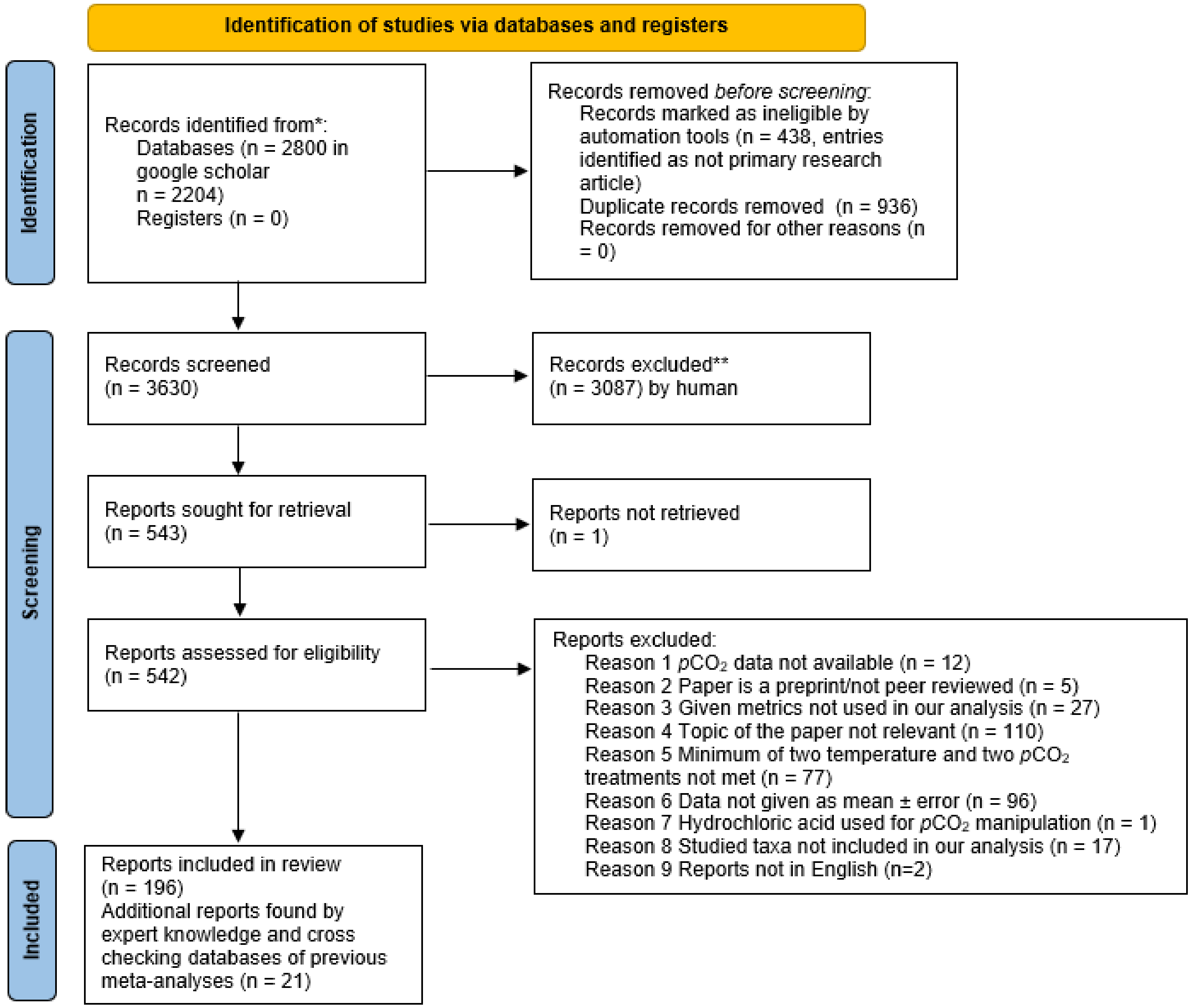

Supplementary Fig. 1: PRISMA flow diagram of the systematic review process of this study. This figure was based on the template provided in Page et al.[59].

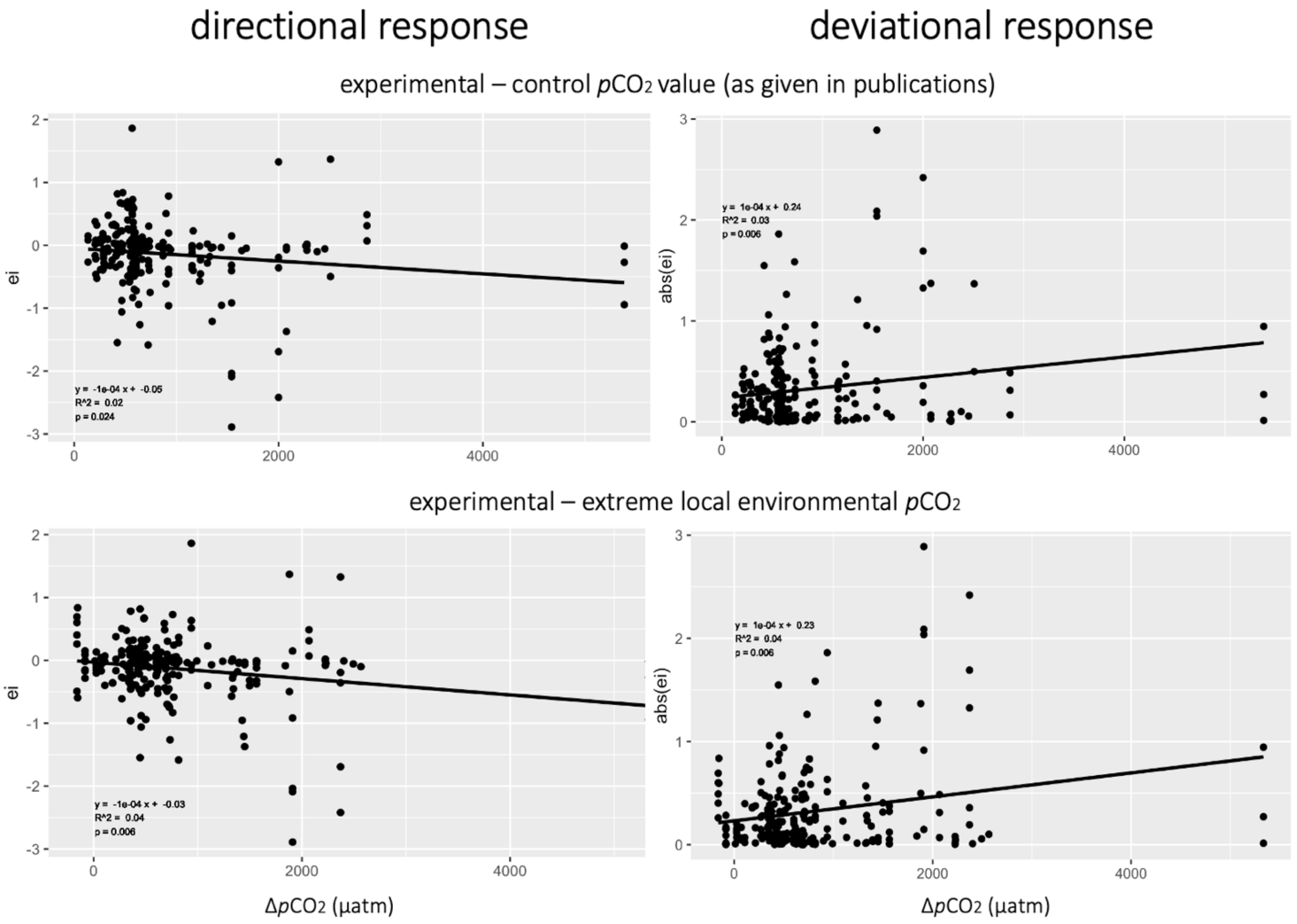

Supplementary Fig. 2: Correlation of biological responses of invertebrates to the intensity of experimental ocean acidification treatments. Directional (lnRR; left panels) and deviational ((abs(lnRR); right panels) responses of invertebrates to the intensity of experimental ocean acidification treatments (ΔpCO_2, µatm) were either calculated by using treatment and control pCO_2 values as given by authors in publications (top panels) or by using extreme local environmental pCO_2 as the control value (bottom panels), following Vargas et al.[46]. The intercept and the coefficient of the linear regression is indicated on plots, along with the R-squared and the p-value associated with the fit of the linear regression to the data set.

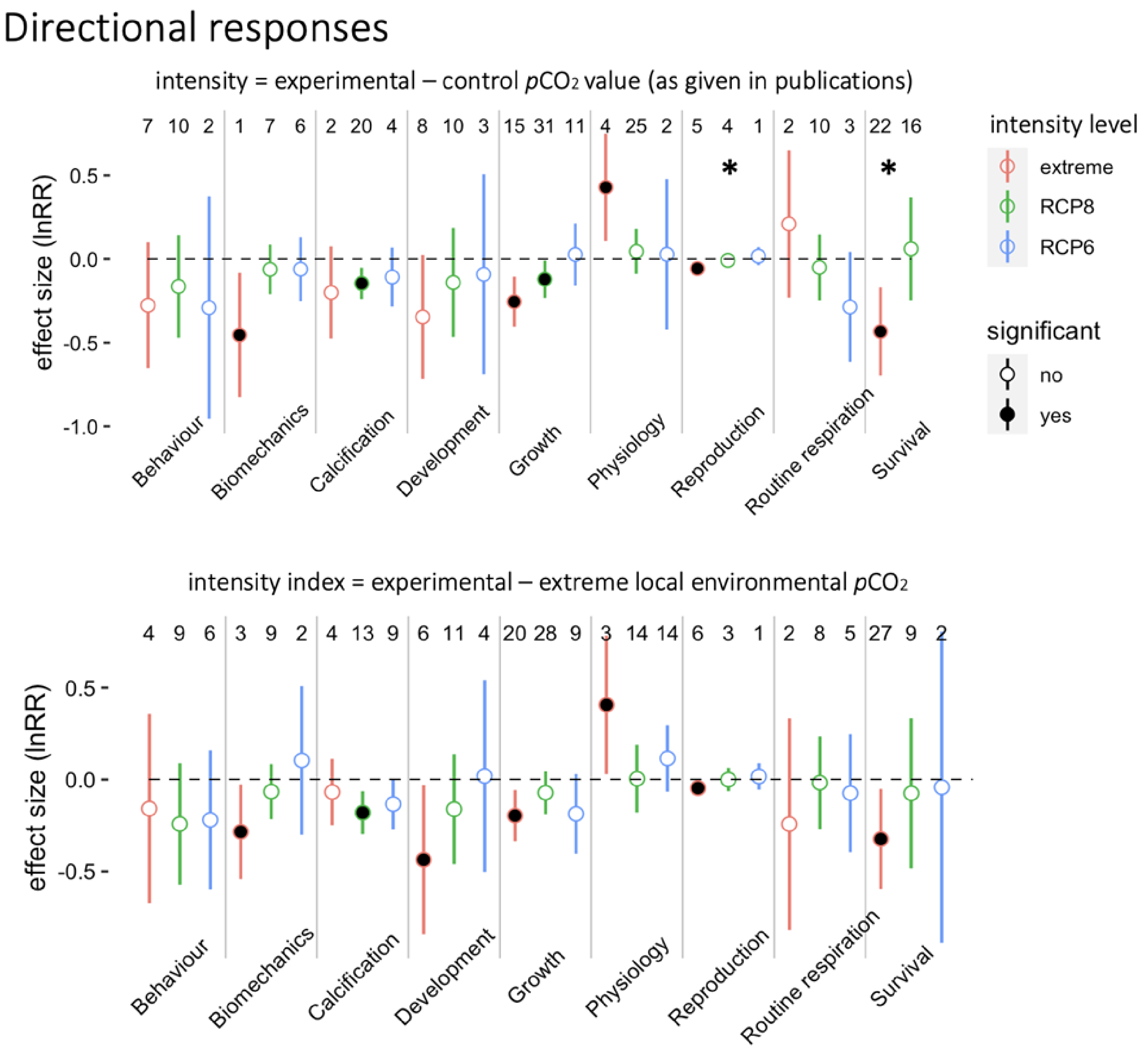

Supplementary Fig. 3: Directional responses (lnRR) of invertebrates to ocean acidification across biological responses at different intensity levels. In the top panel, intensity levels were determined using treatment and control pCO_2 values as given by authors in publications. In the bottom panel, intensity levels were determined using extreme local environmental pCO_2 as control values, following Vargas et al.[46]. Effect sizes are considered significant when 95% confidence intervals do not overlap zero. Significant effect sizes are displayed with black fills, while non-significant effect sizes have white fills. Significant differences between effect sizes for different intensity levels within a biological response is denoted by an *asterisk*. Sample sizes are indicated above effect sizes.

Deviational responses

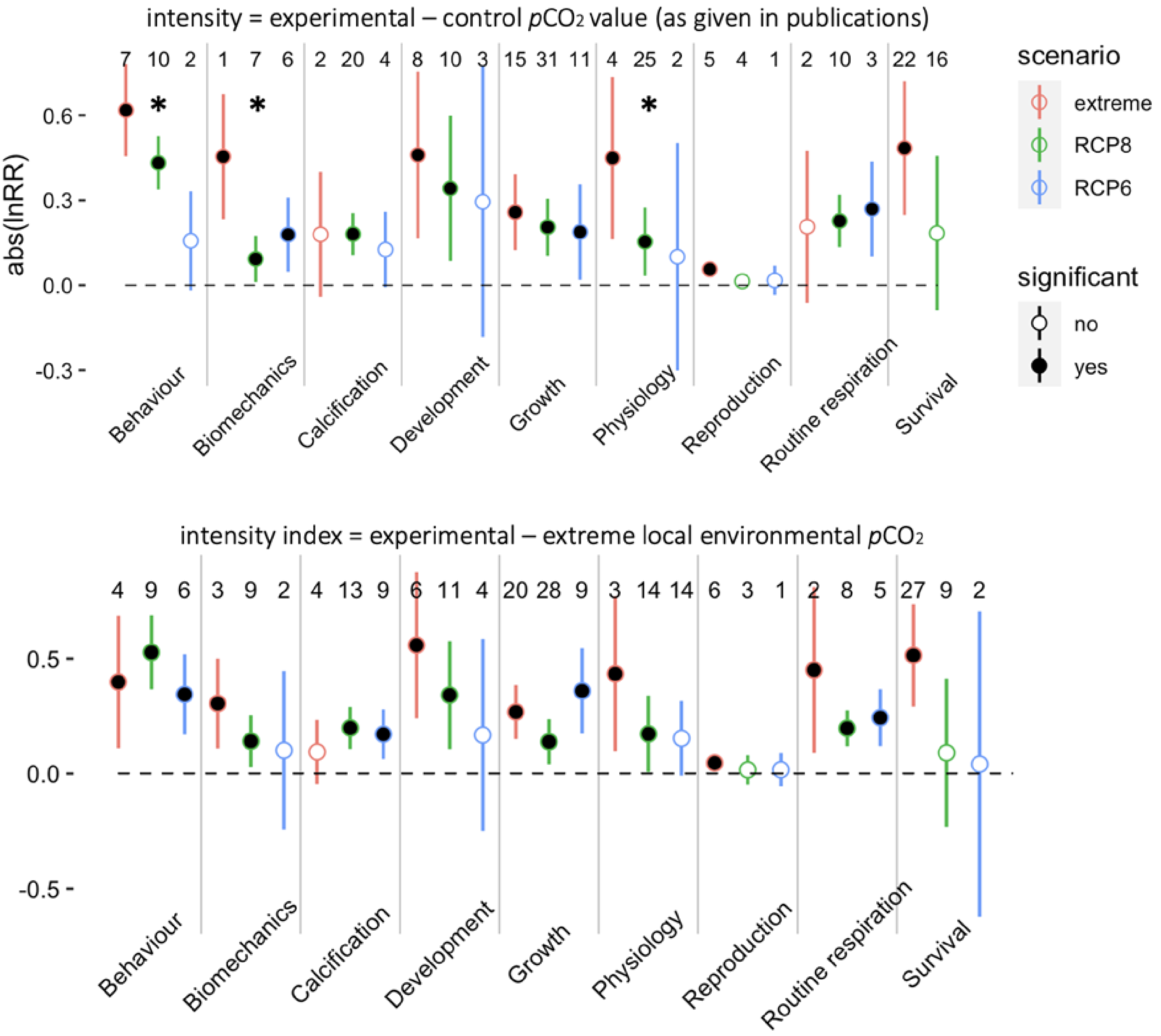

Supplementary Fig. 4: Deviational responses (abs(lnRR)) of invertebrates to ocean acidification across biological responses at different intensity levels. In the top panel, intensity levels were determined using treatment and control pCO_2 values as given by authors in publications. In the bottom panel, intensity levels were determined using extreme local environmental pCO_2 as control values, following Vargas et al.[46]. Effect sizes are considered significant when 95% confidence intervals do not overlap zero. Significant effect sizes are displayed with black fills, while non-significant effect sizes have white fills. Significant differences between effect sizes for different intensity levels within a biological response is denoted by an *asterisk*. Sample sizes are indicated above effect sizes.

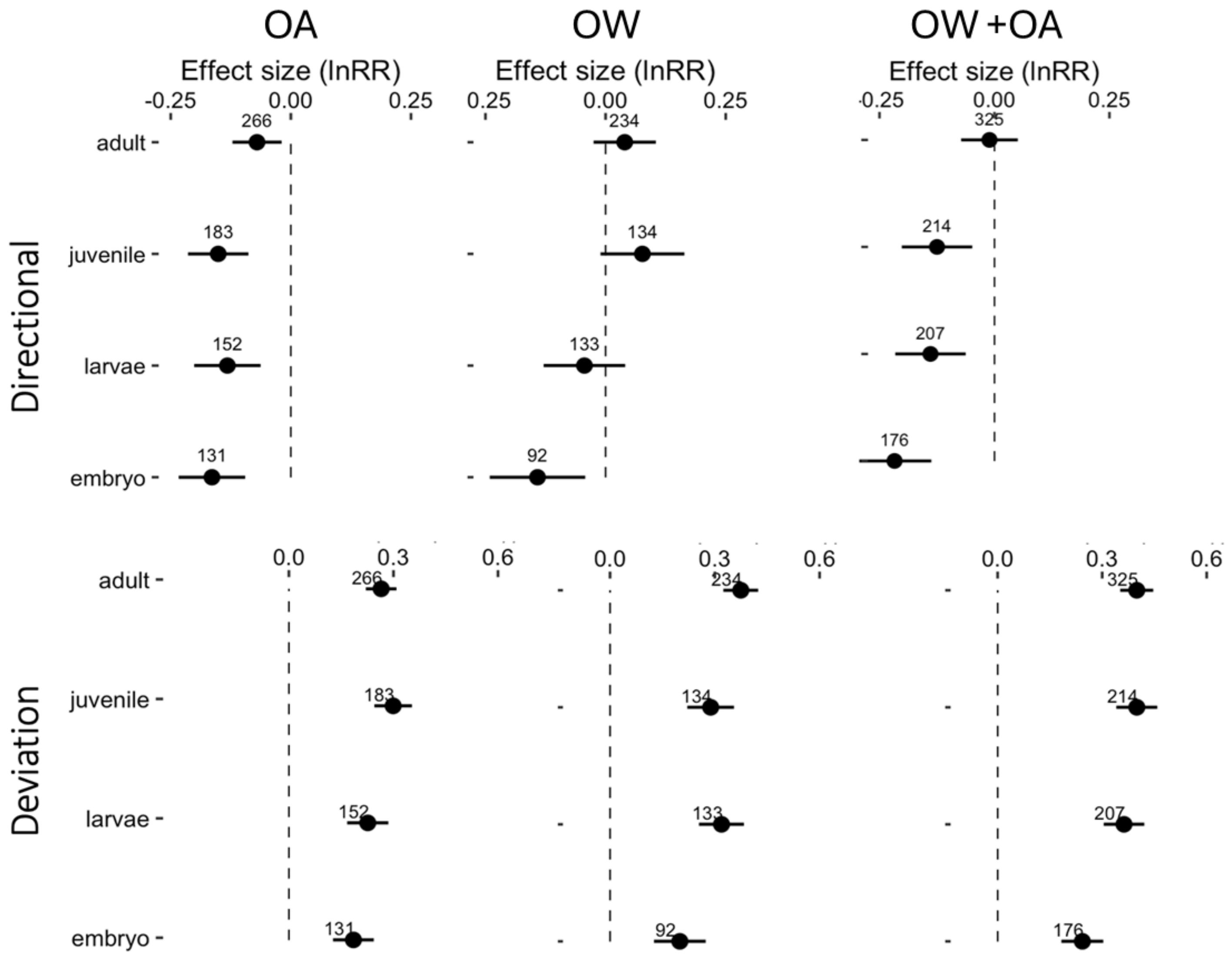

Supplementary Fig. 5: Biological response of invertebrates across life stages to climate drivers. Top panels display directional responses (lnRR) and bottom panels display deviational responses (abs(lnRR)) to ocean acidification (OA), ocean warming (OW) and their combination (OW + OA). Dots and bars represent mean effect size and their associated 95% confidence interval (CI). Numbers indicate the sample size (number of experiments) associated with each effect size. Effect sizes are considered significant when their 95% CI does not overlap zero (hashed lines).

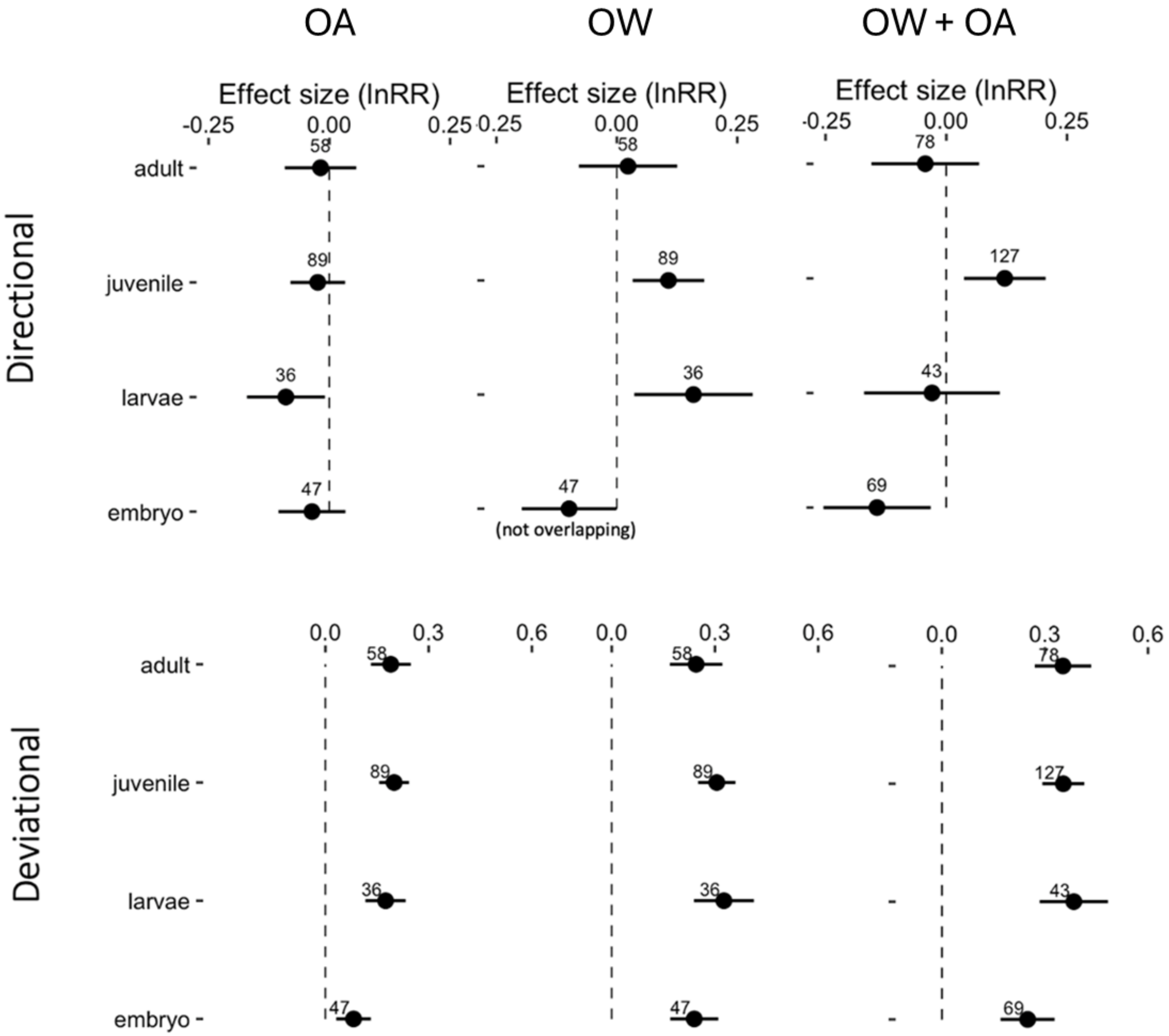

Supplementary Fig. 6: Biological responses of fish across life stages to climate drivers. Top panels display directional responses (lnRR) and bottom panels display deviational responses (abs(lnRR)) to ocean acidification (OA), ocean warming (OW) and their combination (OW + OA). Dots and bars represent mean effect size and their associated 95% confidence interval (CI). Numbers indicate the sample size (number of experiments) associated with each effect size. Effect size are considered significant when their 95% CI does not overlap zero (hashed lines).

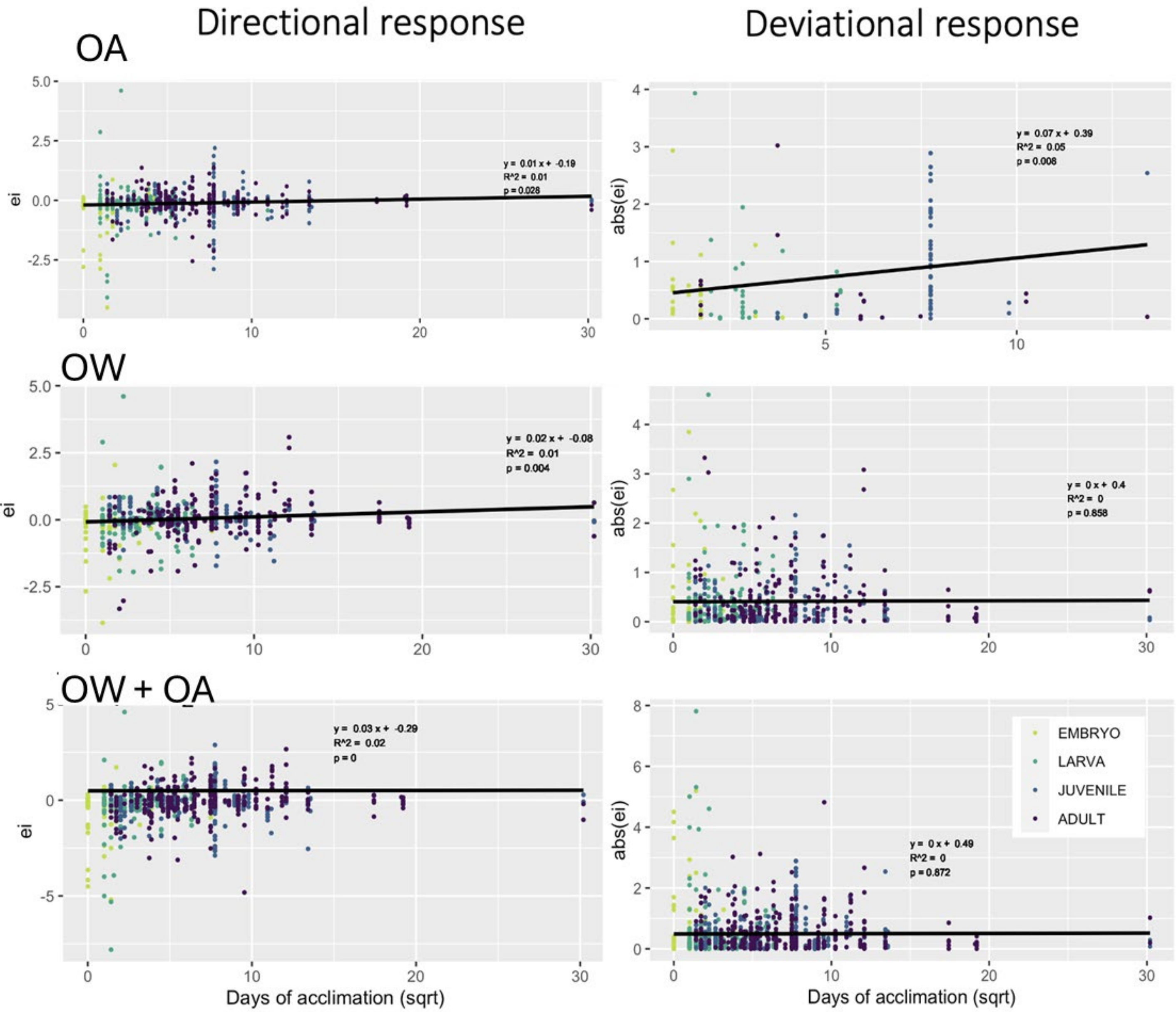

Supplementary Fig. 7: Effect of the number of acclimation days in experiments on the biological responses of invertebrates to climate drivers. Left plots display directional responses (lnRR) and right plots deviational responses (abs(lnRR)) to ocean acidification (OA; top panel), ocean warming (OW; middle panel) and their combination (OW + OA; bottom panel). The color of dots, which each represent the effect size associated with individual experiments, indicates the life stage of the organism tested (embryo, larva, juvenile, and adult). The intercept and the coefficient of the linear regression is indicated on plots, along with the R-squared and the p-value associated with the fit of the linear regression to the data set.

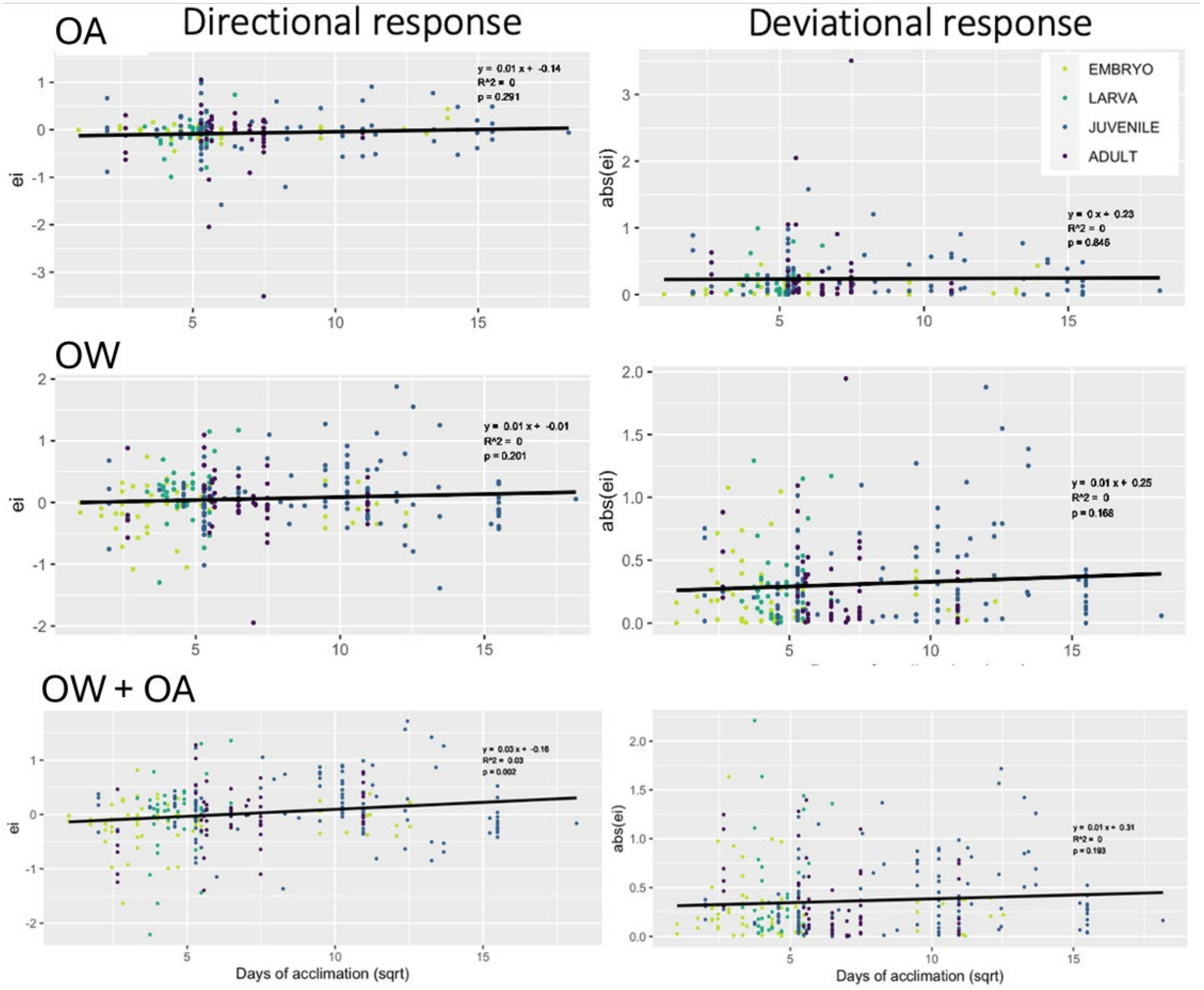

Supplementary Fig. 8: Effect of the number of acclimation days in experiments on the biological response of fish to climate drivers. Left plots display directional responses (lnRR) and right plots deviational responses (abs(lnRR)) to ocean acidification (OA; top panel), ocean warming (OW; middle panel) and their combination (OW + OA; bottom panel). The color of dots, which each represent the effect size associated with individual experiments, indicates the life stage of the organism tested (embryo, larva, juvenile, and adult). The intercept and the coefficient of the linear regression is indicated on plots, along with the R-squared and t p-value associated with the fit of the linear regression to the data set.

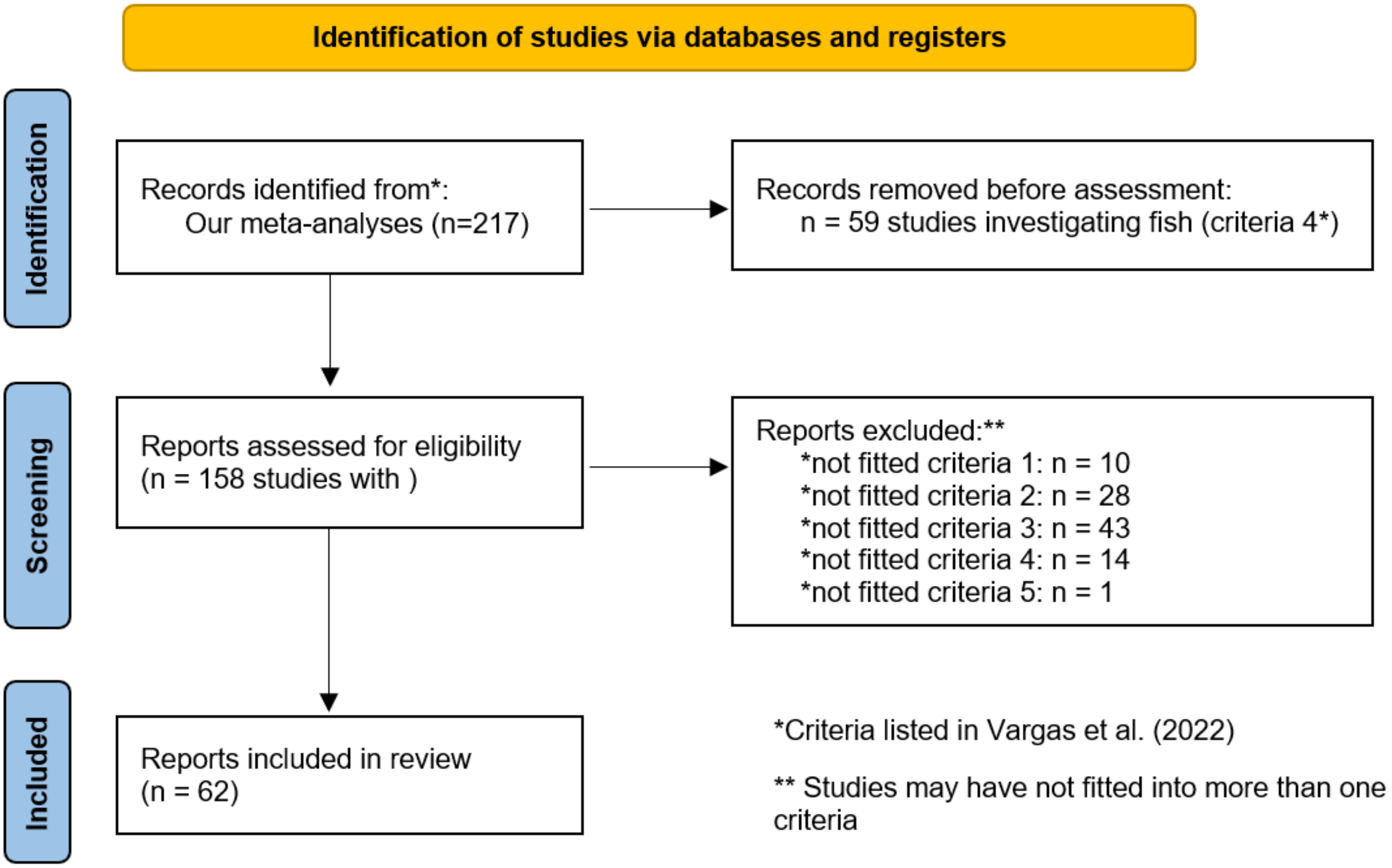

Supplementary Fig. 9: PRISMA flow diagram of the systematic review of the studies used in our meta-analyses against the selection criteria used in Vargas et al.[46]. This figure was based on the template provided in Page et al.[59].

Supplementary Table 1: Model heterogeneity and residual heterogeneity associated with the effect of climate driver intensity level on the directional change (lnRR) of biological responses of fish and invertebrates (one-way ANOVA) to ocean warming (OW), ocean acidification (OA), and their combination (OW+OA).

				**Model heterogeneity**			**Residual heterogeneity**		
**taxa**	**biological response**	**driver**	**n**	**Qm**	**df**	**p-value**	**Qe**	**df**	**p-value**
fish	Behavior	OW	40	4.55	2	0.1	314	37	1.18E-45
		OA	35	2.72	2	0.26	265	32	2.05E-38
		OW+OA	45	3.84	2	0.15	319	31	1.63E-49
	Development	OW	19	0.01	2	1	1916	16	0.00E+00
		OA	15	1.39	2	0.5	1641	12	0.00E+00
		OW+OA	21	5.15	1	0.02	174	6	3.69E-34
	Growth	OW	64	0.53	2	0.77	3026	61	0.00E+00
		OW+OA	74	4.56	2	0.1	2786	44	0.00E+00
		OA	50	0.82	2	0.66	1083	47	5.19E-196
	Metabolism	OW	47	4.32	2	0.12	718	44	9.88E-123
		OA	34	8.84	1	0	331	31	2.13E-51
		OW+OA	50	1.44	1	0.23	443	24	4.06E-78
	Physiology	OW	72	0.63	2	0.73	952	69	2.35E-155
		OW+OA	78	0.29	2	0.87	619	44	1.73E-102
		OA	62	0.34	2	0.84	515	59	5.05E-74
	Reproduction	OW	7	0.68	1	0.41	48	4	4.45E-09
		OA	6	0.89	2	0.64	13	3	3.99E-03
		OW+OA	10	NA	NA	NA	NA	NA	NA
	Survival	OW	31	2.34	2	0.31	588	28	4.31E-106
		OA	25	1.63	2	0.44	897	22	1.66E-175
		OW+OA	35	5.15	1	0.02	2677	19	0.00E+00
invertebrate	Behavior	OA	59	0.59	1	0.44	639	22	2.87E-120
		OW	46	0.39	2	0.82	363	43	2.62E-52
		OW+OA	48	6.07	2	0.05	1001	45	1.76E-180
	Biomechanics	OA	29	1.49	2	0.47	99	26	1.65E-10
		OW	23	1.87	2	0.39	200	20	1.09E-31
		OW+OA	33	1.99	1	0.16	123	15	1.61E-18
	Biodiversity	OA	9	0.65	1	0.42	21	6	3.89E-03
		OW+OA	9	NA	NA	NA	NA	NA	NA
		OW	3	NA	NA	NA	NA	NA	NA
	Calcification	OA	61	2.75	2	0.25	20412	58	0.00E+00
		OW	52	0.13	2	0.94	345	49	3.34E-46
		OW+OA	70	3.11	2	0.21	534	33	1.10E-91
	Development	OA	119	0.25	2	0.88	12967	116	0.00E+00
		OW	84	5.52	2	0.06	6258	81	0.00E+00
		OW+OA	154	7.2	2	0.03	5188	62	0.00E+00
	Growth	OA	147	2.03	2	0.36	2710	144	0.00E+00
		OW	118	1.61	2	0.45	2462	115	0.00E+00
		OW+OA	190	2.11	2	0.35	2707	76	0.00E+00
	Metabolism	OA	54	2.45	2	0.29	194	51	1.31E-18
		OW	53	7.15	1	0.01	514	50	8.47E-78
		OW+OA	67	5.93	1	0.01	708	39	5.05E-123
	Physiology	OA	92	0.11	2	0.95	1941	89	0.00E+00
		OW	85	1.55	2	0.46	2300	82	0.00E+00
		OW+OA	104	0.35	1	0.55	825	48	1.42E-141
	Reproduction	OA	62	0.68	2	0.71	505	59	4.55E-72
		OW	59	0.21	2	0.9	659	56	9.07E-104
		OW+OA	111	1.33	2	0.51	102	37	6.21E-08
	Survival	OW+OA	125	0.47	2	0.79	343	42	7.74E-49
		OA	115	7.21	2	0.03	6936	112	0.00E+00
		OW	68	2.5	2	0.29	755	65	4.02E-118

Supplementary Table 2: Model heterogeneity and residual heterogeneity. Model heterogeneity and residual heterogeneity associated with the effect of life stage as a moderator of the biological responses of fish and invertebrates (one-way ANOVA) to ocean warming (OW), ocean acidification (OA) and their combination (OW+OA). p-values associated with significant results (QMp < 0.05) are indicated in bold.

analysis	taxa	driver	QM	QMdf	QMp	QE	QEdf	QEp
Directional response (lnRR)	fish	OA	2.1003983	3	0.55183219	11396.9693	227	0
		OW	14.3860911	3	**0.00242406**	29946.3318	281	0
		OW+OW	15.0562299	3	**0.00176922**	53172.7851	314	0
	invertebrate	OA	6.45616205	3	0.09140749	75672.452	731	0
		OW	13.2487218	3	**0.00412847**	20497.7892	590	0
		OW+OW	17.5273001	3	**0.00055047**	33796.1618	919	0
Deviation response (abs(lnRR))	fish	OA	13.7703962	3	**0.00323494**	8135.65184	227	0
		OW	3.95232246	3	0.26665868	17546.04	281	0
		OW+OW	5.99009039	3	0.11209335	33809.4588	314	0
	invertebrate	OA	9.02478119	3	**0.02896322**	68522.7863	731	0
		OW	15.3351718	3	**0.00155151**	16336.2081	590	0
		OW+OW	18.8614483	3	**0.00029204**	27462.4165	919	0

Supplementary Table 3: Results of the directional meta-analyses (lnRR) performed for each combination of taxa, driver, and biological response. Lower and upper confidence interval (CI) are the lower and upper boundaries of the 95% CI associated with the effect size ei. n is the number of values from which effect size was calculated. Qt is the between-study heterogeneity, df is the degree of freedom, and pQt is the p-value associated with this heterogeneity. OW = ocean warming. OA = ocean acidification.

taxa	biological response	driver	n	p-value	Lower CI	Upper CI	ei	Qt	df	pQt
Fish	Behavior	OW	40	0.239	-0.05	0.202	0.076	353.046	39	0
		OA	35	0.392	-0.231	0.091	-0.07	264.816	34	0
		OW + OA	45	0.561	-0.085	0.156	0.036	384.317	44	0
	Calcification	OW	4	0.196	-0.186	0.904	0.359	4107.23	3	0
		OA	3	0.079	-0.042	0.754	0.356	236.216	2	0
		OW + OA	4	0.066	-0.037	1.179	0.571	5576.731	3	0
	Development	OW	19	0.001	-0.382	-0.091	-0.236	2632.517	18	0
		OA	15	0.294	-0.205	0.062	-0.071	4690.182	14	0
		OW + OA	21	0	-0.506	-0.147	-0.327	12571.384	20	0
	Growth	OW	64	0.068	-0.005	0.149	0.072	3256.741	63	0
		OA	50	0.015	-0.092	-0.01	-0.051	1125.523	49	0
		OW + OA	74	0.281	-0.033	0.113	0.04	3449.385	73	0
	Metabolism	OW	47	0	0.21	0.45	0.33	1088.218	46	0
		OA	34	0.863	-0.125	0.105	-0.01	606.558	33	0
		OW + OA	50	0	0.138	0.446	0.292	983.115	49	0
	Physiology	OW	72	0.204	-0.025	0.119	0.047	991.018	71	0
		OA	62	0.311	-0.073	0.023	-0.025	530.094	61	0
		OW + OA	78	0.609	-0.064	0.109	0.022	964.585	77	0
	Reproduction	OW	7	0.015	-0.711	-0.076	-0.394	68.539	6	0
		OA	6	0.8	-0.25	0.324	0.037	29.865	5	0
		OW + OA	10	0.051	-0.479	0.001	-0.239	66.186	9	0
	Survival	OW	31	0.009	-0.435	-0.062	-0.248	1325.228	30	0
		OA	25	0.063	-0.154	0.004	-0.075	902.649	24	0
		OW + OA	35	0	-0.614	-0.188	-0.401	3325.817	34	0
Invertebrate	Behavior	OW	48	0.638	-0.117	0.19	0.037	1040.336	47	0
		OA	46	0.007	-0.376	-0.059	-0.218	385.259	45	0
		OW + OA	59	0.621	-0.097	0.163	0.033	1338.354	58	0
	Biomechanics	OW	3	0.425	-0.145	0.344	0.1	6.605	2	0.037
		OA	9	0.387	-0.24	0.093	-0.073	21.655	8	0.006
		OW + OA	9	0.267	-0.337	0.093	-0.122	66.78	8	0
	Biodiversity	OW	23	0.214	-0.231	0.052	-0.089	212.164	22	0
		OA	29	0.016	-0.166	-0.017	-0.092	154.099	28	0
		OW + OA	33	0.022	-0.267	-0.021	-0.144	391.237	32	0
	Calcification	OW	52	0.081	-0.01	0.175	0.083	389.023	51	0
		OA	61	0.002	-0.332	-0.075	-0.204	34599.72	60	0
		OW + OA	70	0.02	-0.249	-0.021	-0.135	1317.046	69	0
	Development	OW	84	0.065	-0.256	0.008	-0.124	9353.035	83	0
		OA	119	0.001	-0.258	-0.064	-0.161	13289.426	118	0
		OW + OA	154	0	-0.392	-0.163	-0.278	12865.201	153	0
	Growth	OW	118	0.185	-0.026	0.135	0.055	2507.029	117	0
		OA	147	0	-0.158	-0.067	-0.112	2878.22	146	0
		OW + OA	190	0.001	-0.171	-0.045	-0.108	5880.285	189	0
	Metabolism	OW	53	0	0.226	0.494	0.36	598.835	52	0
		OA	54	0.258	-0.035	0.132	0.048	199.093	53	0
		OW + OA	67	0	0.296	0.572	0.434	1112.483	66	0
	Physiology	OW	85	0.148	-0.203	0.03	-0.086	2371.551	84	0
		OA	92	0.559	-0.116	0.063	-0.027	2016.32	91	0
		OW + OA	104	0.452	-0.145	0.064	-0.04	2937.69	103	0
	Reproduction	OW	59	0.084	-0.148	0.009	-0.069	665.108	58	0
		OA	62	0.01	-0.236	-0.031	-0.134	506.409	61	0
		OW + OA	111	0	-0.226	-0.083	-0.154	1075.464	110	0
	Survival	OW	68	0.046	-0.23	-0.002	-0.116	790.588	67	0
		OA	115	0	-0.247	-0.105	-0.176	7243.232	114	0
		OW + OA	125	0	-0.375	-0.149	-0.262	2605.915	124	0

Supplementary Table 4: Results of the deviation meta-analyses (abs(lnRR)) performed for each combination of taxa, driver, and biological response. Lower and upper confidence interval (CI) are the lower and upper boundaries of the 95% CI associated with the effect size ei. n is the number of values from which effect size was calculated. Qt is the between-study heterogeneity, df is the degree of freedom, and pQt is the p-value associated with this heterogeneity. OW = ocean warming, OA = ocean acidification.

taxa	biological response	driver	n	p-value	lower CI	upper CI	ei	Qt	df	pQt
Fish	Behavior	OW	40	0.000	0.200	0.363	0.281	154.413	39	0.000
		OA	35	0.000	0.194	0.427	0.310	172.685	34	0.000
		OW + OA	45	0.000	0.217	0.359	0.288	142.483	44	0.000
	Calcification	OW	4	0.185	-0.175	0.905	0.365	4058.259	3	0.000
		OA	3	0.079	-0.042	0.754	0.356	236.216	2	0.000
		OW + OA	4	0.066	-0.037	1.179	0.571	5576.731	3	0.000
	Development	OW	19	0.000	0.131	0.399	0.265	2514.872	18	0.000
		OA	15	0.009	0.037	0.266	0.152	4180.058	14	0.000
		OW + OA	21	0.000	0.204	0.530	0.367	11505.420	20	0.000
	Growth	OW	64	0.000	0.142	0.262	0.202	1949.847	63	0.000
		OA	50	0.000	0.054	0.125	0.089	975.592	49	0.000
		OW + OA	74	0.000	0.161	0.265	0.213	1633.971	73	0.000
	Metabolism	OW	47	0.000	0.320	0.501	0.411	896.096	46	0.000
		OA	34	0.000	0.174	0.316	0.245	272.869	33	0.000
		OW + OA	50	0.000	0.359	0.575	0.467	773.317	49	0.000
	Physiology	OW	72	0.000	0.164	0.264	0.214	673.650	71	0.000
		OA	62	0.000	0.084	0.159	0.121	411.529	61	0.000
		OW + OA	78	0.000	0.206	0.325	0.266	690.083	77	0.000
	Reproduction	OW	7	0.015	0.076	0.711	0.394	68.539	6	0.000
		OA	6	0.060	-0.005	0.273	0.134	29.372	5	0.000
		OW + OA	10	0.001	0.142	0.546	0.344	64.343	9	0.000
	Survival	OW	31	0.000	0.227	0.535	0.381	1225.577	30	0.000
		OA	25	0.000	0.058	0.197	0.127	882.205	24	0.000
		OW + OA	35	0.000	0.332	0.700	0.516	3243.957	34	0.000
Invertebrate	Behavior	OW	40	0.000	0.287	0.484	0.385	589.977	47	0.000
		OA	40	0.000	0.271	0.502	0.386	255.778	45	0.000
		OW + OA	49	0.000	0.282	0.452	0.367	754.881	58	0.000
	Biomechanics	OW	18	0.005	0.056	0.325	0.190	0.726	2	0.696
		OA	23	0.000	0.082	0.275	0.178	8.896	8	0.351
		OW + OA	24	0.006	0.066	0.403	0.234	38.638	8	0.000
	Biodiversity	OW	3	0.000	0.156	0.341	0.249	153.510	22	0.000
		OA	9	0.000	0.105	0.221	0.163	132.988	28	0.000
		OW + OA	9	0.000	0.183	0.361	0.272	325.220	32	0.000
	Calcification	OW	43	0.000	0.163	0.296	0.229	294.945	51	0.000
		OA	51	0.000	0.221	0.450	0.335	32083.800	60	0.000
		OW + OA	56	0.000	0.254	0.431	0.343	885.197	69	0.000
	Development	OW	72	0.000	0.277	0.489	0.383	7911.451	83	0.000
		OA	108	0.000	0.176	0.357	0.266	12212.950	118	0.000
		OW + OA	129	0.000	0.400	0.581	0.491	9752.666	153	0.000
	Growth	OW	92	0.000	0.217	0.341	0.279	1853.062	117	0.000
		OA	124	0.000	0.151	0.227	0.189	2634.171	146	0.000
		OW + OA	148	0.000	0.230	0.332	0.281	5123.488	189	0.000
	Metabolism	OW	41	0.000	0.346	0.568	0.457	380.680	52	0.000
		OA	41	0.000	0.169	0.273	0.221	94.032	53	0.000
		OW + OA	51	0.000	0.418	0.655	0.536	828.539	66	0.000
	Physiology	OW	77	0.000	0.233	0.421	0.327	2181.863	84	0.000
		OA	82	0.000	0.190	0.332	0.261	1651.435	91	0.000
		OW + OA	94	0.000	0.257	0.414	0.335	2355.827	103	0.000
	Reproduction	OW	52	0.000	0.103	0.231	0.167	552.694	58	0.000
		OA	55	0.000	0.099	0.281	0.190	445.037	61	0.000
		OW + OA	92	0.000	0.140	0.273	0.206	953.802	110	0.000
	Survival	OW	64	0.000	0.199	0.396	0.297	727.443	67	0.000
		OA	110	0.000	0.198	0.319	0.258	5686.556	114	0.000
		OW + OA	120	0.000	0.359	0.530	0.445	2454.179	124	0.000

Supplementary Table 5: Model heterogeneity and residual heterogeneity. Model heterogeneity and residual heterogeneity associated with the effect of climate driver intensity level on the deviation in biological responses of fish and invertebrates (one-way ANOVA) to ocean warming (OW), ocean acidification (OA), and their combination (OW+OA).

				Model heterogeneity			Residual heterogeneity		
**taxa**	**biological response**	**driver**	**n**	**Qm**	**df**	**p-value**	**Qe**	**df**	**p-value**
Fish	Behavior	OW	40	1.05	2	0.59	154	37	3.35E-16
		OA	35	4.09	2	0.13	168	32	2.44E-20
		OW+OA	45	0.46	2	0.8	128	31	8.24E-14
	Development	OW	19	0.17	2	0.92	1836	16	0.00E+00
		OA	15	0.91	2	0.64	1539	12	1.24e-322
		OW+OA	21	5.15	1	0.02	174	6	3.69E-34
	Growth	OW	64	2.49	2	0.29	1710	61	3.06e-317
		OW+OA	74	0.14	2	0.93	1206	44	5.13E-224
		OA	50	2.71	2	0.26	947	47	5.74E-168
	Metabolism	OW	47	3.78	2	0.15	503	44	3.53E-79
		OA	34	0.15	1	0.7	270	31	1.76E-39
		OW+OA	50	2.26	1	0.13	186	24	1.32E-26
	Physiology	OW	72	0.49	2	0.78	672	69	1.10E-99
		OW+OA	78	0.57	2	0.75	340	44	1.94E-47
		OA	62	0.79	2	0.67	397	59	1.34E-51
	Reproduction	OW	7	0.68	1	0.41	48	4	4.45E-09
		OA	6	1.28	2	0.53	9	3	2.62E-02
		OW+OA	10	NA	NA	NA	NA	NA	NA
	Survival	OW	31	10.2	2	0.01	542	28	1.17E-96
		OA	25	0.8	2	0.67	879	22	8.78E-172
		OW+OA	35	3.83	1	0.05	2629	19	0.00E+00
Invertebrate	Behavior	OA	59	0.44	1	0.51	256	22	3.60E-41
		OW	46	0.18	2	0.91	204	43	8.20E-23
		OW+OA	48	7	2	0.03	249	45	5.58E-30
	Biomechanics	OA	29	4.88	2	0.09	64	26	5.48E-05
		OW	23	1.42	2	0.49	146	20	4.19E-21
		OW+OA	33	6.02	1	0.01	62	15	2.08E-07
	Biodiversity	OA	9	1.91	1	0.17	7	6	4.31E-01
		OW+OA	9	NA	NA	NA	NA	NA	NA
		OW	3	NA	NA	NA	NA	NA	NA
	Calcification	OA	61	3.25	2	0.2	17735	58	0.00E+00
		OW	52	0.2	2	0.9	228	49	6.41E-25
		OW+OA	70	4.26	2	0.12	471	33	5.32E-79
	Development	OA	119	0.01	2	1	12210	116	0.00E+00
		OW	84	3.41	2	0.18	5221	81	0.00E+00
		OW+OA	154	3.29	2	0.19	4567	62	0.00E+00
	Growth	OA	147	0.07	2	0.97	2516	144	0.00E+00
		OW	118	11.92	2	0	1831	115	1.05E-306
		OW+OA	190	2.9	2	0.23	2400	76	0.00E+00
	Metabolism	OA	54	0.38	2	0.83	86	51	1.64E-03
		OW	53	4.42	1	0.04	349	50	5.02E-46
		OW+OA	67	6.06	1	0.01	517	39	3.11E-84
	Physiology	OA	92	2.24	2	0.33	1644	89	1.54E-284
		OW	85	1.55	2	0.46	2174	82	0.00E+00
		OW+OA	104	0.78	1	0.38	677	48	2.14E-111
	Reproduction	OA	62	3.77	2	0.15	442	59	3.89E-60
		OW	59	3.49	2	0.18	534	56	4.24E-79
		OW+OA	111	1.49	2	0.47	85	37	1.34E-05
	Survival	OW+OA	125	4.33	2	0.11	304	42	2.00E-41
		OA	115	9.03	2	0.01	5190	112	0.00E+00
		OW	68	5.94	2	0.05	694	65	4.78E-106

Supplementary Table 6: Sensitivity analyses. Rosenthal safe number, associated Rosenthal threshold, trim and fill p-value and original p-value associated with significant directional effect sizes (lnRR). OW = ocean warming, OA = ocean acidification.

taxa	biological response	driver	Rosenthal number	threshold	original p-value	trim p-value
fish	Development	OW	9934	105	0.001	0.001
		OW+OA	23357	115	0.000	0.000
	Growth	OA	2242	260	0.015	0.015
	Metabolism	OW	24009	245	0.000	0.000
		OW+OA	8360	260	0.000	0.000
	Reproduction	OW	102	45	0.015	0.015
	Survival	OW	11983	165	0.009	0.009
		OW+OA	45688	185	0.000	0.000
invertebrate	Behavior	OA	1196	240	0.007	0.007
	Biomechanics	OA	350	155	0.016	0.002
		OW+OA	846	175	0.022	0.022
	Calcification	OA	29589	315	0.002	0.000
		OW+OA	2151	360	0.020	0.020
	Development	OA	18217	605	0.001	0.001
		OW+OA	117531	780	0.000	0.000
	Growth	OA	33580	745	0.000	0.000
		OW+OA	43172	960	0.001	0.001
	Metabolism	OA	6725	275	0.000	0.000
		OW+OA	13954	345	0.000	0.000
	Reproduction	OW	2422	320	0.010	0.010
		OW+OA	12853	565	0.000	0.000
	Survival	OW	3352	350	0.046	0.001
		OA	14050	585	0.000	0.000
		OW+OA	29322	635	0.000	0.000

Supplementary Table 7: Additional benthic species. Collection sites of additional sessile and gregarious or low-vagility benthic invertebrate species added to those used in Vargas et al.[46].

	**Species**	**Collection site**
Oysters	*Ostrea edulis*	low intertidal
Gastropods	*Austrocochlea concamerata*	low intertidal
	Littorina littorea	intertidal
	Phasianella australis	5-10 m depth
	Rissoa interrupta	low intertidal and shallow subtidal
	Rissoa parva	low intertidal and shallow subtidal
	Stylocheilus striatus	low intertidal and shallow subtidal
	Thalotia conica	1-2 m depth
Crustaceans	*Calliopius laeviusculus*	low intertidal and shallow subtidal
	Echinogammarus marinus	intertidal
	Elminius modestus	intertidal
	Gammarus locusta	1-5 m depth
	Hyas araneus	7-12 m depth
	Menippe mercenaria (larvae)	surface water
	Peramphithoe parmerong	1-3 m depth
	Semibalanus balanoides	intertidal
	Stenothoe monoculoides	intertidal
Corals	*Fungia fungites*	3-20 m depth
	Lithophyllon repanda	1-30 m depth
	Pocillopora damicornis	1-2 m depth
Mussels	*Scrobicularia plana*	intertidal
Moss animals	*Celleporaria nodulosa*	1-3 m depth
Sponge	*Aphrocallistes vastus*	scuba dive site
Worms	*Platynereis dumerilii*	low intertidal and shallow subtidal

Supplementary Table 8: Model heterogeneity and residual heterogeneity associated with the effect of climate driver intensity level on the biological responses of invertebrates to ocean acidification. Climate driver intensity levels were determined either using the experimental minus the control partial pressure of carbon dioxide (pCO_2; intensity = experimental – control pCO_2) as given in publications (top part of the table) or using an intensity index according to Vargas et al.[46] for which the experimental pCO_2 is subtracted from the upper extreme local environmental pCO_2 (intensity = experimental – extreme local environmental pCO_2; bottom part of the table).

	**biological response**	**Qm**	**df (Qm)**	**p-value (Qm)**	**Qe**	**df (Qe)**	**p-value (Qe)**
Intensity = experimental – control pCO_2 value							
directional response (lnRR)	Survival	5.73	1	0.017	148.88	37	0.000
	Biomechanics	3.90	2	0.142	27.17	13	0.004
	Behavior	0.26	2	0.879	165.01	18	0.000
	Calcification	0.32	2	0.851	165.41	25	0.000
	Development	0.84	2	0.657	487.40	20	0.000
	Growth	5.46	2	0.065	1150.24	56	0.000
	Physiology	4.78	2	0.091	448.82	30	0.000
	Reproduction	11.11	2	0.004	12.29	9	0.091
	Metabolism	3.25	2	0.197	34.88	14	0.000
deviation response (abs(lnRR))	Survival	2.66	1	0.103	133.16	37	0.000
	Biomechanics	9.34	2	0.009	15.83	13	0.147
	Behavior	14.48	2	0.001	23.06	18	0.112
	Calcification	0.50	2	0.778	68.92	25	0.000
	Development	0.49	2	0.782	478.69	20	0.000
	Growth	0.52	2	0.770	1078.34	56	0.000
	Physiology	3.68	2	0.159	388.51	30	0.000
	Reproduction	7.50	2	0.024	11.40	9	0.122
	Metabolism	0.24	2	0.889	17.15	14	0.144
intensity = experimental – extreme local environmental pCO_2							
directional response (lnRR)	Survival	1.20	2	0.549	148.92	37	0.000
	Biomechanics	3.15	2	0.207	37.31	13	0.000
	Behavior	0.07	2	0.965	139.55	18	0.000
	Calcification	1.07	2	0.584	134.85	25	0.000
	Development	2.03	2	0.362	489.94	20	0.000
	Growth	2.10	2	0.350	876.02	56	0.000
	Physiology	3.60	2	0.165	450.24	30	0.000
	Reproduction	3.63	2	0.163	16.89	9	0.018
	Metabolism	0.50	2	0.778	44.22	14	0.000
deviation response (abs(lnRR))	Biomechanics	2.23	2	0.328	24.41	13	0.011
	Behavior	2.36	2	0.307	36.84	18	0.002
	Calcification	1.51	2	0.469	83.76	25	0.000
	Development	2.30	2	0.317	481.94	20	0.000
	Growth	5.46	2	0.065	779.60	56	0.000
	Physiology	2.24	2	0.326	391.93	30	0.000
	Reproduction	1.07	2	0.585	16.64	9	0.020
	Metabolism	2.03	2	0.363	15.56	14	0.212
