# Supplementary material for: Hidden impacts of ocean warming and acidification on biological responses of marine animals revealed through meta-analysis
Source: Nat Commun. 2024 Apr 3;15:2885. doi: 10.1038/s41467-024-47064-3 (PMC10991405; doi:10.1038/s41467-024-47064-3)
Supplement: Supplementary file 3 — Description of Additional Supplementary Files [file 41467_2024_47064_MOESM3_ESM.pdf]

Description of Additional Supplementary Files

Supplementary Data 1: Forest plots and funnel plots associated with the meta-analyses models tested in this study.

Supplementary Data 2: Choice of metrics selected in the meta-analysis when multiple metrics falling under the same biological response category were measured in one study. Studies in which only one metric per category was measured are not listed.

Supplementary Data 3: Classification of metrics into the ten categories of biological responses considered, and into the three categories of effects of a metric's increase on fitness (positive, negative, or ambiguous).

Supplementary Data 4: Experts' votes on the effect of a metric's increase on animal fitness. Based on the expertise of five co-authors, metrics were classified according to whether an increase in that metrics' value was considered beneficial (1), detrimental (-1), ambiguous (0), or unknown (NA).

Supplementary Data 5: Summary statistics associated with the meta-analyses on the directional change in biological responses of fish and invertebrates to different intensity levels of ocean warming (OW), ocean acidification (OA) and their combination (OW + OA). Values provided are the p-value, degree of freedom (df), between-study heterogeneity (Qt), and p-value associated with between-study heterogeneity (pQt), for each calculated effect size shown in Figure 5.

Supplementary Data 6: Summary statistics associated with the meta-analyses on the deviation change in biological responses of fish and invertebrates to different intensity levels of ocean warming (OW), ocean acidification (OA) and their combination (OW + OA). Values provided are the p-value, degree of freedom (df), between-study heterogeneity (Qt), and p-value associated with between-study heterogeneity (pQt), for each calculated effect size shown in Figure 7.
