# Supplementary material for: Hidden impacts of ocean warming and acidification on biological responses of marine animals revealed through meta-analysis
Source: Nat Commun. 2024 Apr 3;15:2885. doi: 10.1038/s41467-024-47064-3 (PMC10991405; doi:10.1038/s41467-024-47064-3)
Supplement: Supplementary file 4 — Supplementary Data 1 [file 41467_2024_47064_MOESM4_ESM.docx]

Forest and Funnel plots

## Sensitivity analysis table
sensitivity <- tibble(taxa=character(), stressor=character(), metric=character(),Rosenthal=numeric(),threshold=numeric(),pval=numeric(),trimfill=numeric())

## Inverts, Metabolism
InvertMetabolism <- Inverts[Category %in% c("Routine respiration","Aerobic scope respiration")]
InvertMetabolism$Category <- c("Metabolism")
InvertMet <- MA_TpH("inverts","Metabolism",InvertMetabolism,sensitivity)

##
## Random-Effects Model (k = 53; tau^2 estimator: REML)
##
## tau^2 (estimated amount of total heterogeneity): 0.2056 (SE = 0.0478)
## tau (square root of estimated tau^2 value): 0.4534
## I^2 (total heterogeneity / total variability): 93.18%
## H^2 (total variability / sampling variability): 14.66
##
## Test for Heterogeneity:
## Q(df = 52) = 598.8352, p-val < .0001
##
## Model Results:
##
## estimate se zval pval ci.lb ci.ub ​
## 0.3599 0.0684 5.2614 <.0001 0.2258 0.4939 ***
##
## ---
## Signif. codes: 0 '***' 0.001 '**' 0.01 '*' 0.05 '.' 0.1 ' ' 1


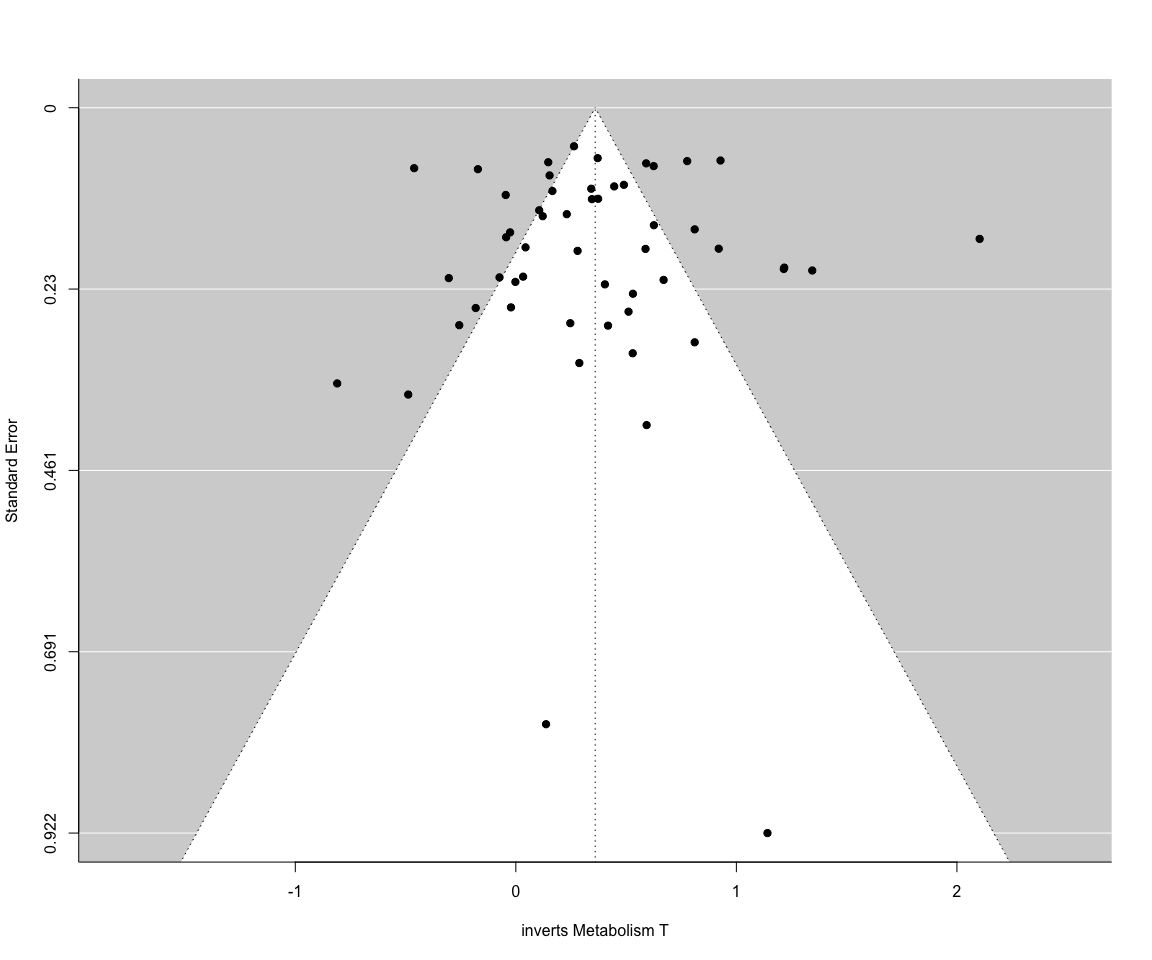

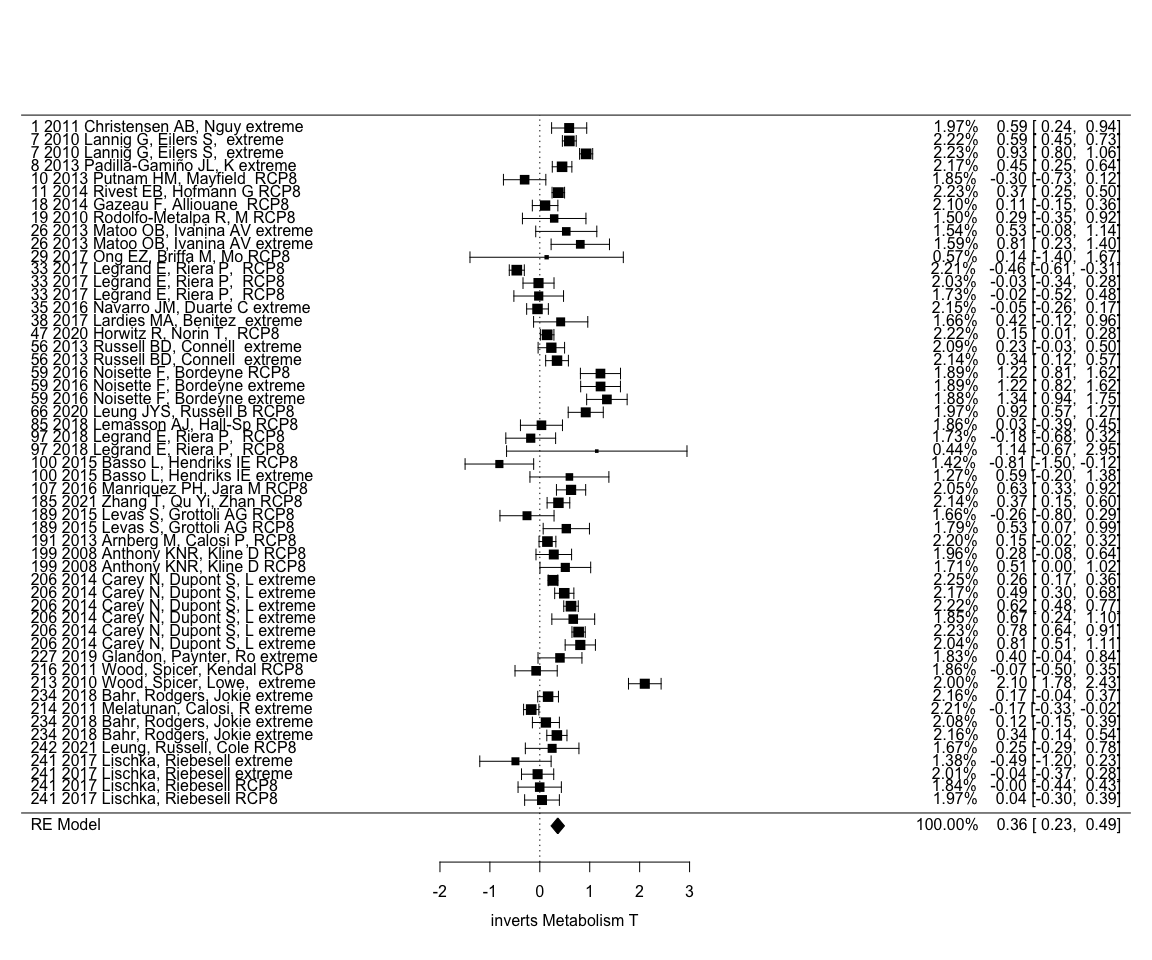


##
## Random-Effects Model (k = 54; tau^2 estimator: REML)
##
## tau^2 (estimated amount of total heterogeneity): 0.0630 (SE = 0.0181)
## tau (square root of estimated tau^2 value): 0.2511
## I^2 (total heterogeneity / total variability): 79.25%
## H^2 (total variability / sampling variability): 4.82
##
## Test for Heterogeneity:
## Q(df = 53) = 199.0926, p-val < .0001
##
## Model Results:
##
## estimate se zval pval ci.lb ci.ub ​
## 0.0482 0.0426 1.1307 0.2582 -0.0353 0.1317
##
## ---
## Signif. codes: 0 '***' 0.001 '**' 0.01 '*' 0.05 '.' 0.1 ' ' 1


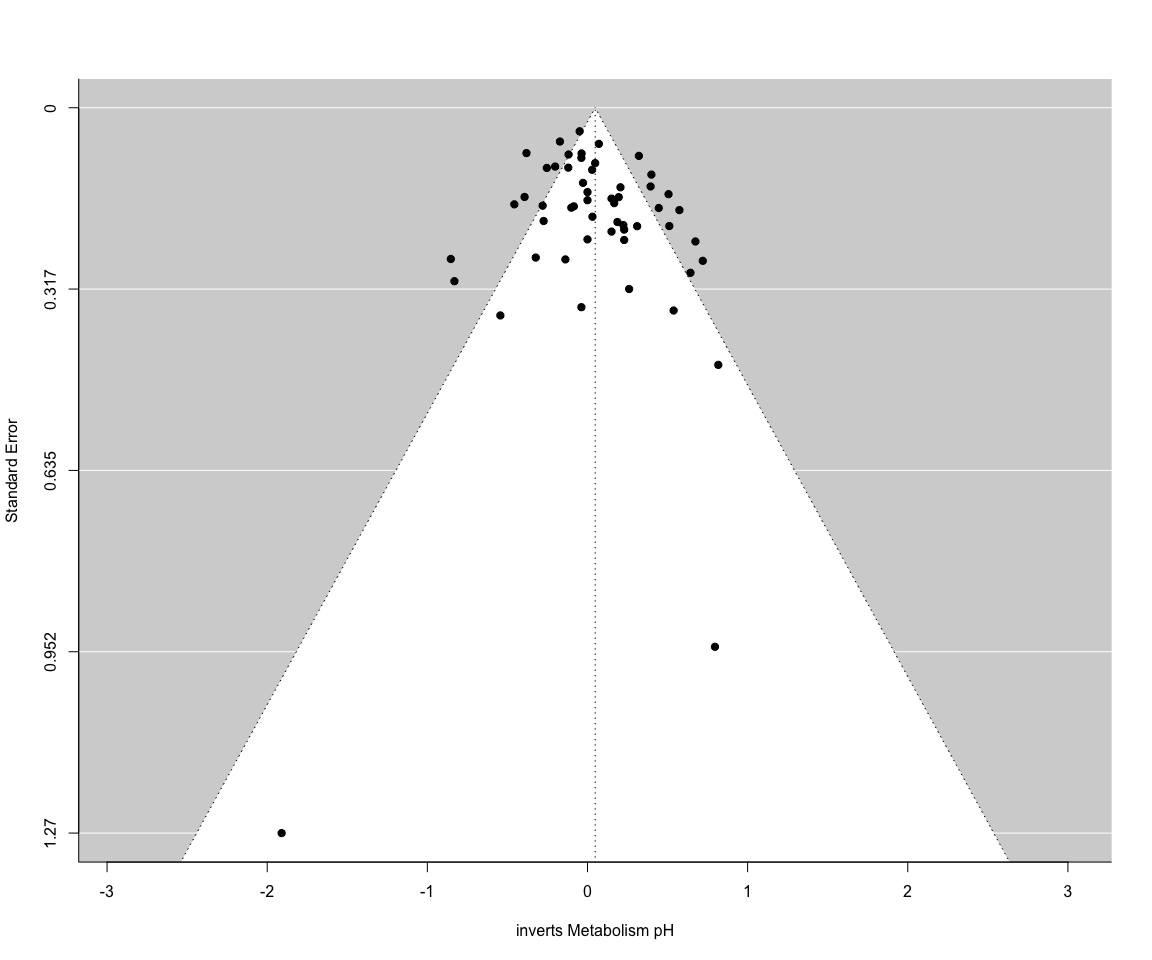

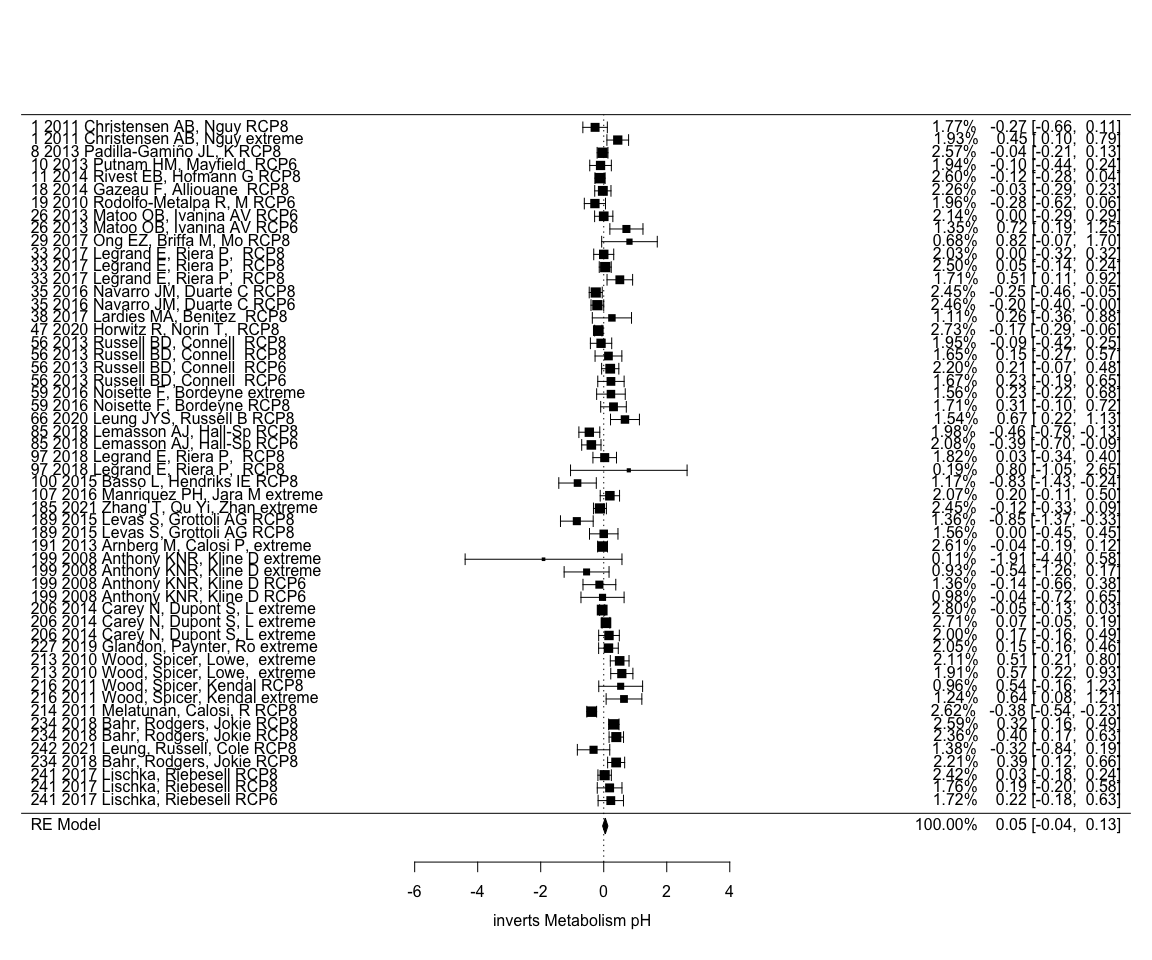


##
## Random-Effects Model (k = 67; tau^2 estimator: REML)
##
## tau^2 (estimated amount of total heterogeneity): 0.2853 (SE = 0.0566)
## tau (square root of estimated tau^2 value): 0.5341
## I^2 (total heterogeneity / total variability): 94.63%
## H^2 (total variability / sampling variability): 18.62
##
## Test for Heterogeneity:
## Q(df = 66) = 1112.4834, p-val < .0001
##
## Model Results:
##
## estimate se zval pval ci.lb ci.ub ​
## 0.4343 0.0705 6.1602 <.0001 0.2961 0.5725 ***
##
## ---
## Signif. codes: 0 '***' 0.001 '**' 0.01 '*' 0.05 '.' 0.1 ' ' 1


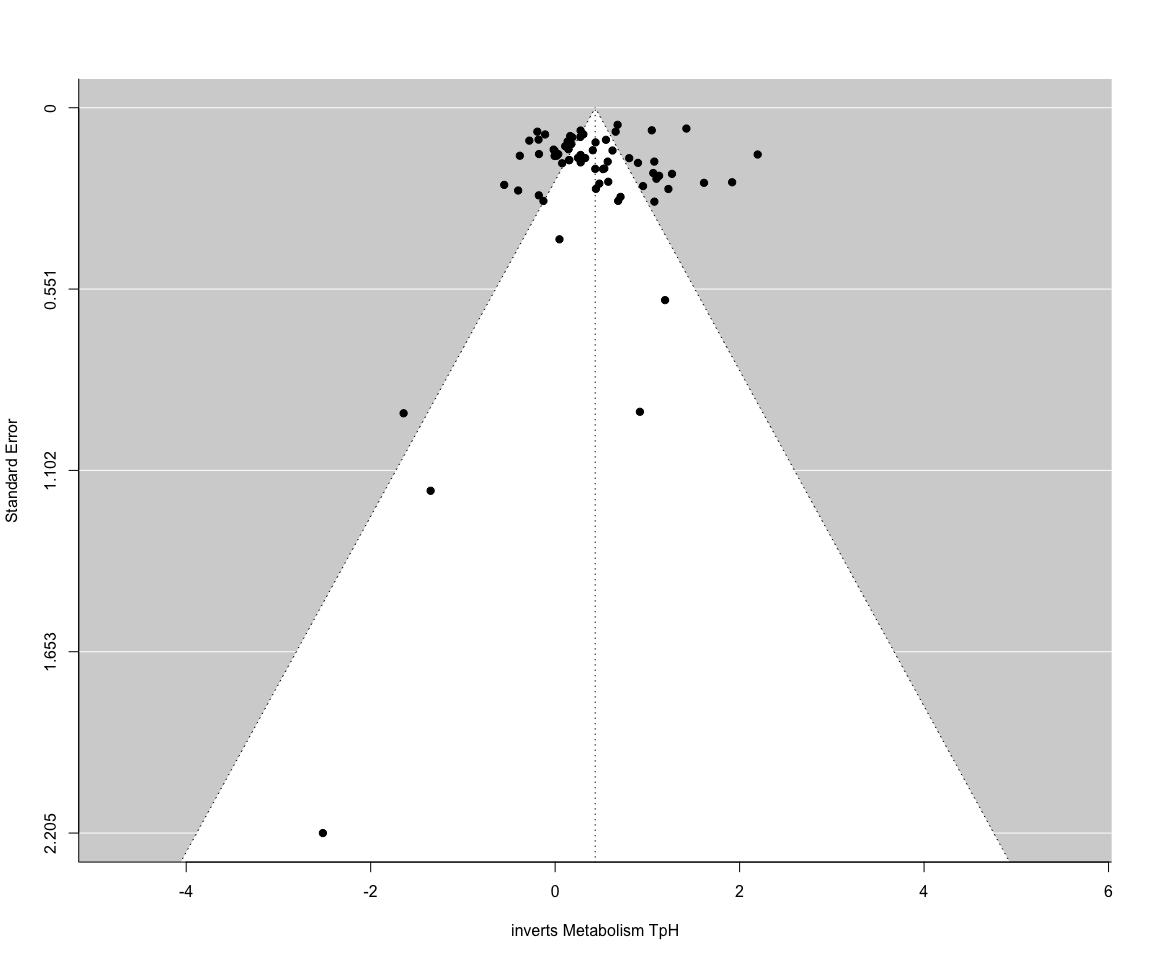

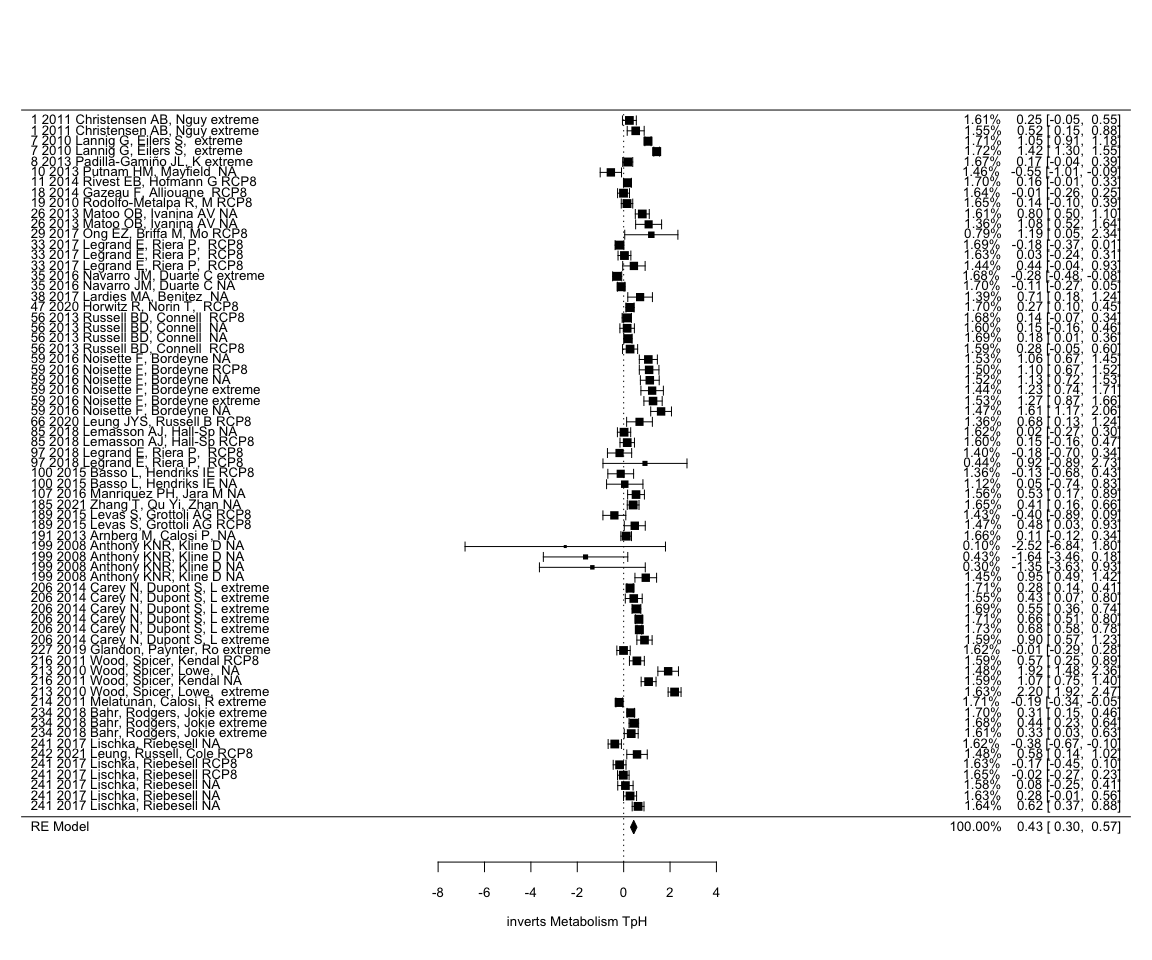


Abs_InvertMet <- MA_TpH_abs("inverts","Metabolism",InvertMetabolism)

## Fish, Metabolism
FishMetabolism <- Fish[Category %in% c("Routine respiration","Aerobic scope respiration")]
FishMetabolism$Category <- c("Metabolism")
FishMet <- MA_TpH("fish","Metabolism",FishMetabolism,sensitivity)

## Random-Effects Model (k = 47; tau^2 estimator: REML)
##
## tau^2 (estimated amount of total heterogeneity): 0.1594 (SE = 0.0366)
## tau (square root of estimated tau^2 value): 0.3993
## I^2 (total heterogeneity / total variability): 98.83%
## H^2 (total variability / sampling variability): 85.32
##
## Test for Heterogeneity:
## Q(df = 46) = 1088.2175, p-val < .0001
##
## Model Results:
##
## estimate se zval pval ci.lb ci.ub ​
## 0.3298 0.0613 5.3835 <.0001 0.2097 0.4499 ***
##
## ---
## Signif. codes: 0 '***' 0.001 '**' 0.01 '*' 0.05 '.' 0.1 ' ' 1


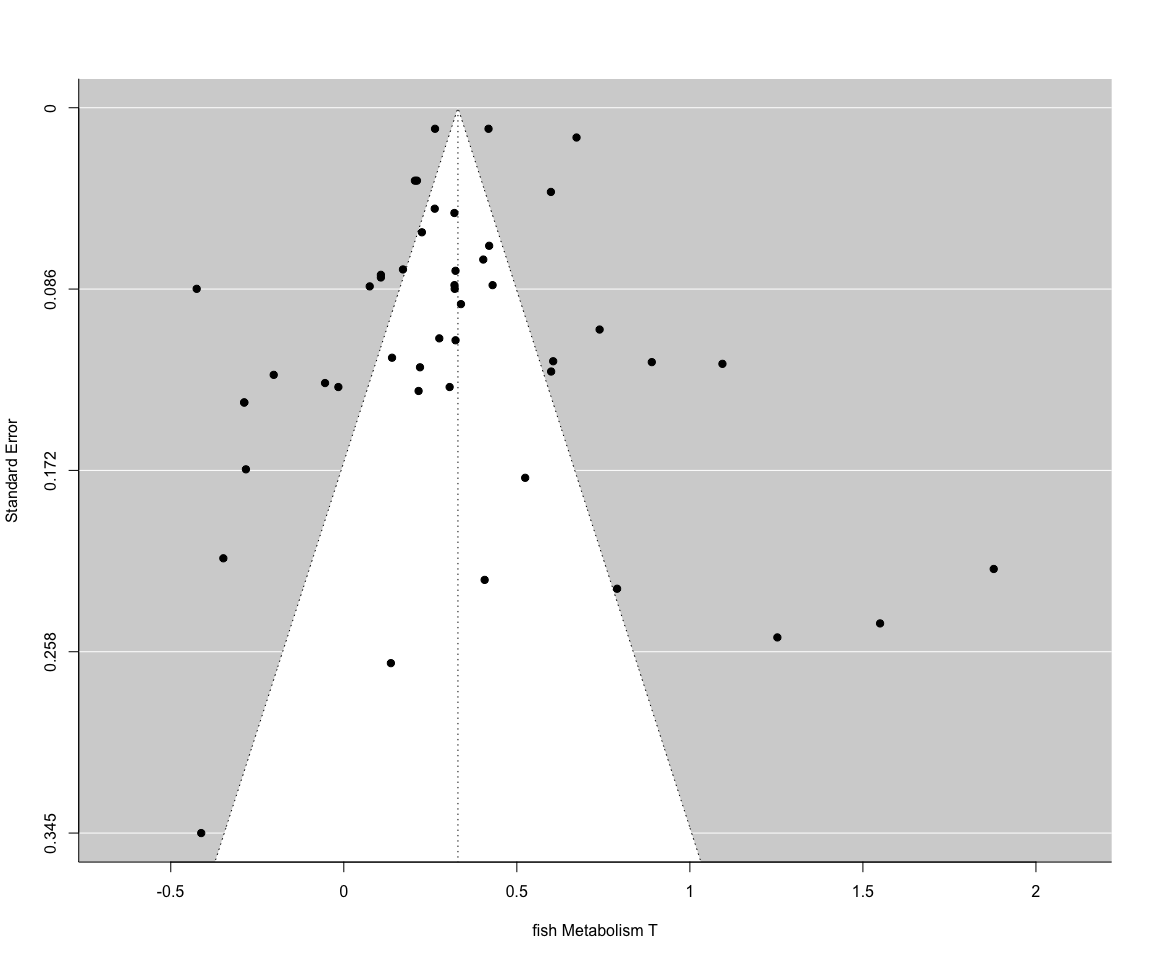

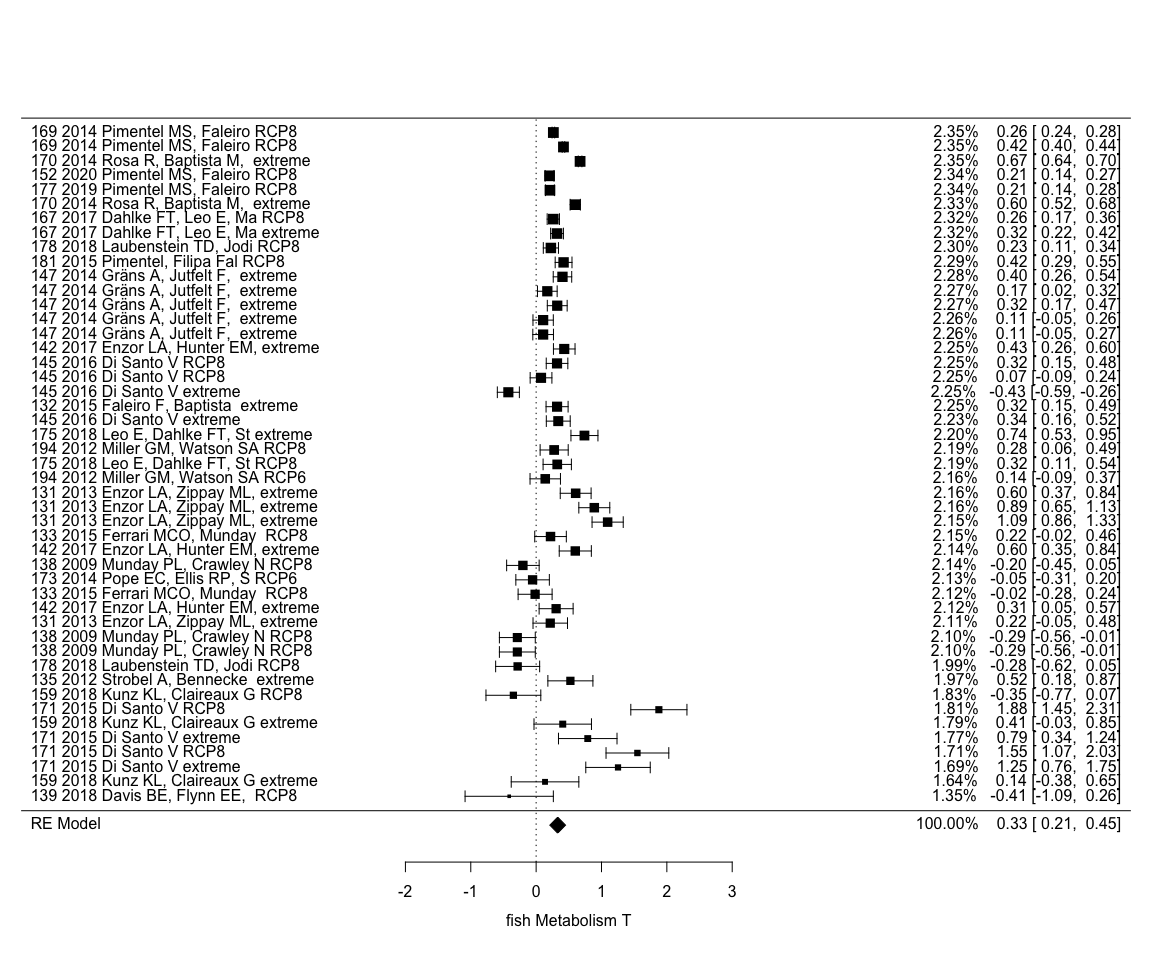


##
## Random-Effects Model (k = 34; tau^2 estimator: REML)
##
## tau^2 (estimated amount of total heterogeneity): 0.0992 (SE = 0.0290)
## tau (square root of estimated tau^2 value): 0.3150
## I^2 (total heterogeneity / total variability): 98.54%
## H^2 (total variability / sampling variability): 68.59
##
## Test for Heterogeneity:
## Q(df = 33) = 587.5758, p-val < .0001
##
## Model Results:
##
## estimate se zval pval ci.lb ci.ub ​
## -0.0112 0.0594 -0.1887 0.8503 -0.1277 0.1052
##
## ---
## Signif. codes: 0 '***' 0.001 '**' 0.01 '*' 0.05 '.' 0.1 ' ' 1


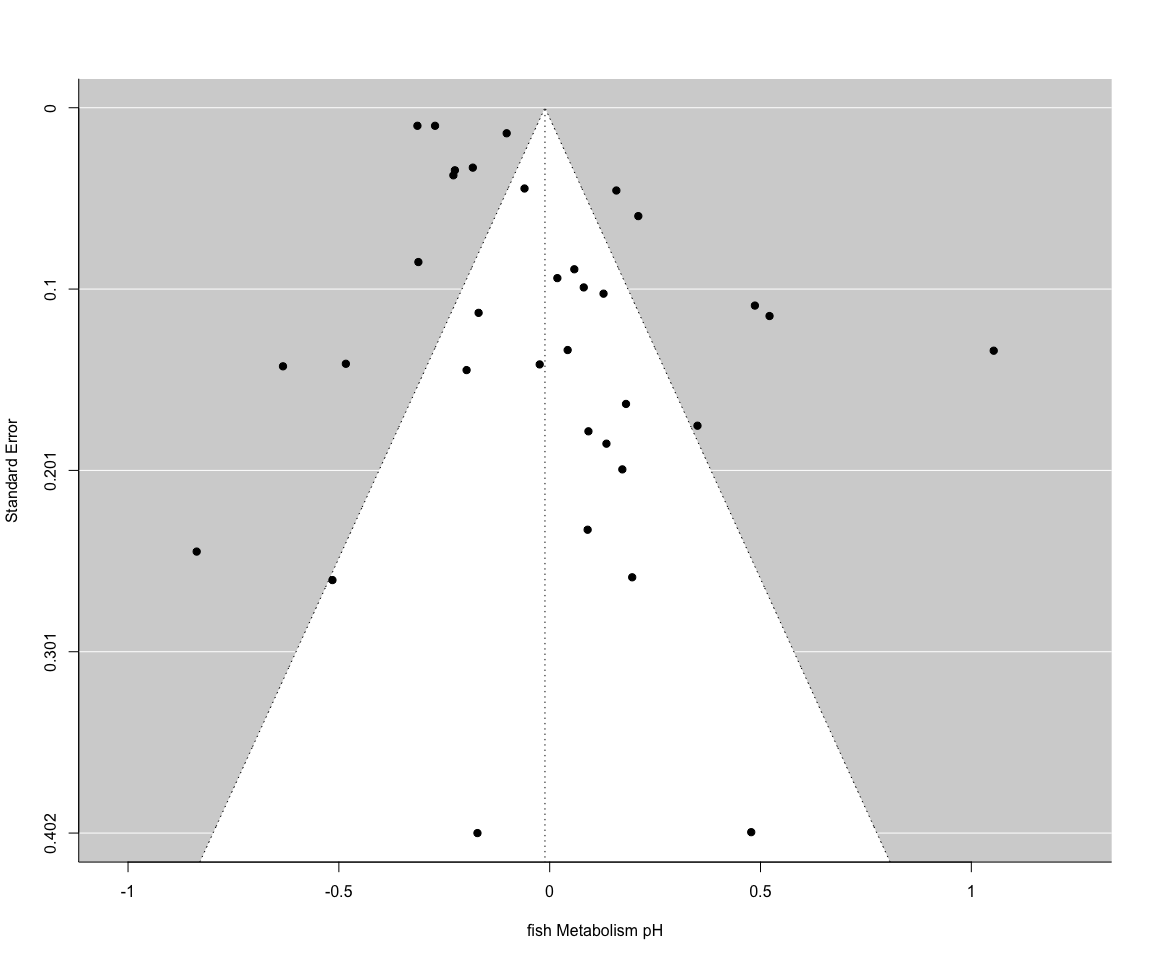

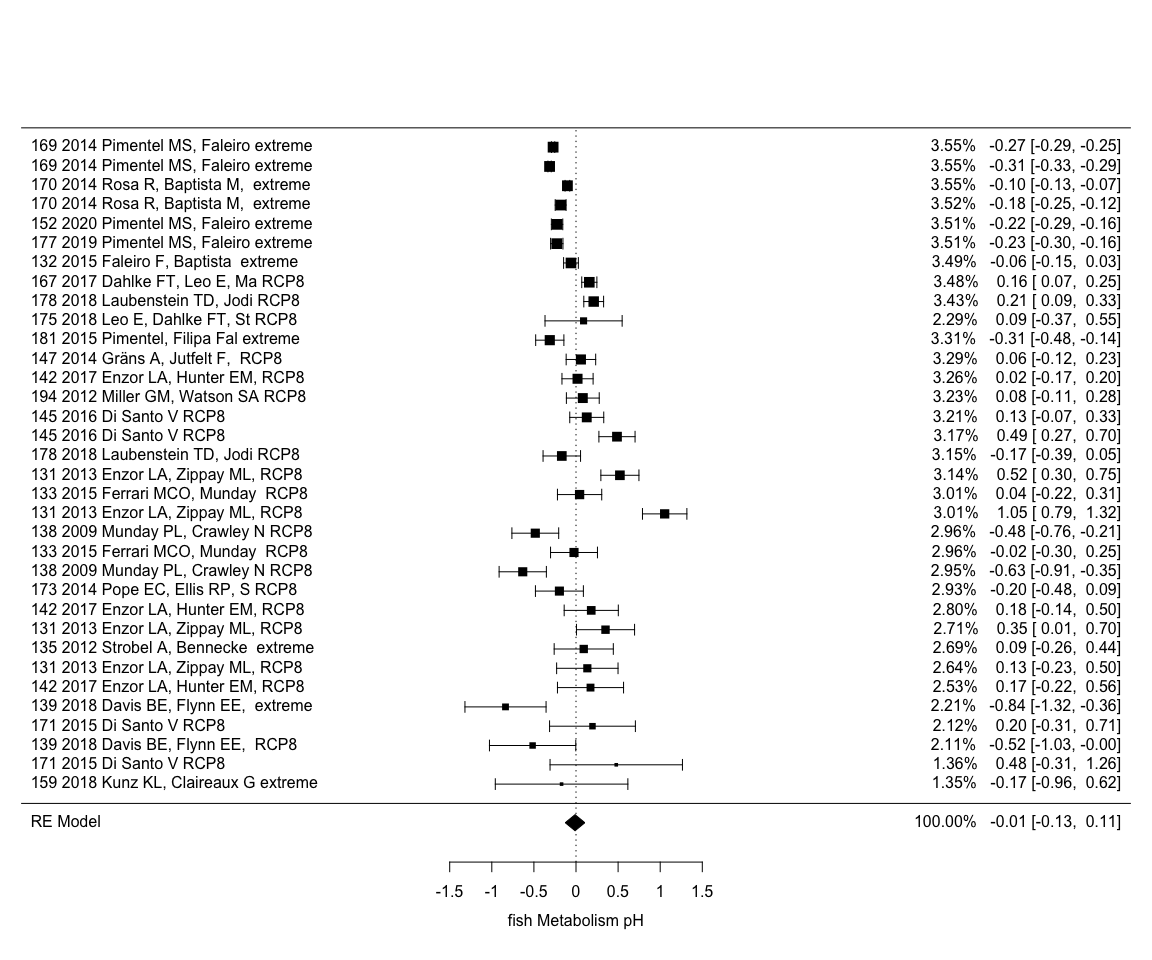


##
## Random-Effects Model (k = 50; tau^2 estimator: REML)
##
## tau^2 (estimated amount of total heterogeneity): 0.2894 (SE = 0.0622)
## tau (square root of estimated tau^2 value): 0.5380
## I^2 (total heterogeneity / total variability): 99.25%
## H^2 (total variability / sampling variability): 132.50
##
## Test for Heterogeneity:
## Q(df = 49) = 983.1153, p-val < .0001
##
## Model Results:
##
## estimate se zval pval ci.lb ci.ub ​
## 0.2918 0.0786 3.7134 0.0002 0.1378 0.4458 ***
##
## ---
## Signif. codes: 0 '***' 0.001 '**' 0.01 '*' 0.05 '.' 0.1 ' ' 1


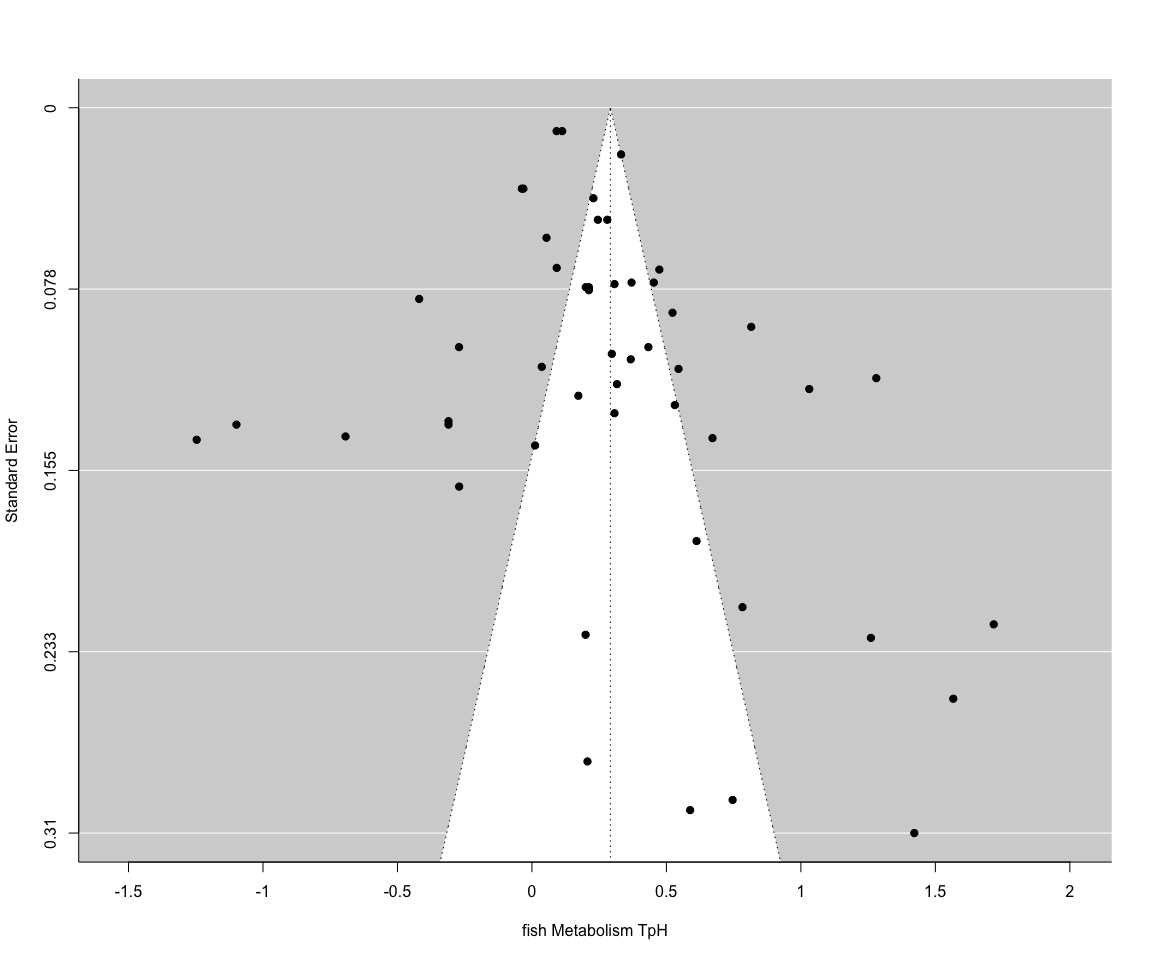

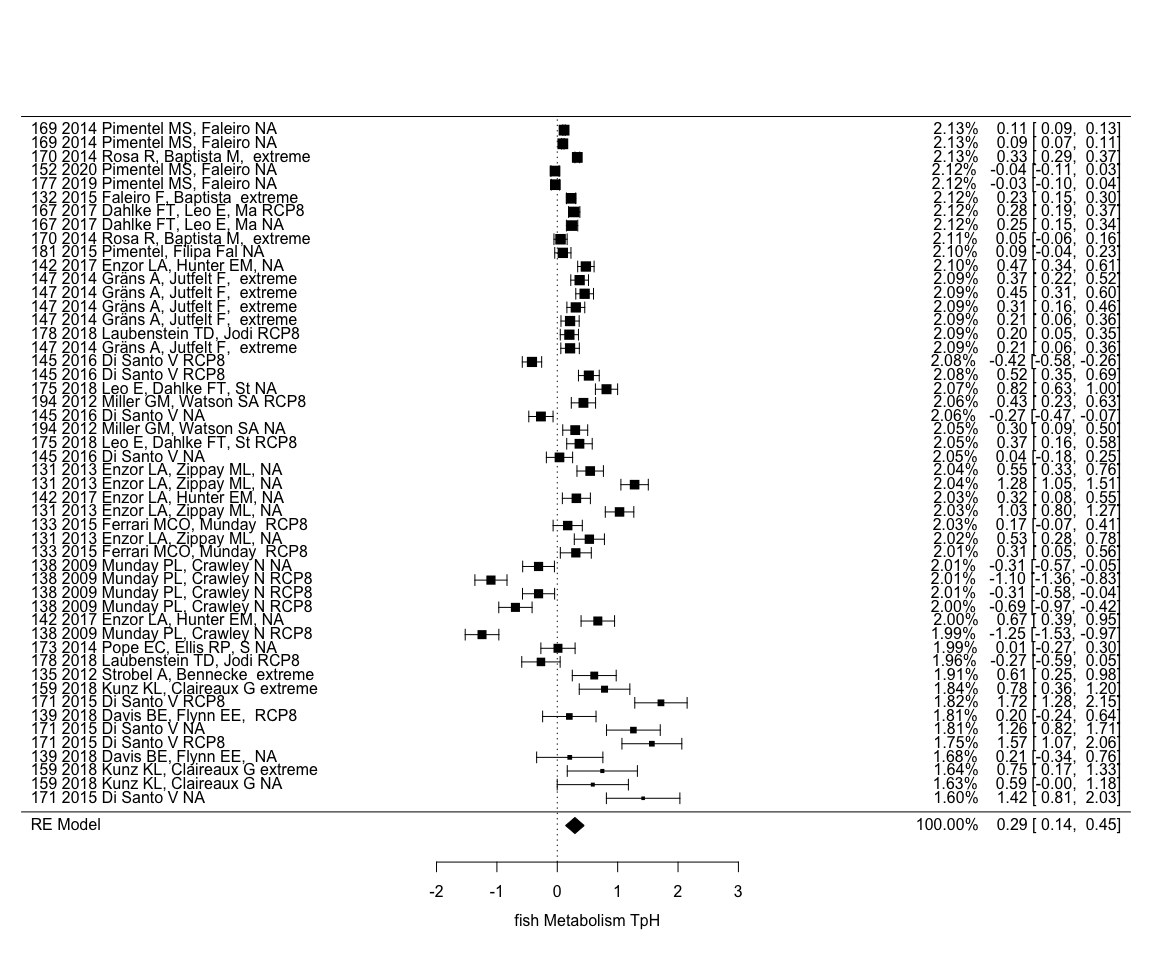


Abs_FishMet <- MA_TpH_abs("fish","Metabolism",FishMetabolism)

## Invertebrates, Calcification
InvertCalci <- MA_TpH("inverts","Calcification", Inverts,sensitivity)

## Random-Effects Model (k = 52; tau^2 estimator: REML)
##
## tau^2 (estimated amount of total heterogeneity): 0.0759 (SE = 0.0210)
## tau (square root of estimated tau^2 value): 0.2754
## I^2 (total heterogeneity / total variability): 97.45%
## H^2 (total variability / sampling variability): 39.14
##
## Test for Heterogeneity:
## Q(df = 51) = 389.0232, p-val < .0001
##
## Model Results:
##
## estimate se zval pval ci.lb ci.ub ​
## 0.0825 0.0473 1.7464 0.0807 -0.0101 0.1752 .
##
## ---
## Signif. codes: 0 '***' 0.001 '**' 0.01 '*' 0.05 '.' 0.1 ' ' 1


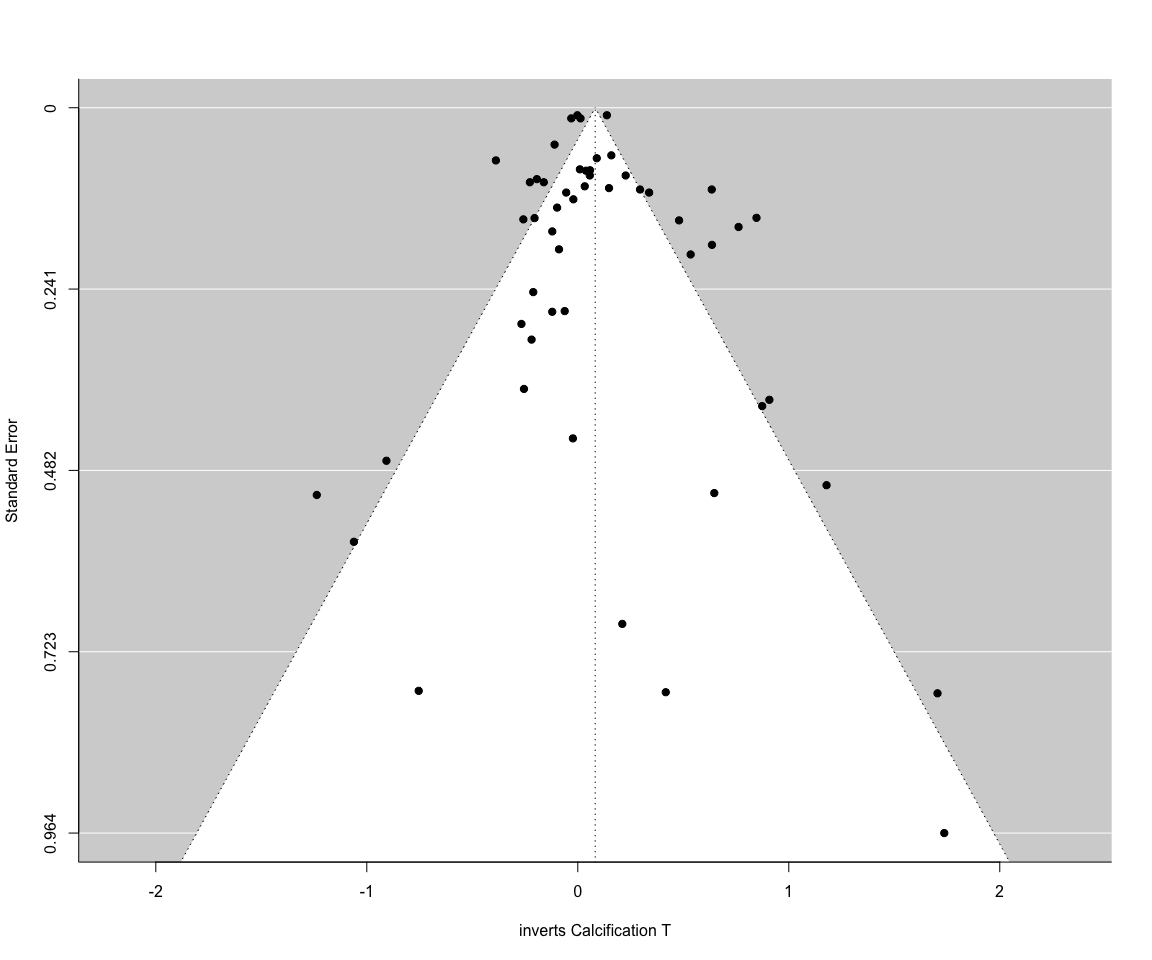

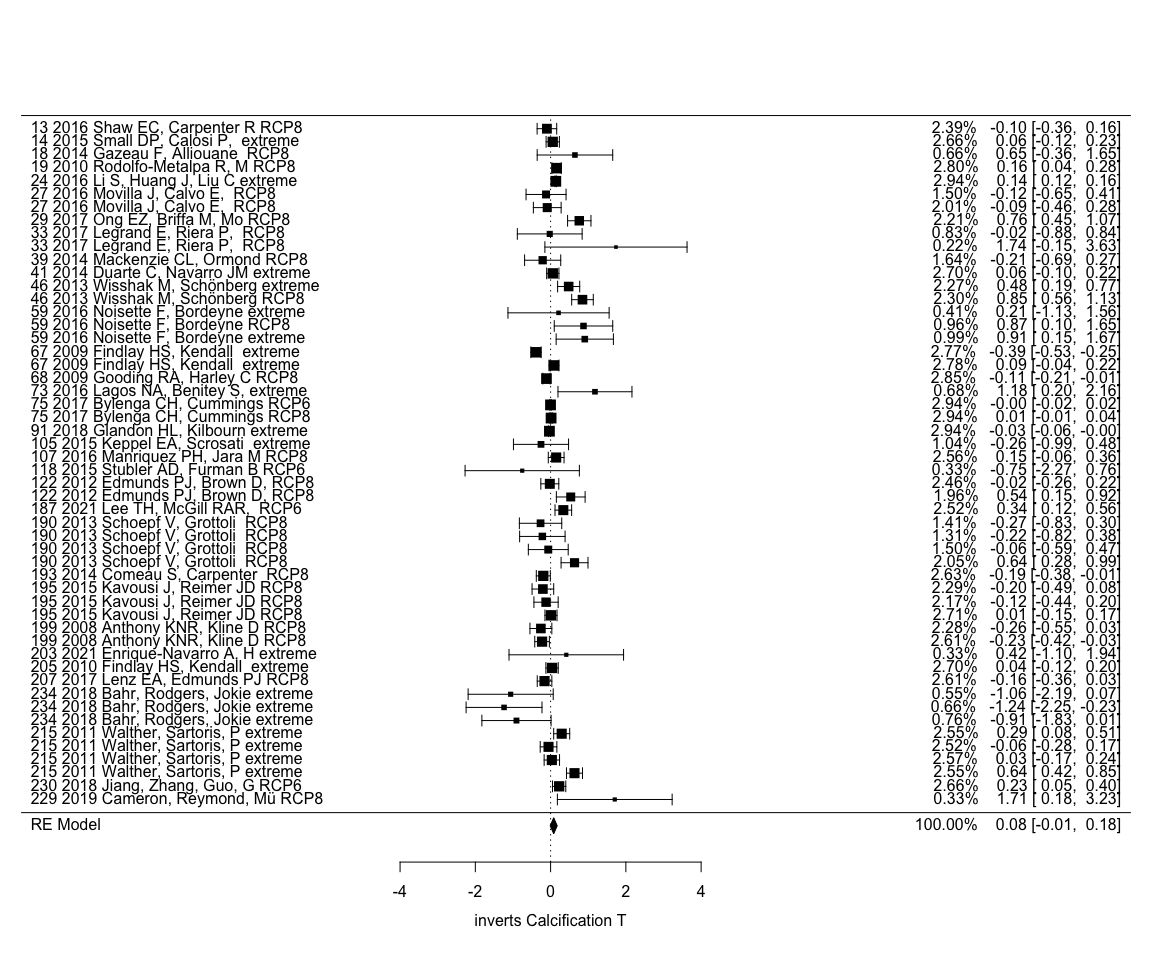


##
## Random-Effects Model (k = 61; tau^2 estimator: REML)
##
## tau^2 (estimated amount of total heterogeneity): 0.2112 (SE = 0.0461)
## tau (square root of estimated tau^2 value): 0.4596
## I^2 (total heterogeneity / total variability): 99.07%
## H^2 (total variability / sampling variability): 107.90
##
## Test for Heterogeneity:
## Q(df = 60) = 34599.7198, p-val < .0001
##
## Model Results:
##
## estimate se zval pval ci.lb ci.ub ​
## -0.2039 0.0655 -3.1125 0.0019 -0.3323 -0.0755 **
##
## ---
## Signif. codes: 0 '***' 0.001 '**' 0.01 '*' 0.05 '.' 0.1 ' ' 1


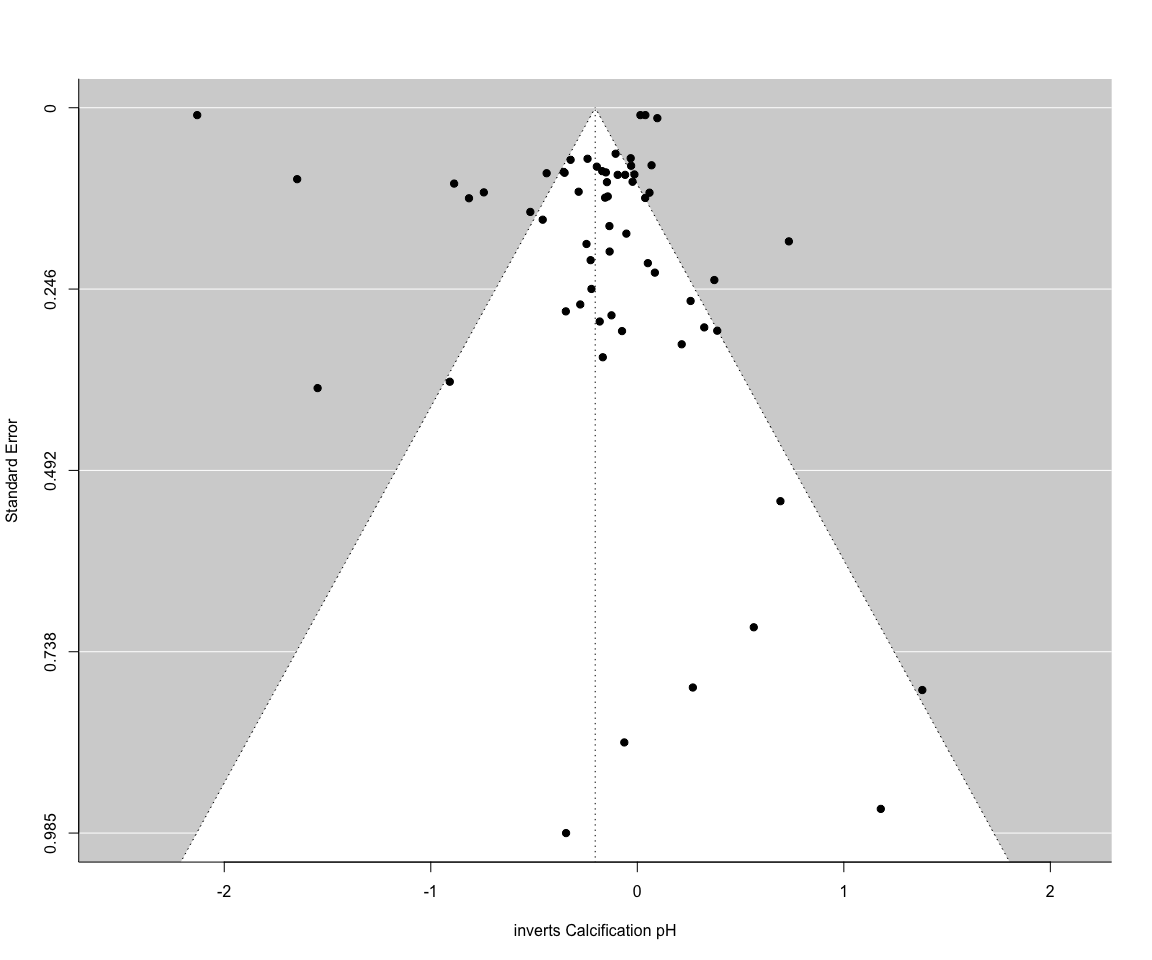

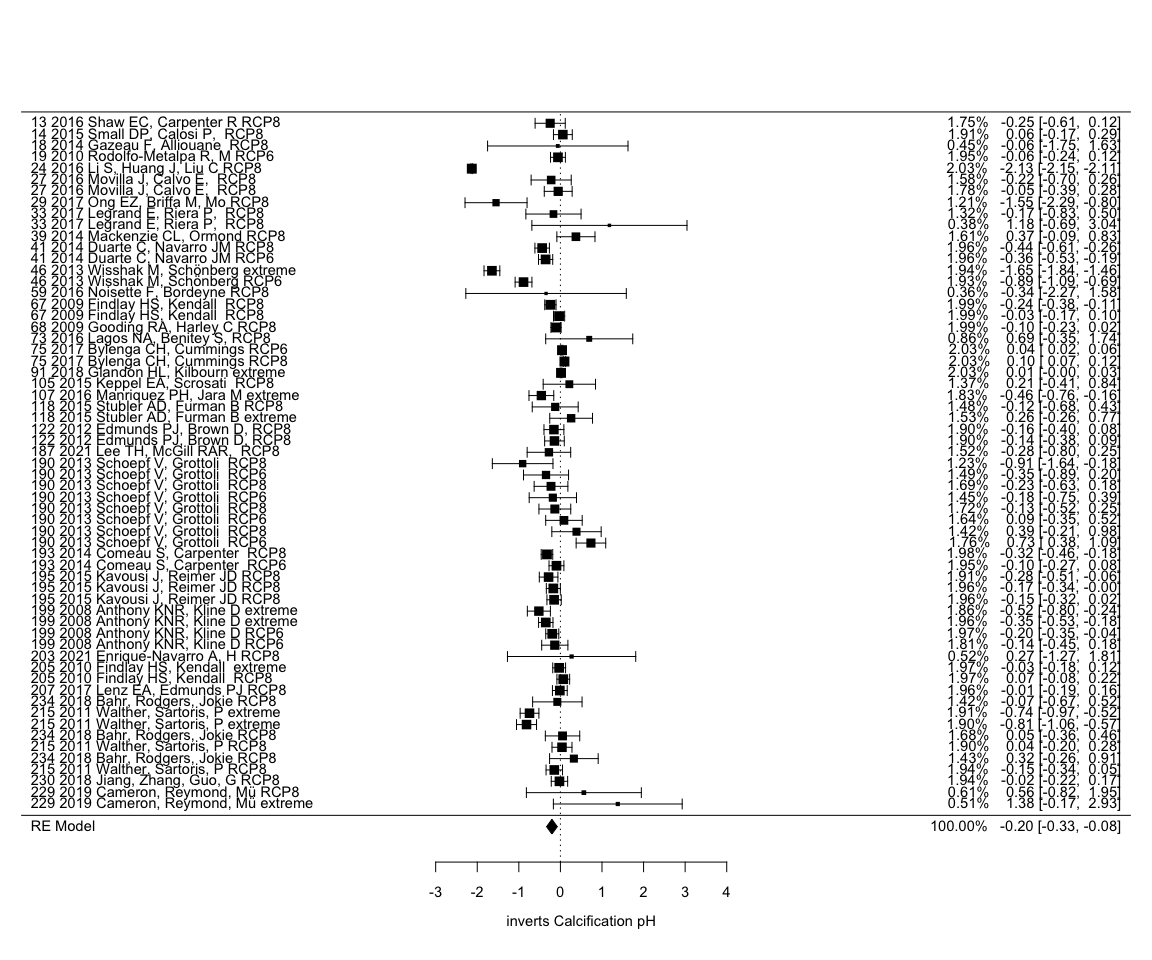


##
## Random-Effects Model (k = 70; tau^2 estimator: REML)
##
## tau^2 (estimated amount of total heterogeneity): 0.1788 (SE = 0.0379)
## tau (square root of estimated tau^2 value): 0.4228
## I^2 (total heterogeneity / total variability): 99.00%
## H^2 (total variability / sampling variability): 100.45
##
## Test for Heterogeneity:
## Q(df = 69) = 1317.0460, p-val < .0001
##
## Model Results:
##
## estimate se zval pval ci.lb ci.ub ​
## -0.1351 0.0581 -2.3247 0.0201 -0.2490 -0.0212 *
##
## ---
## Signif. codes: 0 '***' 0.001 '**' 0.01 '*' 0.05 '.' 0.1 ' ' 1


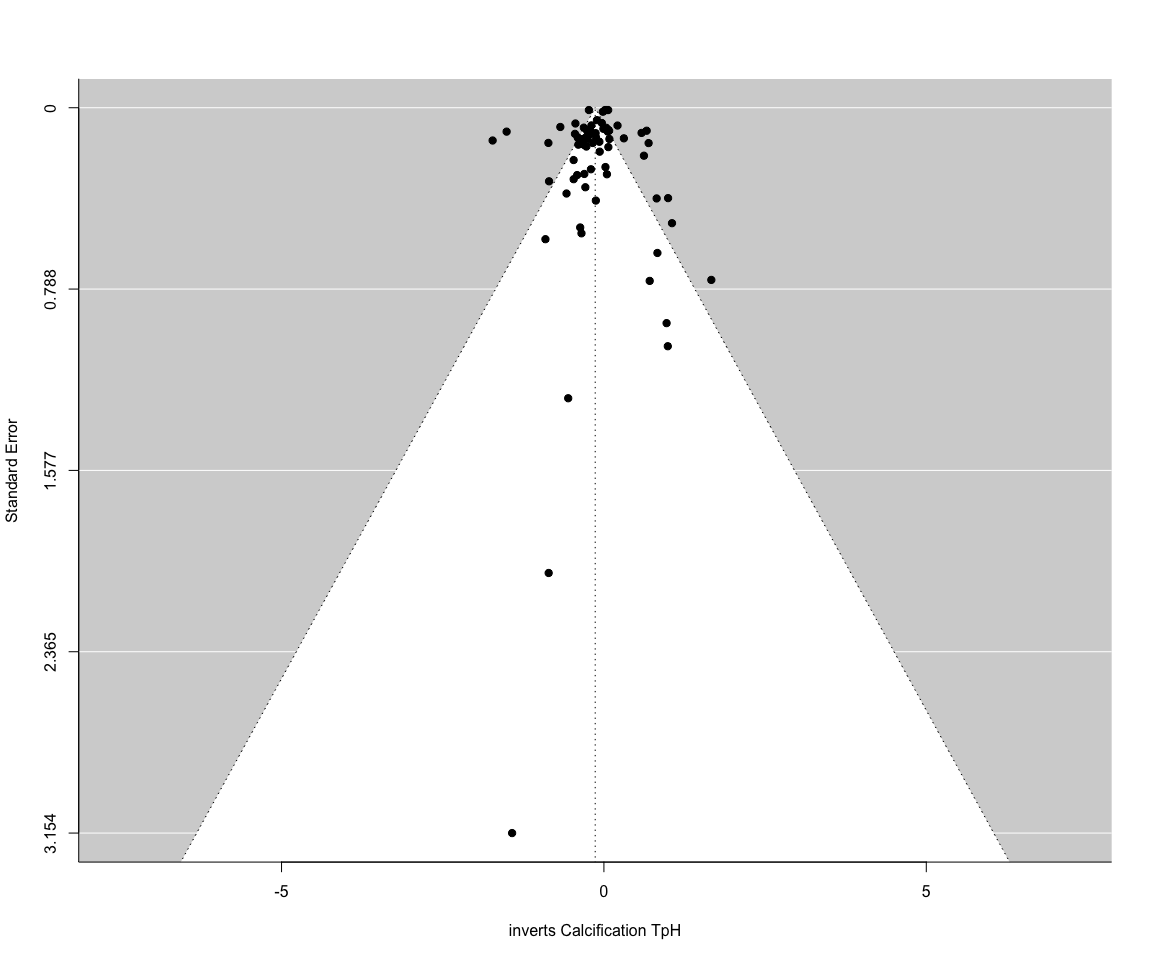

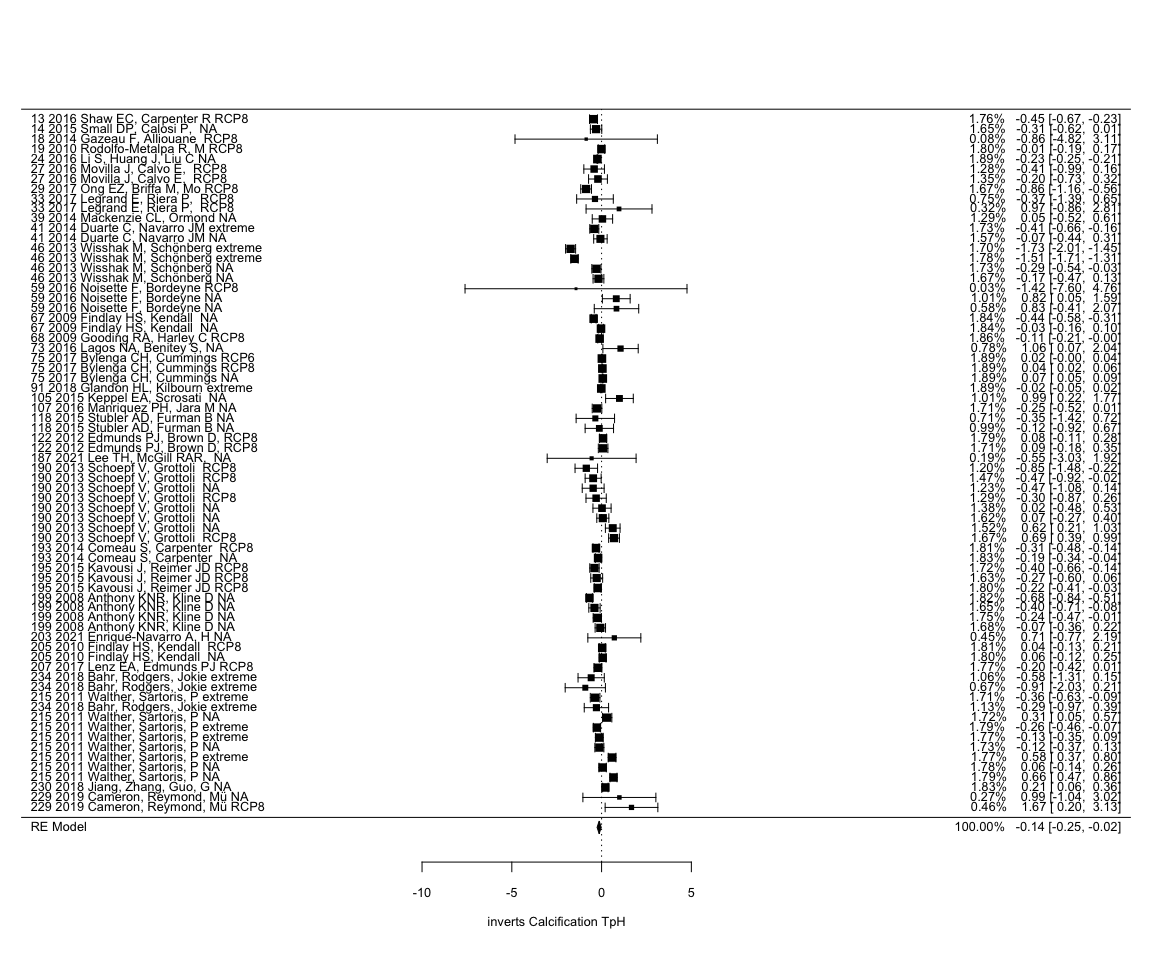


Abs_InvertCalci <- MA_TpH_abs("inverts","Calcification", Inverts)
## Fish, Calcification
FishCalci <- MA_TpH("fish","Calcification", Fish,sensitivity)

##
## Random-Effects Model (k = 4; tau^2 estimator: REML)
##
## tau^2 (estimated amount of total heterogeneity): 0.3002 (SE = 0.2523)
## tau (square root of estimated tau^2 value): 0.5479
## I^2 (total heterogeneity / total variability): 99.86%
## H^2 (total variability / sampling variability): 715.23
##
## Test for Heterogeneity:
## Q(df = 3) = 4107.2296, p-val < .0001
##
## Model Results:
##
## estimate se zval pval ci.lb ci.ub ​
## 0.3592 0.2780 1.2924 0.1962 -0.1856 0.9040
##
## ---
## Signif. codes: 0 '***' 0.001 '**' 0.01 '*' 0.05 '.' 0.1 ' ' 1


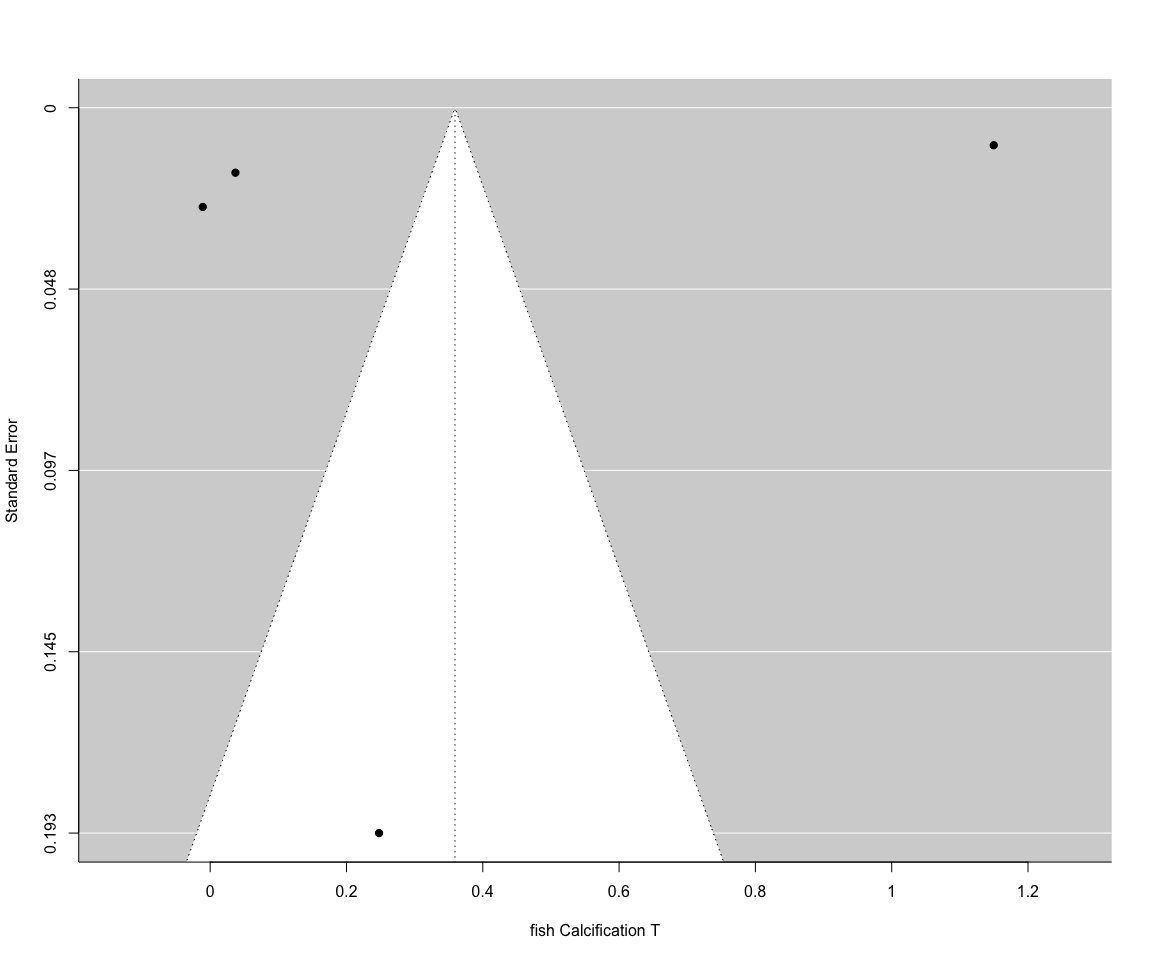

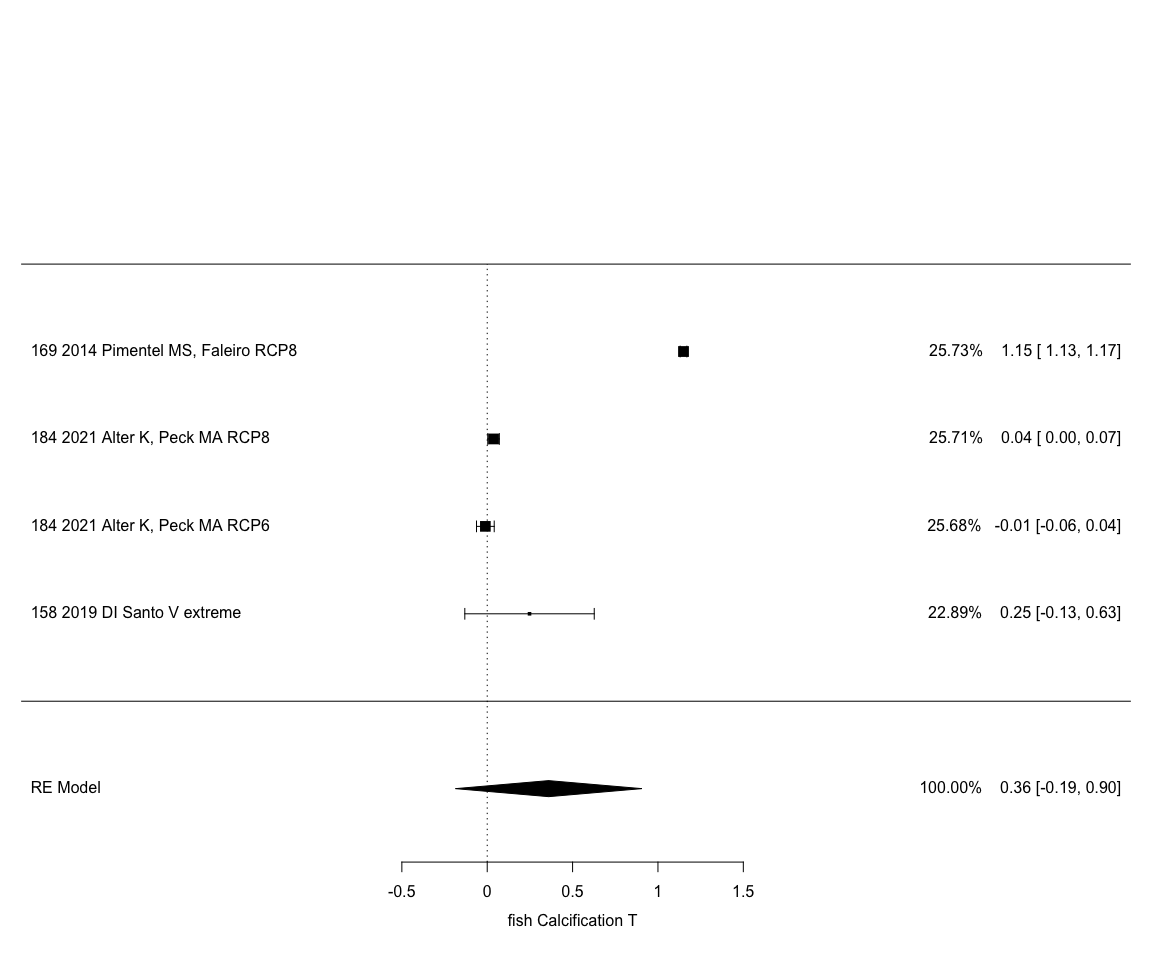


##
## Random-Effects Model (k = 3; tau^2 estimator: REML)
##
## tau^2 (estimated amount of total heterogeneity): 0.1139 (SE = 0.1241)
## tau (square root of estimated tau^2 value): 0.3375
## I^2 (total heterogeneity / total variability): 99.57%
## H^2 (total variability / sampling variability): 231.53
##
## Test for Heterogeneity:
## Q(df = 2) = 236.2163, p-val < .0001
##
## Model Results:
##
## estimate se zval pval ci.lb ci.ub ​
## 0.3564 0.2031 1.7546 0.0793 -0.0417 0.7544 .
##
## ---
## Signif. codes: 0 '***' 0.001 '**' 0.01 '*' 0.05 '.' 0.1 ' ' 1


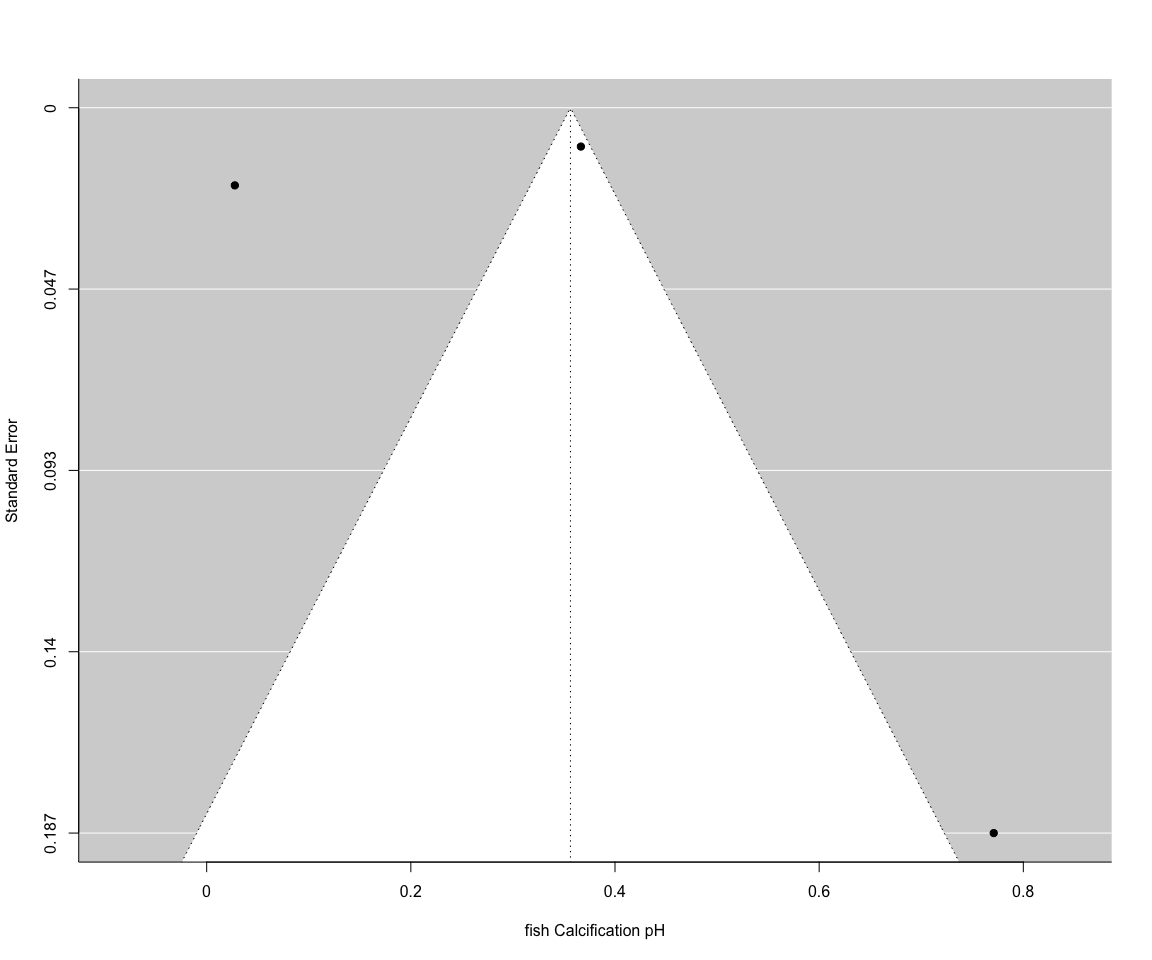

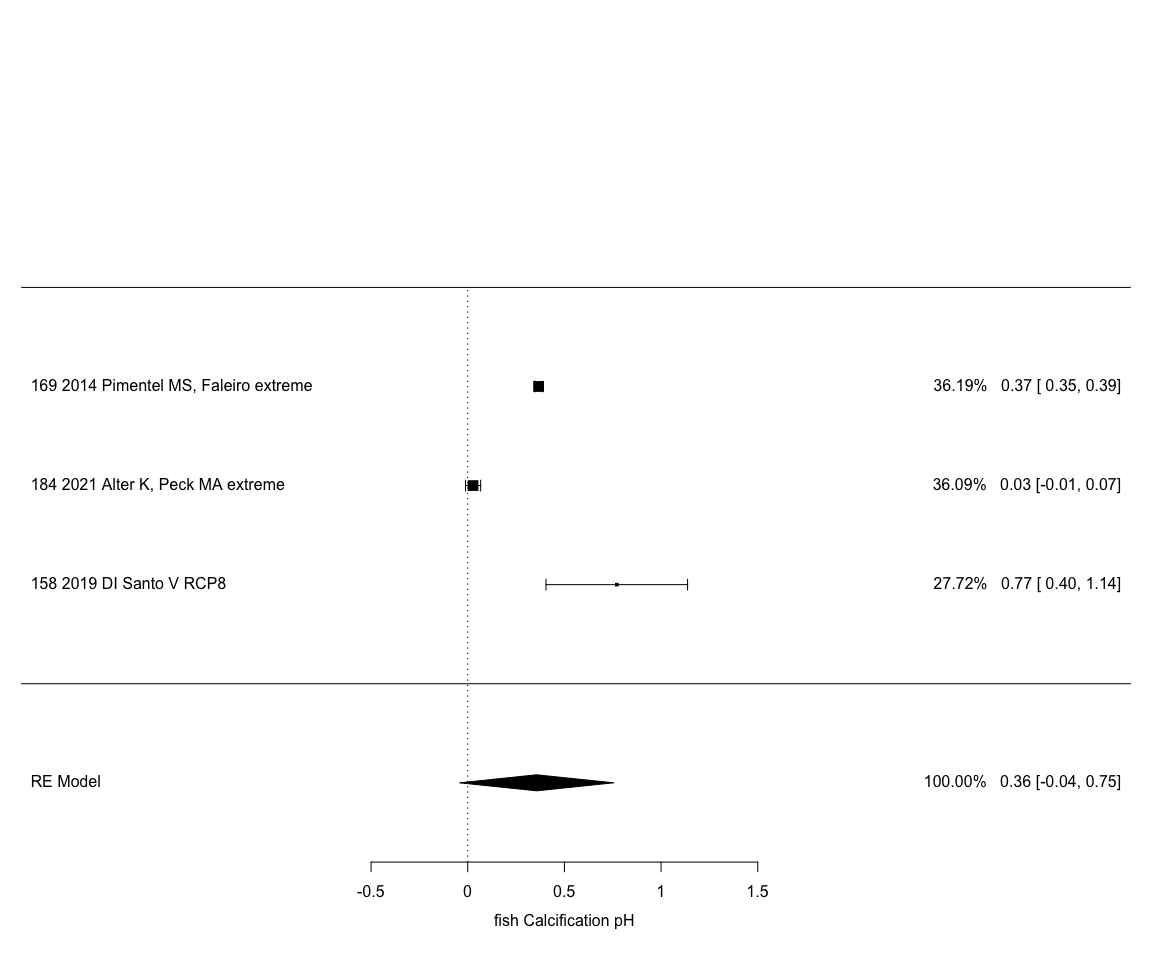


##
## Random-Effects Model (k = 4; tau^2 estimator: REML)
##
## tau^2 (estimated amount of total heterogeneity): 0.3778 (SE = 0.3143)
## tau (square root of estimated tau^2 value): 0.6147
## I^2 (total heterogeneity / total variability): 99.91%
## H^2 (total variability / sampling variability): 1067.74
##
## Test for Heterogeneity:
## Q(df = 3) = 5576.7308, p-val < .0001
##
## Model Results:
##
## estimate se zval pval ci.lb ci.ub ​
## 0.5707 0.3102 1.8397 0.0658 -0.0373 1.1788 .
##
## ---
## Signif. codes: 0 '***' 0.001 '**' 0.01 '*' 0.05 '.' 0.1 ' ' 1


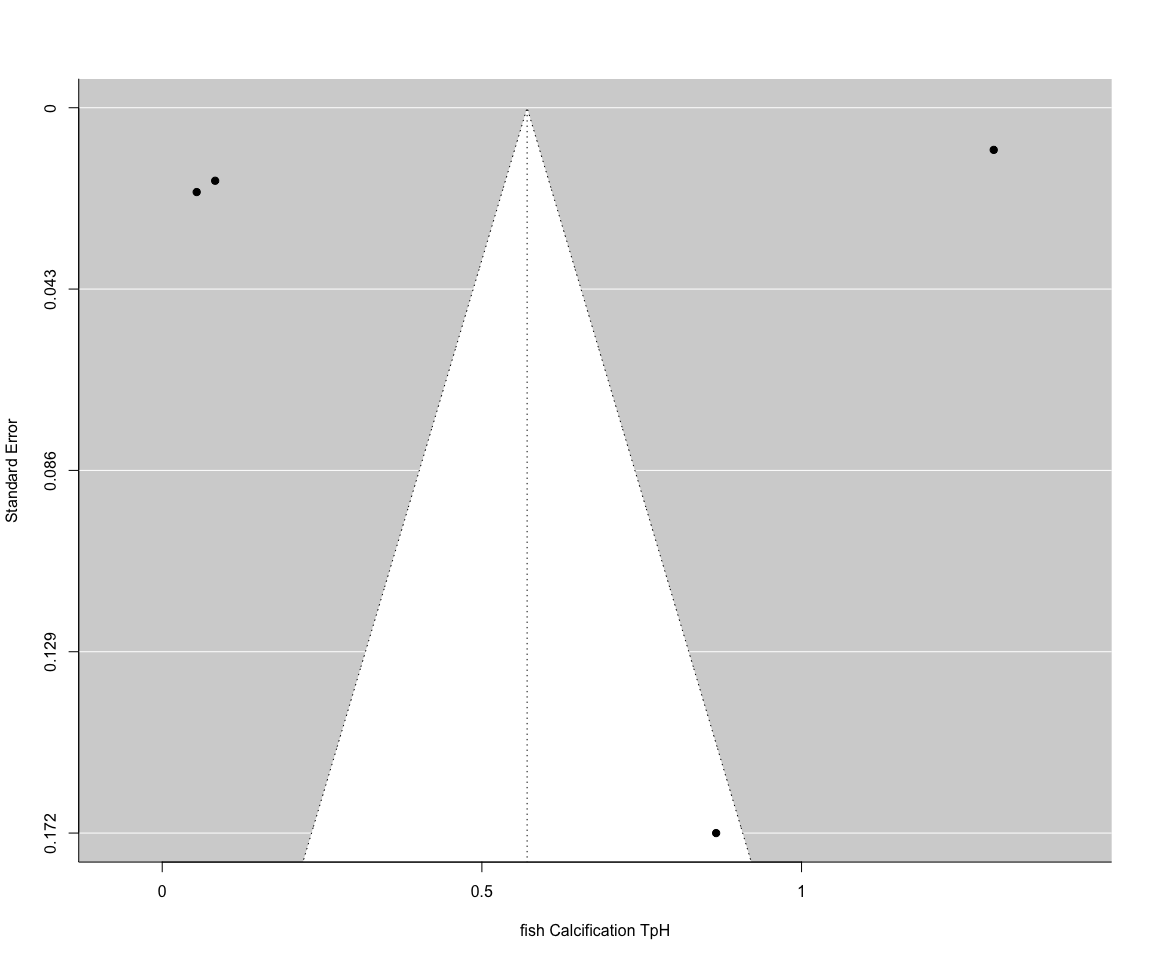

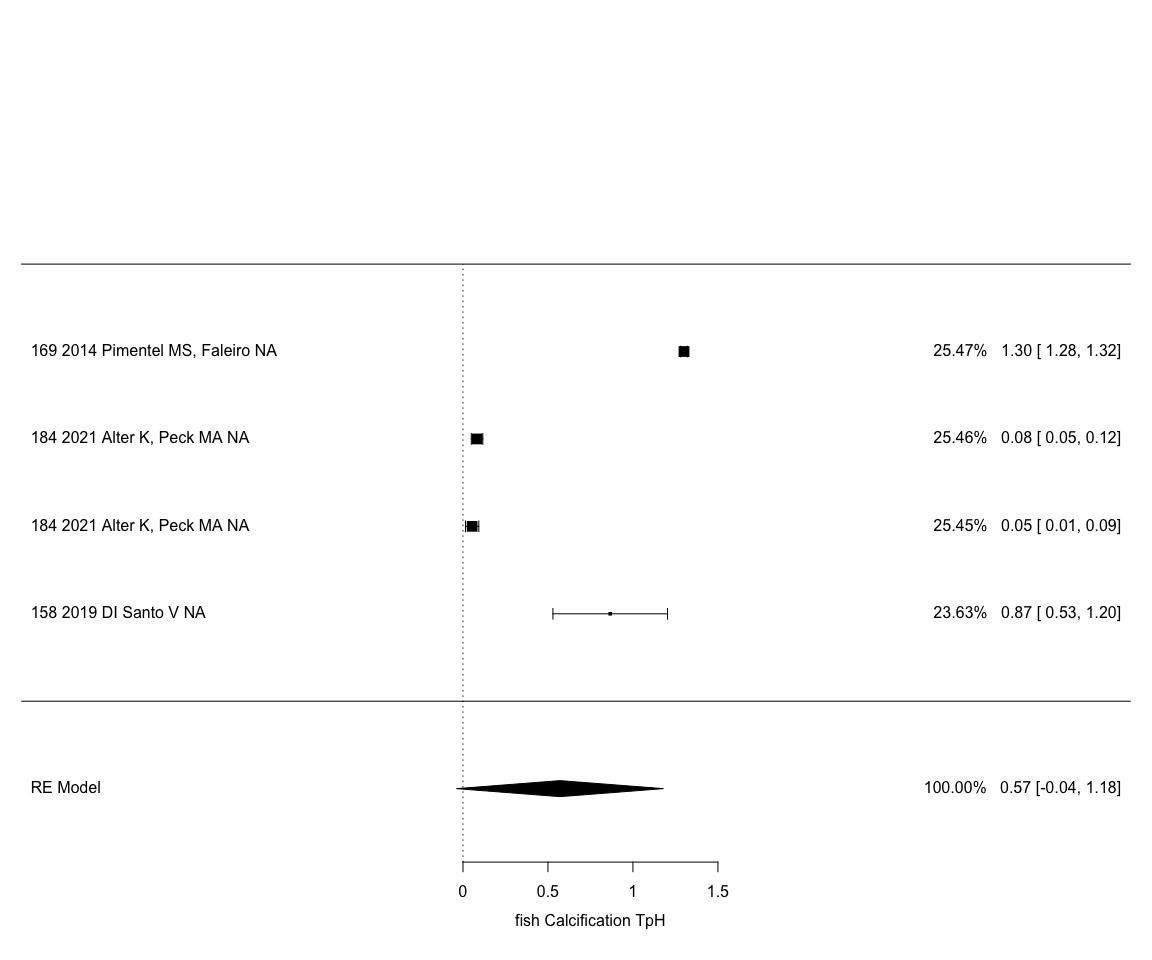


Abs_FishCalci <- MA_TpH_abs("fish","Calcification", Fish)

## Invertebrate, Biodiversity
InvertBiodiv <- MA_TpH("inverts","Biodiversity", Inverts,sensitivity)

##
## Random-Effects Model (k = 3; tau^2 estimator: REML)
##
## tau^2 (estimated amount of total heterogeneity): 0.0326 (SE = 0.0468)
## tau (square root of estimated tau^2 value): 0.1806
## I^2 (total heterogeneity / total variability): 69.75%
## H^2 (total variability / sampling variability): 3.31
##
## Test for Heterogeneity:
## Q(df = 2) = 6.6051, p-val = 0.0368
##
## Model Results:
##
## estimate se zval pval ci.lb ci.ub ​
## 0.0996 0.1249 0.7974 0.4252 -0.1452 0.3443
##
## ---
## Signif. codes: 0 '***' 0.001 '**' 0.01 '*' 0.05 '.' 0.1 ' ' 1


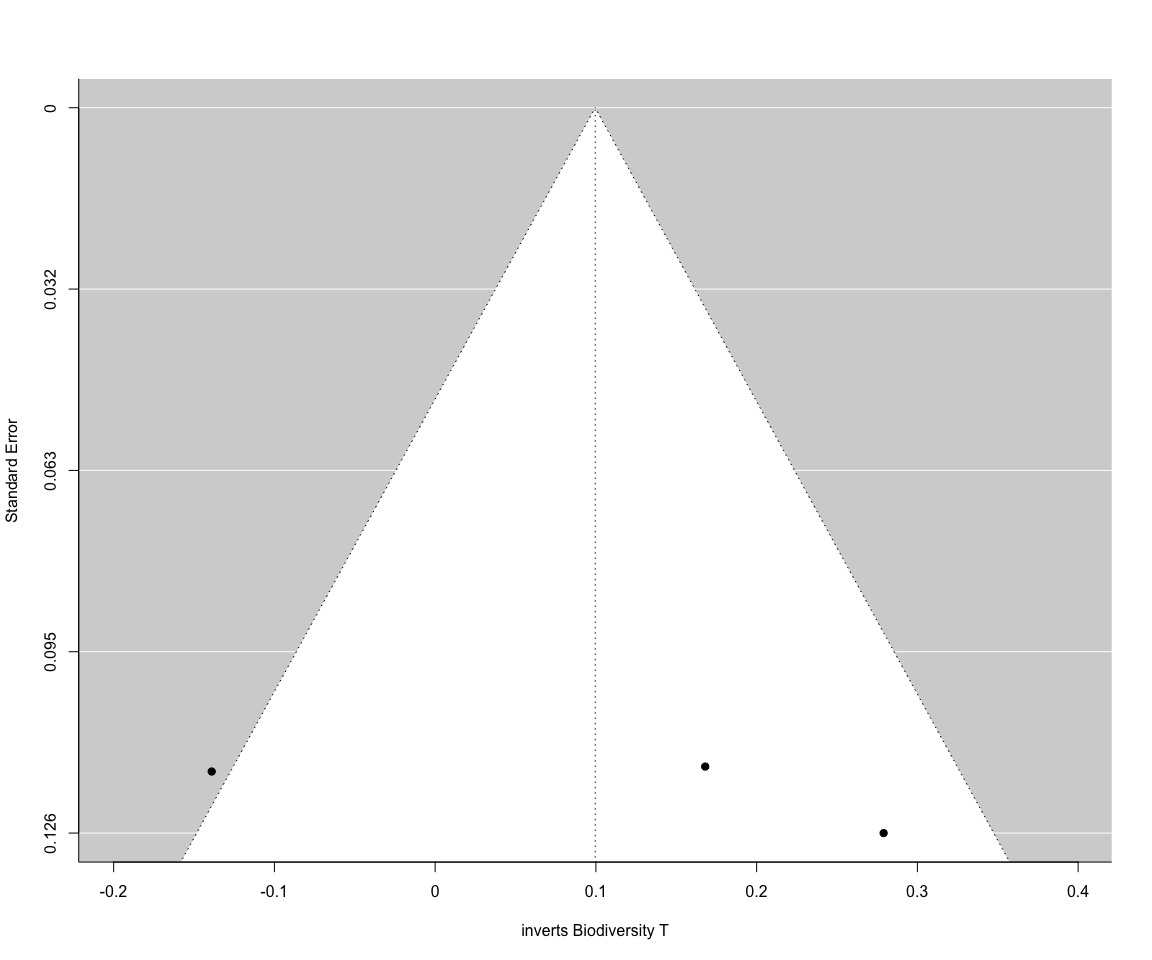

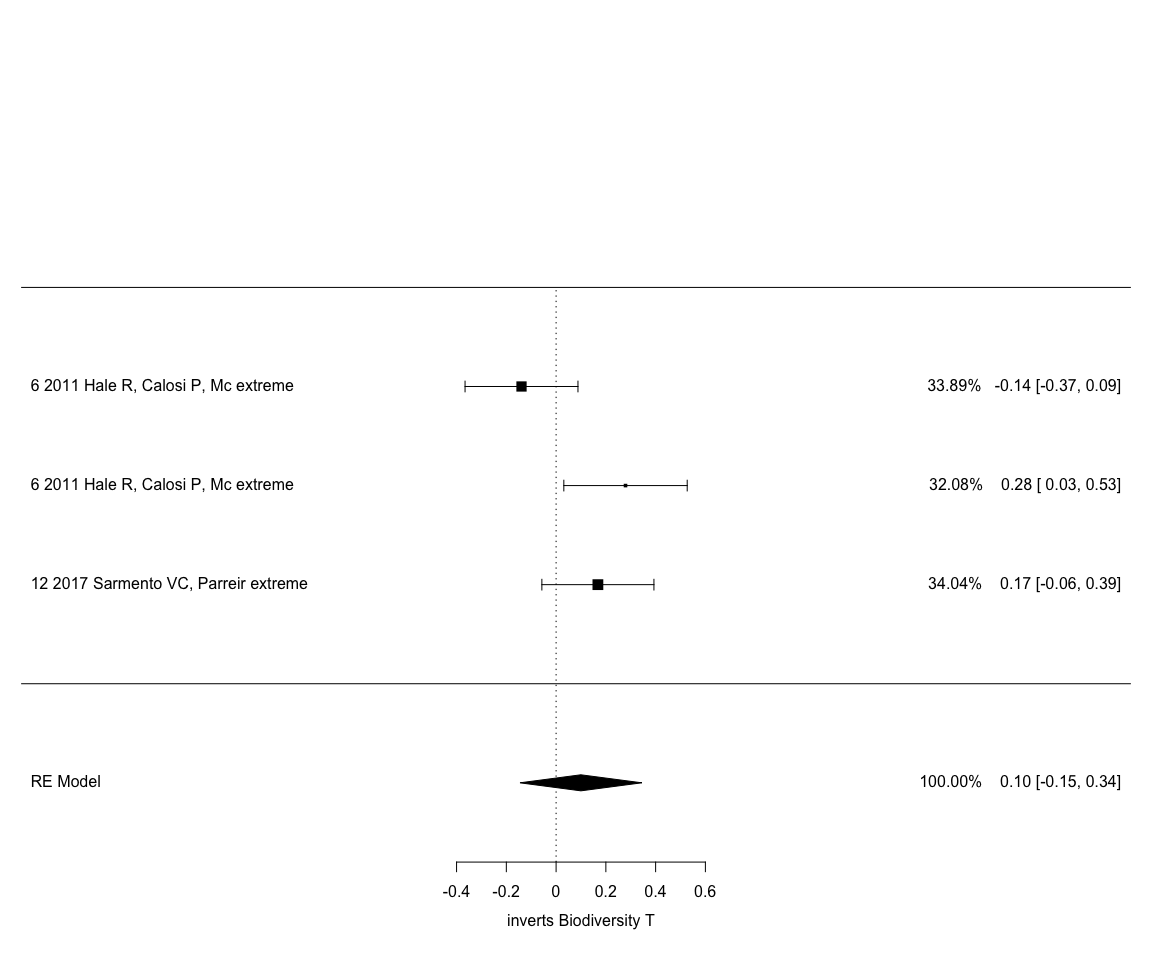


##
## Random-Effects Model (k = 9; tau^2 estimator: REML)
##
## tau^2 (estimated amount of total heterogeneity): 0.0377 (SE = 0.0314)
## tau (square root of estimated tau^2 value): 0.1943
## I^2 (total heterogeneity / total variability): 62.77%
## H^2 (total variability / sampling variability): 2.69
##
## Test for Heterogeneity:
## Q(df = 8) = 21.6547, p-val = 0.0056
##
## Model Results:
##
## estimate se zval pval ci.lb ci.ub ​
## -0.0733 0.0848 -0.8643 0.3874 -0.2395 0.0929
##
## ---
## Signif. codes: 0 '***' 0.001 '**' 0.01 '*' 0.05 '.' 0.1 ' ' 1


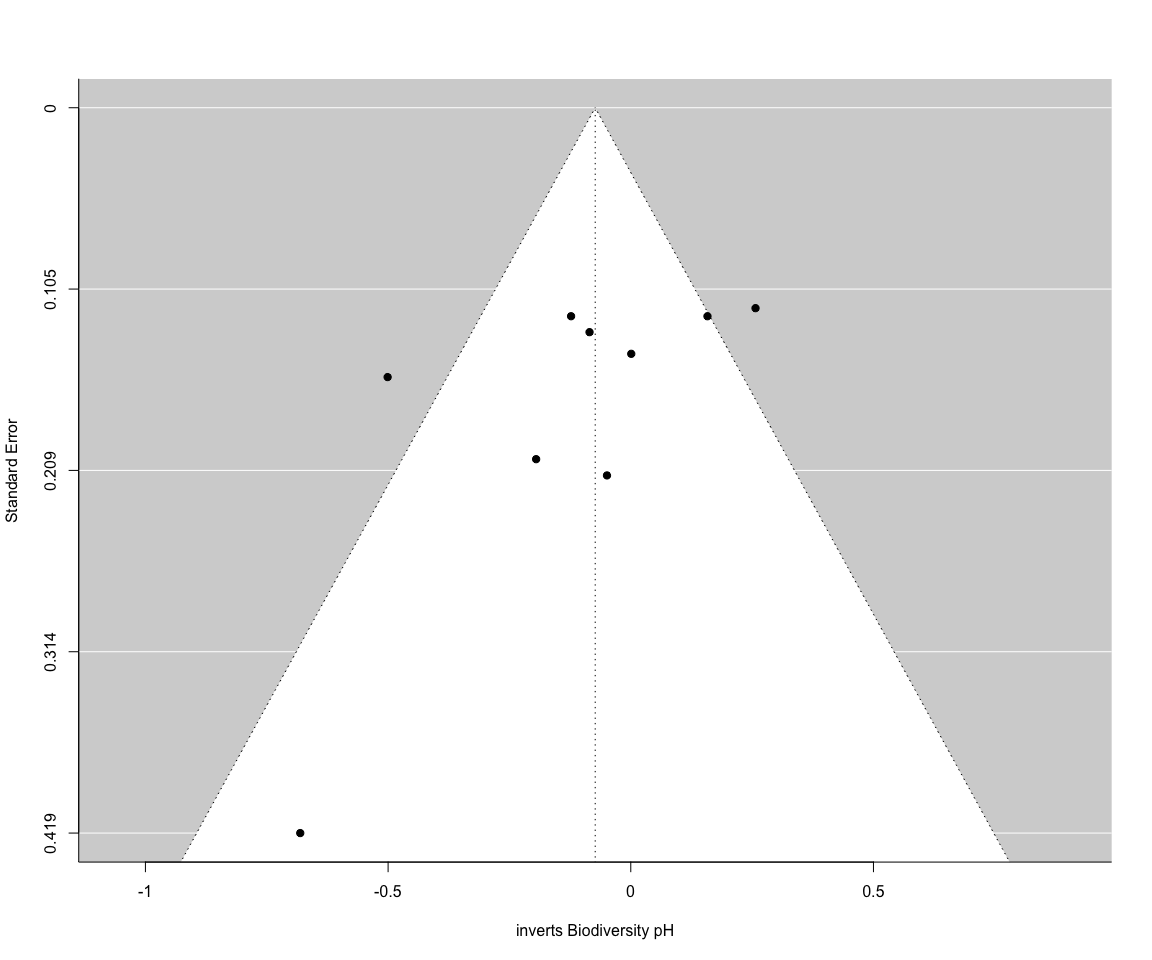

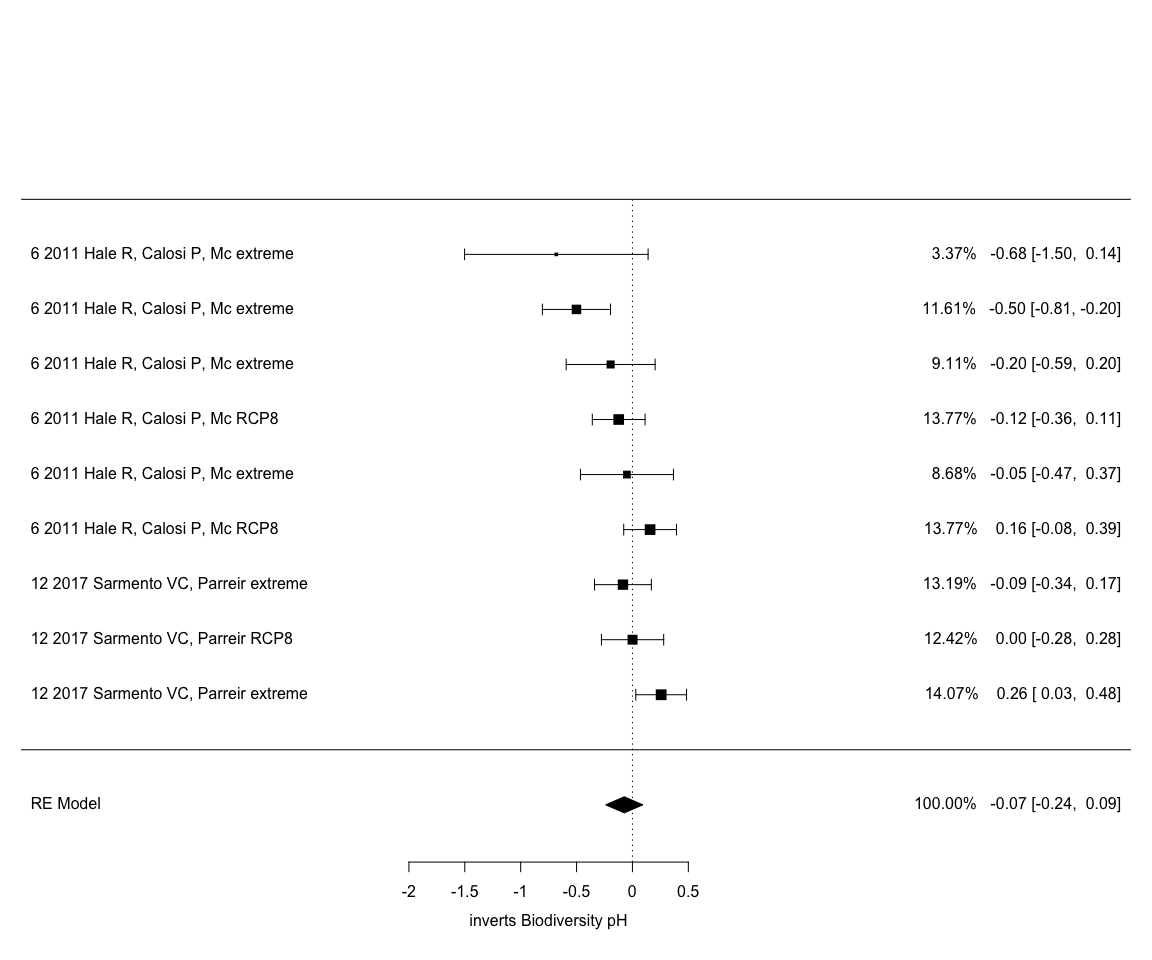


##
## Random-Effects Model (k = 9; tau^2 estimator: REML)
##
## tau^2 (estimated amount of total heterogeneity): 0.0876 (SE = 0.0541)
## tau (square root of estimated tau^2 value): 0.2961
## I^2 (total heterogeneity / total variability): 84.00%
## H^2 (total variability / sampling variability): 6.25
##
## Test for Heterogeneity:
## Q(df = 8) = 66.7796, p-val < .0001
##
## Model Results:
##
## estimate se zval pval ci.lb ci.ub ​
## -0.1220 0.1099 -1.1097 0.2671 -0.3374 0.0934
##
## ---
## Signif. codes: 0 '***' 0.001 '**' 0.01 '*' 0.05 '.' 0.1 ' ' 1


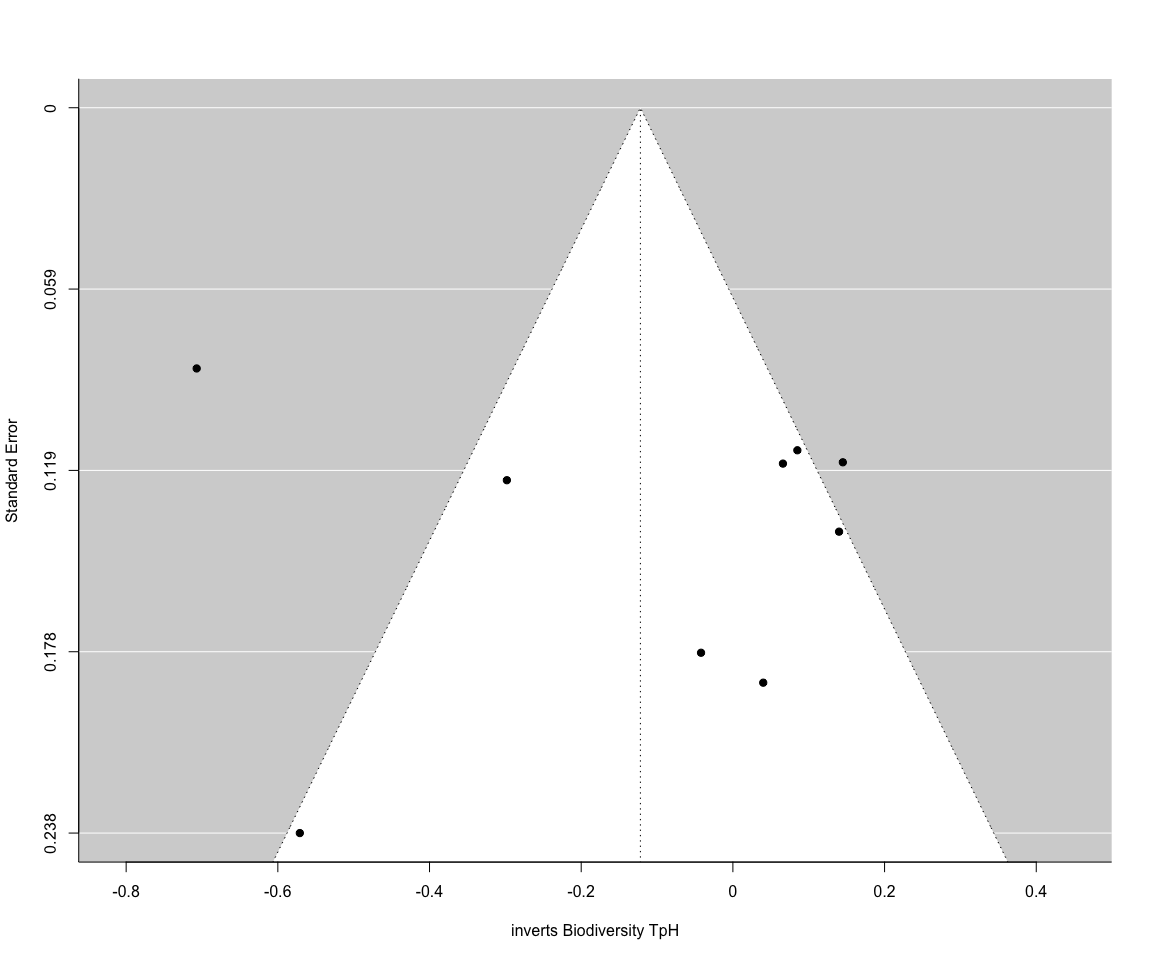

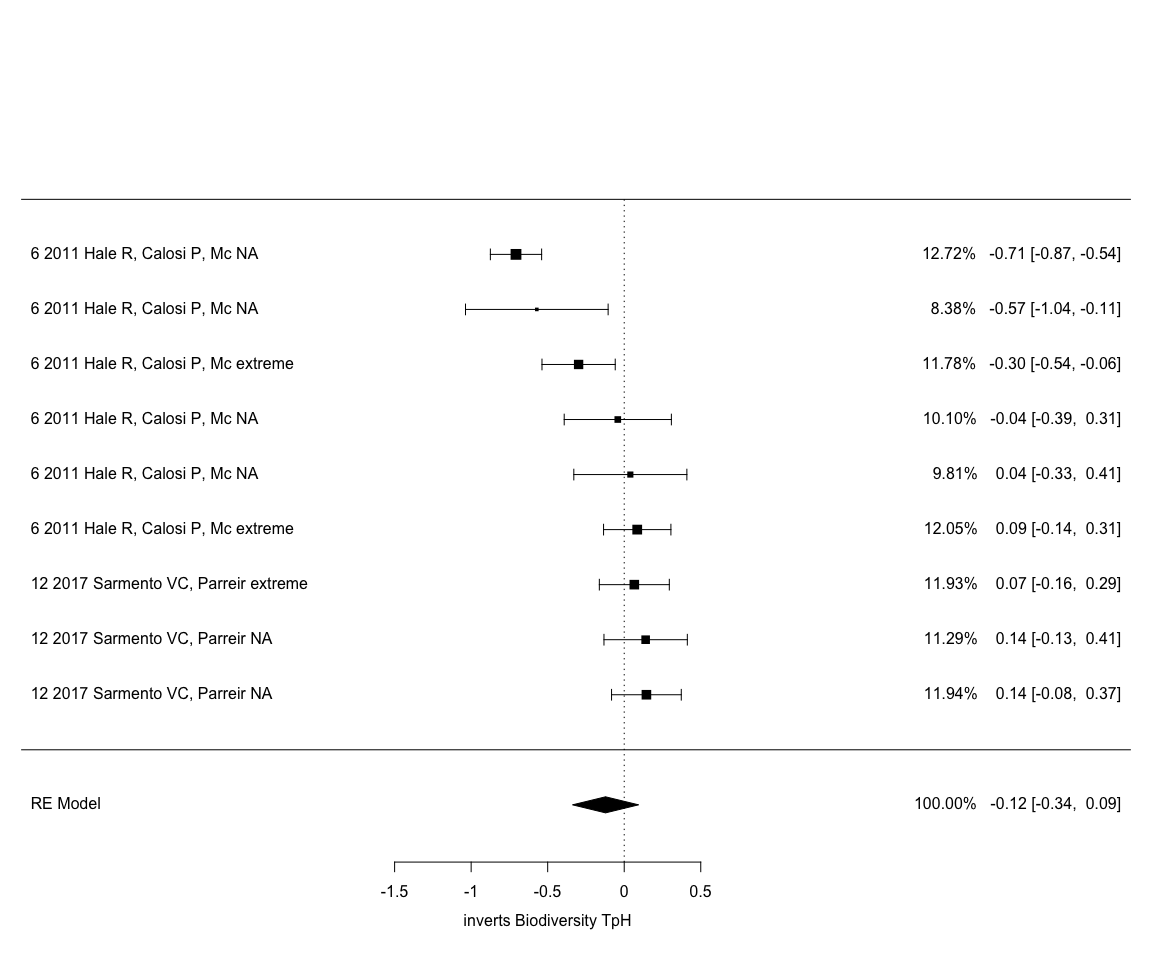


Abs_InvertBiodiv <- MA_TpH_abs("inverts","Biodiversity", Inverts)

## Invertebrate, Survival
InvertSurvi <- MA_TpH("inverts","Survival", Inverts,sensitivity)

##
## Random-Effects Model (k = 68; tau^2 estimator: REML)
##
## tau^2 (estimated amount of total heterogeneity): 0.1622 (SE = 0.0362)
## tau (square root of estimated tau^2 value): 0.4027
## I^2 (total heterogeneity / total variability): 99.21%
## H^2 (total variability / sampling variability): 127.24
##
## Test for Heterogeneity:
## Q(df = 67) = 790.5876, p-val < .0001
##
## Model Results:
##
## estimate se zval pval ci.lb ci.ub ​
## -0.1161 0.0582 -1.9958 0.0460 -0.2301 -0.0021 *
##
## ---
## Signif. codes: 0 '***' 0.001 '**' 0.01 '*' 0.05 '.' 0.1 ' ' 1


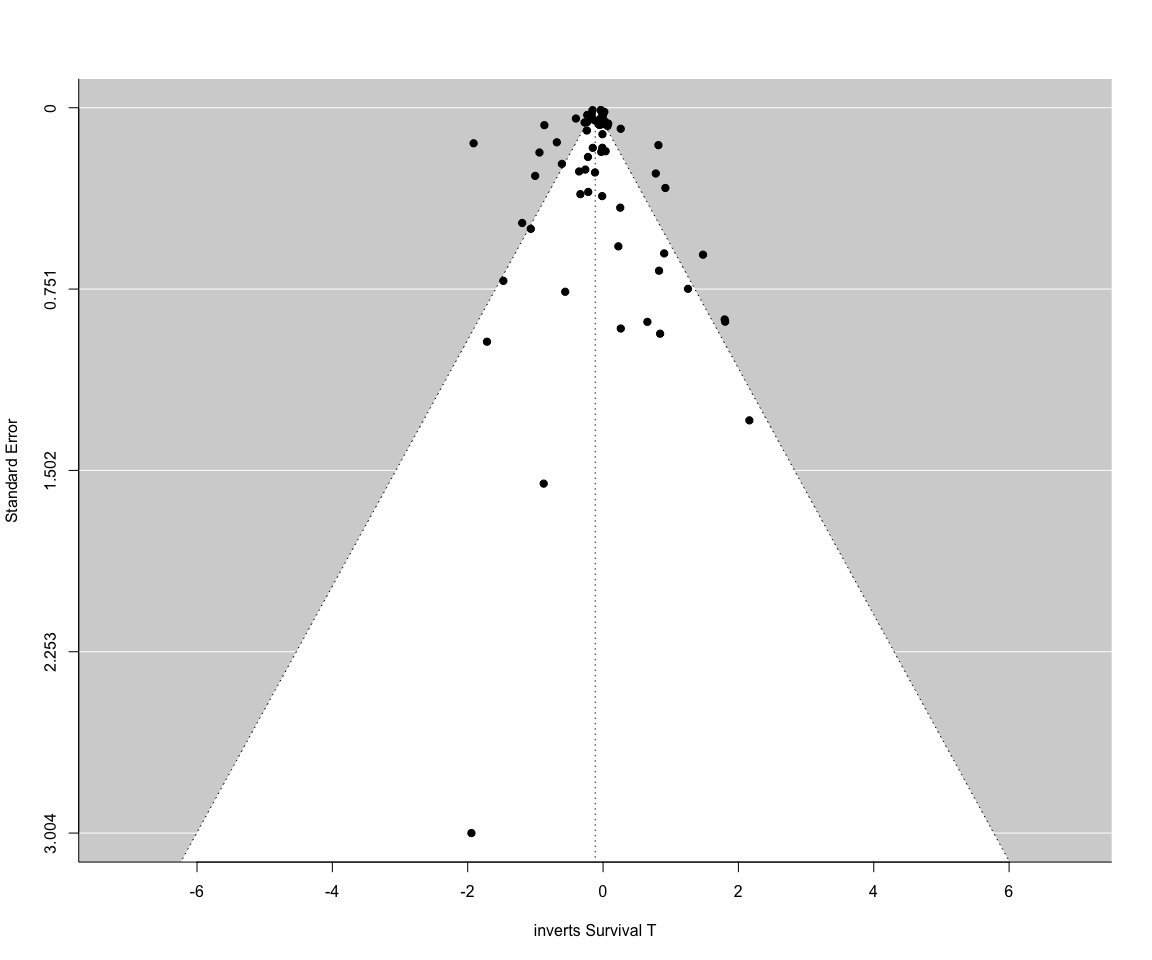

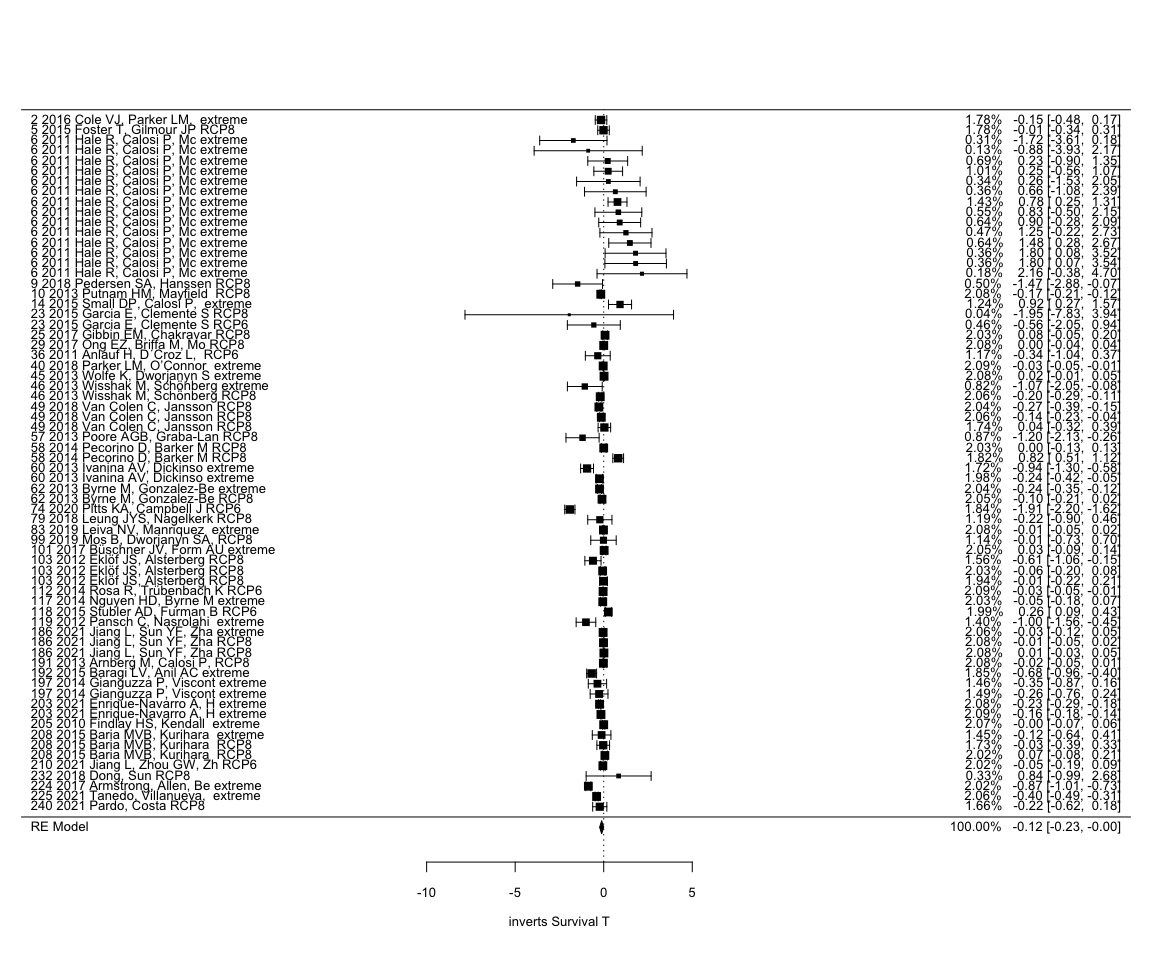


##
## Random-Effects Model (k = 115; tau^2 estimator: REML)
##
## tau^2 (estimated amount of total heterogeneity): 0.0985 (SE = 0.0178)
## tau (square root of estimated tau^2 value): 0.3139
## I^2 (total heterogeneity / total variability): 99.21%
## H^2 (total variability / sampling variability): 126.29
##
## Test for Heterogeneity:
## Q(df = 114) = 13754.3949, p-val < .0001
##
## Model Results:
##
## estimate se zval pval ci.lb ci.ub ​
## -0.1756 0.0362 -4.8489 <.0001 -0.2466 -0.1046 ***
##
## ---
## Signif. codes: 0 '***' 0.001 '**' 0.01 '*' 0.05 '.' 0.1 ' ' 1


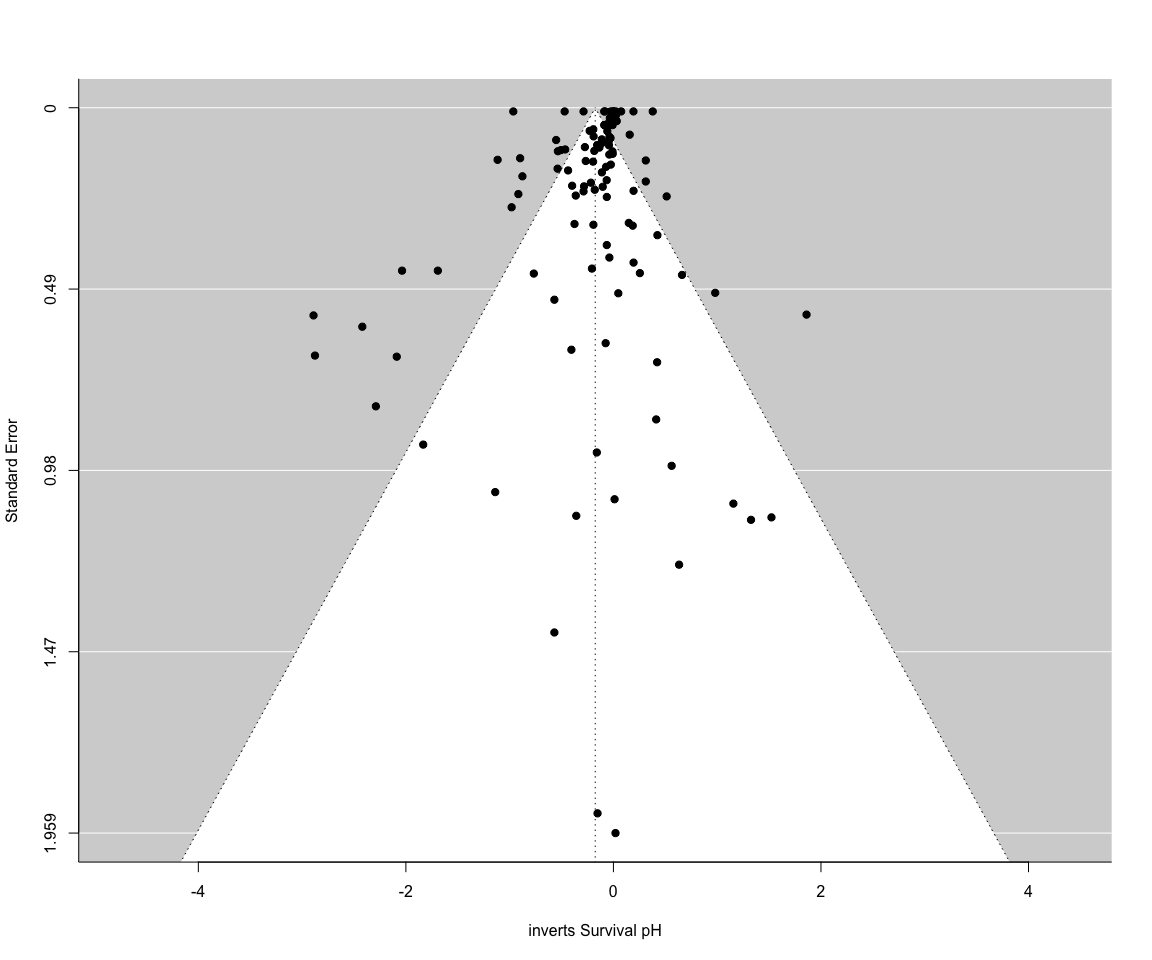

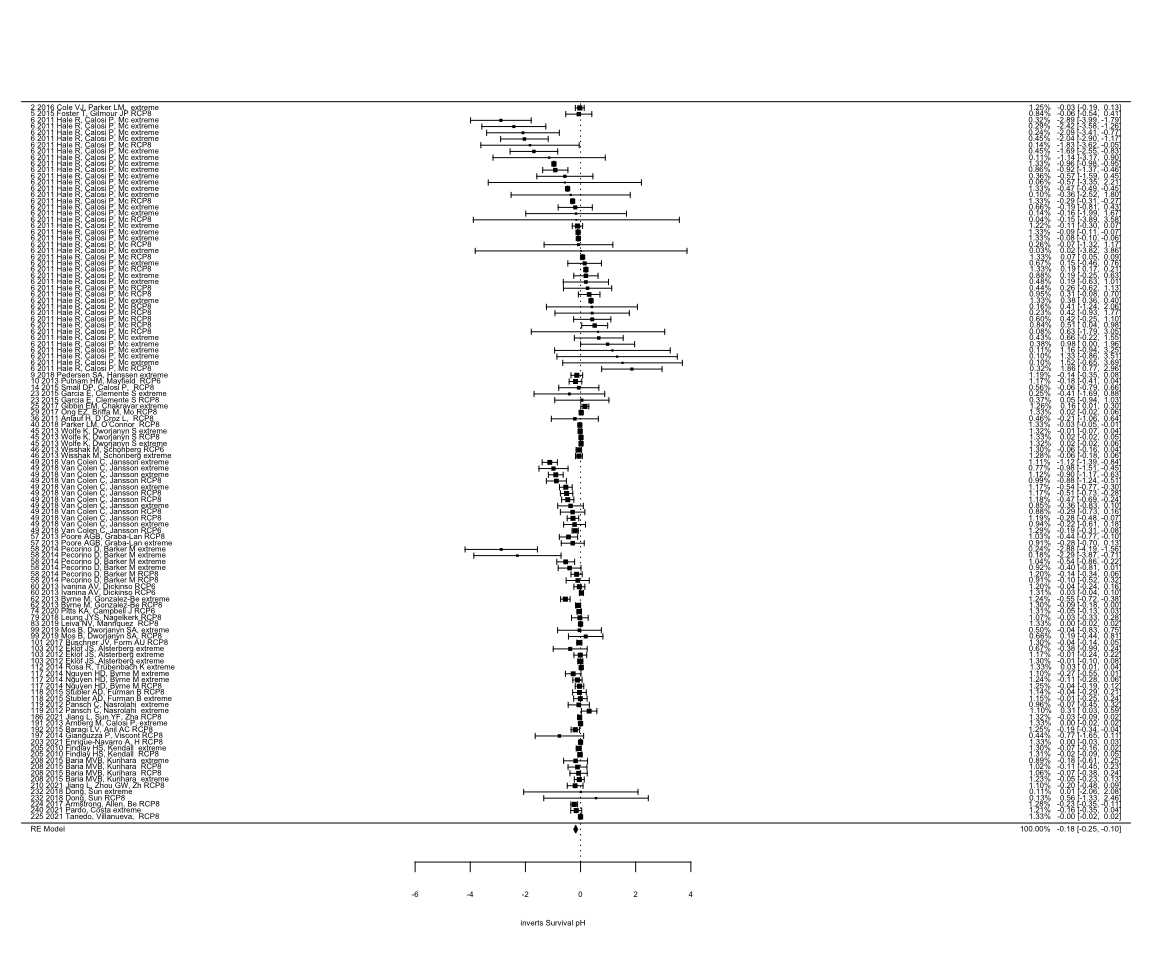


##
## Random-Effects Model (k = 125; tau^2 estimator: REML)
##
## tau^2 (estimated amount of total heterogeneity): 0.3072 (SE = 0.0490)
## tau (square root of estimated tau^2 value): 0.5542
## I^2 (total heterogeneity / total variability): 99.42%
## H^2 (total variability / sampling variability): 173.32
##
## Test for Heterogeneity:
## Q(df = 124) = 3688.6842, p-val < .0001
##
## Model Results:
##
## estimate se zval pval ci.lb ci.ub ​
## -0.2621 0.0575 -4.5612 <.0001 -0.3748 -0.1495 ***
##
## ---
## Signif. codes: 0 '***' 0.001 '**' 0.01 '*' 0.05 '.' 0.1 ' ' 1


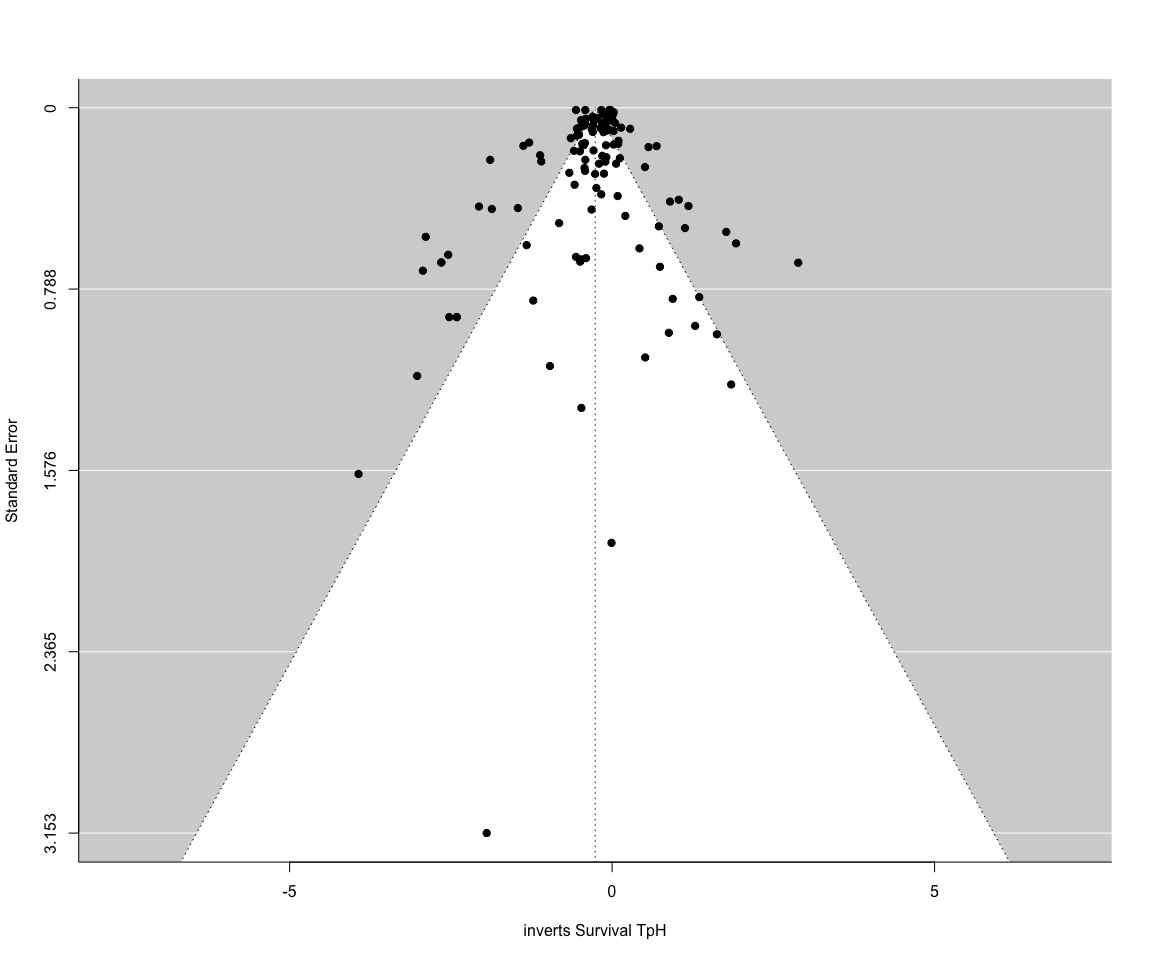

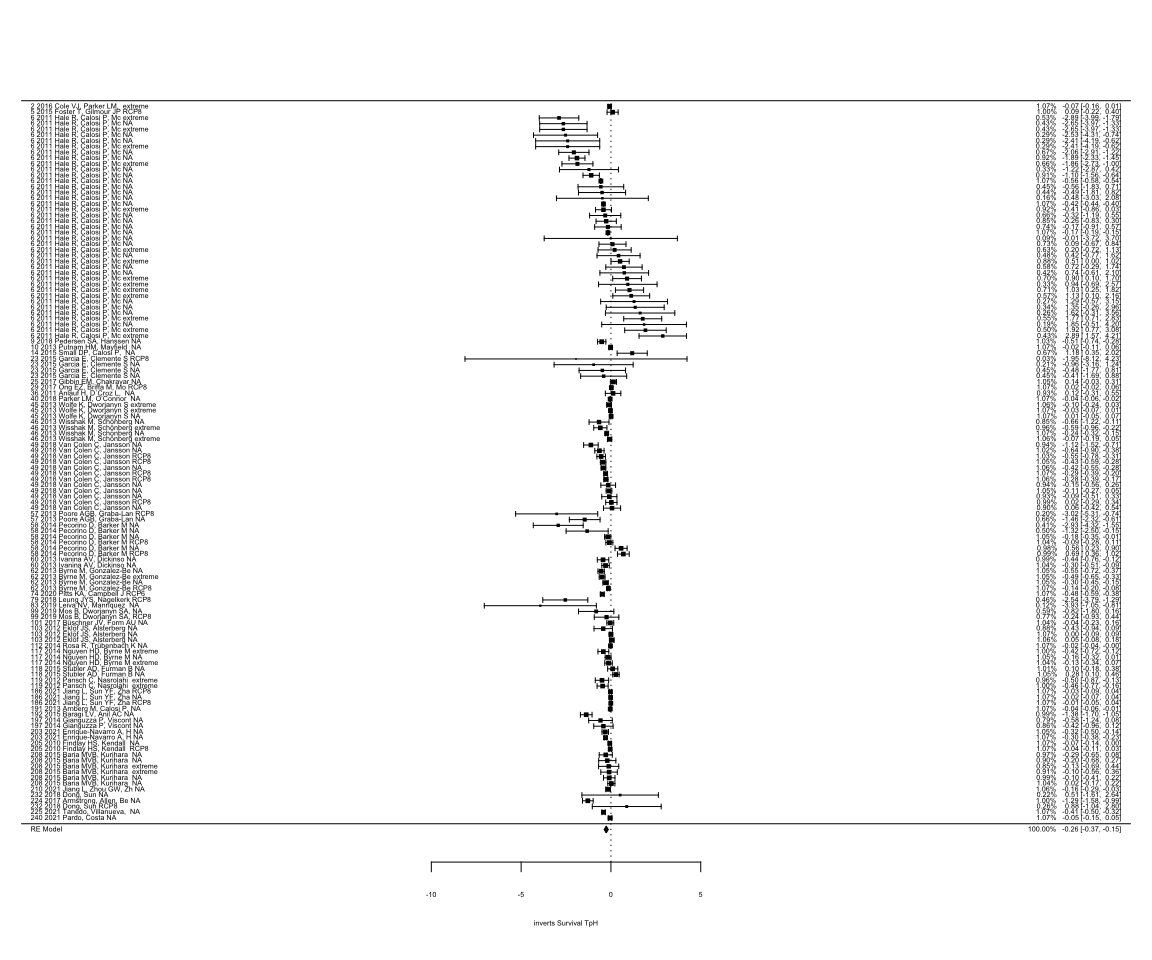


Abs_InvertSurvi <- MA_TpH_abs("inverts","Survival", Inverts)

## Fish, Survival
FishSurvi <- MA_TpH("fish","Survival", Fish,sensitivity)

##
## Random-Effects Model (k = 31; tau^2 estimator: REML)
##
## tau^2 (estimated amount of total heterogeneity): 0.2471 (SE = 0.0716)
## tau (square root of estimated tau^2 value): 0.4971
## I^2 (total heterogeneity / total variability): 99.81%
## H^2 (total variability / sampling variability): 515.29
##
## Test for Heterogeneity:
## Q(df = 30) = 1452.4270, p-val < .0001
##
## Model Results:
##
## estimate se zval pval ci.lb ci.ub ​
## -0.2484 0.0953 -2.6054 0.0092 -0.4353 -0.0615 **
##
## ---
## Signif. codes: 0 '***' 0.001 '**' 0.01 '*' 0.05 '.' 0.1 ' ' 1


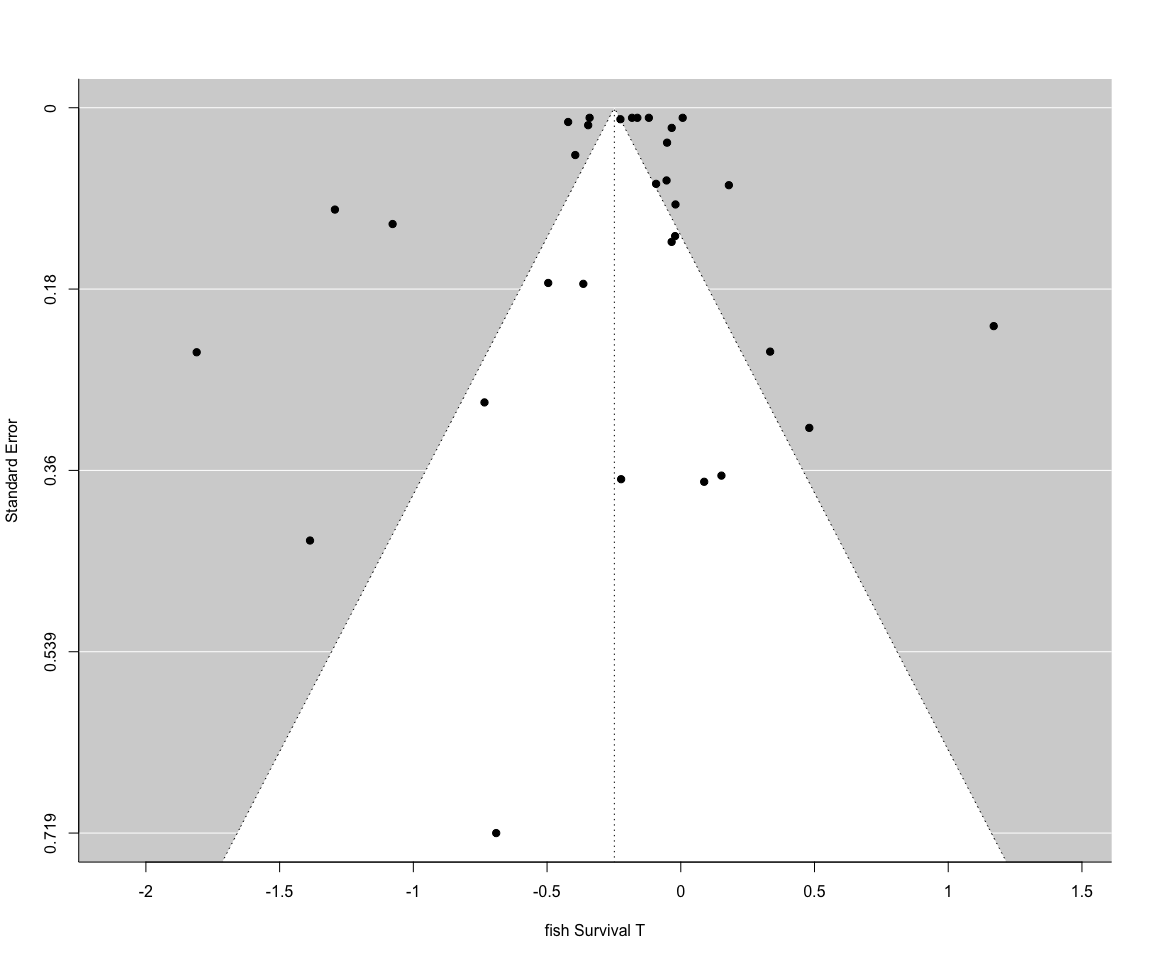

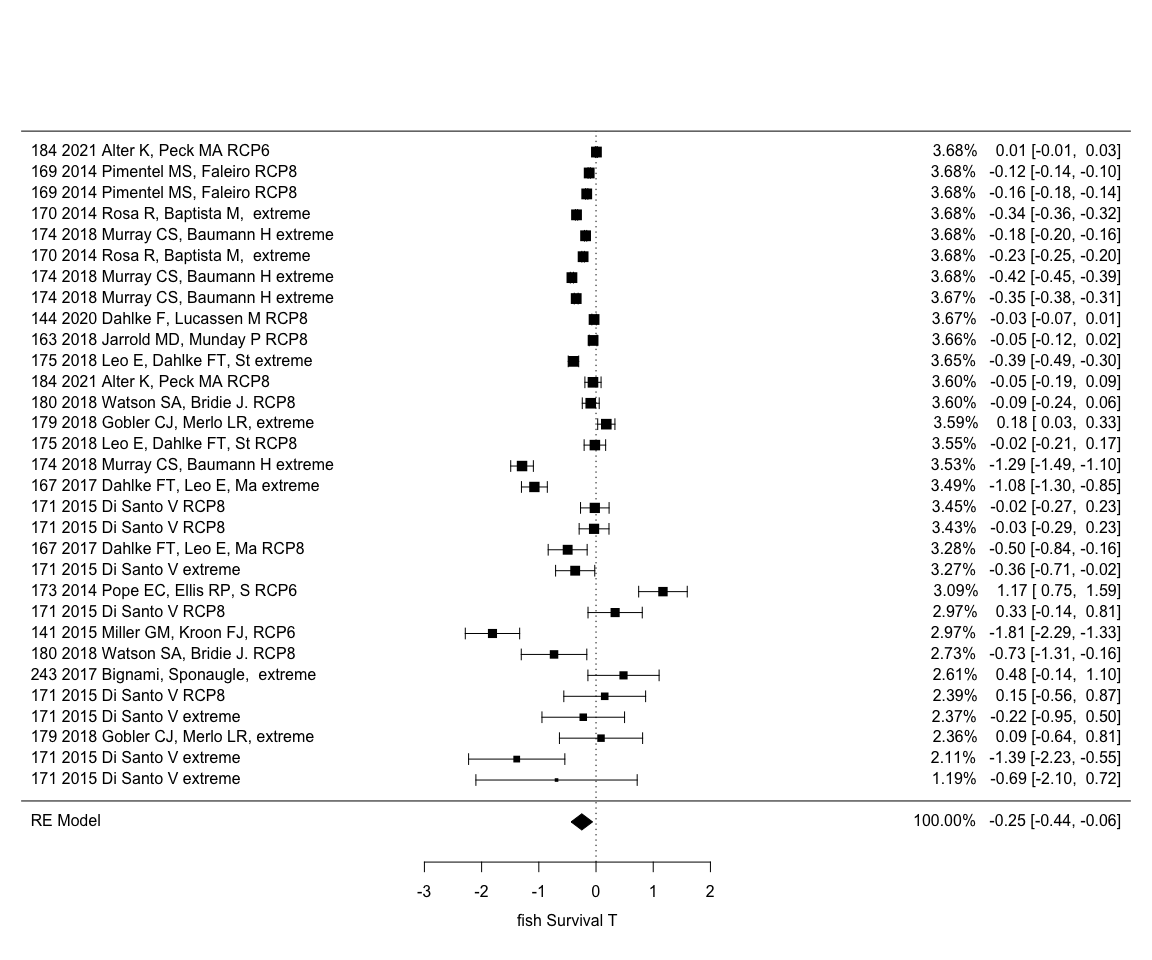


##
## Random-Effects Model (k = 25; tau^2 estimator: REML)
##
## tau^2 (estimated amount of total heterogeneity): 0.0291 (SE = 0.0108)
## tau (square root of estimated tau^2 value): 0.1705
## I^2 (total heterogeneity / total variability): 98.62%
## H^2 (total variability / sampling variability): 72.60
##
## Test for Heterogeneity:
## Q(df = 24) = 1099.2848, p-val < .0001
##
## Model Results:
##
## estimate se zval pval ci.lb ci.ub ​
## -0.0749 0.0404 -1.8551 0.0636 -0.1540 0.0042 .
##
## ---
## Signif. codes: 0 '***' 0.001 '**' 0.01 '*' 0.05 '.' 0.1 ' ' 1


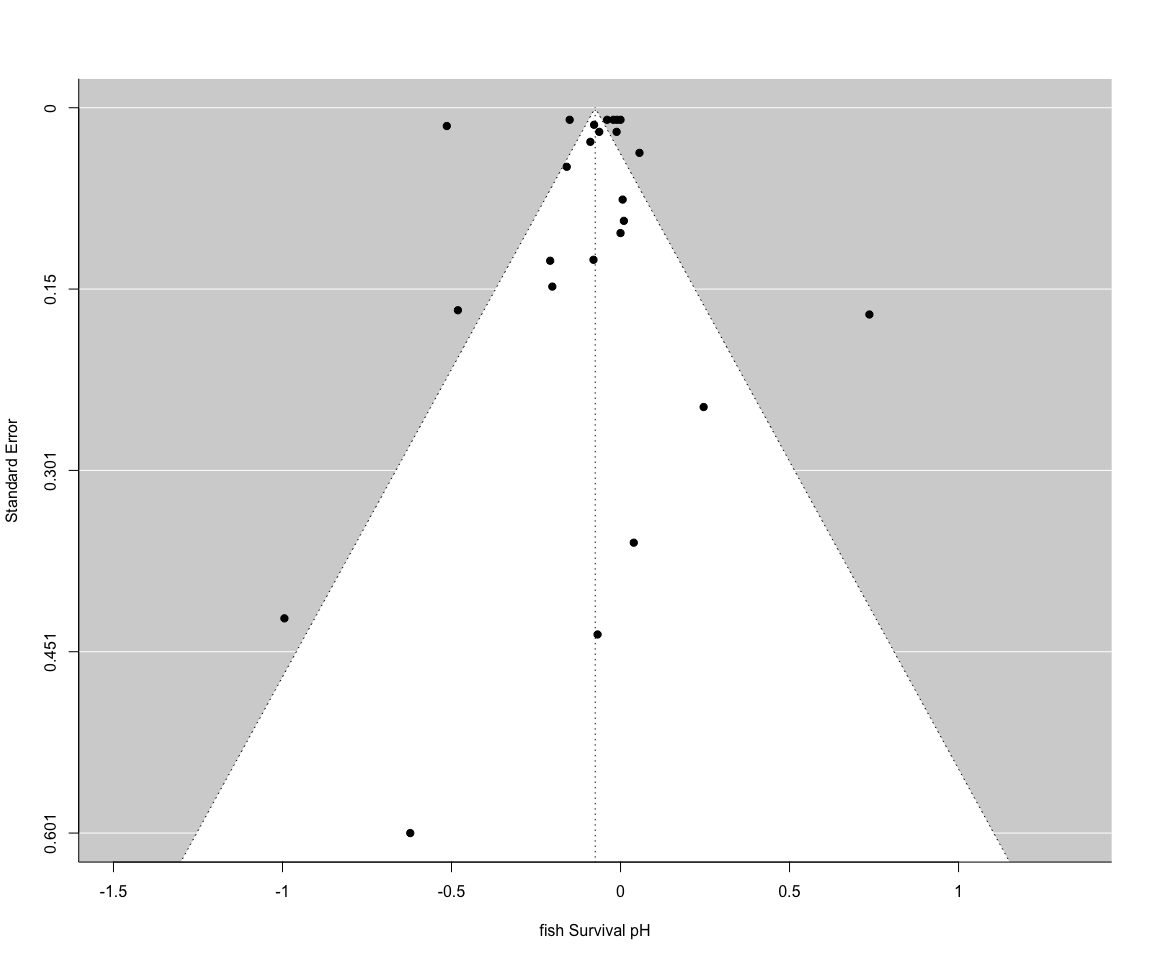

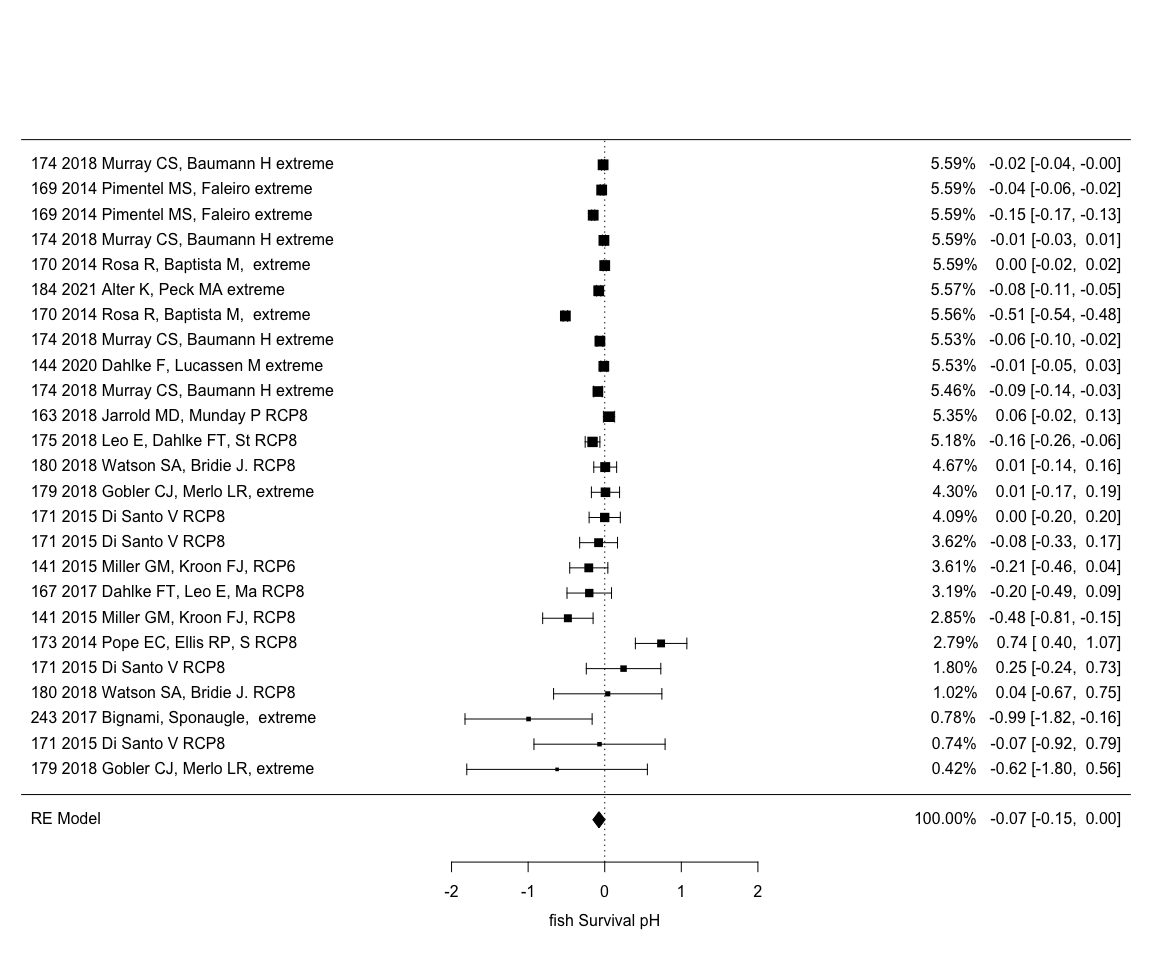


##
## Random-Effects Model (k = 35; tau^2 estimator: REML)
##
## tau^2 (estimated amount of total heterogeneity): 0.3750 (SE = 0.0993)
## tau (square root of estimated tau^2 value): 0.6123
## I^2 (total heterogeneity / total variability): 99.87%
## H^2 (total variability / sampling variability): 752.52
##
## Test for Heterogeneity:
## Q(df = 34) = 3490.6626, p-val < .0001
##
## Model Results:
##
## estimate se zval pval ci.lb ci.ub ​
## -0.4012 0.1087 -3.6903 0.0002 -0.6143 -0.1881 ***
##
## ---
## Signif. codes: 0 '***' 0.001 '**' 0.01 '*' 0.05 '.' 0.1 ' ' 1


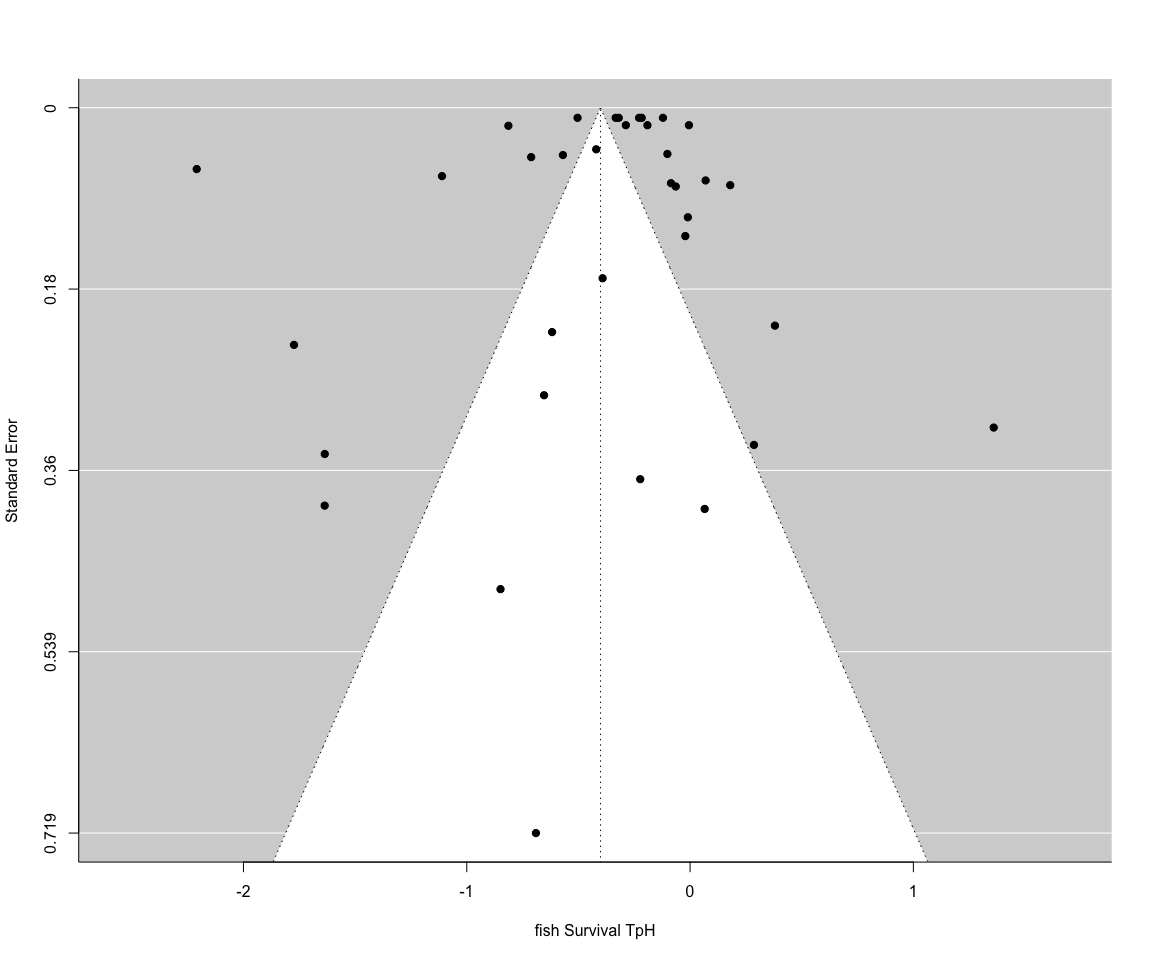

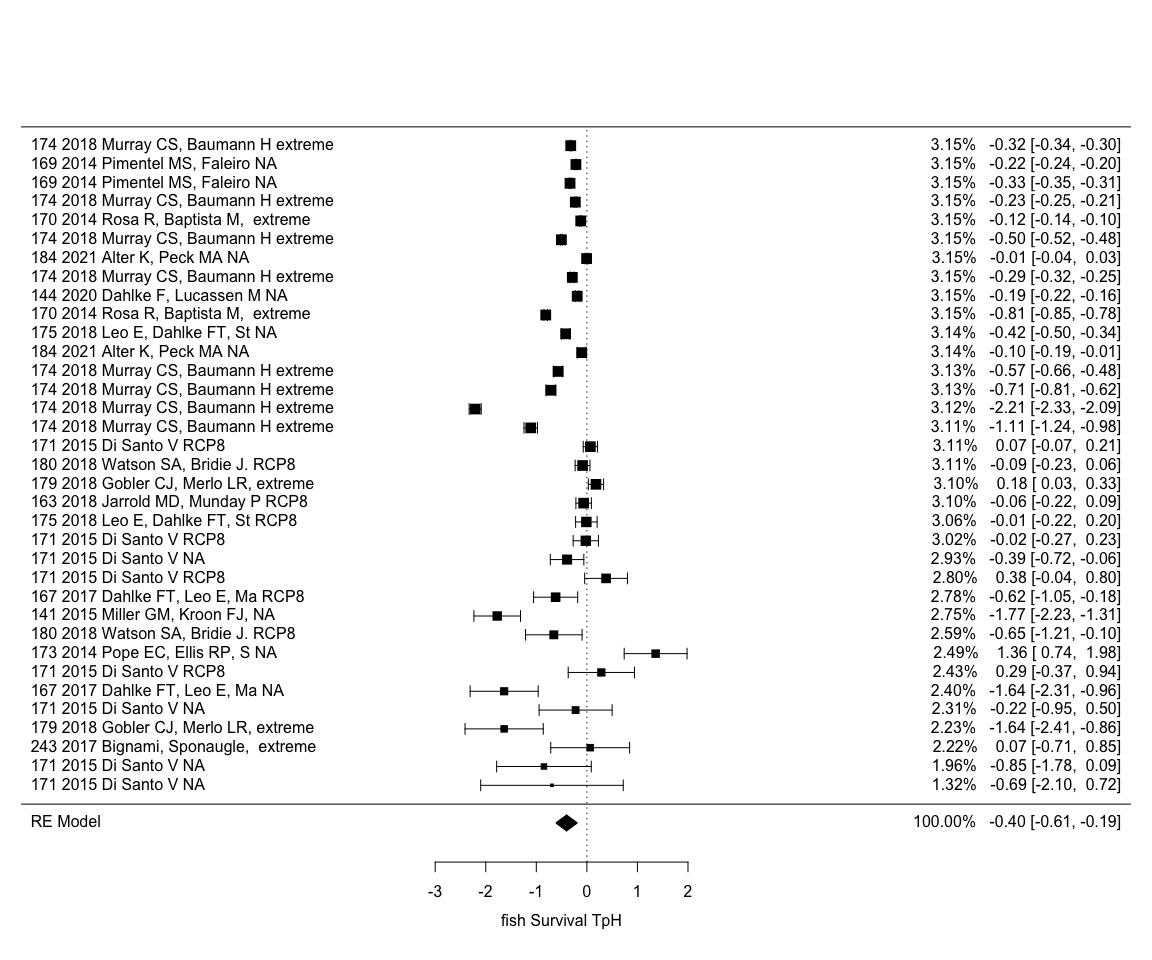


Abs_FishSurvi <- MA_TpH_abs("fish","Survival", Fish)

## Invertebrate, Reproduction
InvertRepro <- MA_TpH("inverts","Reproduction", Inverts,sensitivity)

##
## Random-Effects Model (k = 59; tau^2 estimator: REML)
##
## tau^2 (estimated amount of total heterogeneity): 0.0797 (SE = 0.0168)
## tau (square root of estimated tau^2 value): 0.2823
## I^2 (total heterogeneity / total variability): 99.00%
## H^2 (total variability / sampling variability): 99.94
##
## Test for Heterogeneity:
## Q(df = 58) = 665.1083, p-val < .0001
##
## Model Results:
##
## estimate se zval pval ci.lb ci.ub ​
## -0.0692 0.0400 -1.7292 0.0838 -0.1477 0.0092 .
##
## ---
## Signif. codes: 0 '***' 0.001 '**' 0.01 '*' 0.05 '.' 0.1 ' ' 1


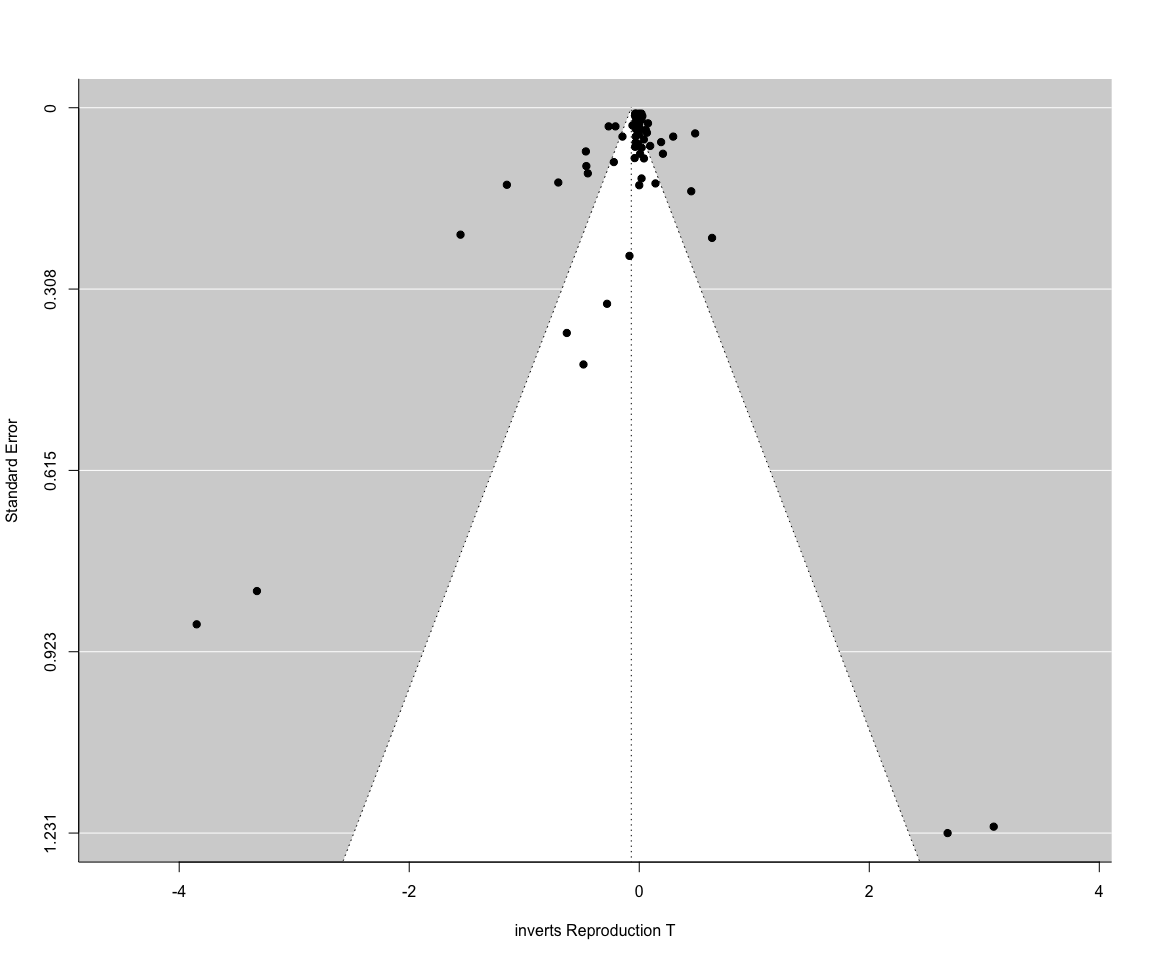

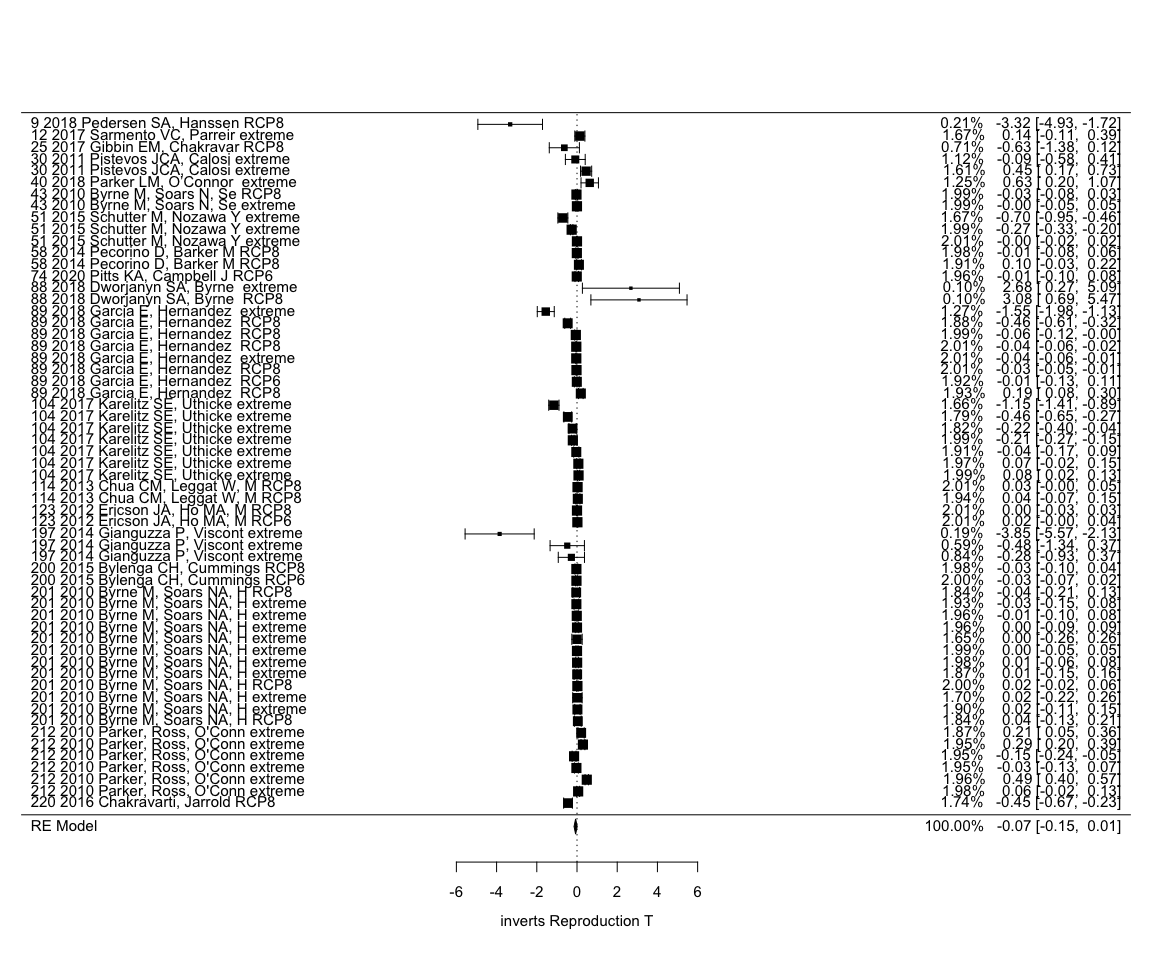


##
## Random-Effects Model (k = 62; tau^2 estimator: REML)
##
## tau^2 (estimated amount of total heterogeneity): 0.1544 (SE = 0.0301)
## tau (square root of estimated tau^2 value): 0.3929
## I^2 (total heterogeneity / total variability): 99.51%
## H^2 (total variability / sampling variability): 203.91
##
## Test for Heterogeneity:
## Q(df = 61) = 509.0600, p-val < .0001
##
## Model Results:
##
## estimate se zval pval ci.lb ci.ub ​
## -0.1337 0.0522 -2.5622 0.0104 -0.2360 -0.0314 *
##
## ---
## Signif. codes: 0 '***' 0.001 '**' 0.01 '*' 0.05 '.' 0.1 ' ' 1


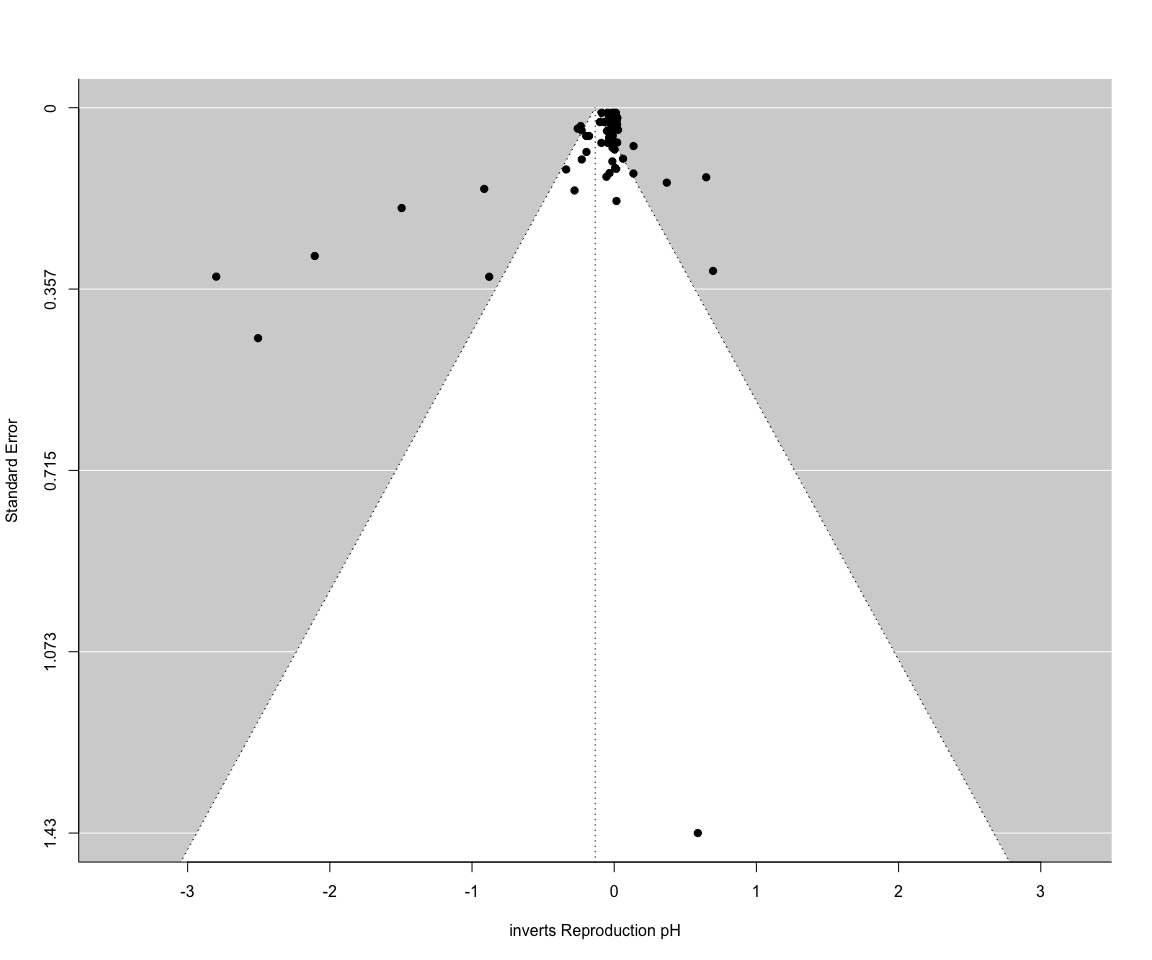


## Warning in sqrt(varSr): NaNs produced


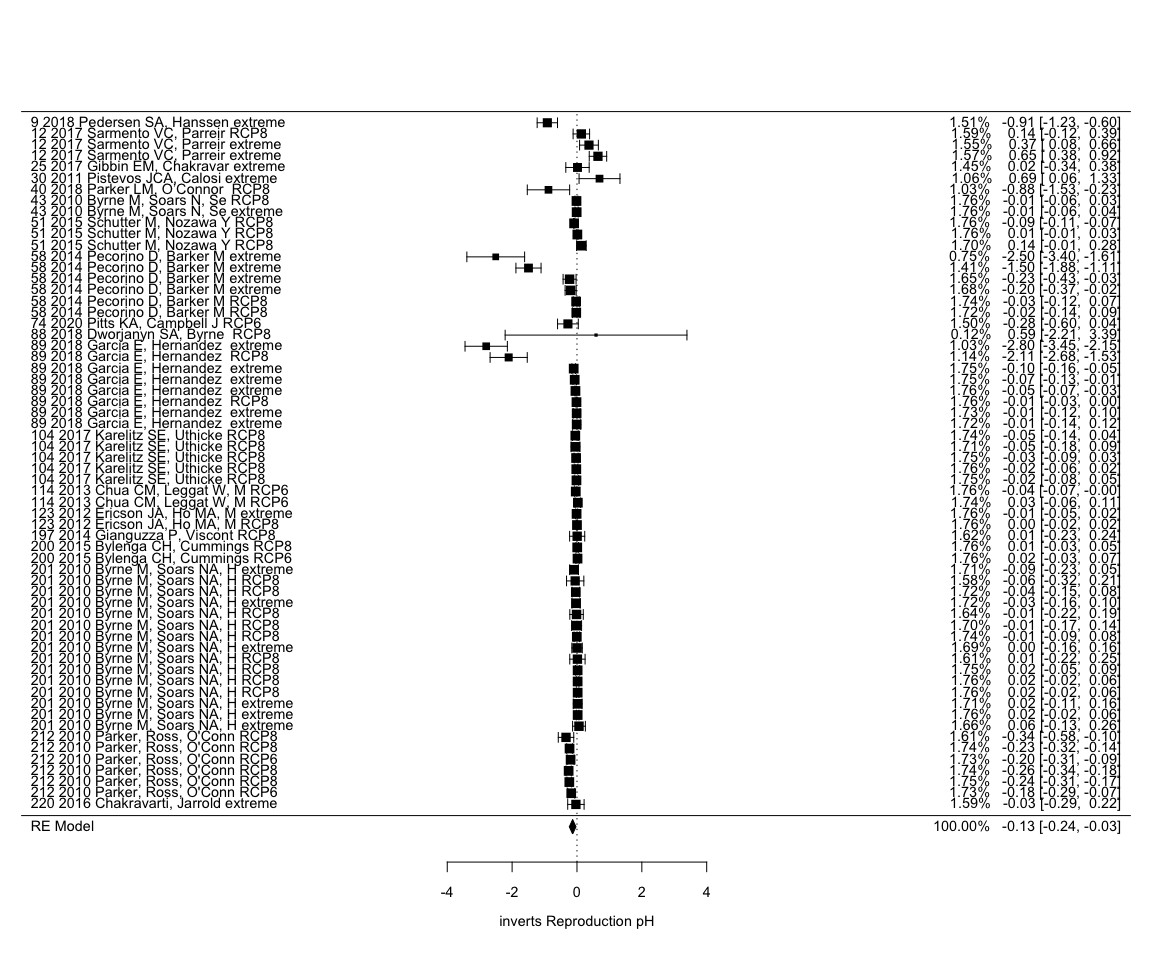


##
## Random-Effects Model (k = 111; tau^2 estimator: REML)
##
## tau^2 (estimated amount of total heterogeneity): 0.1293 (SE = 0.0193)
## tau (square root of estimated tau^2 value): 0.3596
## I^2 (total heterogeneity / total variability): 99.34%
## H^2 (total variability / sampling variability): 152.14
##
## Test for Heterogeneity:
## Q(df = 110) = 1075.7529, p-val < .0001
##
## Model Results:
##
## estimate se zval pval ci.lb ci.ub ​
## -0.1543 0.0365 -4.2246 <.0001 -0.2259 -0.0827 ***
##
## ---
## Signif. codes: 0 '***' 0.001 '**' 0.01 '*' 0.05 '.' 0.1 ' ' 1


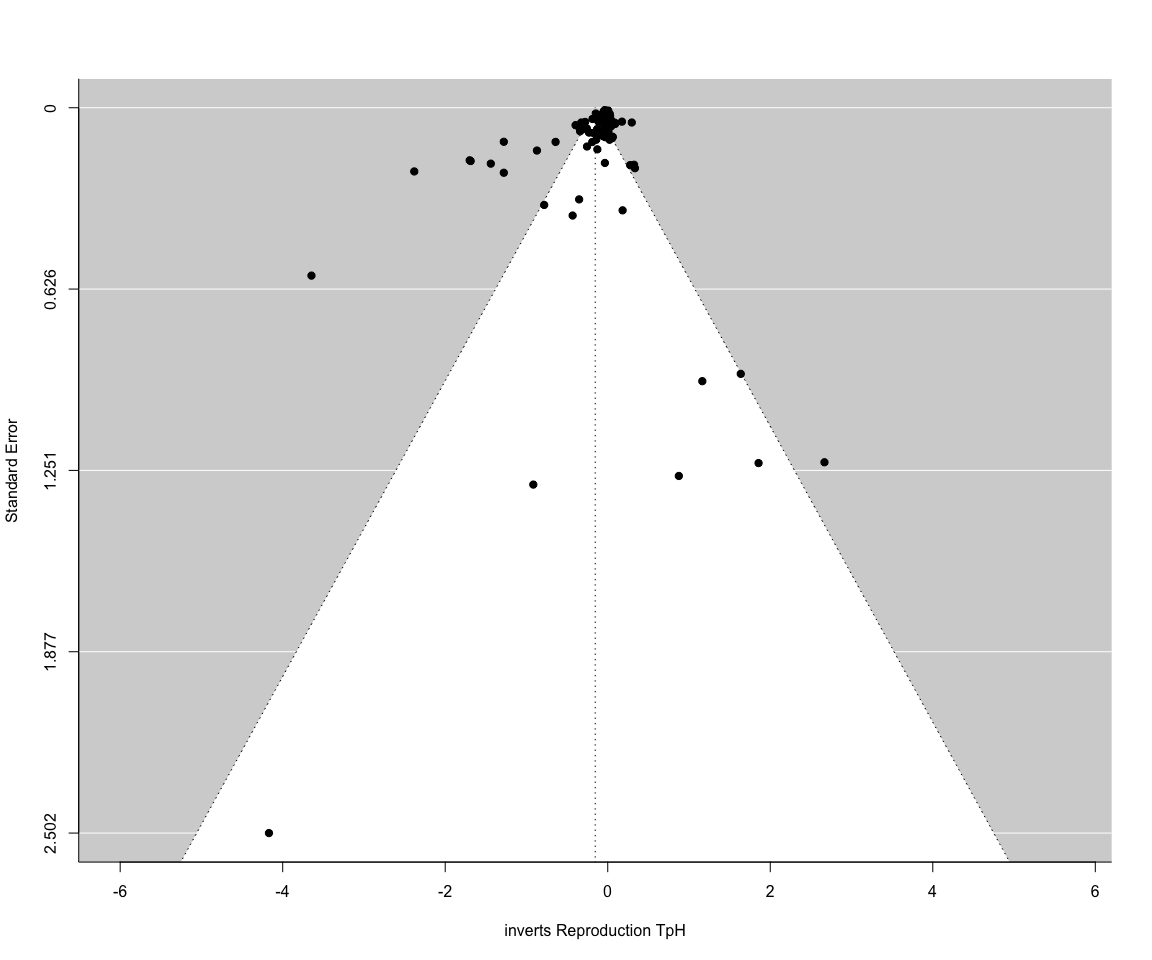

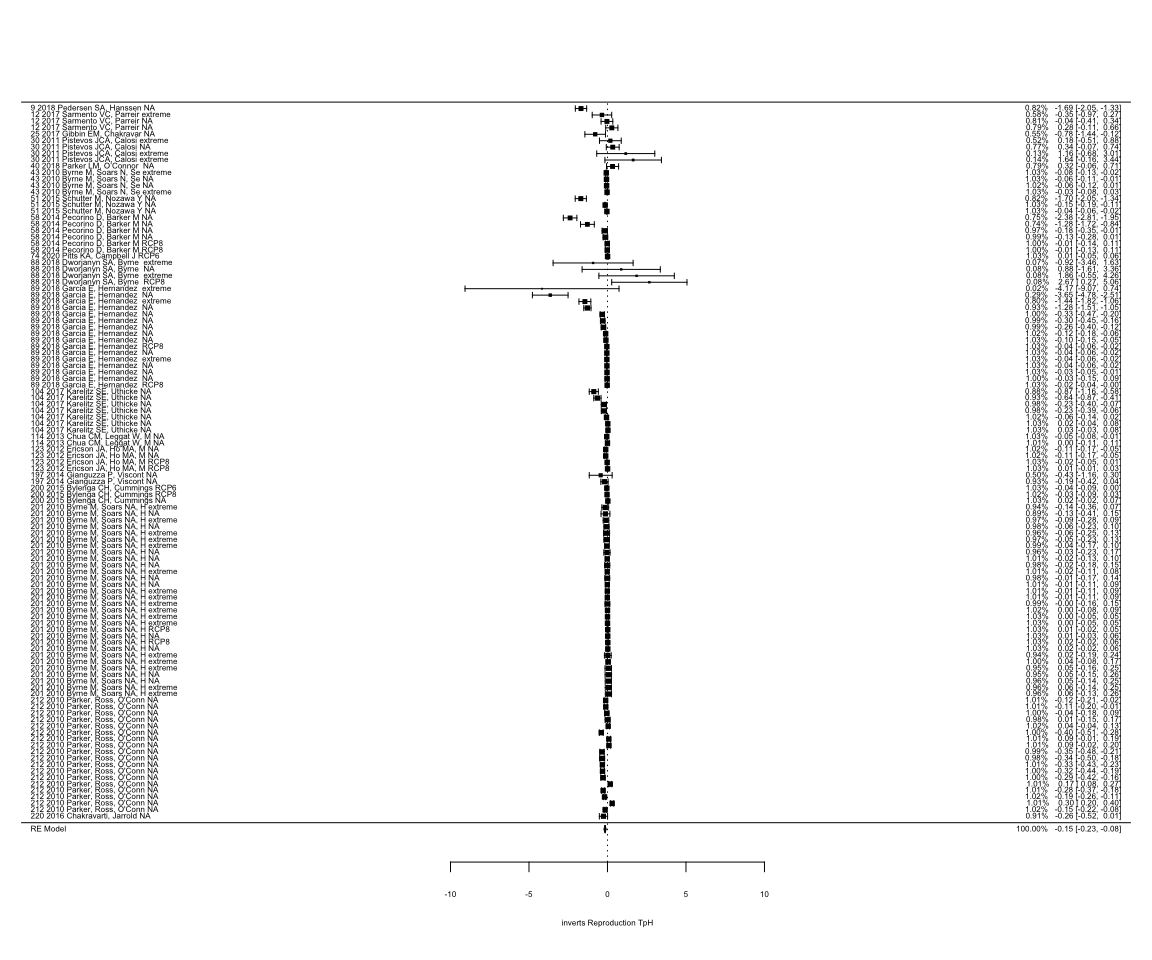


Abs_InvertRepro <- MA_TpH_abs("inverts","Reproduction", Inverts)

## Fish, Reproduction
FishRepro <- MA_TpH("fish","Reproduction", Fish,sensitivity)

##
## Random-Effects Model (k = 7; tau^2 estimator: REML)
##
## tau^2 (estimated amount of total heterogeneity): 0.1213 (SE = 0.0985)
## tau (square root of estimated tau^2 value): 0.3483
## I^2 (total heterogeneity / total variability): 95.23%
## H^2 (total variability / sampling variability): 20.94
##
## Test for Heterogeneity:
## Q(df = 6) = 68.5394, p-val < .0001
##
## Model Results:
##
## estimate se zval pval ci.lb ci.ub ​
## -0.3937 0.1620 -2.4310 0.0151 -0.7111 -0.0763 *
##
## ---
## Signif. codes: 0 '***' 0.001 '**' 0.01 '*' 0.05 '.' 0.1 ' ' 1


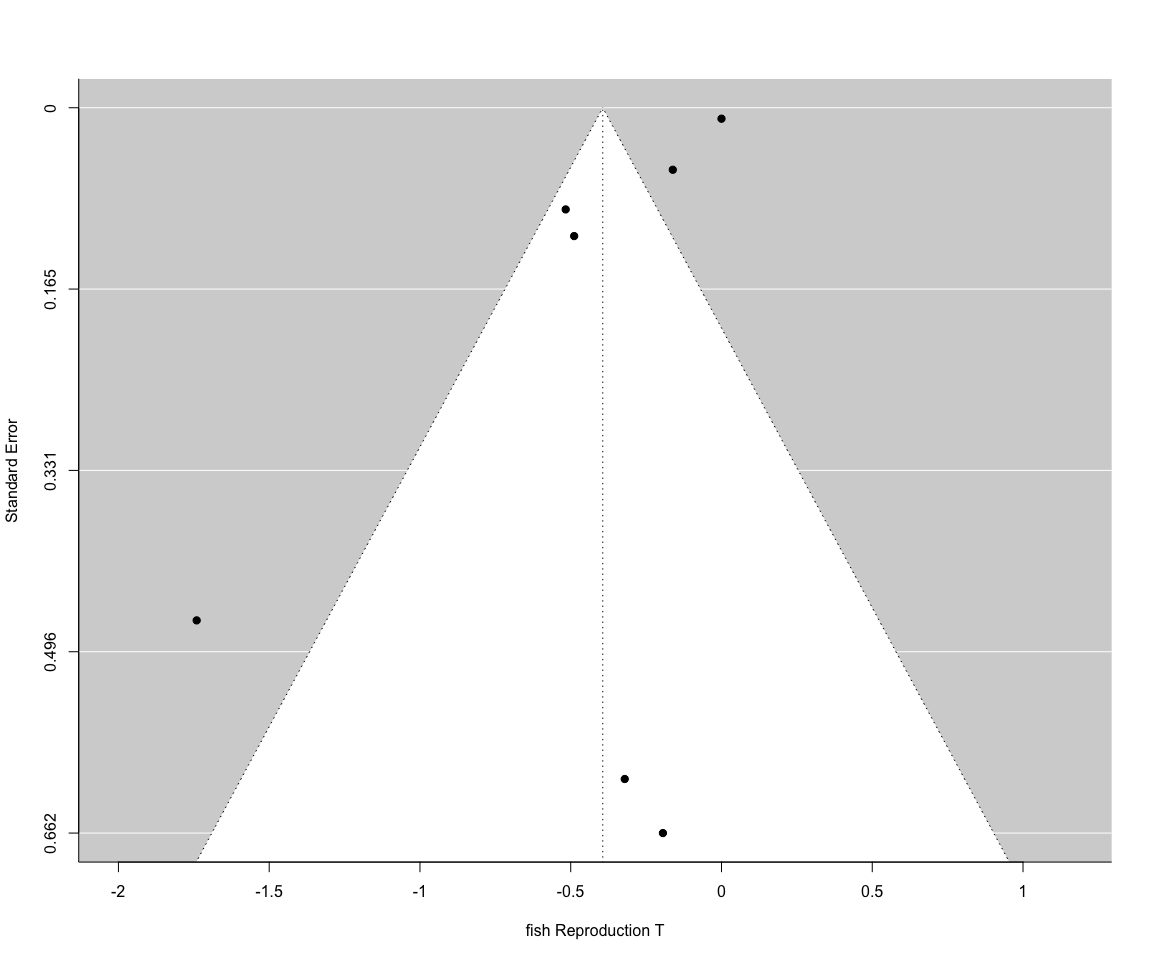

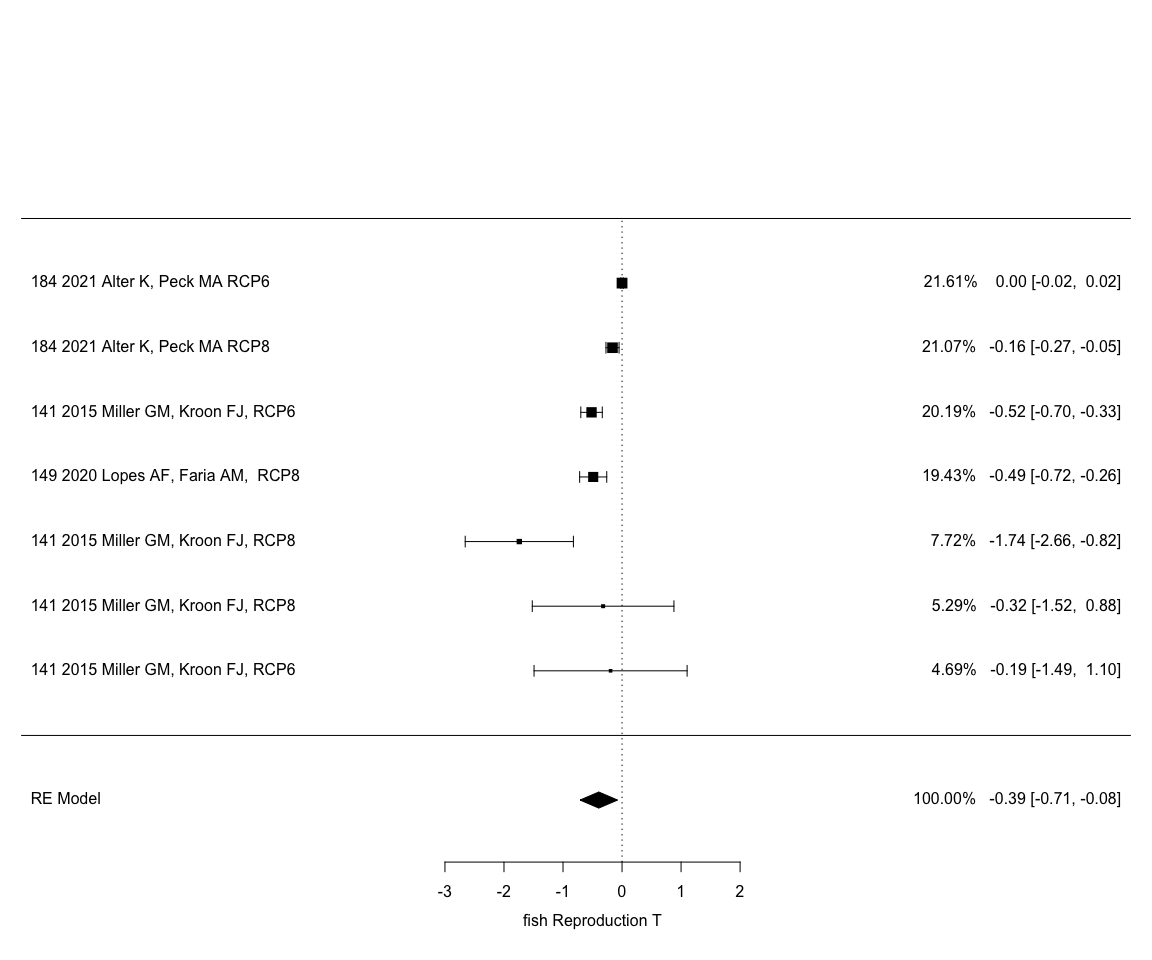


##
## Random-Effects Model (k = 6; tau^2 estimator: REML)
##
## tau^2 (estimated amount of total heterogeneity): 0.0937 (SE = 0.0767)
## tau (square root of estimated tau^2 value): 0.3061
## I^2 (total heterogeneity / total variability): 96.60%
## H^2 (total variability / sampling variability): 29.45
##
## Test for Heterogeneity:
## Q(df = 5) = 29.8652, p-val < .0001
##
## Model Results:
##
## estimate se zval pval ci.lb ci.ub ​
## 0.0371 0.1465 0.2535 0.7999 -0.2500 0.3243
##
## ---
## Signif. codes: 0 '***' 0.001 '**' 0.01 '*' 0.05 '.' 0.1 ' ' 1


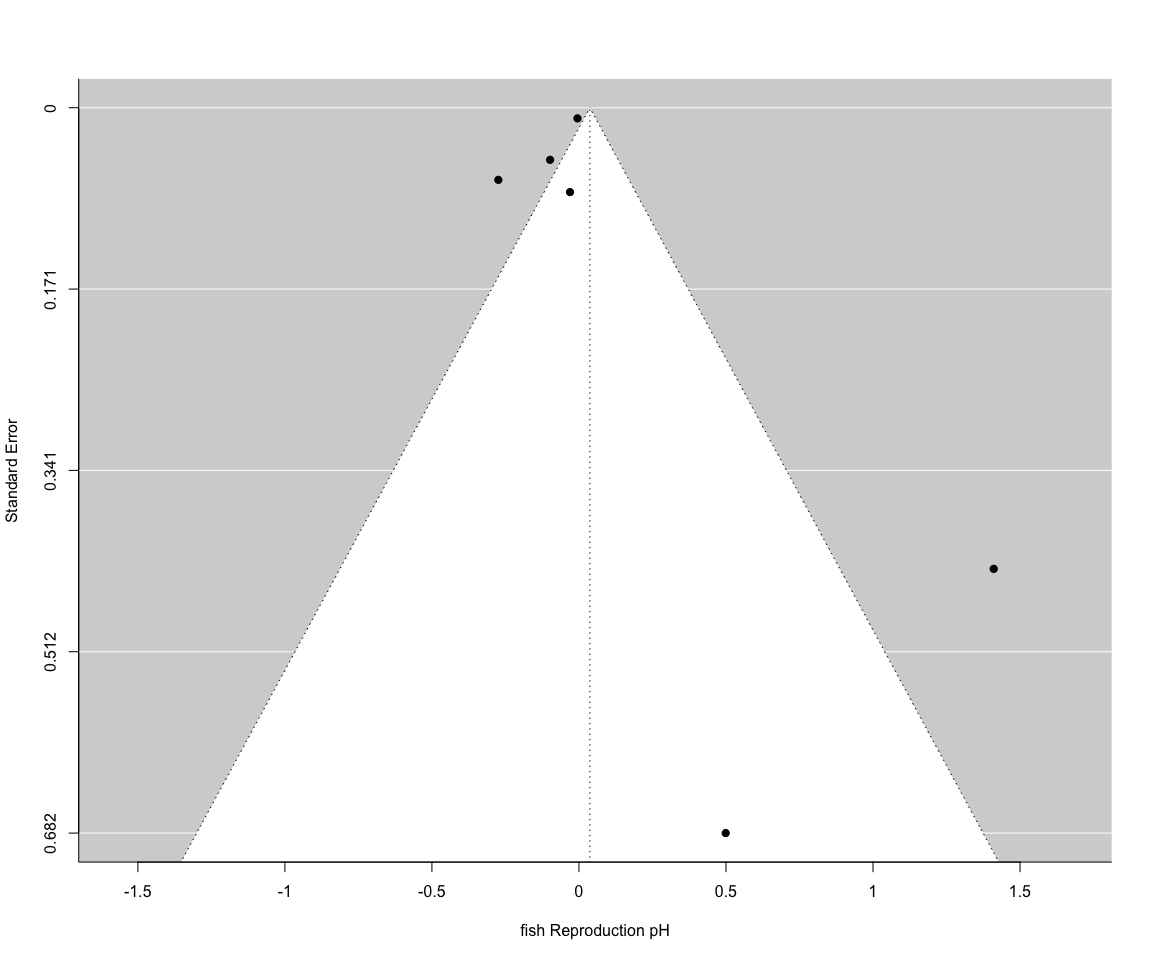

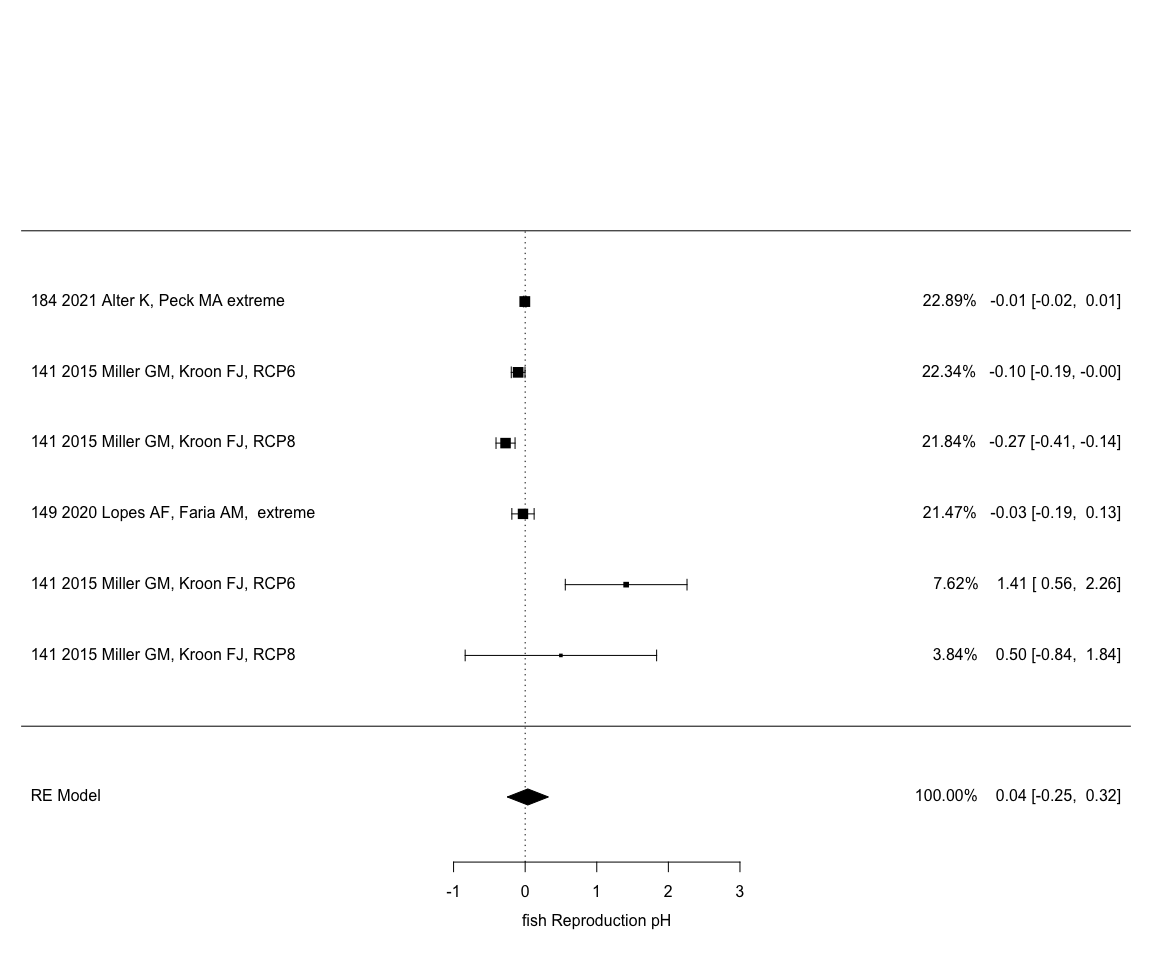


##
## Random-Effects Model (k = 10; tau^2 estimator: REML)
##
## tau^2 (estimated amount of total heterogeneity): 0.0879 (SE = 0.0650)
## tau (square root of estimated tau^2 value): 0.2966
## I^2 (total heterogeneity / total variability): 96.97%
## H^2 (total variability / sampling variability): 33.04
##
## Test for Heterogeneity:
## Q(df = 9) = 66.1860, p-val < .0001
##
## Model Results:
##
## estimate se zval pval ci.lb ci.ub ​
## -0.2391 0.1225 -1.9510 0.0511 -0.4792 0.0011 .
##
## ---
## Signif. codes: 0 '***' 0.001 '**' 0.01 '*' 0.05 '.' 0.1 ' ' 1


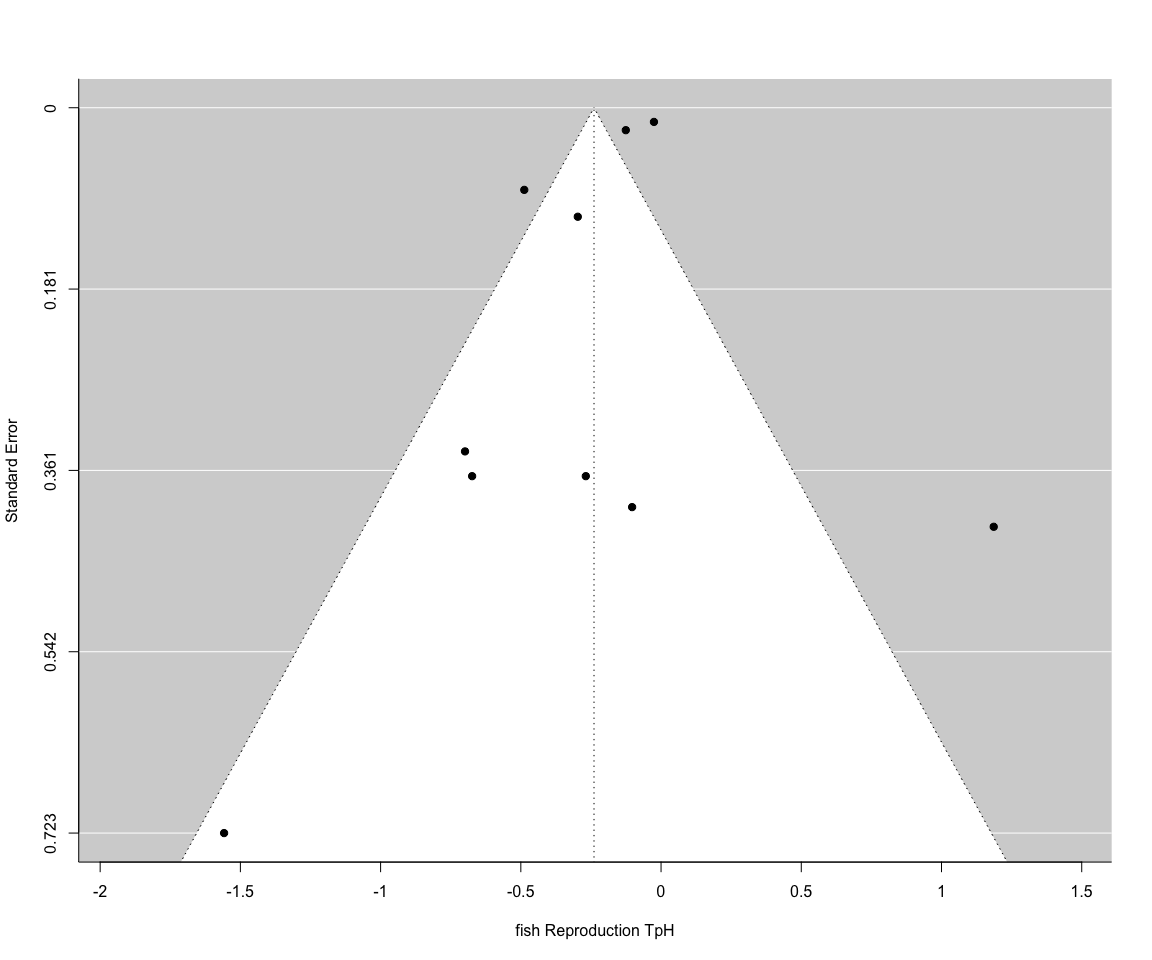

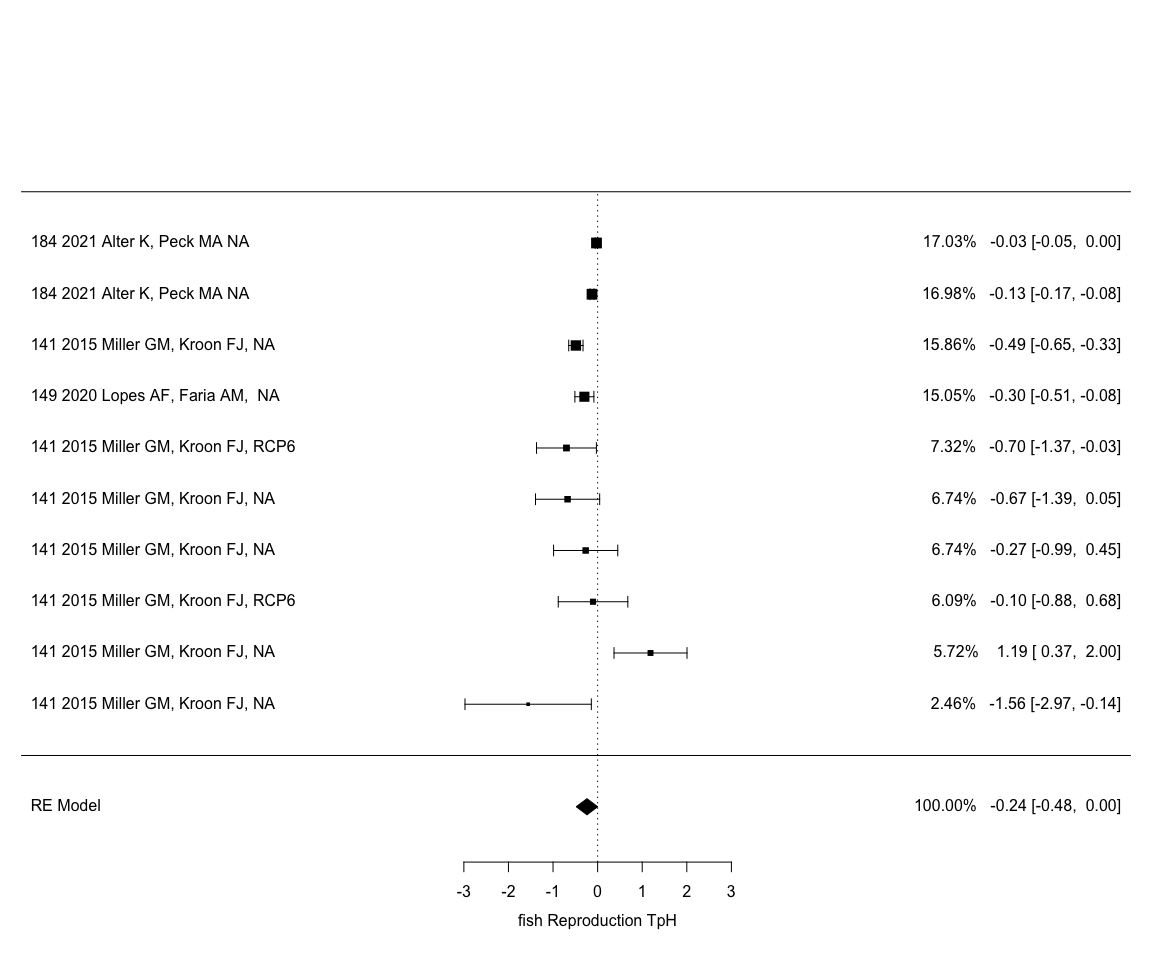


Abs_FishRepro <- MA_TpH_abs("fish","Reproduction", Fish)

## Invertebrate, Behavior
InvertBehav <- MA_TpH("inverts","Behavior", Inverts,sensitivity)

##
## Random-Effects Model (k = 48; tau^2 estimator: REML)
##
## tau^2 (estimated amount of total heterogeneity): 0.2445 (SE = 0.0597)
## tau (square root of estimated tau^2 value): 0.4944
## I^2 (total heterogeneity / total variability): 97.26%
## H^2 (total variability / sampling variability): 36.43
##
## Test for Heterogeneity:
## Q(df = 47) = 1040.3365, p-val < .0001
##
## Model Results:
##
## estimate se zval pval ci.lb ci.ub ​
## 0.0368 0.0783 0.4703 0.6381 -0.1166 0.1903
##
## ---
## Signif. codes: 0 '***' 0.001 '**' 0.01 '*' 0.05 '.' 0.1 ' ' 1


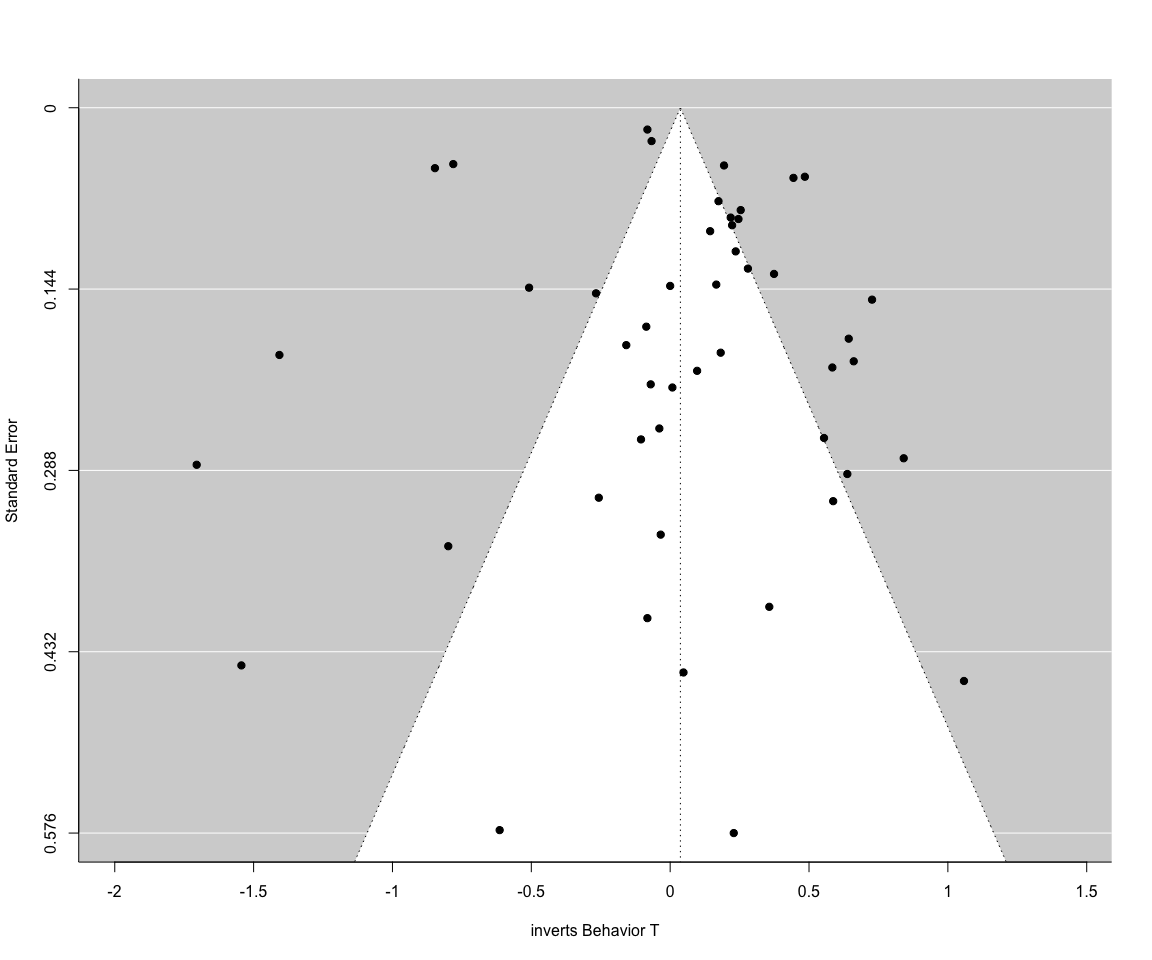

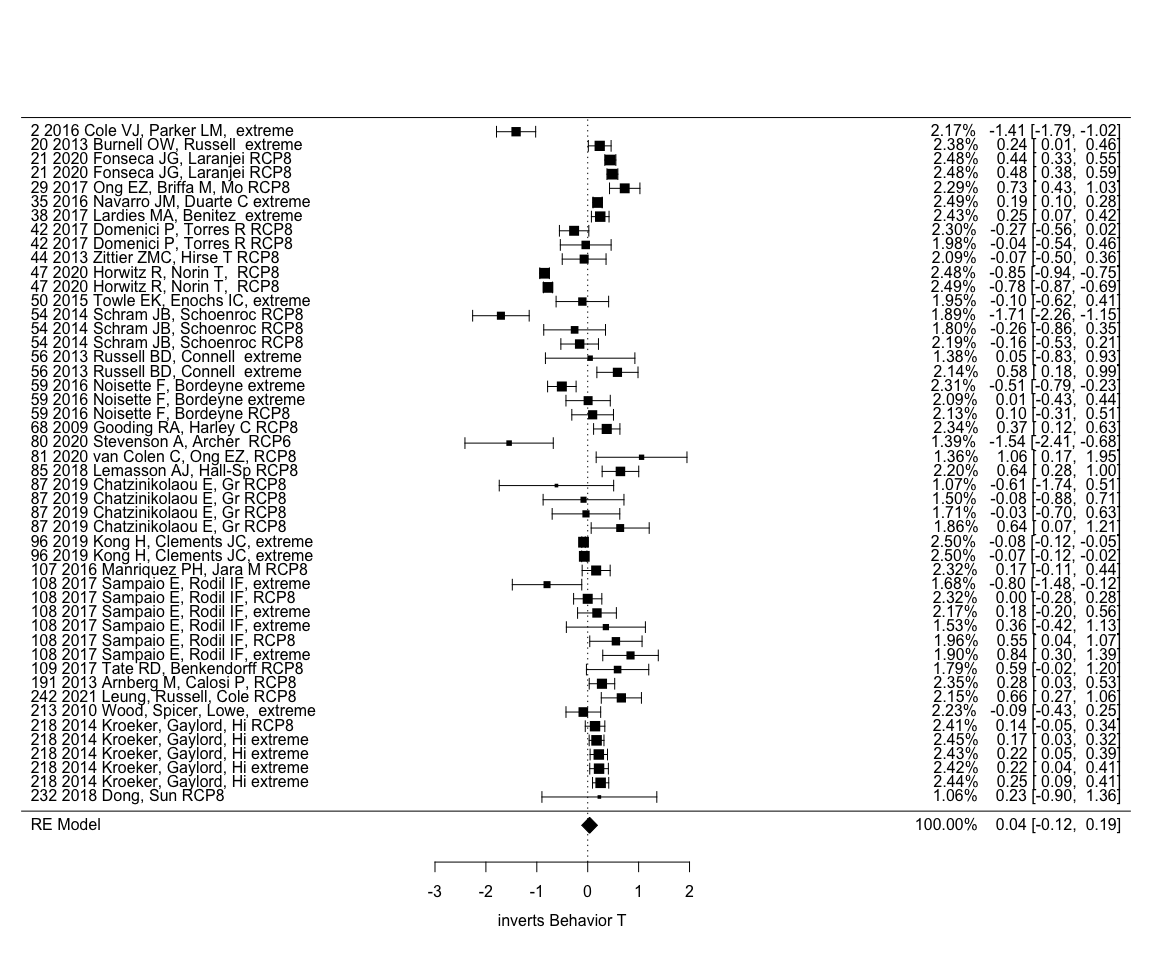


##
## Random-Effects Model (k = 46; tau^2 estimator: REML)
##
## tau^2 (estimated amount of total heterogeneity): 0.2335 (SE = 0.0613)
## tau (square root of estimated tau^2 value): 0.4832
## I^2 (total heterogeneity / total variability): 95.97%
## H^2 (total variability / sampling variability): 24.80
##
## Test for Heterogeneity:
## Q(df = 45) = 385.2587, p-val < .0001
##
## Model Results:
##
## estimate se zval pval ci.lb ci.ub ​
## -0.2175 0.0808 -2.6923 0.0071 -0.3759 -0.0592 **
##
## ---
## Signif. codes: 0 '***' 0.001 '**' 0.01 '*' 0.05 '.' 0.1 ' ' 1


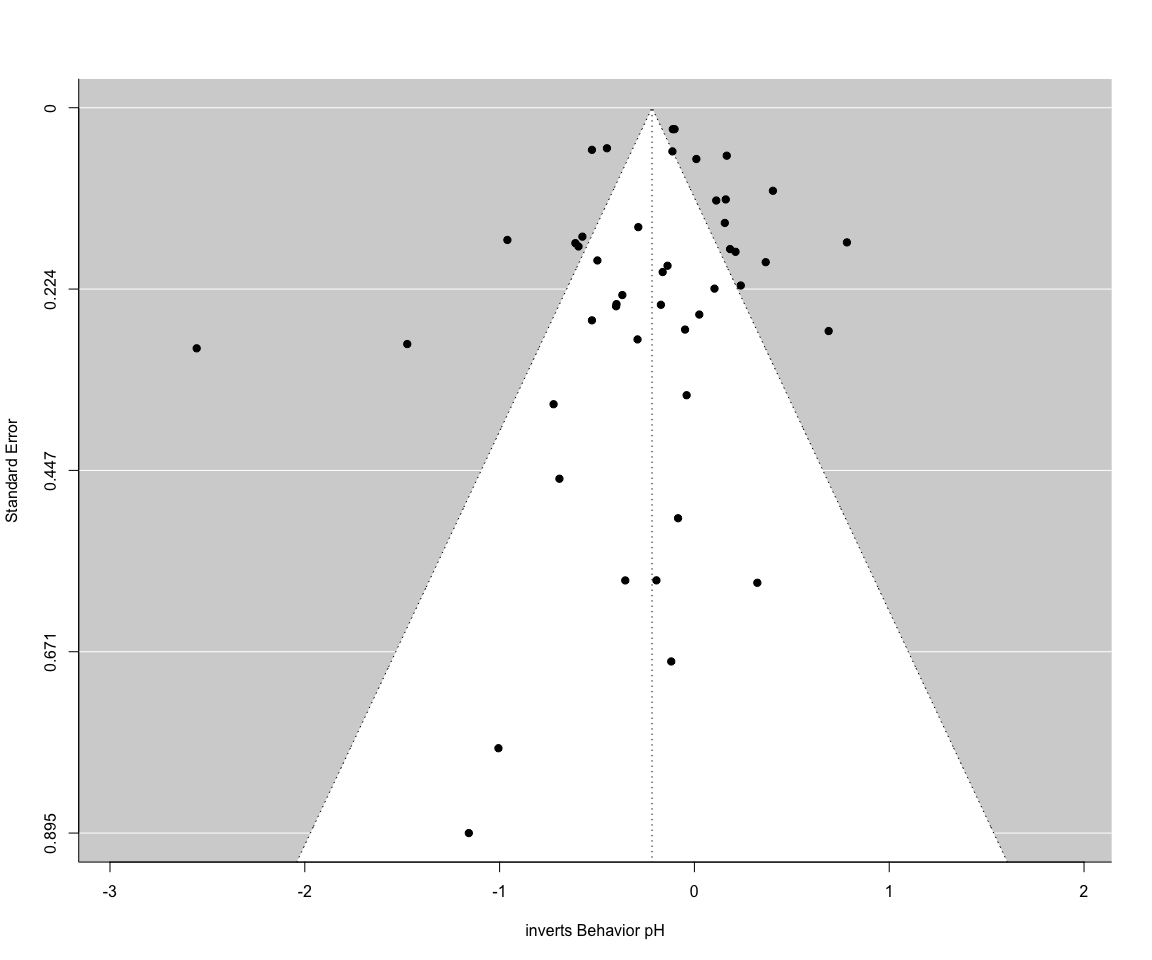

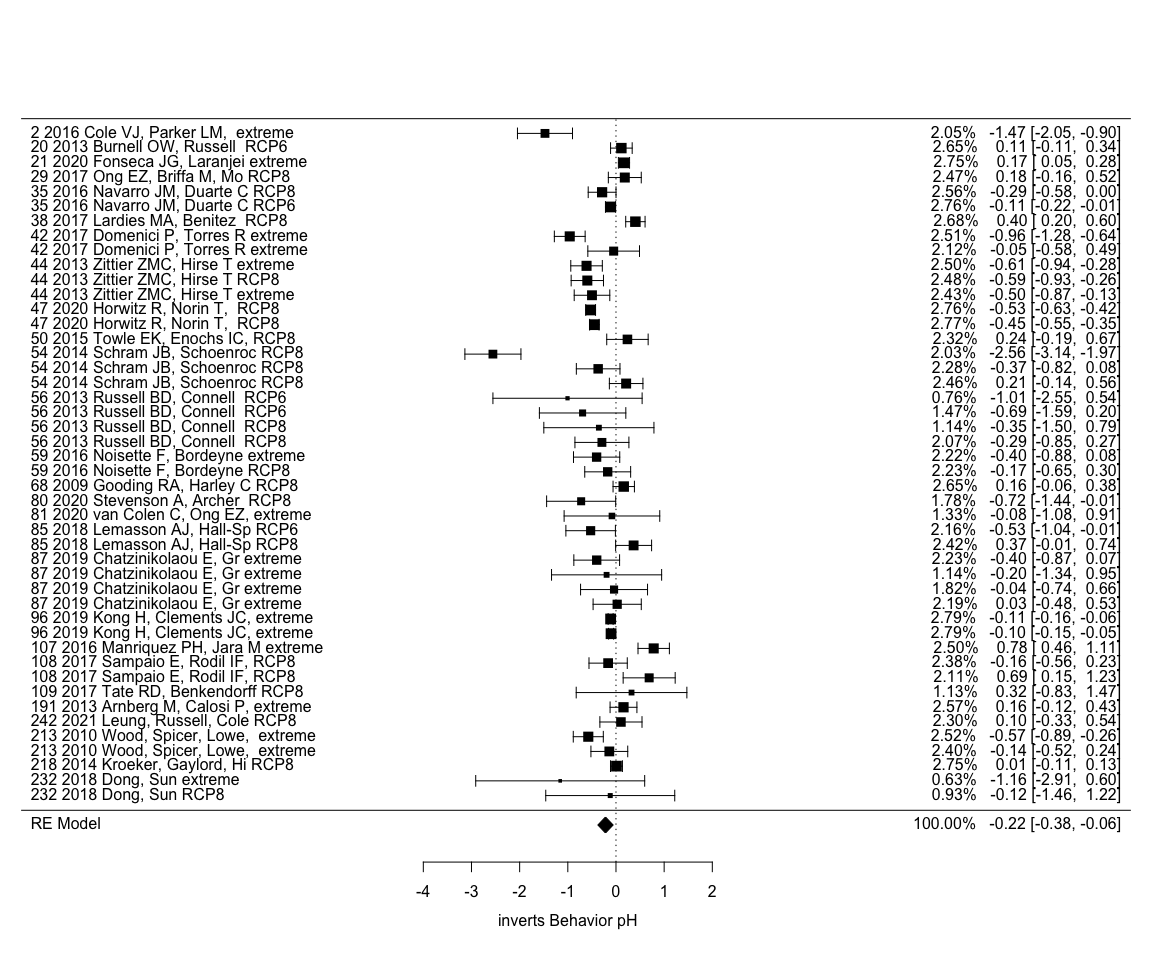


##
## Random-Effects Model (k = 59; tau^2 estimator: REML)
##
## tau^2 (estimated amount of total heterogeneity): 0.2088 (SE = 0.0472)
## tau (square root of estimated tau^2 value): 0.4569
## I^2 (total heterogeneity / total variability): 95.99%
## H^2 (total variability / sampling variability): 24.94
##
## Test for Heterogeneity:
## Q(df = 58) = 1338.3536, p-val < .0001
##
## Model Results:
##
## estimate se zval pval ci.lb ci.ub ​
## 0.0329 0.0664 0.4949 0.6207 -0.0973 0.1631
##
## ---
## Signif. codes: 0 '***' 0.001 '**' 0.01 '*' 0.05 '.' 0.1 ' ' 1


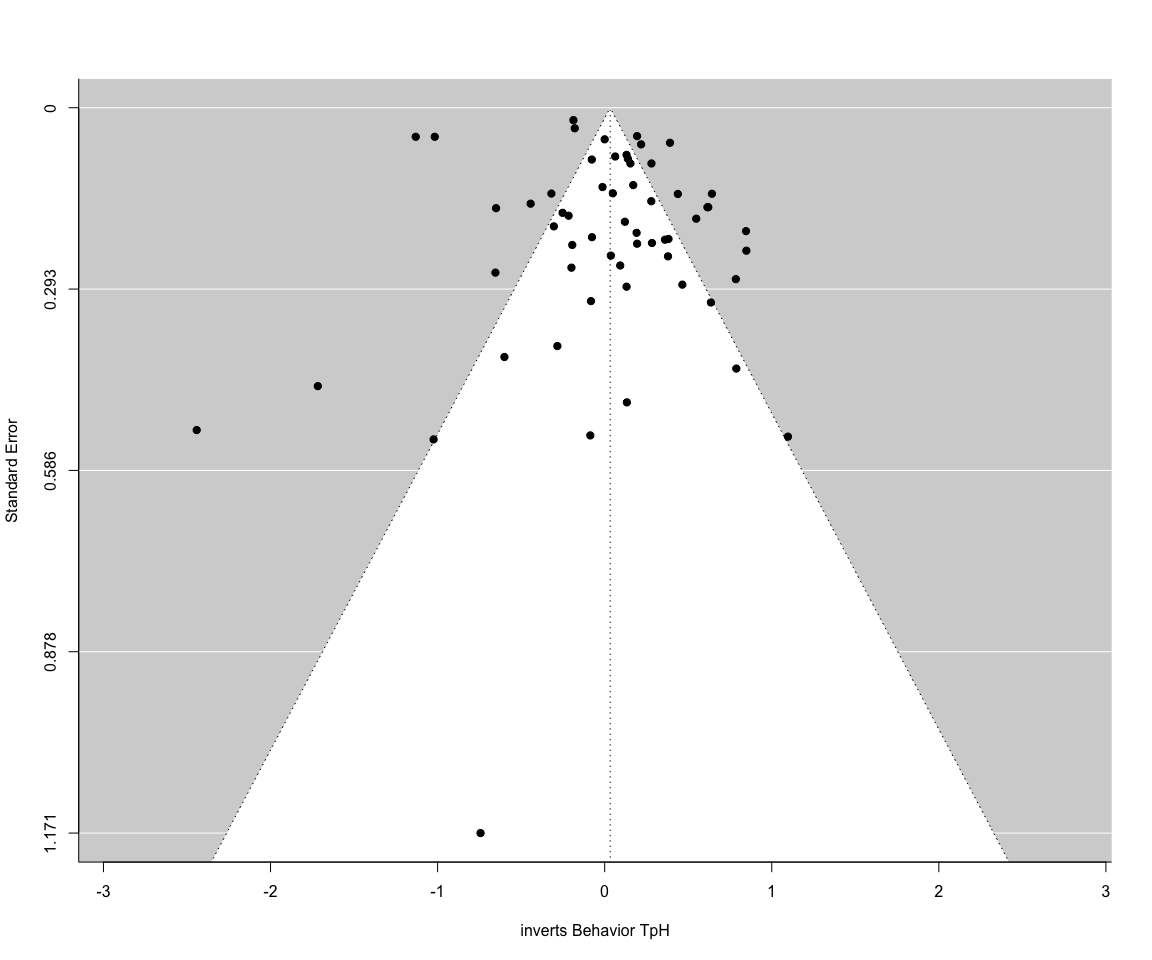

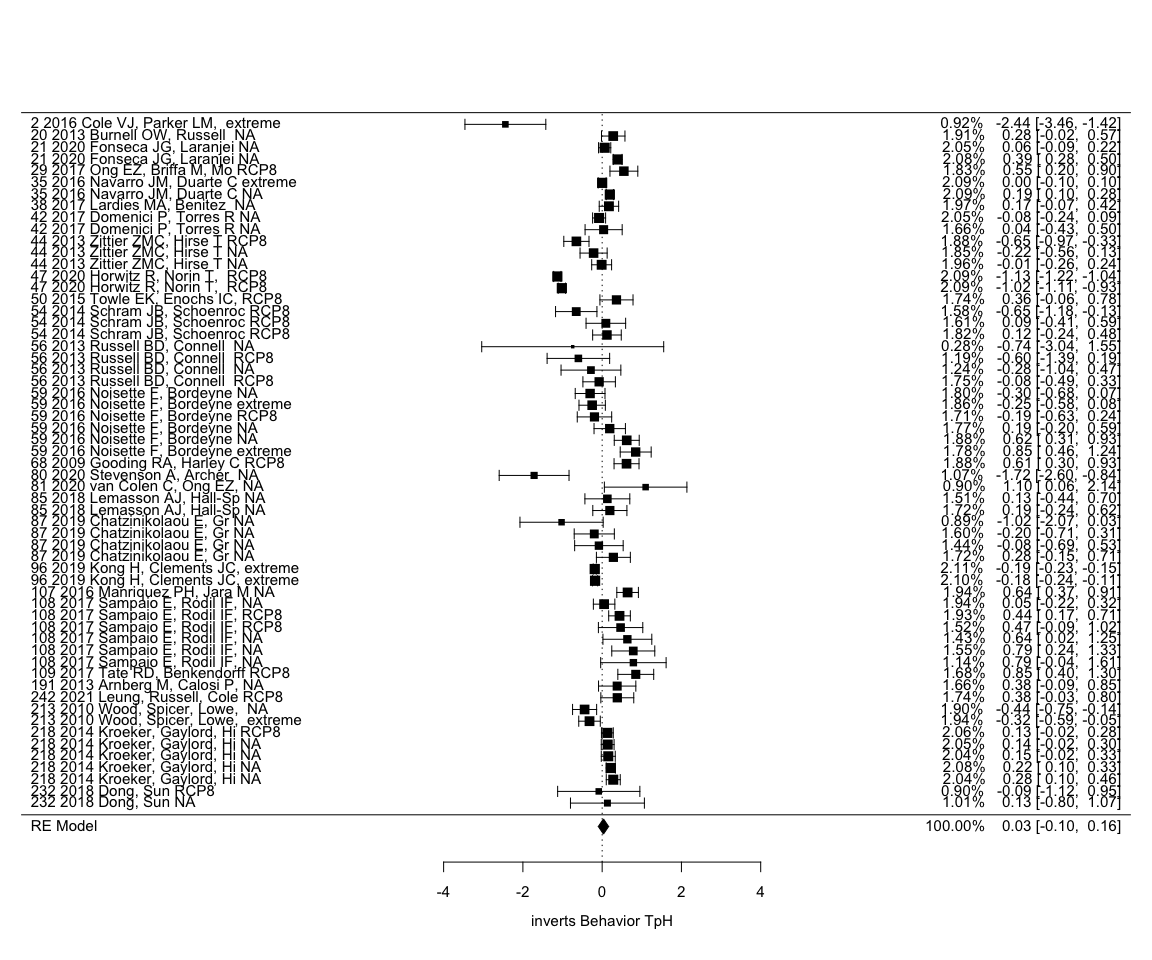


Abs_InvertBehav <- MA_TpH_abs("inverts","Behavior", Inverts)

## Fish, Behavior
FishBehav <- MA_TpH("fish","Behaviour", Fish,sensitivity)

##
## Random-Effects Model (k = 40; tau^2 estimator: REML)
##
## tau^2 (estimated amount of total heterogeneity): 0.1323 (SE = 0.0365)
## tau (square root of estimated tau^2 value): 0.3637
## I^2 (total heterogeneity / total variability): 92.65%
## H^2 (total variability / sampling variability): 13.61
##
## Test for Heterogeneity:
## Q(df = 39) = 353.0461, p-val < .0001
##
## Model Results:
##
## estimate se zval pval ci.lb ci.ub ​
## 0.0757 0.0642 1.1782 0.2387 -0.0502 0.2015
##
## ---
## Signif. codes: 0 '***' 0.001 '**' 0.01 '*' 0.05 '.' 0.1 ' ' 1


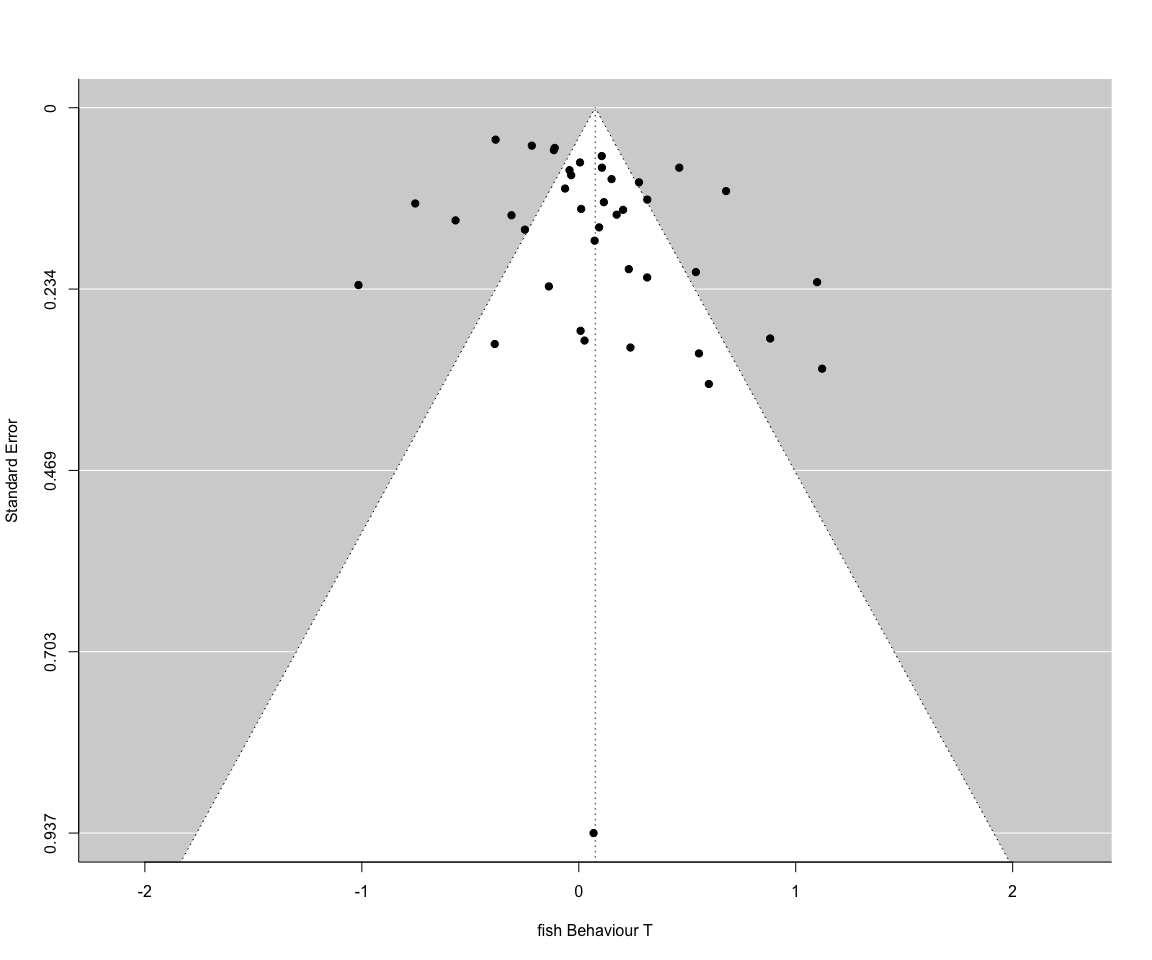

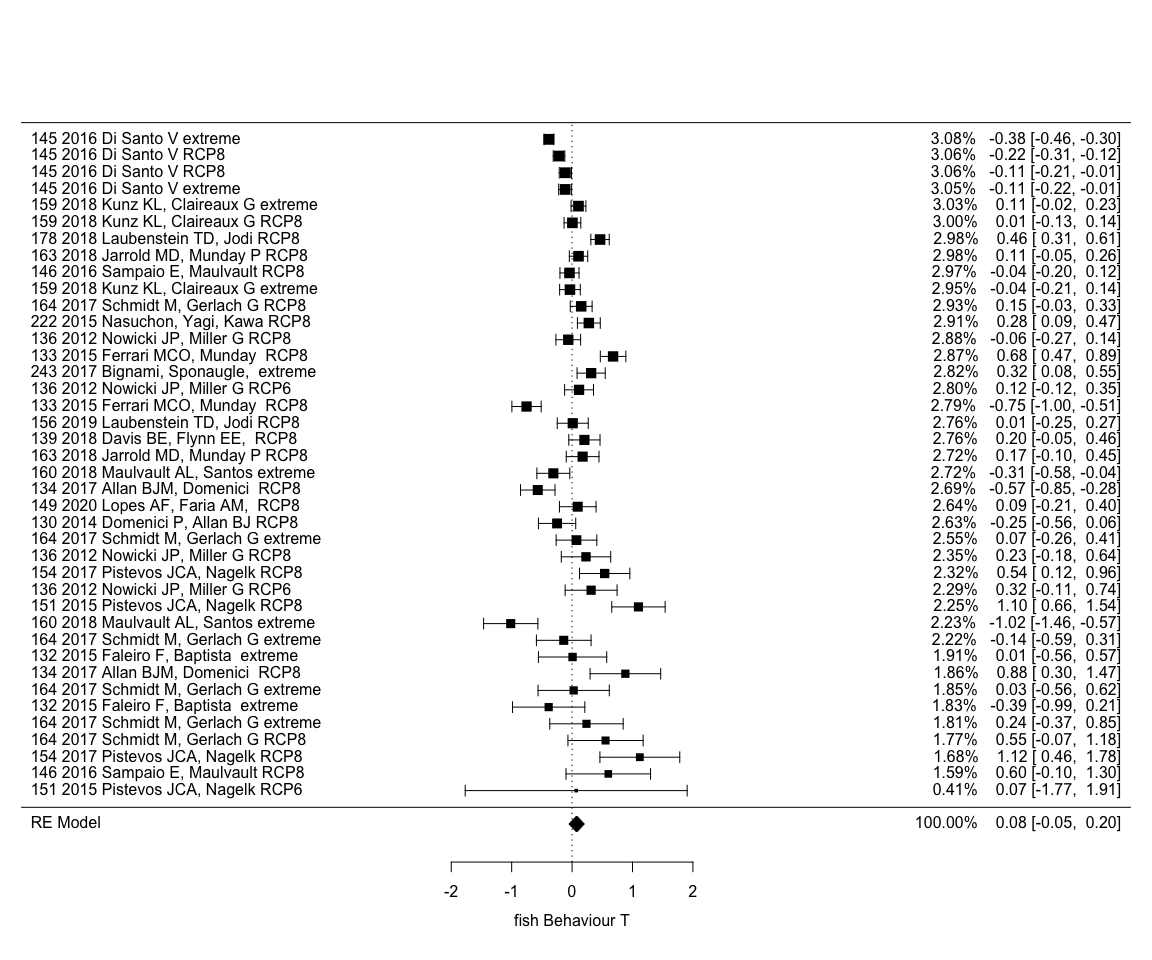


##
## Random-Effects Model (k = 35; tau^2 estimator: REML)
##
## tau^2 (estimated amount of total heterogeneity): 0.1930 (SE = 0.0561)
## tau (square root of estimated tau^2 value): 0.4393
## I^2 (total heterogeneity / total variability): 92.56%
## H^2 (total variability / sampling variability): 13.44
##
## Test for Heterogeneity:
## Q(df = 34) = 264.8158, p-val < .0001
##
## Model Results:
##
## estimate se zval pval ci.lb ci.ub ​
## -0.0702 0.0820 -0.8556 0.3922 -0.2309 0.0906
##
## ---
## Signif. codes: 0 '***' 0.001 '**' 0.01 '*' 0.05 '.' 0.1 ' ' 1


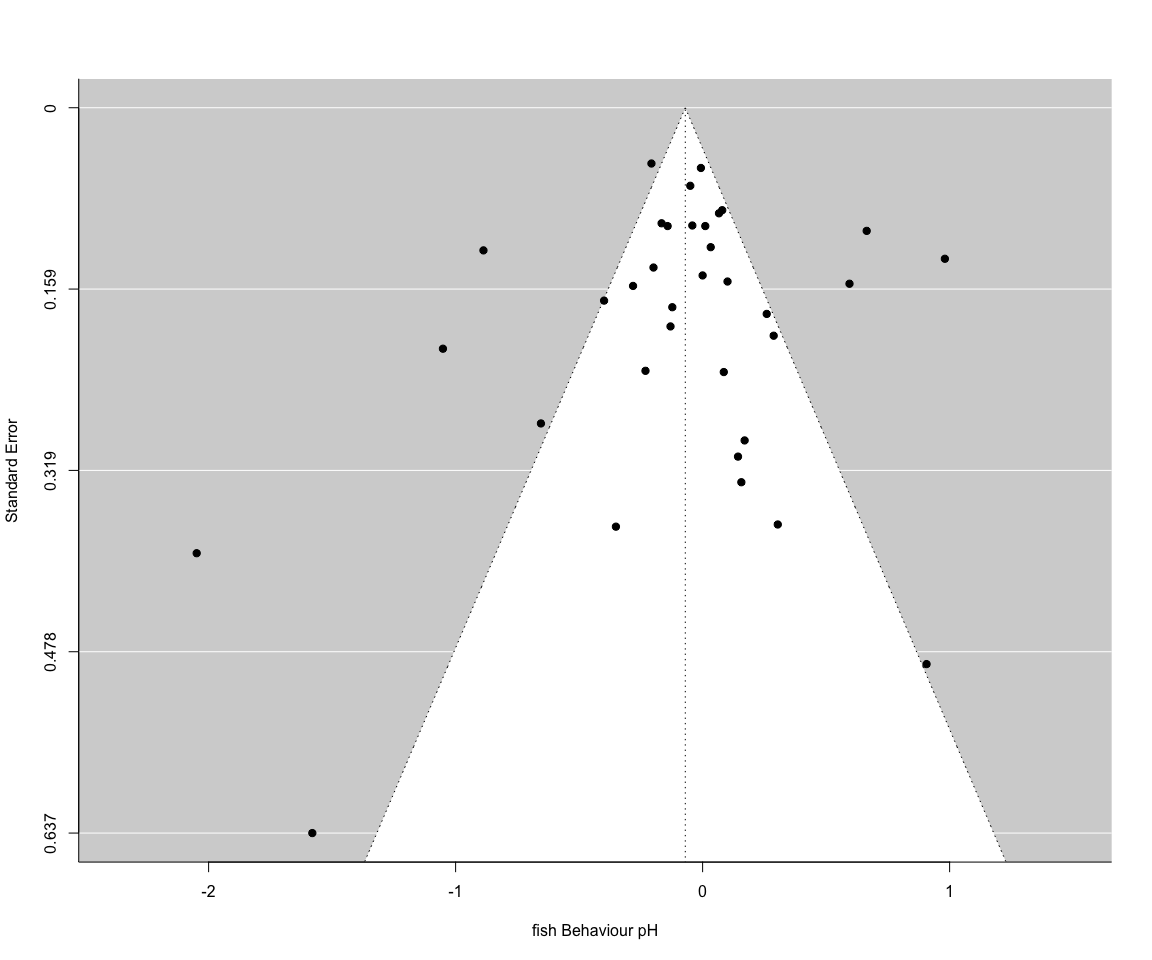

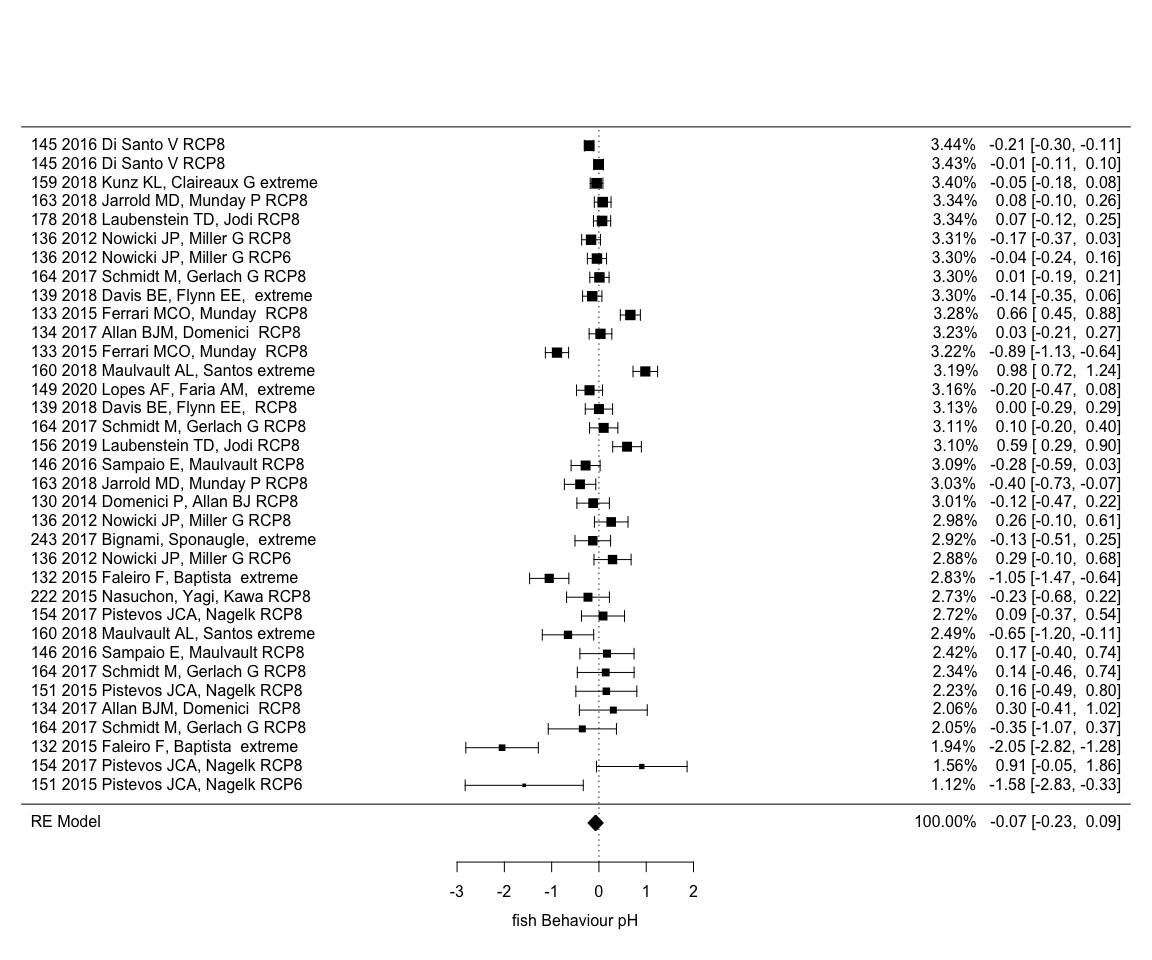


##
## Random-Effects Model (k = 45; tau^2 estimator: REML)
##
## tau^2 (estimated amount of total heterogeneity): 0.1374 (SE = 0.0353)
## tau (square root of estimated tau^2 value): 0.3706
## I^2 (total heterogeneity / total variability): 93.44%
## H^2 (total variability / sampling variability): 15.24
##
## Test for Heterogeneity:
## Q(df = 44) = 384.3174, p-val < .0001
##
## Model Results:
##
## estimate se zval pval ci.lb ci.ub ​
## 0.0356 0.0613 0.5807 0.5614 -0.0845 0.1557
##
## ---
## Signif. codes: 0 '***' 0.001 '**' 0.01 '*' 0.05 '.' 0.1 ' ' 1


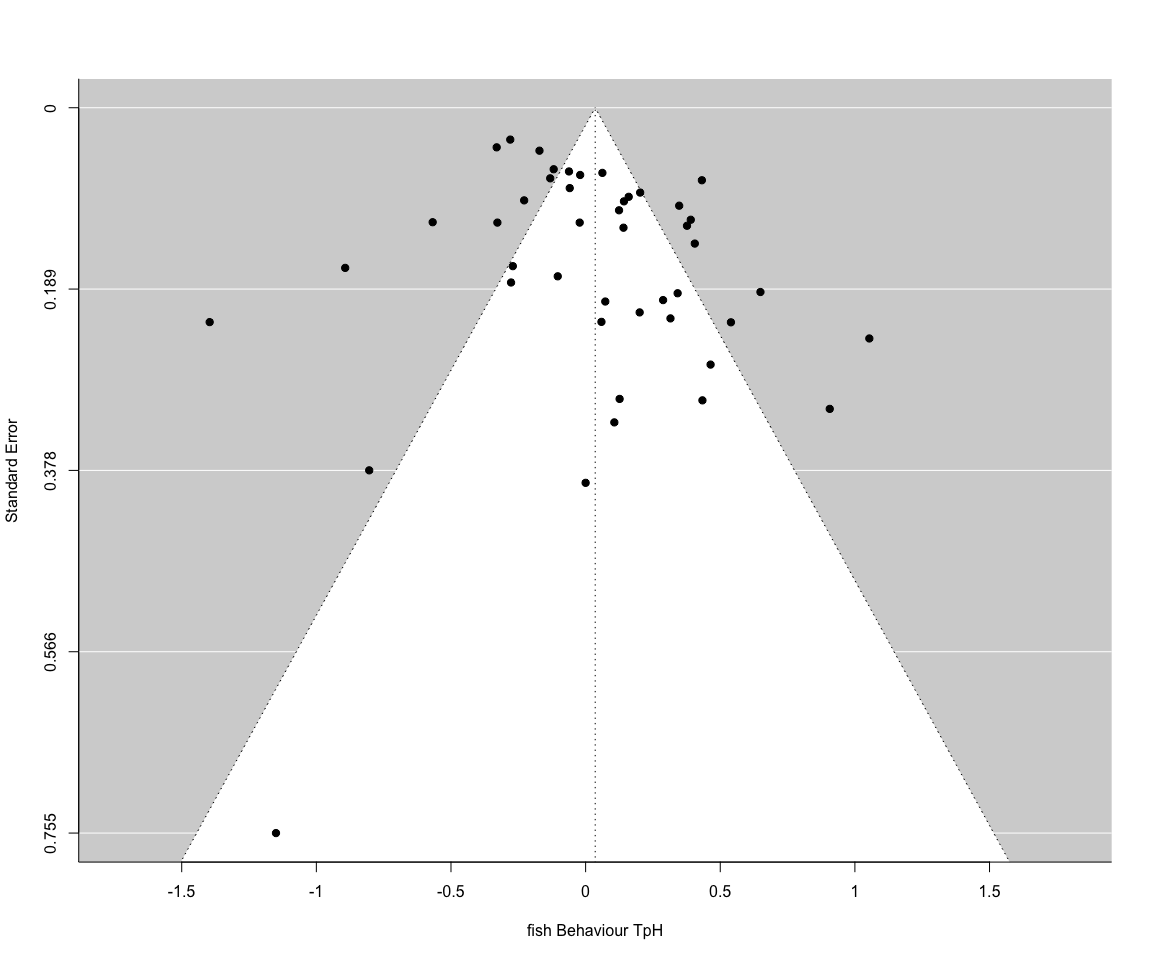

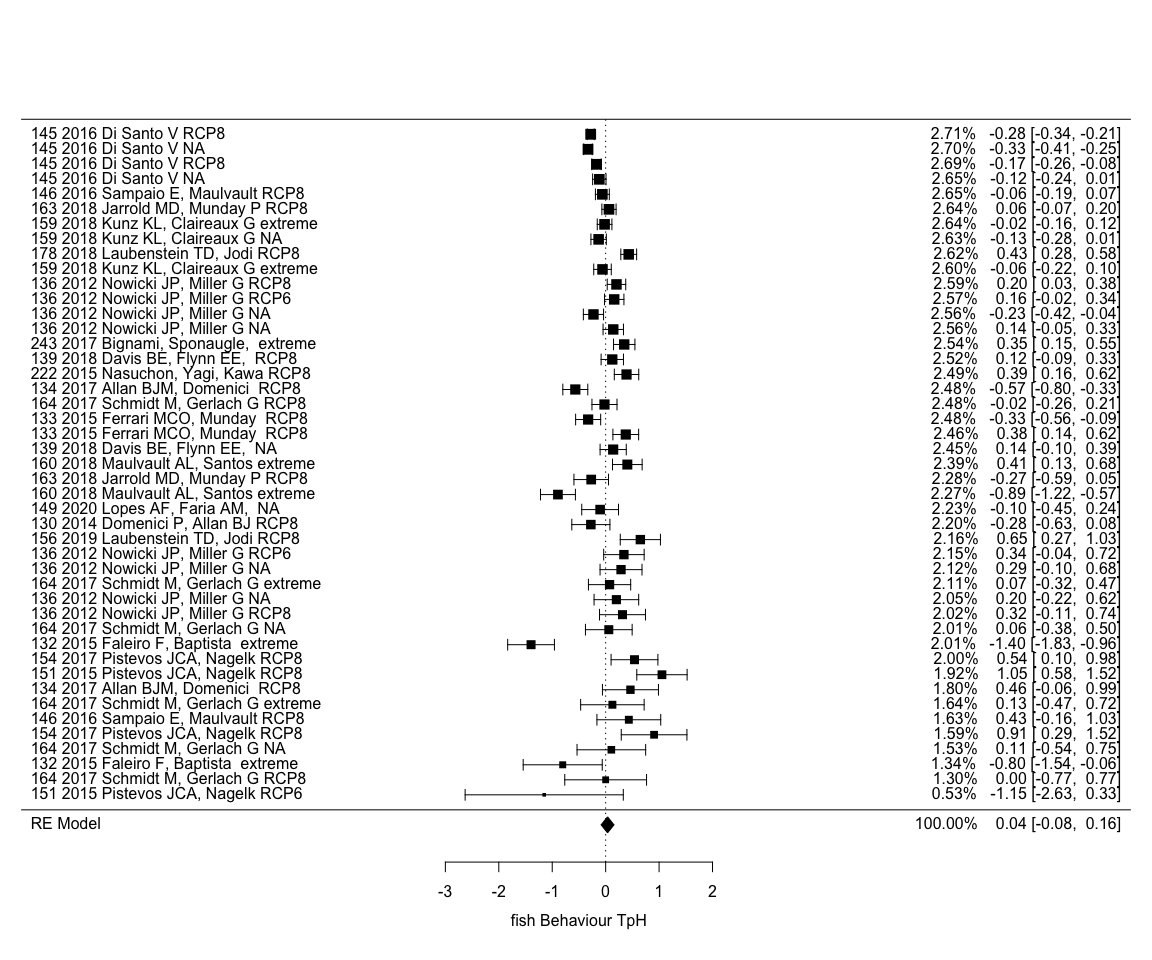


Abs_FishBehav <- MA_TpH_abs("fish","Behaviour", Fish)

## Invertebrate, Biomechanics
InvertBio <- MA_TpH("inverts","Biomechanics", Inverts,sensitivity)

##
## Random-Effects Model (k = 23; tau^2 estimator: REML)
##
## tau^2 (estimated amount of total heterogeneity): 0.0978 (SE = 0.0352)
## tau (square root of estimated tau^2 value): 0.3128
## I^2 (total heterogeneity / total variability): 95.54%
## H^2 (total variability / sampling variability): 22.43
##
## Test for Heterogeneity:
## Q(df = 22) = 212.1644, p-val < .0001
##
## Model Results:
##
## estimate se zval pval ci.lb ci.ub ​
## -0.0895 0.0721 -1.2413 0.2145 -0.2308 0.0518
##
## ---
## Signif. codes: 0 '***' 0.001 '**' 0.01 '*' 0.05 '.' 0.1 ' ' 1


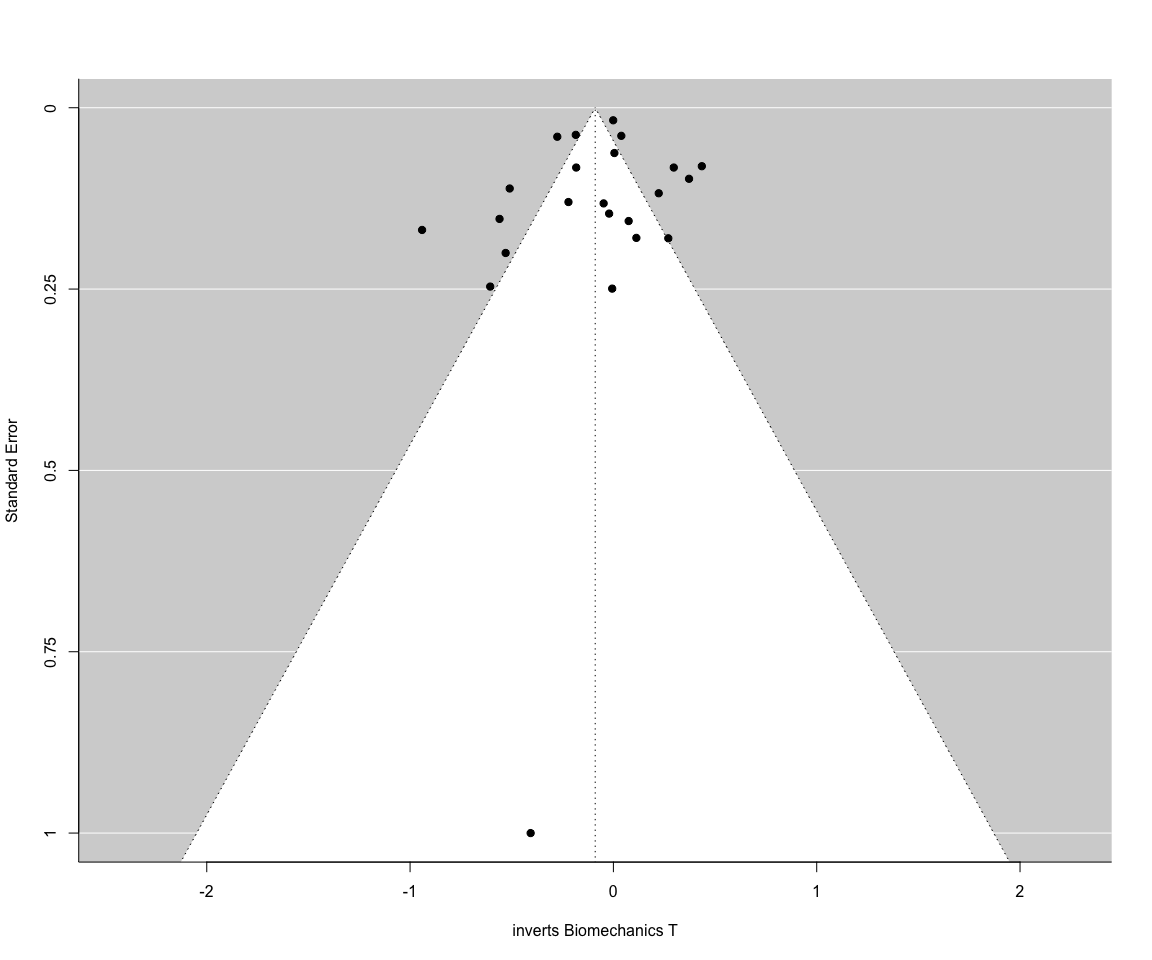

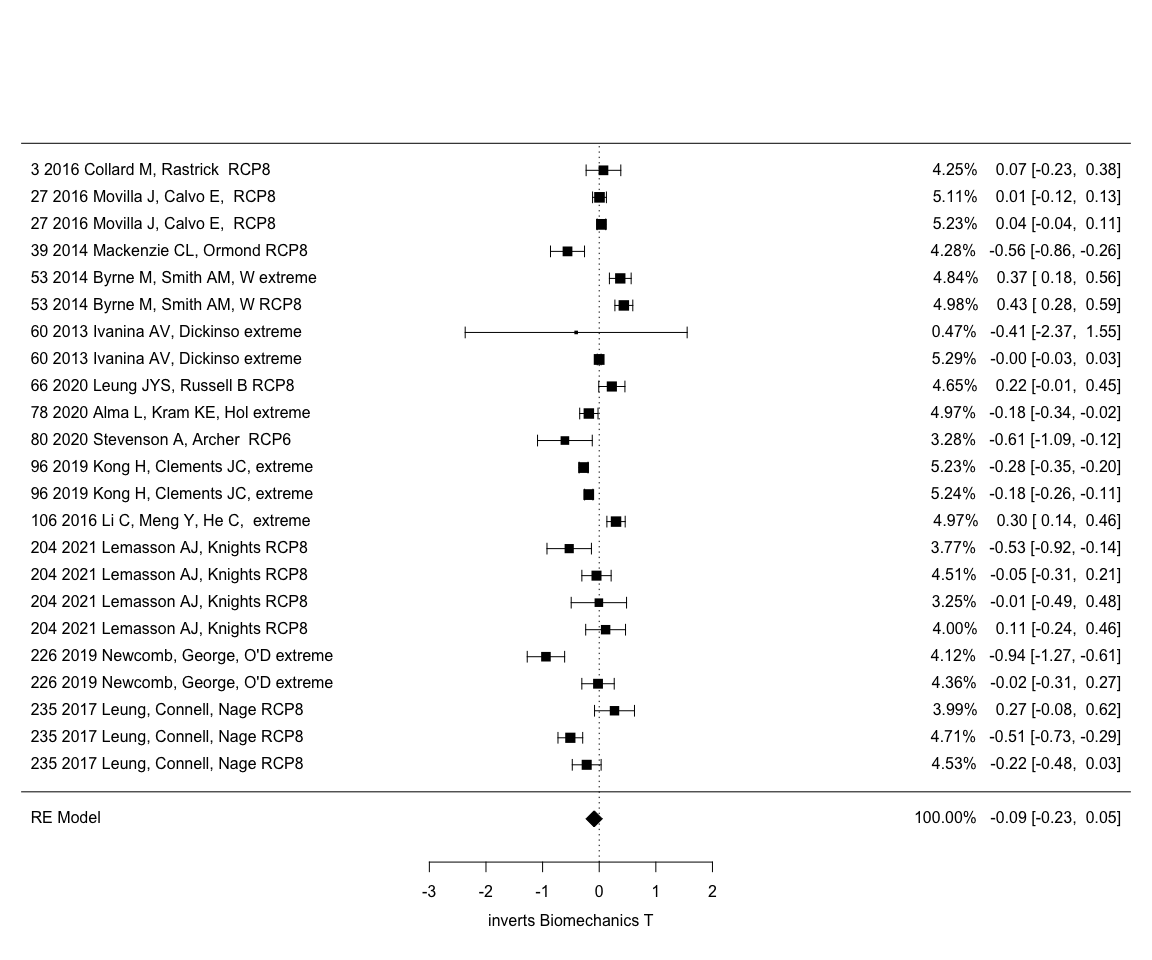


##
## Random-Effects Model (k = 29; tau^2 estimator: REML)
##
## tau^2 (estimated amount of total heterogeneity): 0.0280 (SE = 0.0107)
## tau (square root of estimated tau^2 value): 0.1673
## I^2 (total heterogeneity / total variability): 88.88%
## H^2 (total variability / sampling variability): 9.00
##
## Test for Heterogeneity:
## Q(df = 28) = 154.0990, p-val < .0001
##
## Model Results:
##
## estimate se zval pval ci.lb ci.ub ​
## -0.0918 0.0380 -2.4144 0.0158 -0.1663 -0.0173 *
##
## ---
## Signif. codes: 0 '***' 0.001 '**' 0.01 '*' 0.05 '.' 0.1 ' ' 1


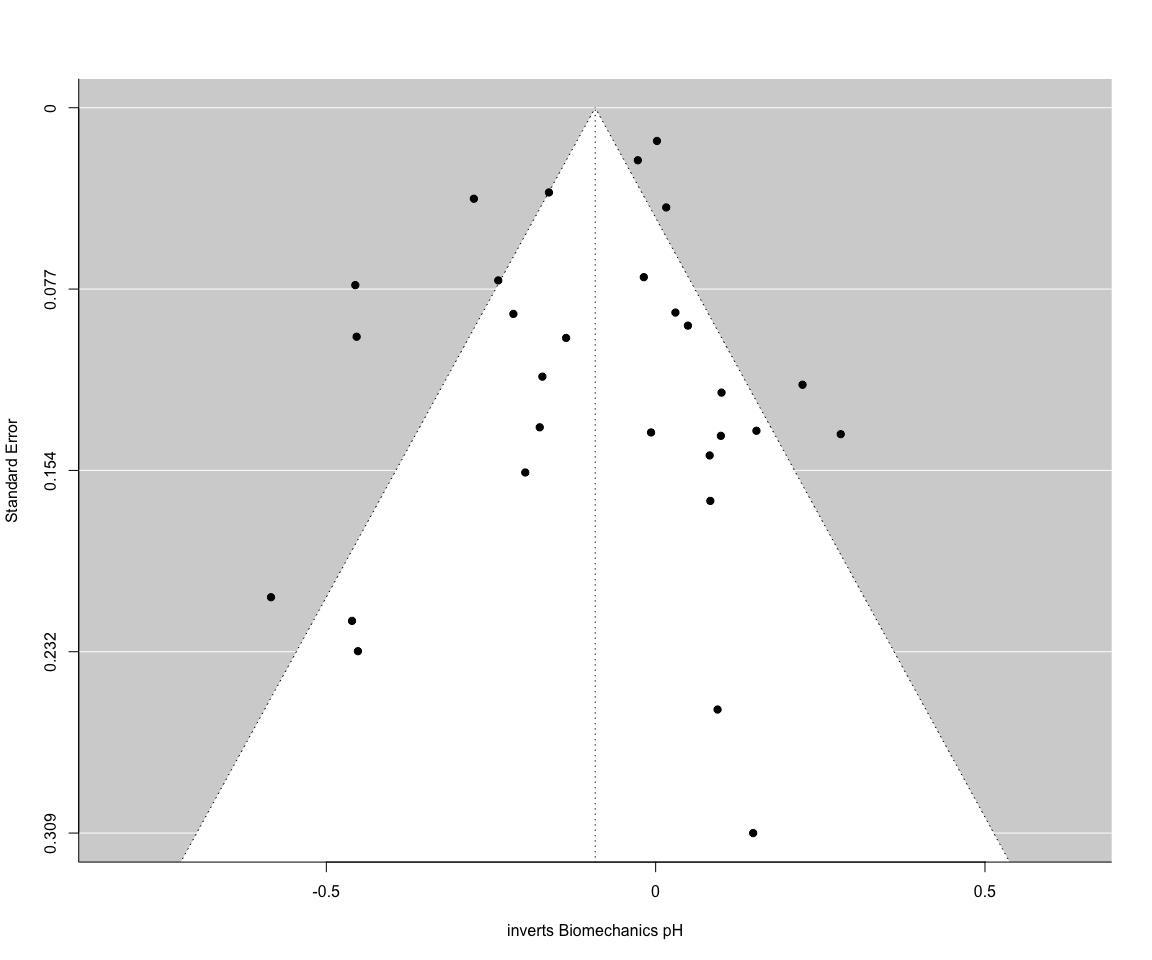

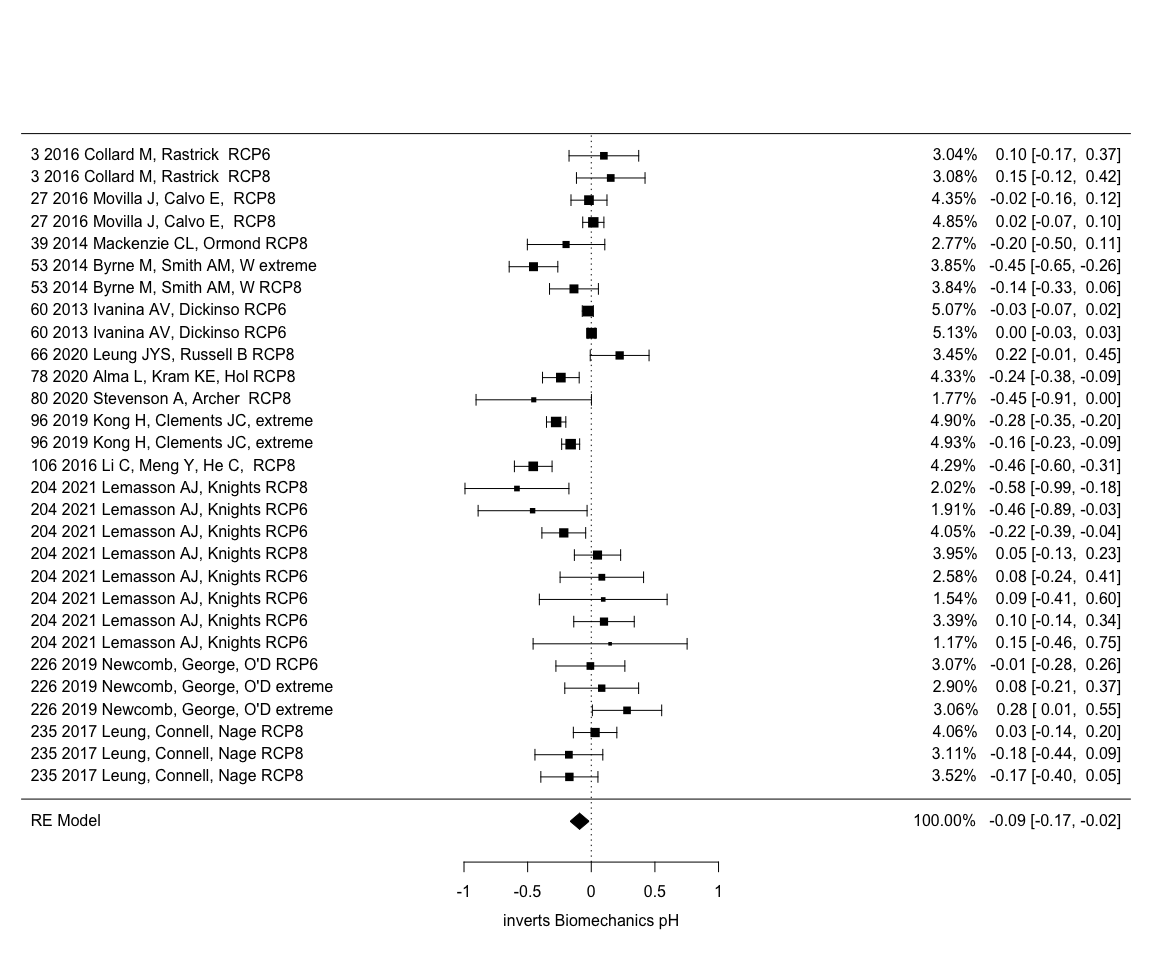


##
## Random-Effects Model (k = 33; tau^2 estimator: REML)
##
## tau^2 (estimated amount of total heterogeneity): 0.1076 (SE = 0.0322)
## tau (square root of estimated tau^2 value): 0.3281
## I^2 (total heterogeneity / total variability): 96.94%
## H^2 (total variability / sampling variability): 32.63
##
## Test for Heterogeneity:
## Q(df = 32) = 391.2367, p-val < .0001
##
## Model Results:
##
## estimate se zval pval ci.lb ci.ub ​
## -0.1440 0.0628 -2.2921 0.0219 -0.2671 -0.0209 *
##
## ---
## Signif. codes: 0 '***' 0.001 '**' 0.01 '*' 0.05 '.' 0.1 ' ' 1


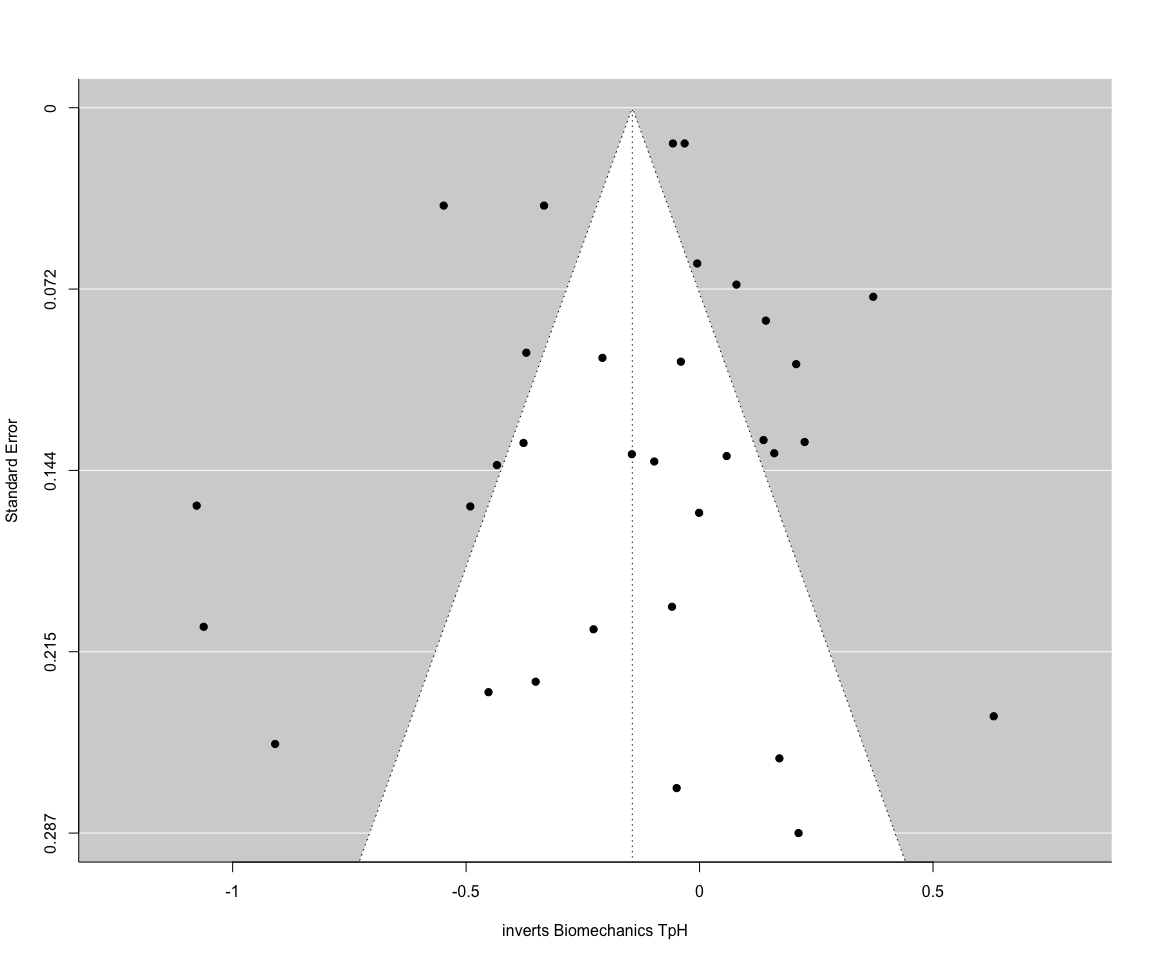

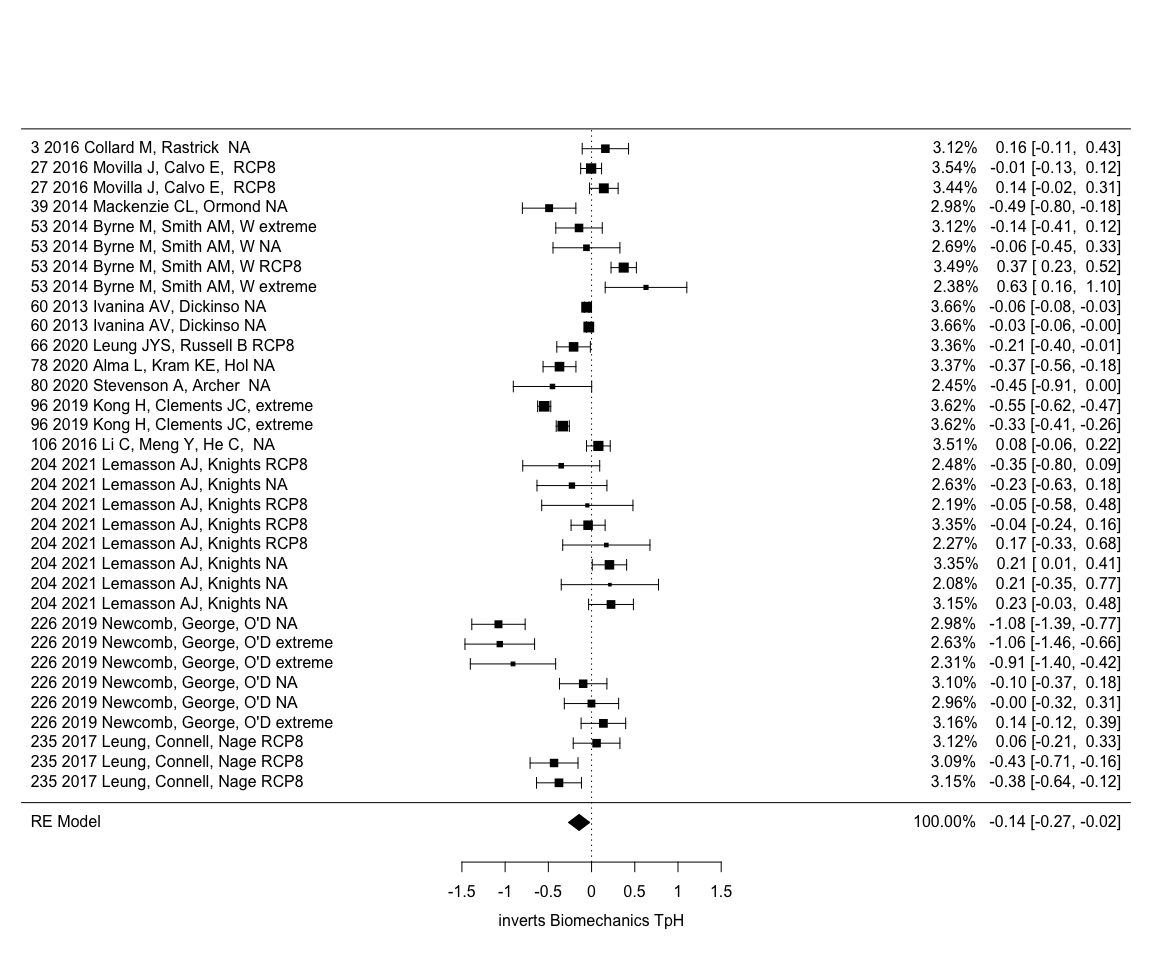


Abs_InvertBio <- MA_TpH_abs("inverts","Biomechanics", Inverts)

## Invertebrate, Development
InvertDev <- MA_TpH("inverts","Development", Inverts,sensitivity)

##
## Random-Effects Model (k = 84; tau^2 estimator: REML)
##
## tau^2 (estimated amount of total heterogeneity): 0.3397 (SE = 0.0577)
## tau (square root of estimated tau^2 value): 0.5828
## I^2 (total heterogeneity / total variability): 99.68%
## H^2 (total variability / sampling variability): 309.42
##
## Test for Heterogeneity:
## Q(df = 83) = 9353.0351, p-val < .0001
##
## Model Results:
##
## estimate se zval pval ci.lb ci.ub ​
## -0.1243 0.0674 -1.8445 0.0651 -0.2563 0.0078 .
##
## ---
## Signif. codes: 0 '***' 0.001 '**' 0.01 '*' 0.05 '.' 0.1 ' ' 1


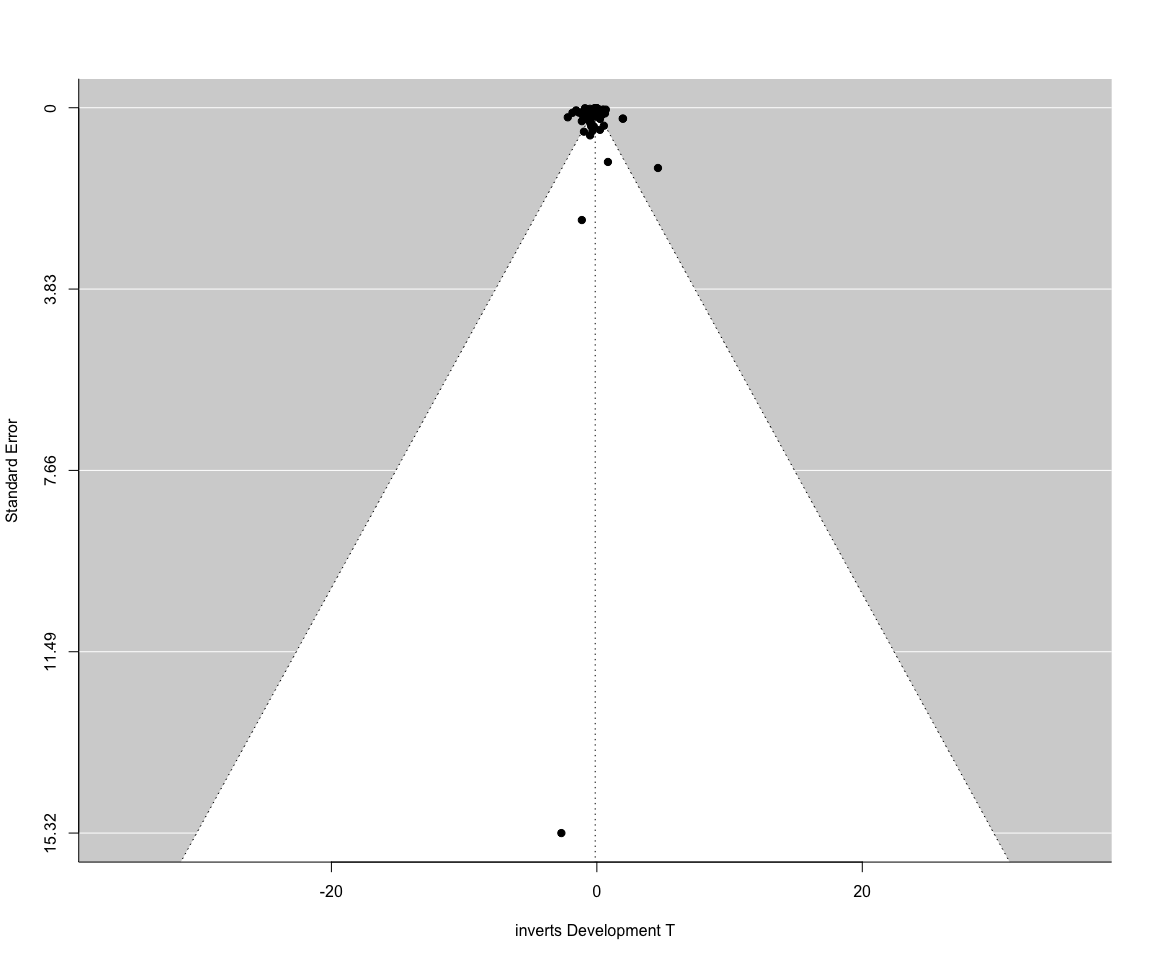

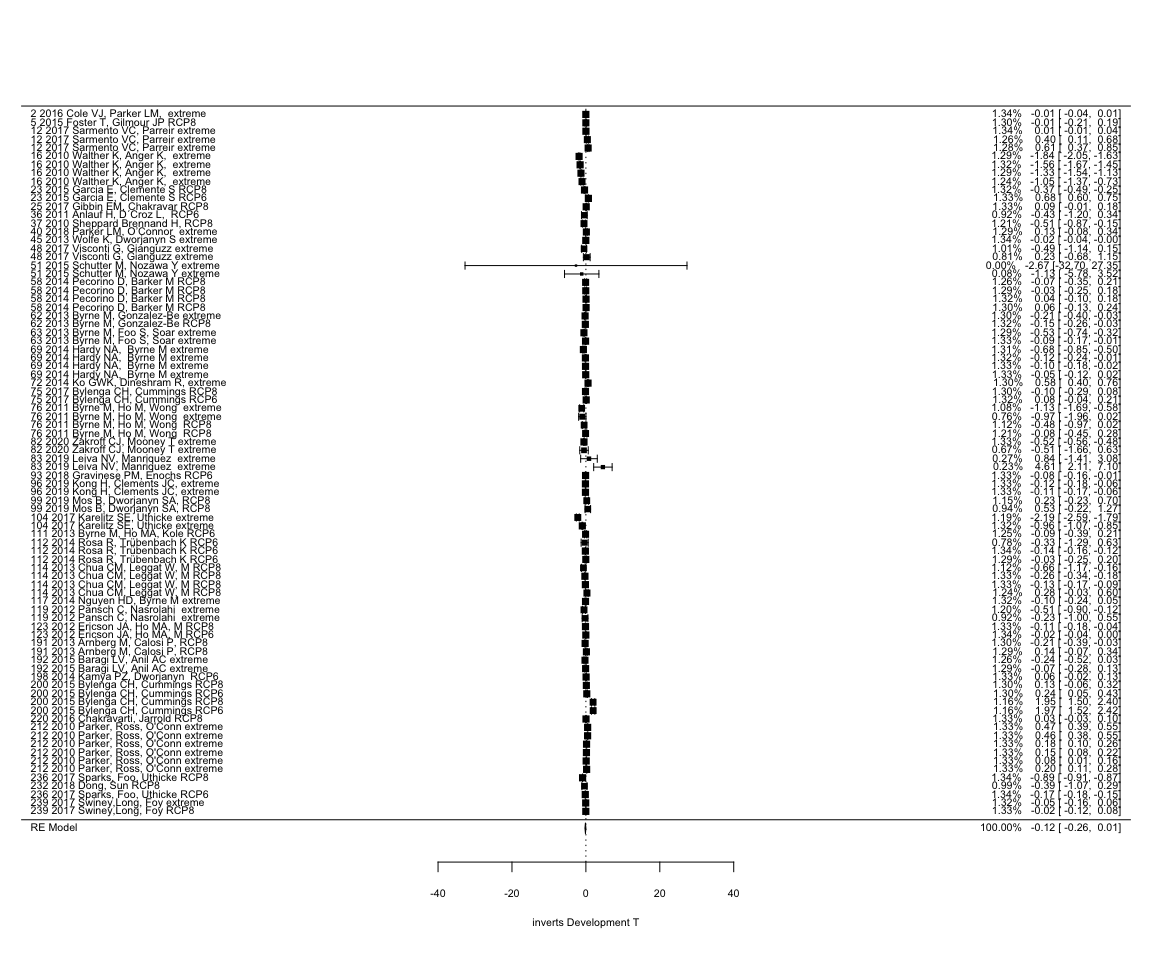


##
## Random-Effects Model (k = 119; tau^2 estimator: REML)
##
## tau^2 (estimated amount of total heterogeneity): 0.2497 (SE = 0.0367)
## tau (square root of estimated tau^2 value): 0.4997
## I^2 (total heterogeneity / total variability): 99.55%
## H^2 (total variability / sampling variability): 222.53
##
## Test for Heterogeneity:
## Q(df = 118) = 13289.4258, p-val < .0001
##
## Model Results:
##
## estimate se zval pval ci.lb ci.ub ​
## -0.1613 0.0494 -3.2615 0.0011 -0.2582 -0.0644 **
##
## ---
## Signif. codes: 0 '***' 0.001 '**' 0.01 '*' 0.05 '.' 0.1 ' ' 1


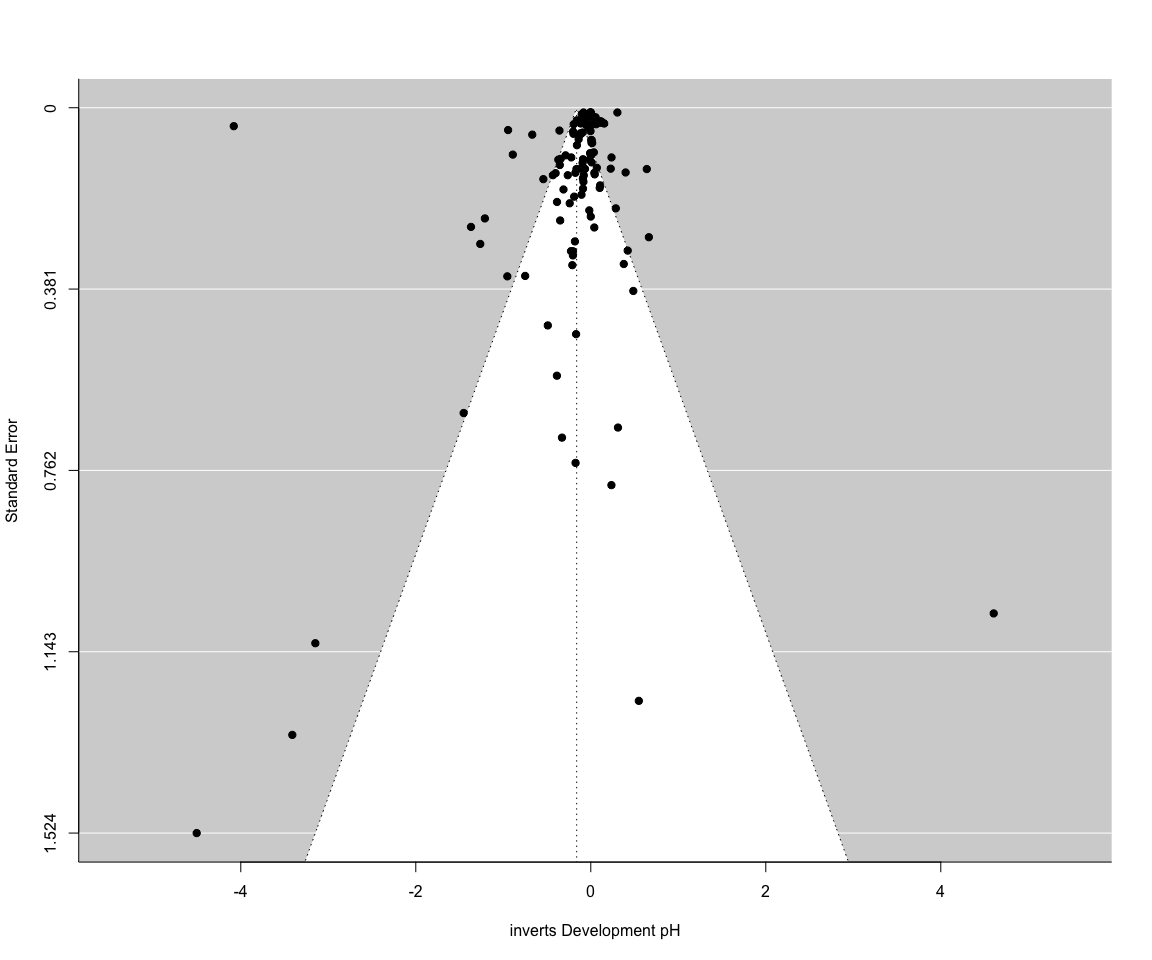


## Warning: Ratio of largest to smallest sampling variance extremely large. May not
## be able to obtain stable results.


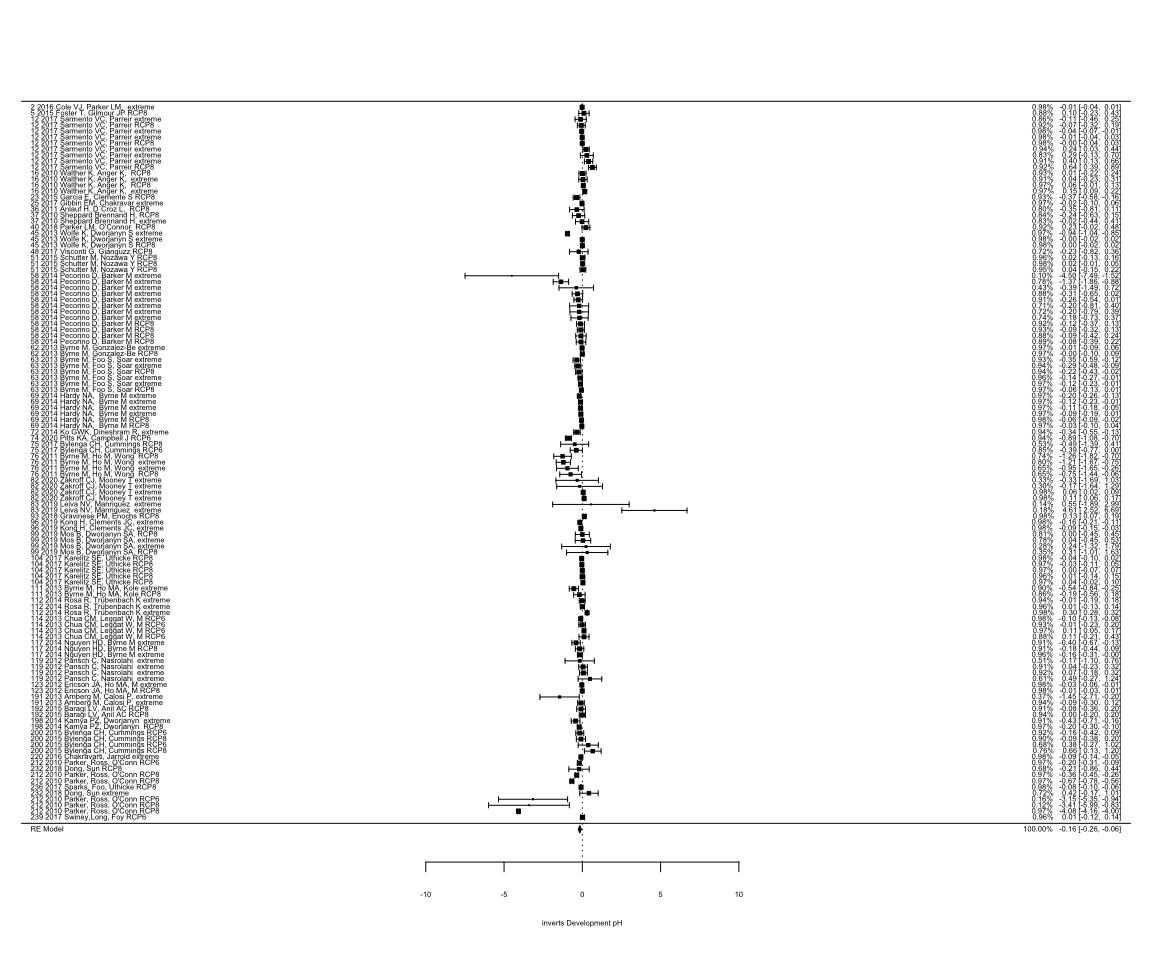


##
## Random-Effects Model (k = 154; tau^2 estimator: REML)
##
## tau^2 (estimated amount of total heterogeneity): 0.4701 (SE = 0.0586)
## tau (square root of estimated tau^2 value): 0.6856
## I^2 (total heterogeneity / total variability): 99.71%
## H^2 (total variability / sampling variability): 349.79
##
## Test for Heterogeneity:
## Q(df = 153) = 12865.5570, p-val < .0001
##
## Model Results:
##
## estimate se zval pval ci.lb ci.ub ​
## -0.2778 0.0584 -4.7564 <.0001 -0.3923 -0.1633 ***
##
## ---
## Signif. codes: 0 '***' 0.001 '**' 0.01 '*' 0.05 '.' 0.1 ' ' 1


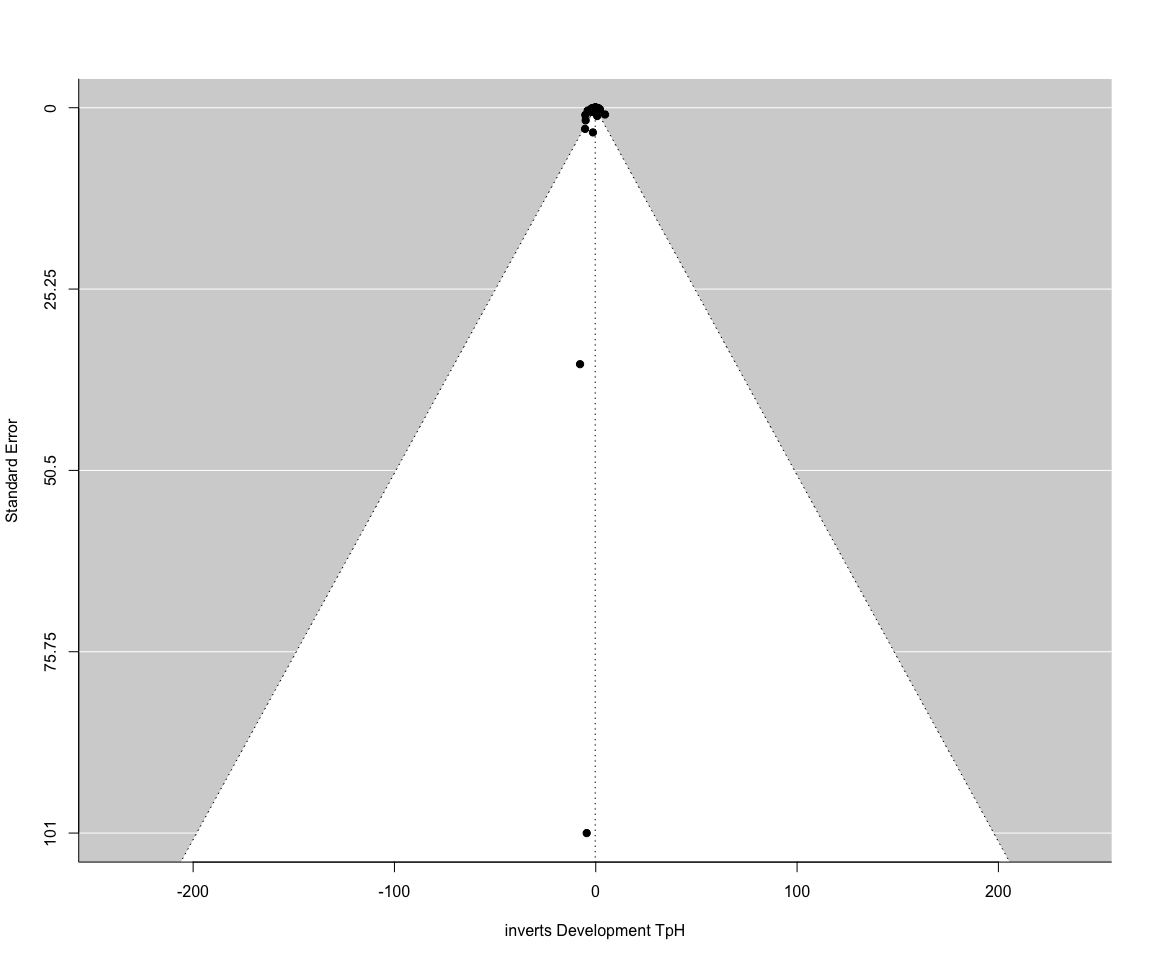


## Warning: Ratio of largest to smallest sampling variance extremely large. May not
## be able to obtain stable results.


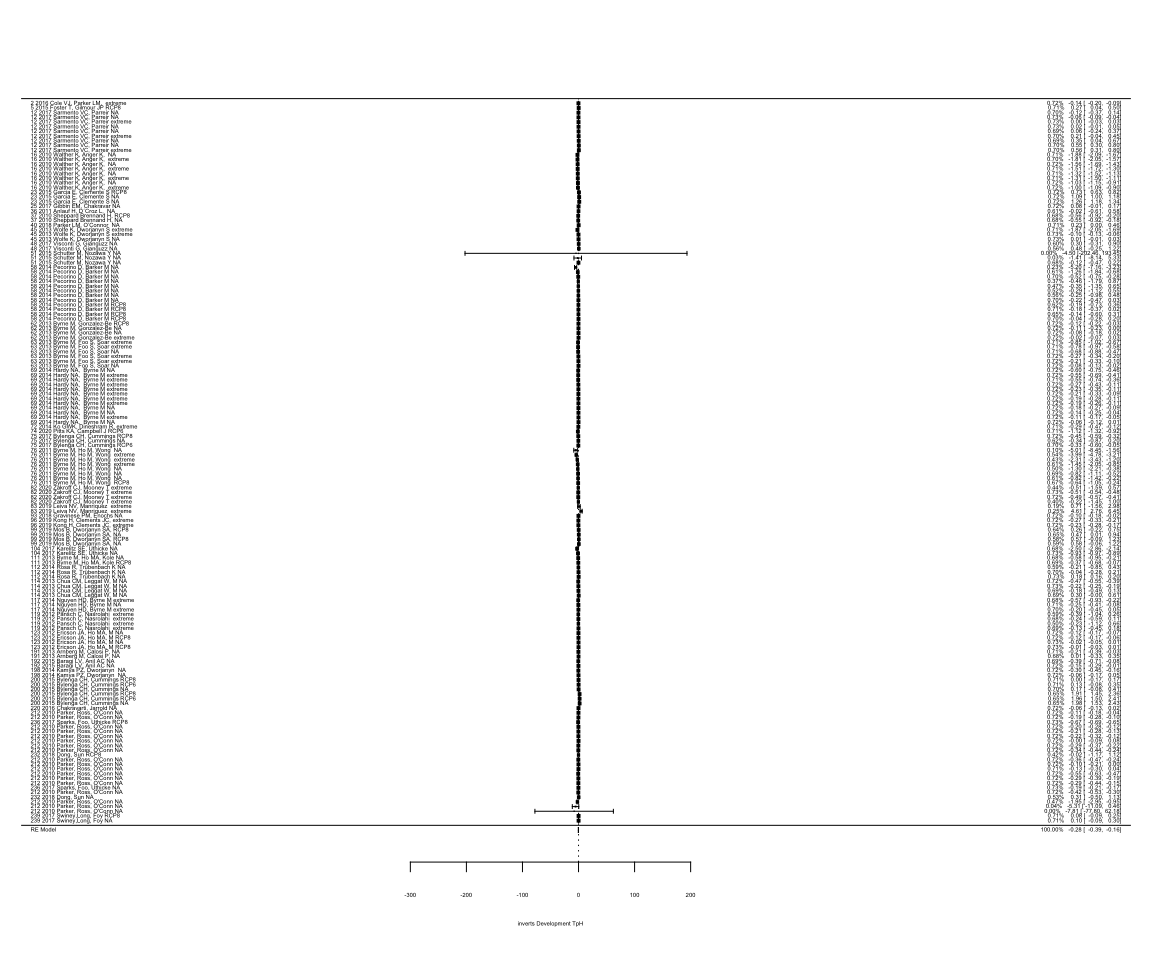


Abs_InvertDev <- MA_TpH_abs("inverts","Development", Inverts)

## Warning: Ratio of largest to smallest sampling variance extremely large. May not be able to obtain stable results.

## Fish, Development
FishDev <- MA_TpH("fish","Development", Fish,sensitivity)

## Random-Effects Model (k = 19; tau^2 estimator: REML)
##
## tau^2 (estimated amount of total heterogeneity): 0.1014 (SE = 0.0348)
## tau (square root of estimated tau^2 value): 0.3185
## I^2 (total heterogeneity / total variability): 99.57%
## H^2 (total variability / sampling variability): 232.54
##
## Test for Heterogeneity:
## Q(df = 18) = 2632.5175, p-val < .0001
##
## Model Results:
##
## estimate se zval pval ci.lb ci.ub ​
## -0.2365 0.0742 -3.1876 0.0014 -0.3819 -0.0911 **
##
## ---
## Signif. codes: 0 '***' 0.001 '**' 0.01 '*' 0.05 '.' 0.1 ' ' 1


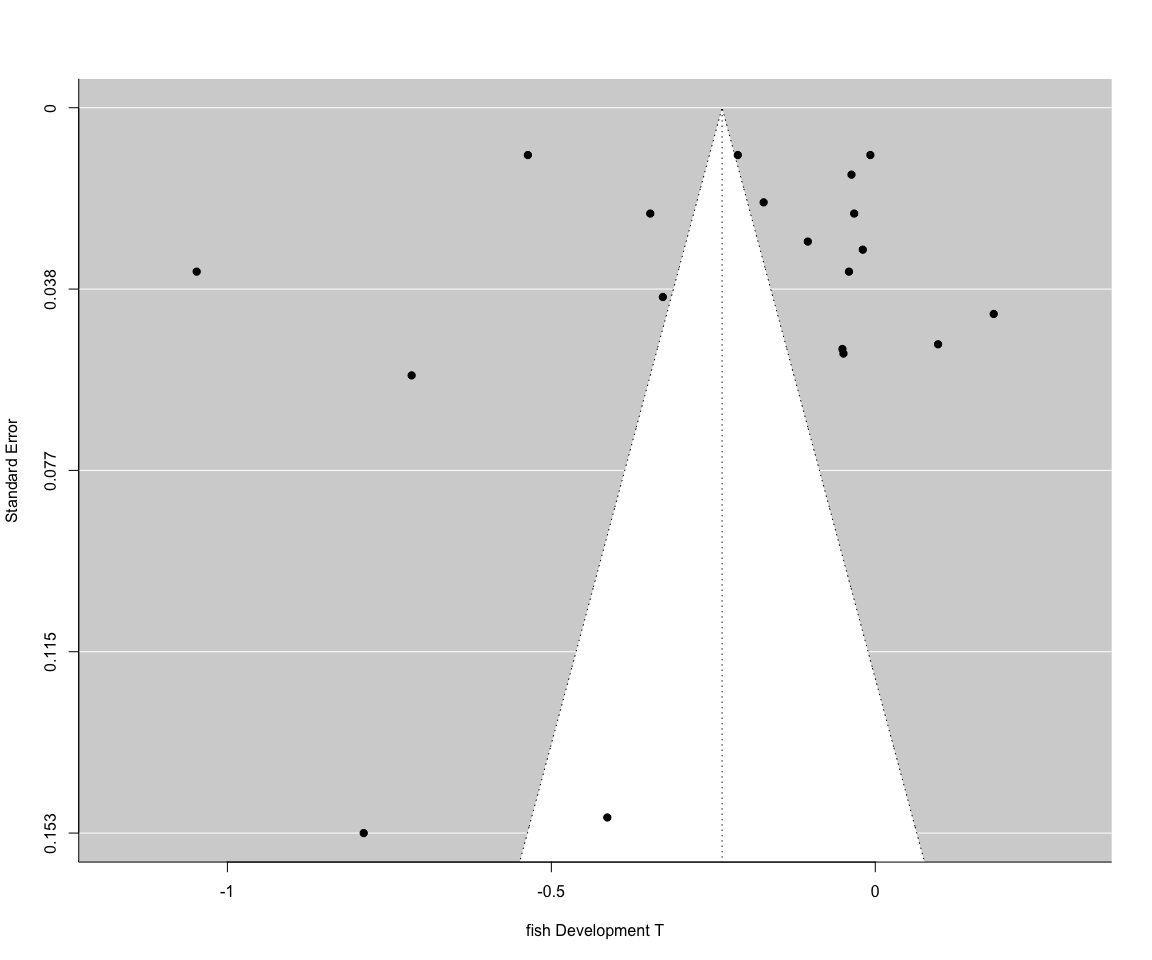

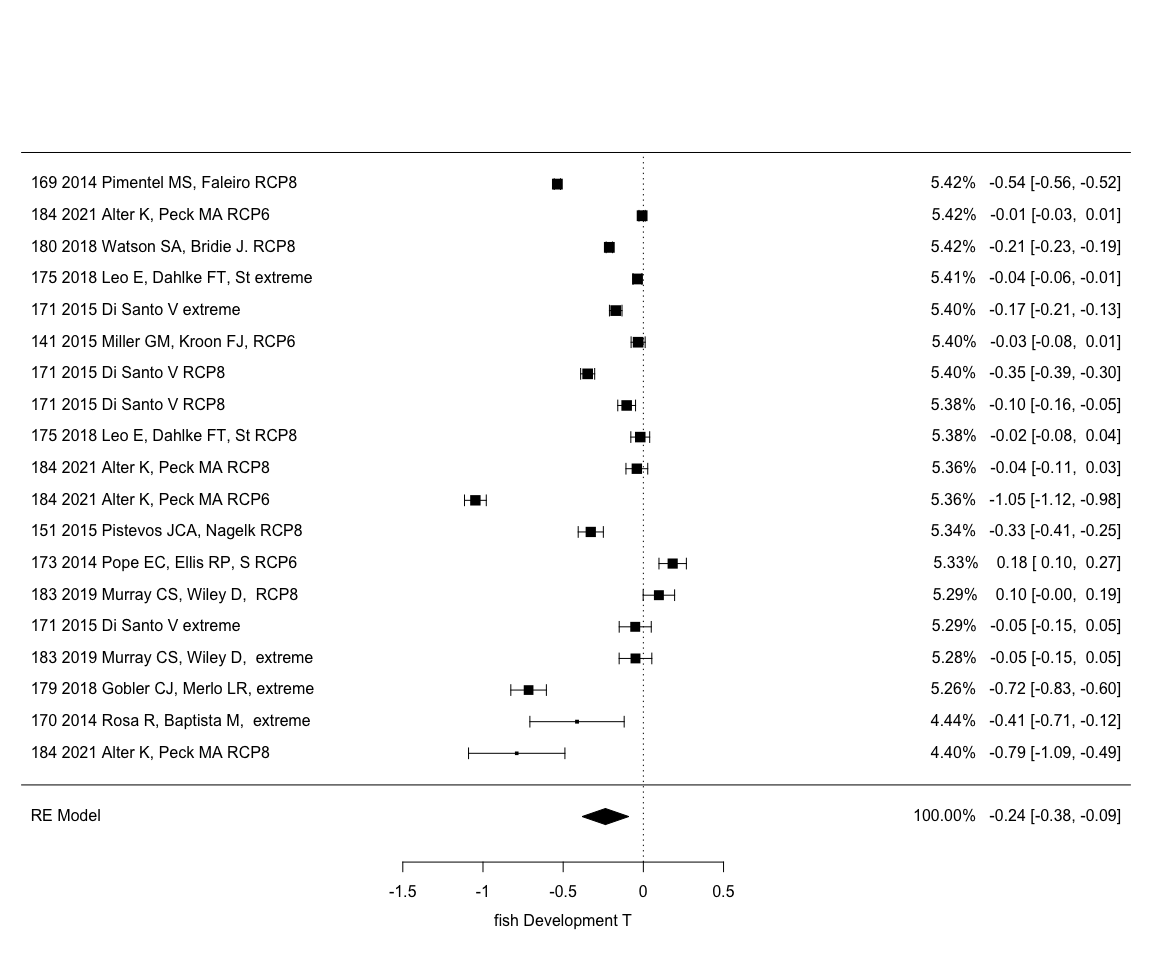


##
## Random-Effects Model (k = 15; tau^2 estimator: REML)
##
## tau^2 (estimated amount of total heterogeneity): 0.0657 (SE = 0.0260)
## tau (square root of estimated tau^2 value): 0.2564
## I^2 (total heterogeneity / total variability): 99.34%
## H^2 (total variability / sampling variability): 151.57
##
## Test for Heterogeneity:
## Q(df = 14) = 4814.2243, p-val < .0001
##
## Model Results:
##
## estimate se zval pval ci.lb ci.ub ​
## -0.0712 0.0677 -1.0515 0.2930 -0.2040 0.0615
##
## ---
## Signif. codes: 0 '***' 0.001 '**' 0.01 '*' 0.05 '.' 0.1 ' ' 1


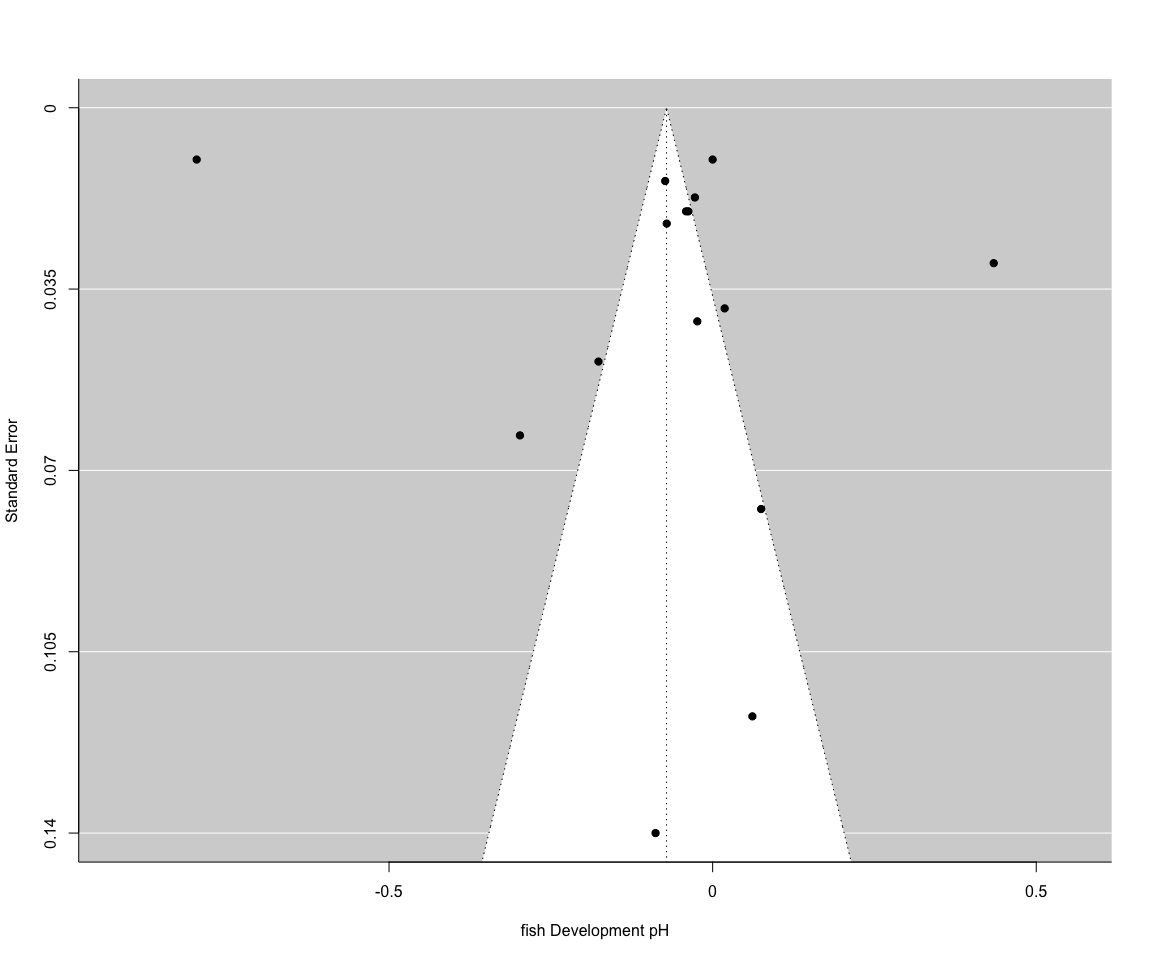

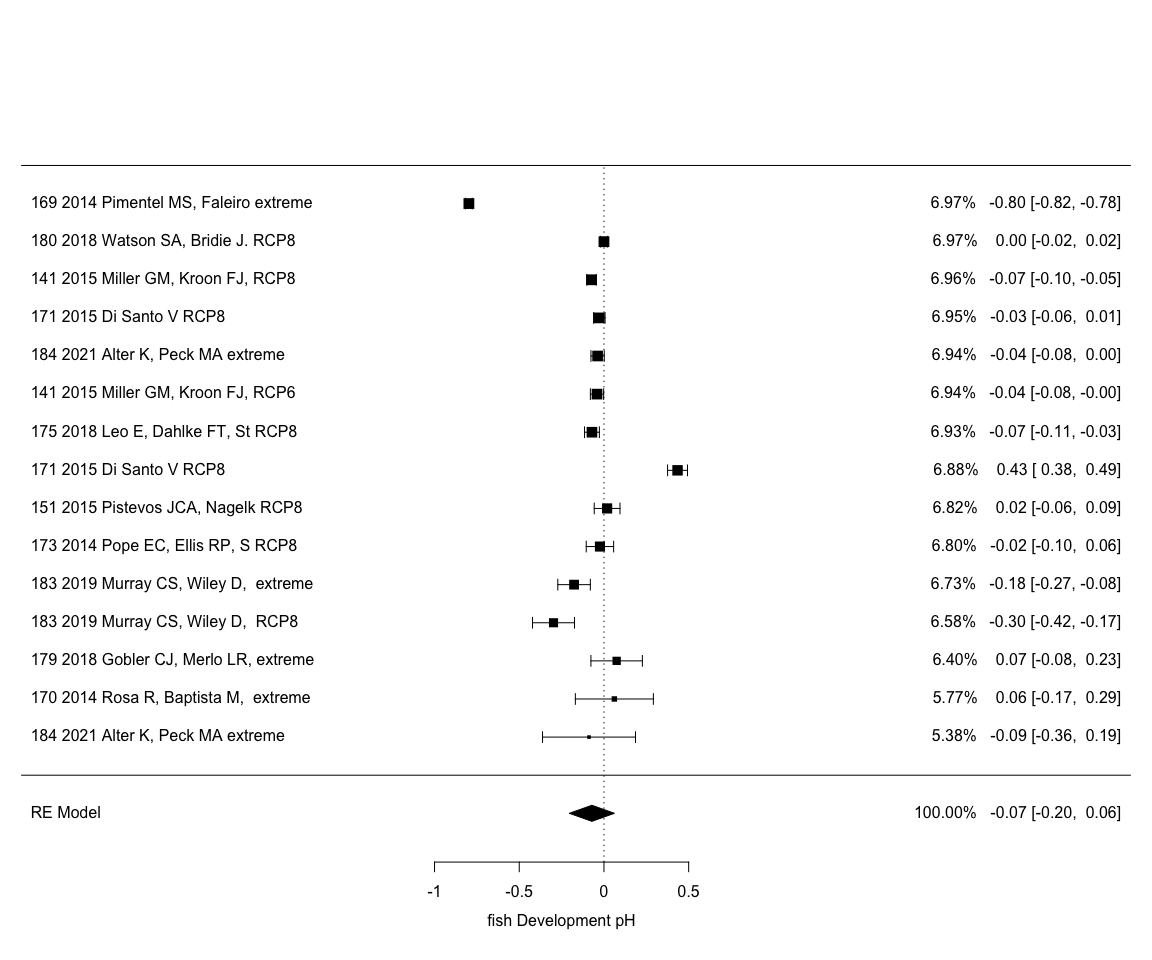


##
## Random-Effects Model (k = 21; tau^2 estimator: REML)
##
## tau^2 (estimated amount of total heterogeneity): 0.1722 (SE = 0.0557)
## tau (square root of estimated tau^2 value): 0.4150
## I^2 (total heterogeneity / total variability): 99.62%
## H^2 (total variability / sampling variability): 261.20
##
## Test for Heterogeneity:
## Q(df = 20) = 12571.3841, p-val < .0001
##
## Model Results:
##
## estimate se zval pval ci.lb ci.ub ​
## -0.3266 0.0916 -3.5658 0.0004 -0.5061 -0.1471 ***
##
## ---
## Signif. codes: 0 '***' 0.001 '**' 0.01 '*' 0.05 '.' 0.1 ' ' 1


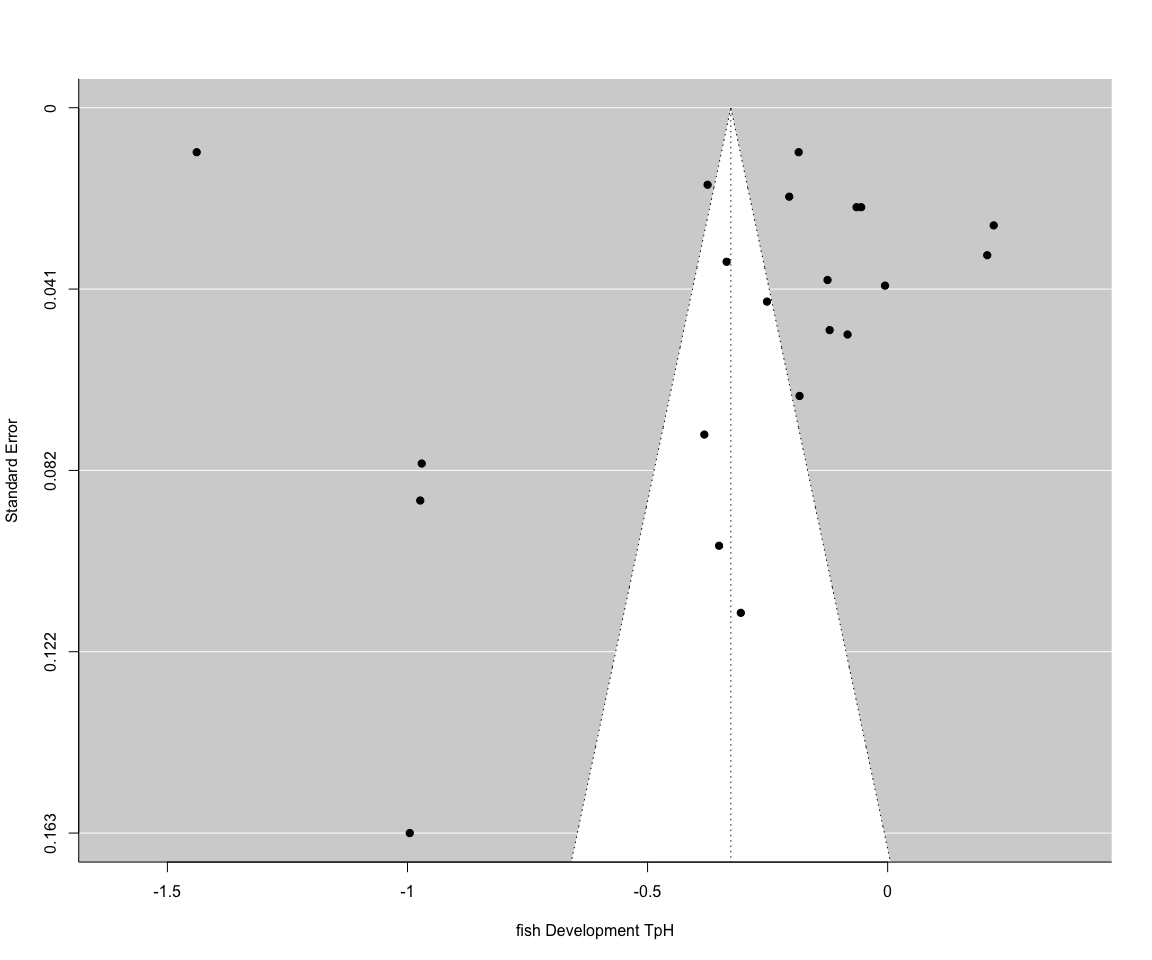

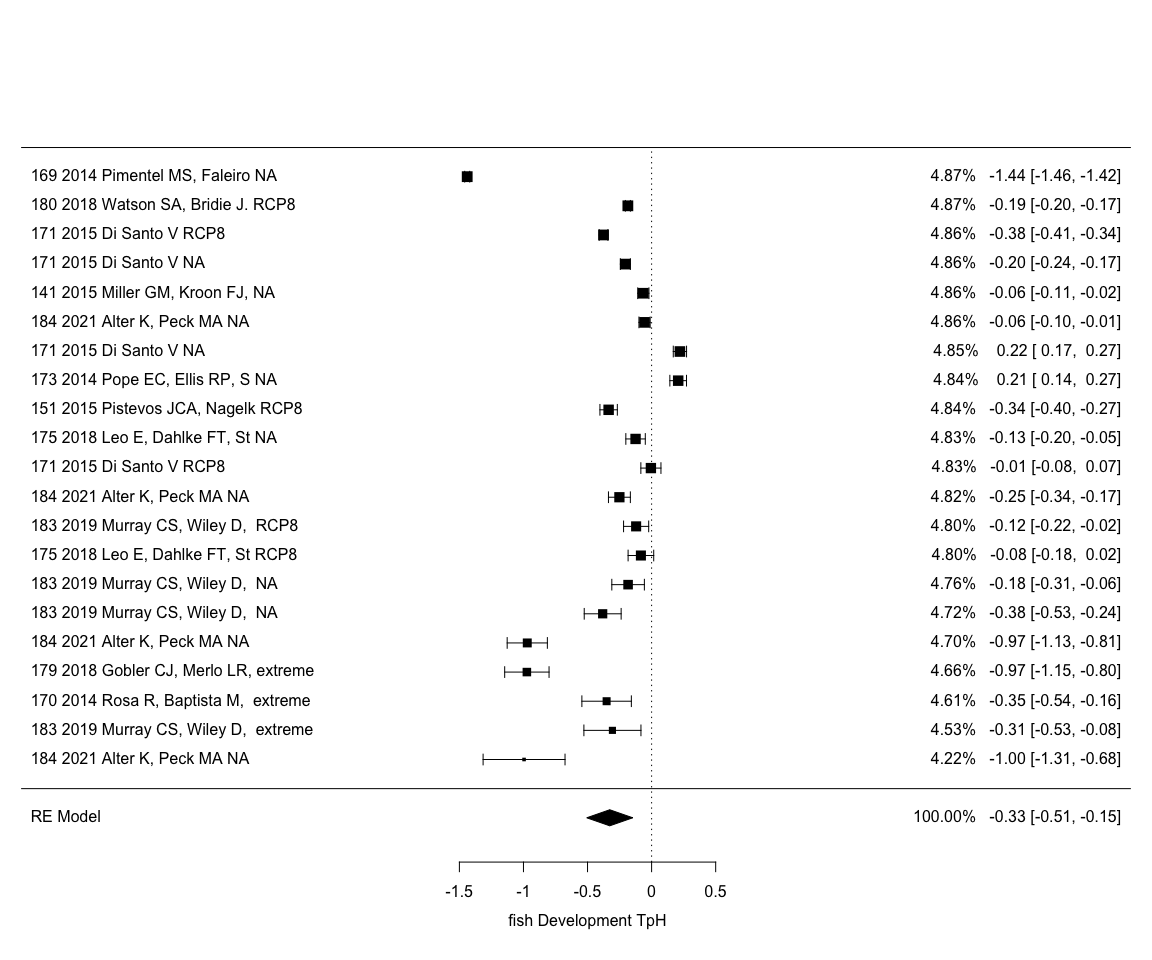


Abs_FishDev <- MA_TpH_abs("fish","Development", Fish)

## Invertebrate, Growth
InvertGrowth <- MA_TpH("inverts","Growth", Inverts,sensitivity)

##
## Random-Effects Model (k = 118; tau^2 estimator: REML)
##
## tau^2 (estimated amount of total heterogeneity): 0.1780 (SE = 0.0257)
## tau (square root of estimated tau^2 value): 0.4219
## I^2 (total heterogeneity / total variability): 99.43%
## H^2 (total variability / sampling variability): 174.35
##
## Test for Heterogeneity:
## Q(df = 117) = 2507.0291, p-val < .0001
##
## Model Results:
##
## estimate se zval pval ci.lb ci.ub ​
## 0.0545 0.0411 1.3266 0.1846 -0.0260 0.1351
##
## ---
## Signif. codes: 0 '***' 0.001 '**' 0.01 '*' 0.05 '.' 0.1 ' ' 1


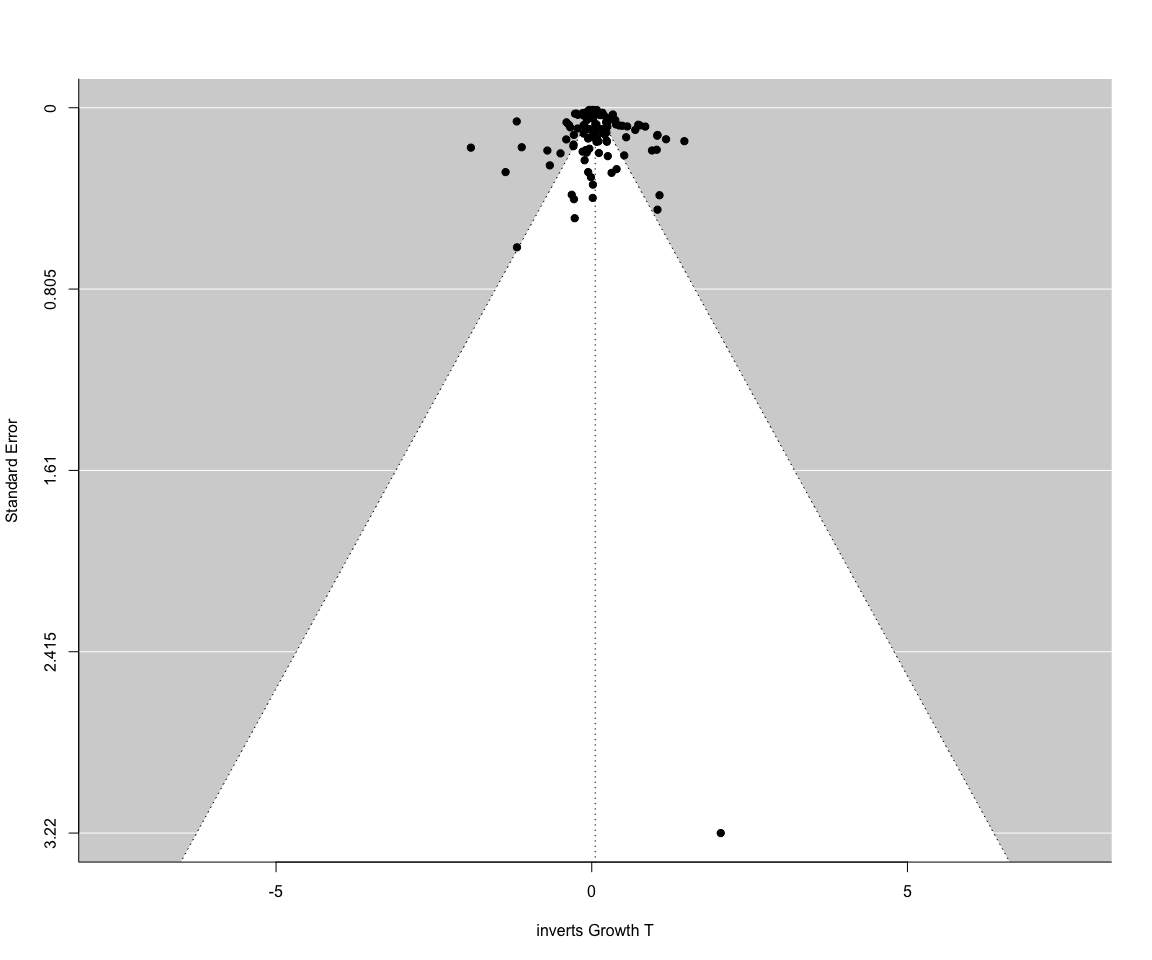

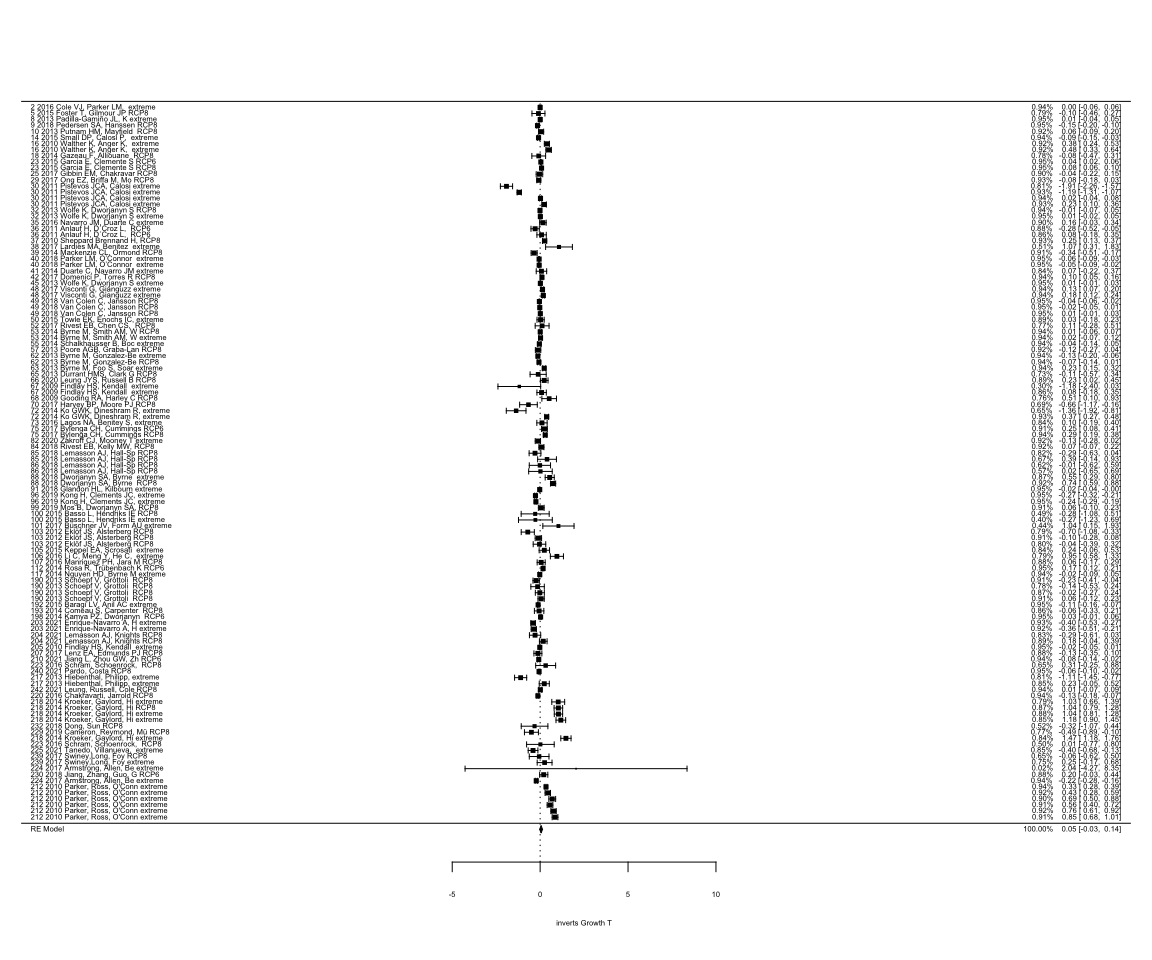


##
## Random-Effects Model (k = 147; tau^2 estimator: REML)
##
## tau^2 (estimated amount of total heterogeneity): 0.0627 (SE = 0.0088)
## tau (square root of estimated tau^2 value): 0.2503
## I^2 (total heterogeneity / total variability): 98.71%
## H^2 (total variability / sampling variability): 77.26
##
## Test for Heterogeneity:
## Q(df = 146) = 2878.2198, p-val < .0001
##
## Model Results:
##
## estimate se zval pval ci.lb ci.ub ​
## -0.1124 0.0231 -4.8717 <.0001 -0.1576 -0.0672 ***
##
## ---
## Signif. codes: 0 '***' 0.001 '**' 0.01 '*' 0.05 '.' 0.1 ' ' 1


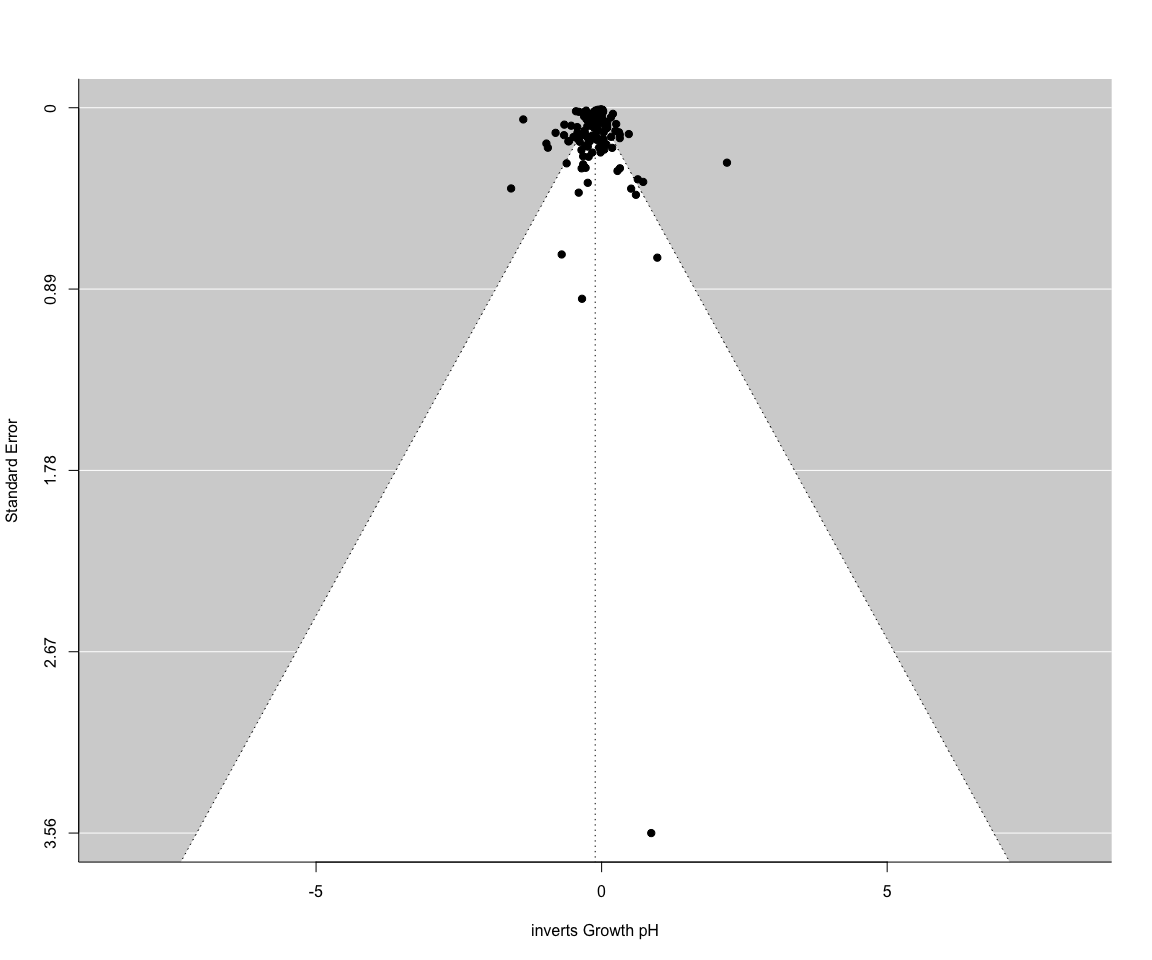


## Warning: Ratio of largest to smallest sampling variance extremely large. May not
## be able to obtain stable results.


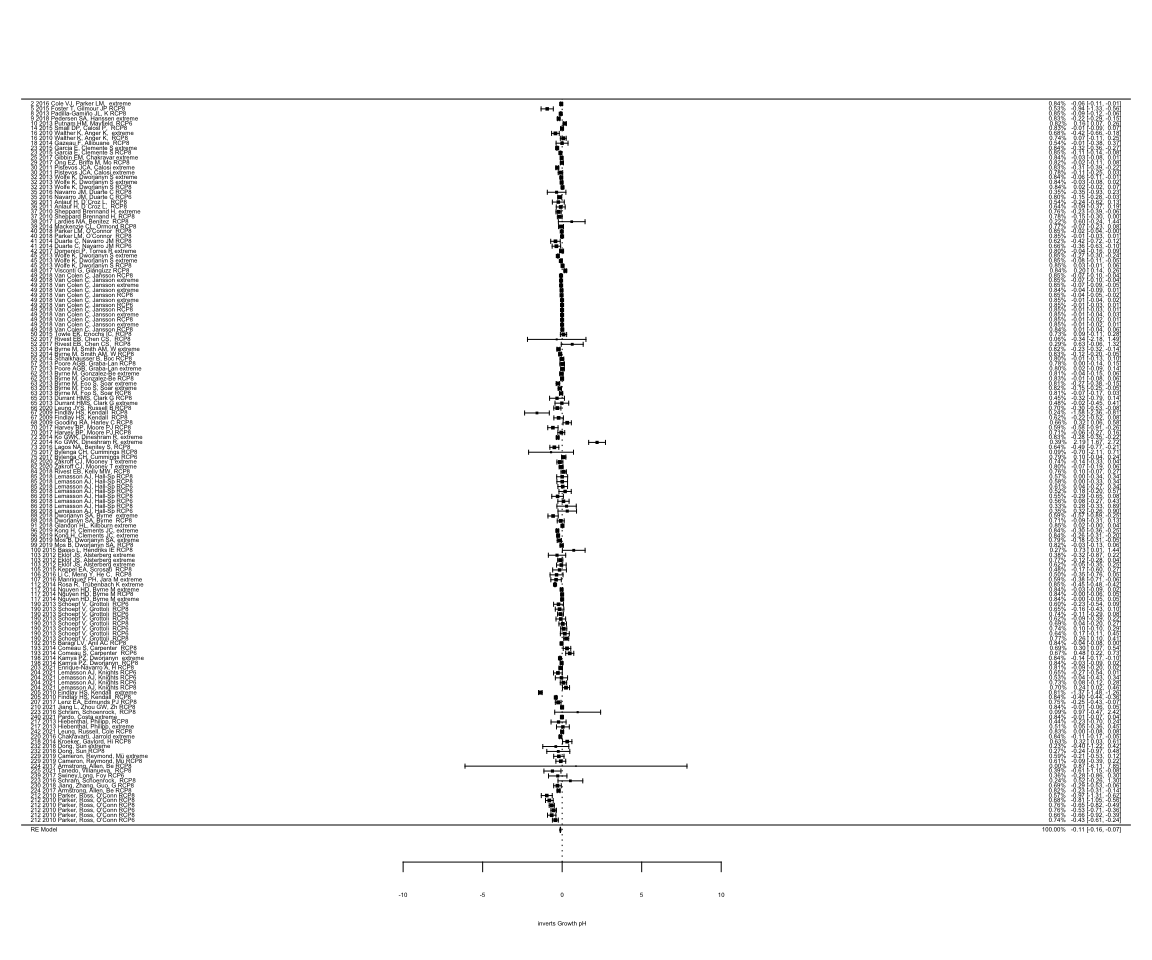


##
## Random-Effects Model (k = 190; tau^2 estimator: REML)
##
## tau^2 (estimated amount of total heterogeneity): 0.1765 (SE = 0.0200)
## tau (square root of estimated tau^2 value): 0.4201
## I^2 (total heterogeneity / total variability): 99.41%
## H^2 (total variability / sampling variability): 170.32
##
## Test for Heterogeneity:
## Q(df = 189) = 5880.2854, p-val < .0001
##
## Model Results:
##
## estimate se zval pval ci.lb ci.ub ​
## -0.1080 0.0323 -3.3403 0.0008 -0.1714 -0.0446 ***
##
## ---
## Signif. codes: 0 '***' 0.001 '**' 0.01 '*' 0.05 '.' 0.1 ' ' 1


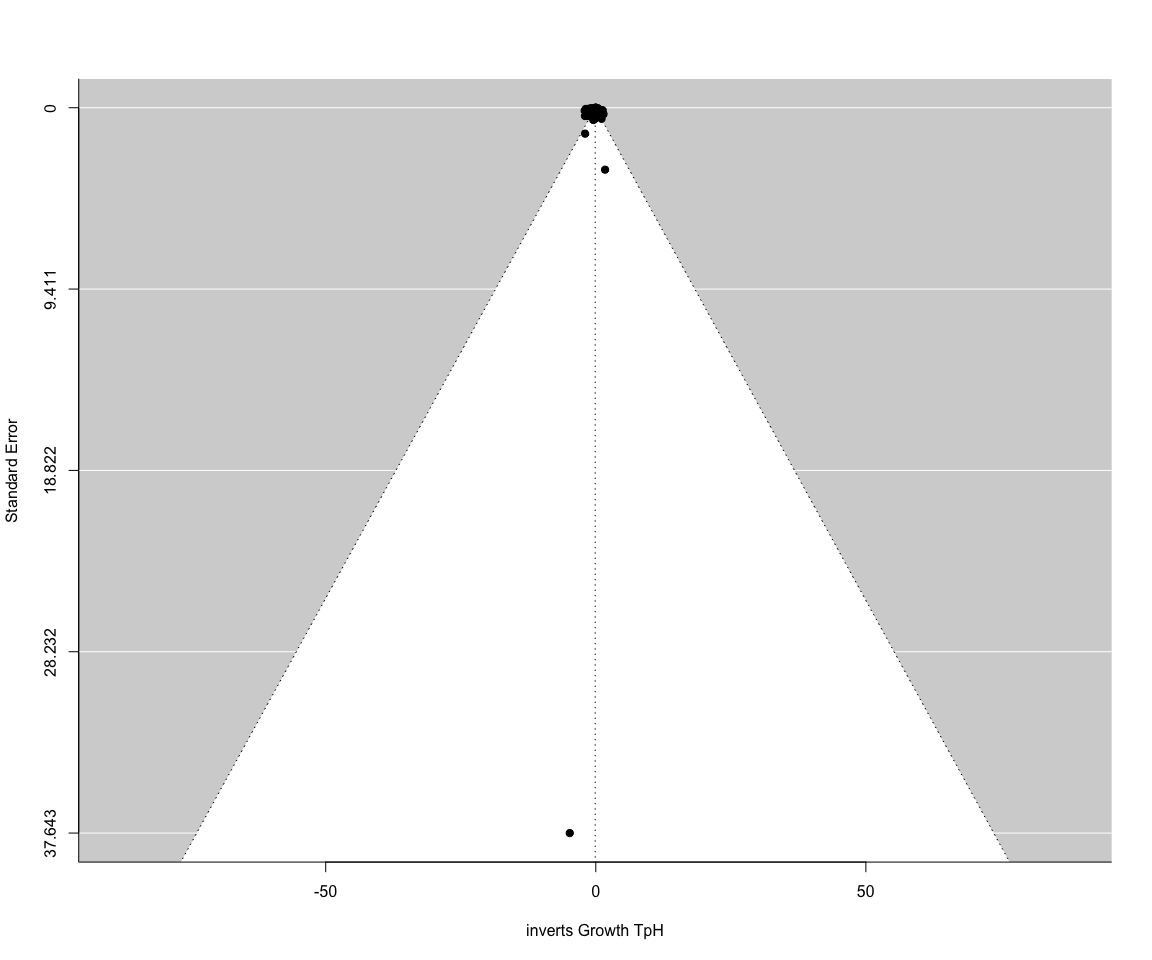


## Warning: Ratio of largest to smallest sampling variance extremely large. May not
## be able to obtain stable results.


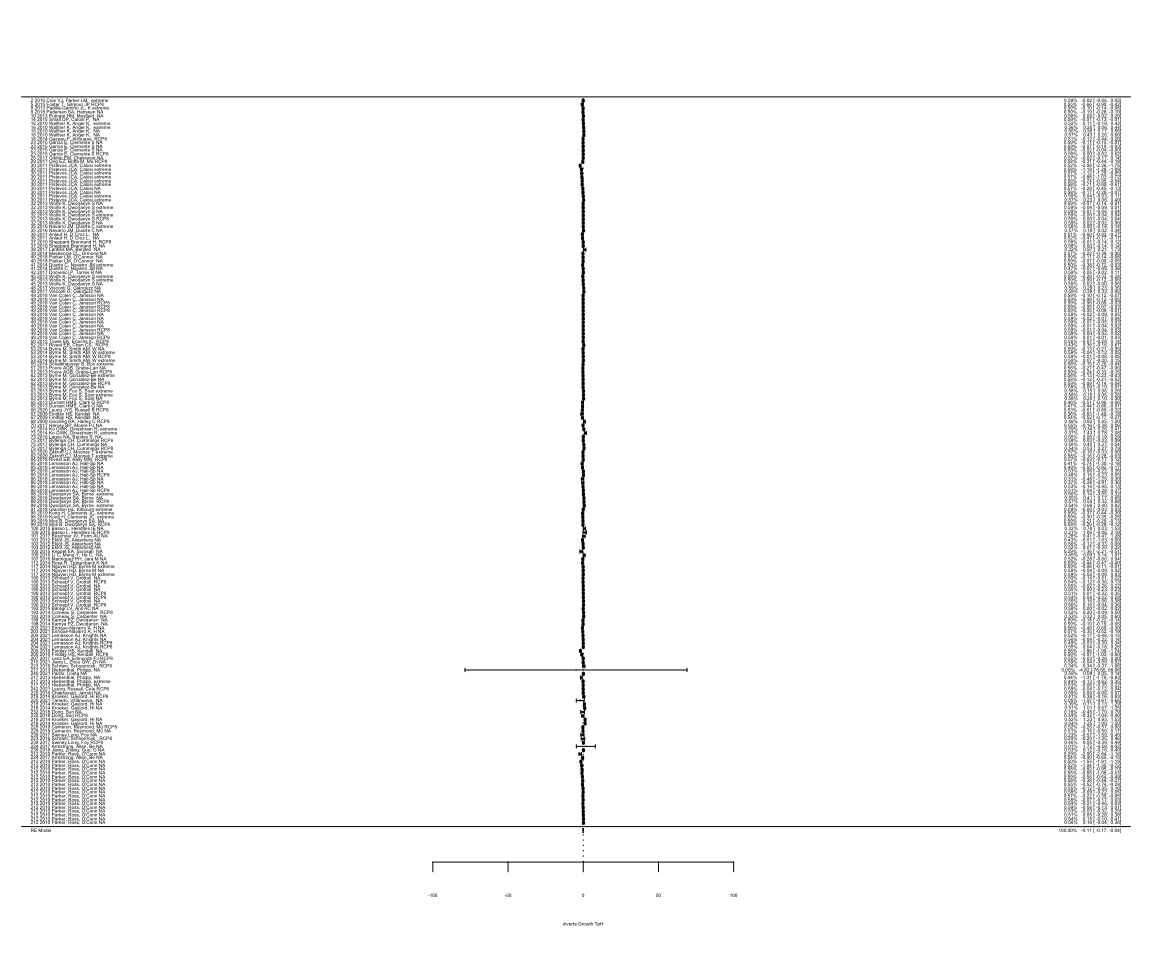


Abs_InvertGrowth <- MA_TpH_abs("inverts","Growth", Inverts)

## Warning: Ratio of largest to smallest sampling variance extremely large. May not be able to obtain stable results.

##Fish, Growth
FishGrowth <- MA_TpH("fish","Growth", Fish,sensitivity)

## Warning: Fisher scoring algorithm may have gotten stuck at a local maximum.
## Setting tau^2 = 0. Check the profile likelihood plot with profile().

## Random-Effects Model (k = 64; tau^2 estimator: REML)
##
## tau^2 (estimated amount of total heterogeneity): 0.0863 (SE = 0.0172)
## tau (square root of estimated tau^2 value): 0.2938
## I^2 (total heterogeneity / total variability): 99.39%
## H^2 (total variability / sampling variability): 164.04
##
## Test for Heterogeneity:
## Q(df = 63) = 3256.7406, p-val < .0001
##
## Model Results:
## ## estimate se zval pval ci.lb ci.ub ​
## 0.0717 0.0392 1.8283 0.0675 -0.0052 0.1486 .
## ---
## Signif. codes: 0 '***' 0.001 '**' 0.01 '*' 0.05 '.' 0.1 ' ' 1


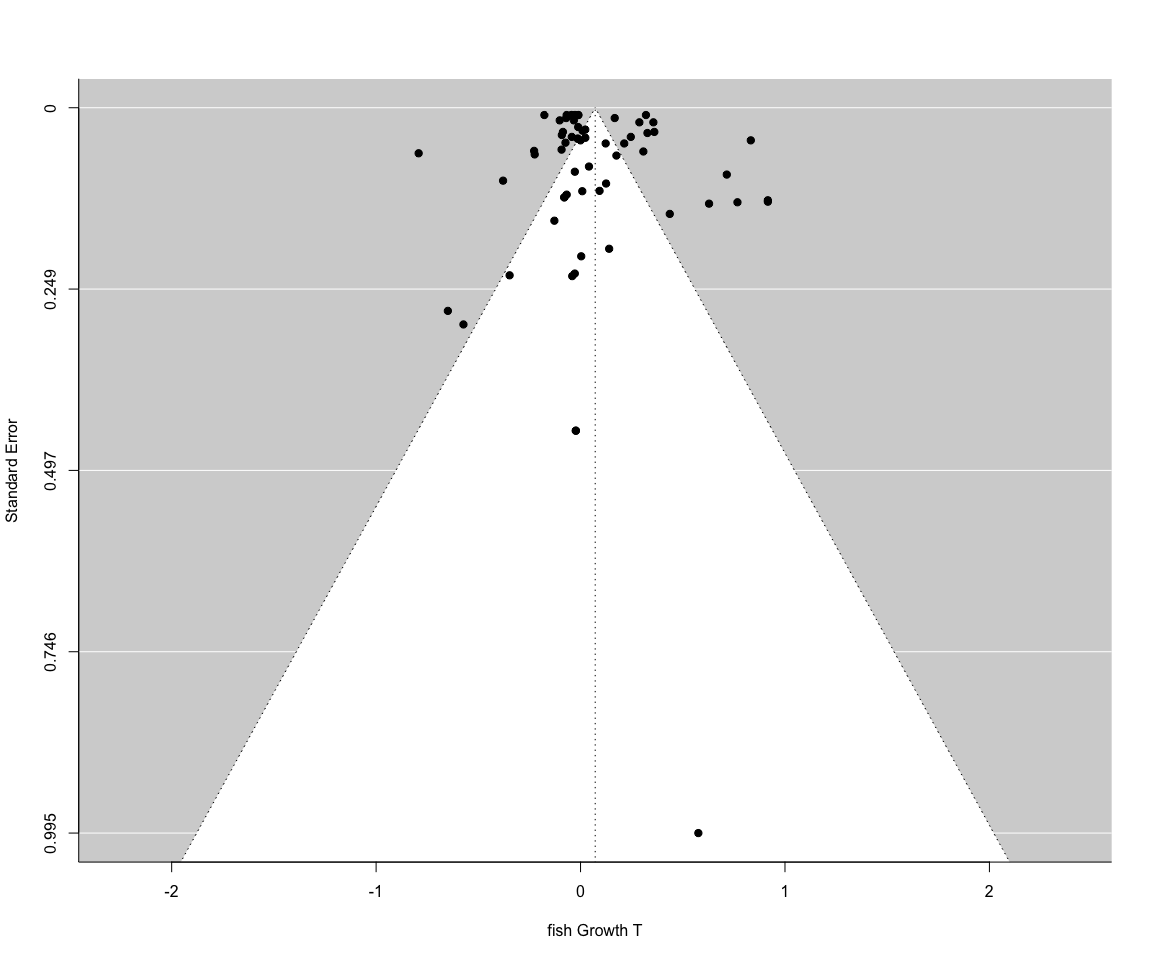

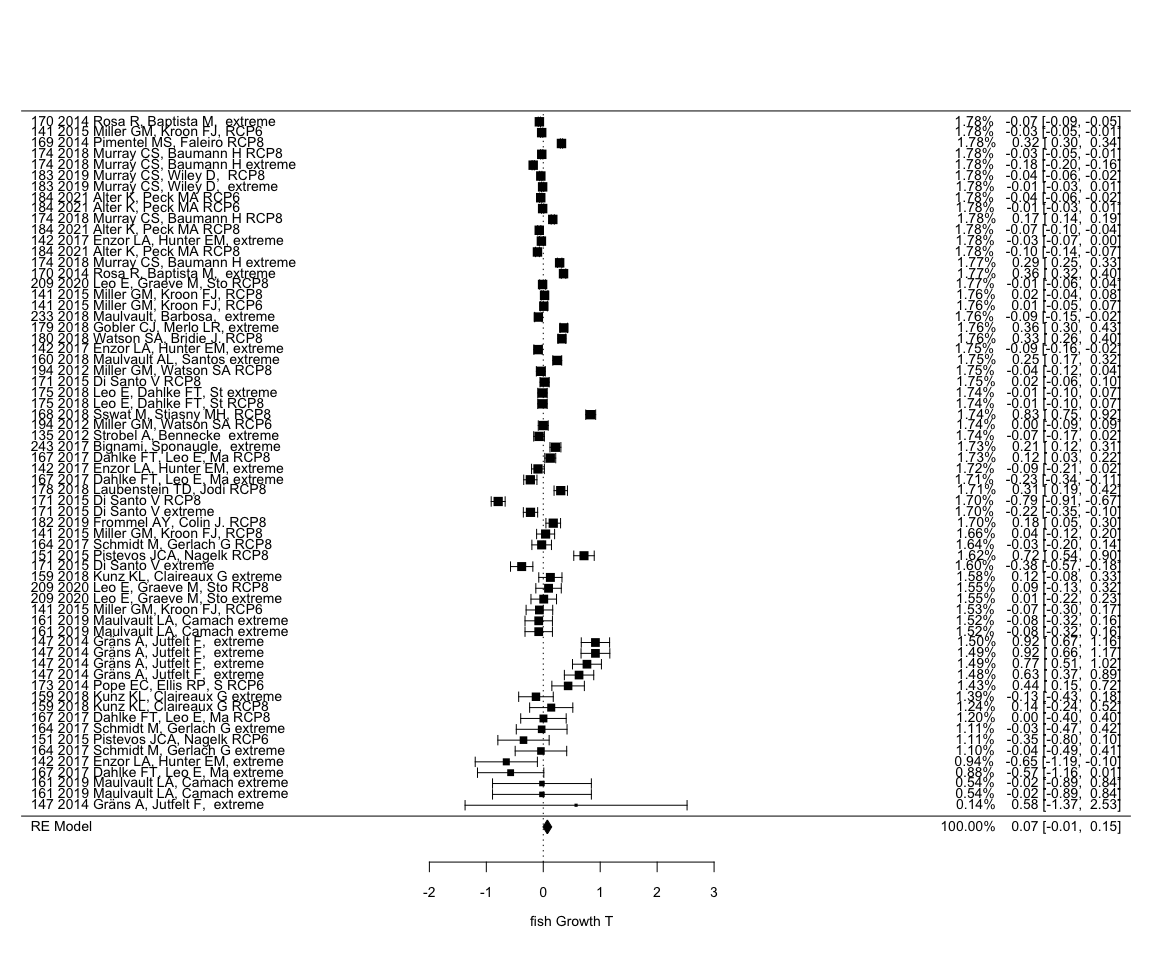


##
## Random-Effects Model (k = 50; tau^2 estimator: REML)
##
## tau^2 (estimated amount of total heterogeneity): 0.0158 (SE = 0.0041)
## tau (square root of estimated tau^2 value): 0.1258
## I^2 (total heterogeneity / total variability): 97.59%
## H^2 (total variability / sampling variability): 41.56
##
## Test for Heterogeneity:
## Q(df = 49) = 1125.5233, p-val < .0001
##
## Model Results:
##
## estimate se zval pval ci.lb ci.ub ​
## -0.0508 0.0210 -2.4214 0.0155 -0.0919 -0.0097 *
##
## ---
## Signif. codes: 0 '***' 0.001 '**' 0.01 '*' 0.05 '.' 0.1 ' ' 1


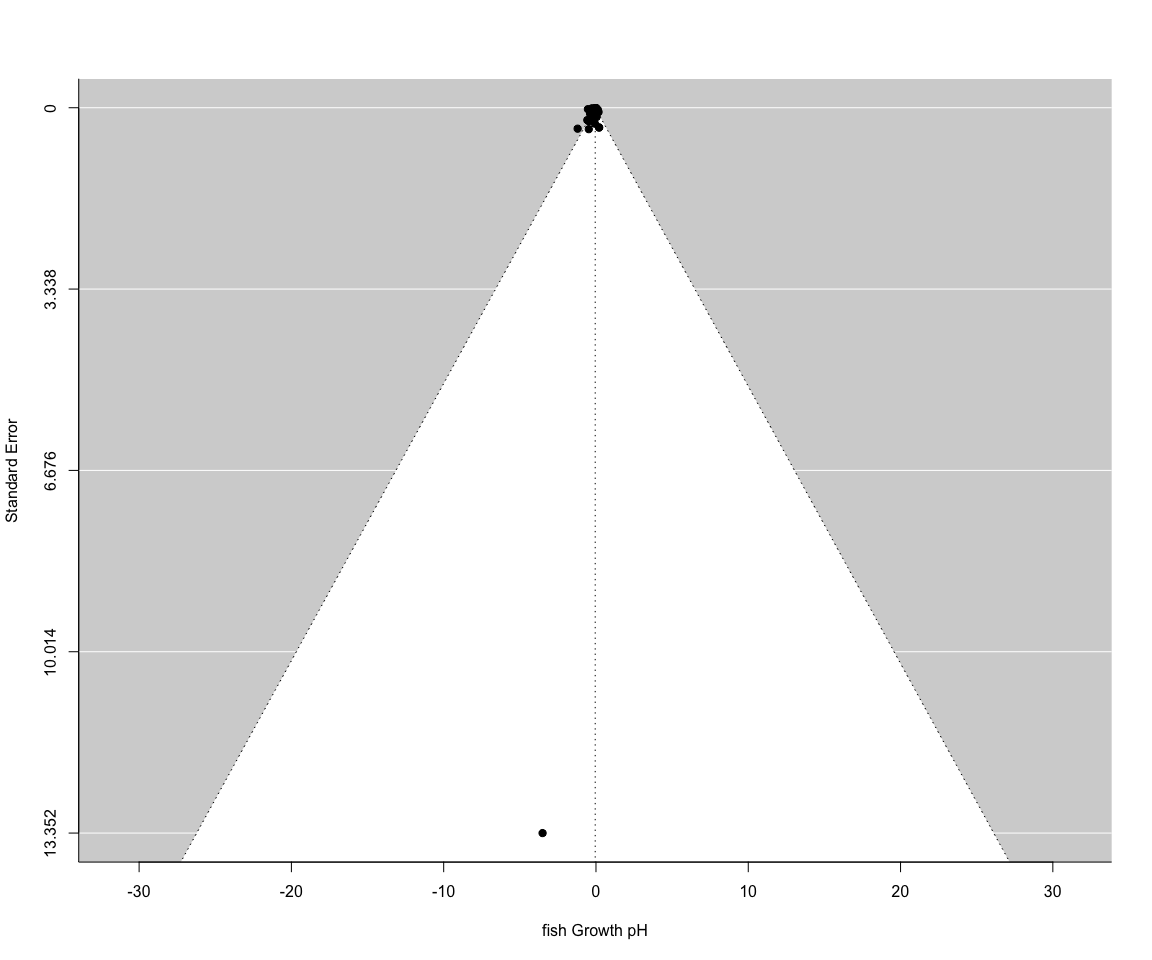

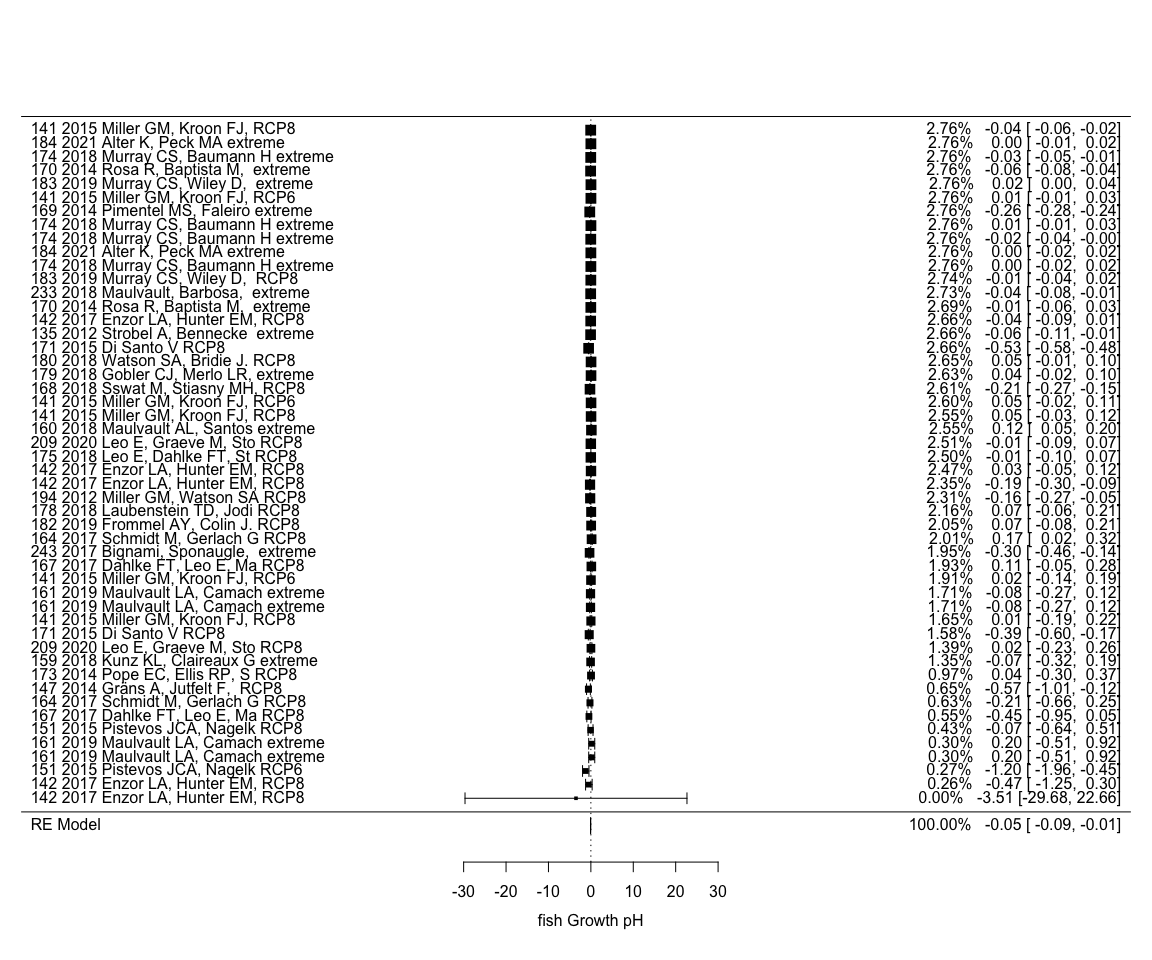


##
## Random-Effects Model (k = 74; tau^2 estimator: REML)
##
## tau^2 (estimated amount of total heterogeneity): 0.0915 (SE = 0.0167)
## tau (square root of estimated tau^2 value): 0.3026
## I^2 (total heterogeneity / total variability): 99.45%
## H^2 (total variability / sampling variability): 180.82
##
## Test for Heterogeneity:
## Q(df = 73) = 3449.3852, p-val < .0001
##
## Model Results:
##
## estimate se zval pval ci.lb ci.ub ​
## 0.0403 0.0373 1.0784 0.2808 -0.0329 0.1134
##
## ---
## Signif. codes: 0 '***' 0.001 '**' 0.01 '*' 0.05 '.' 0.1 ' ' 1


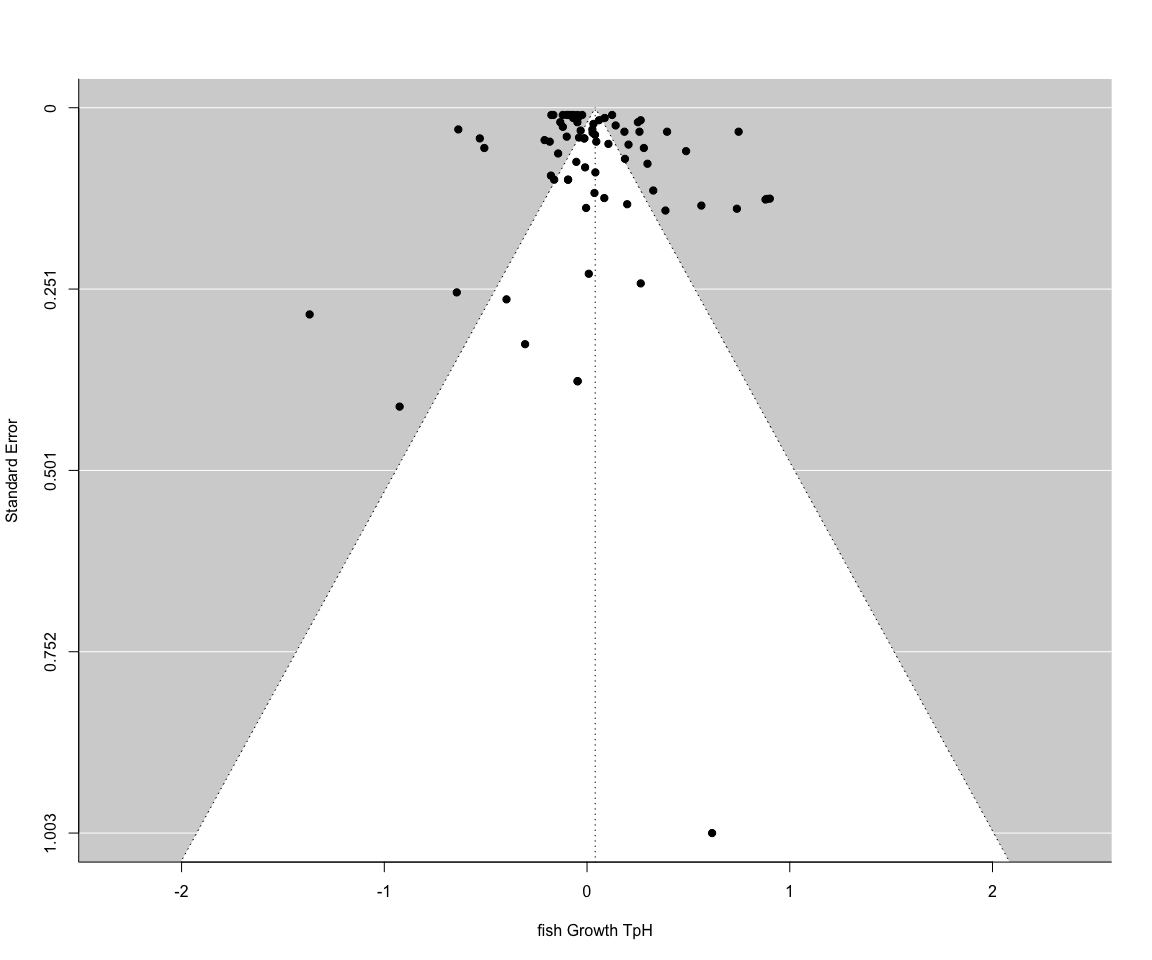

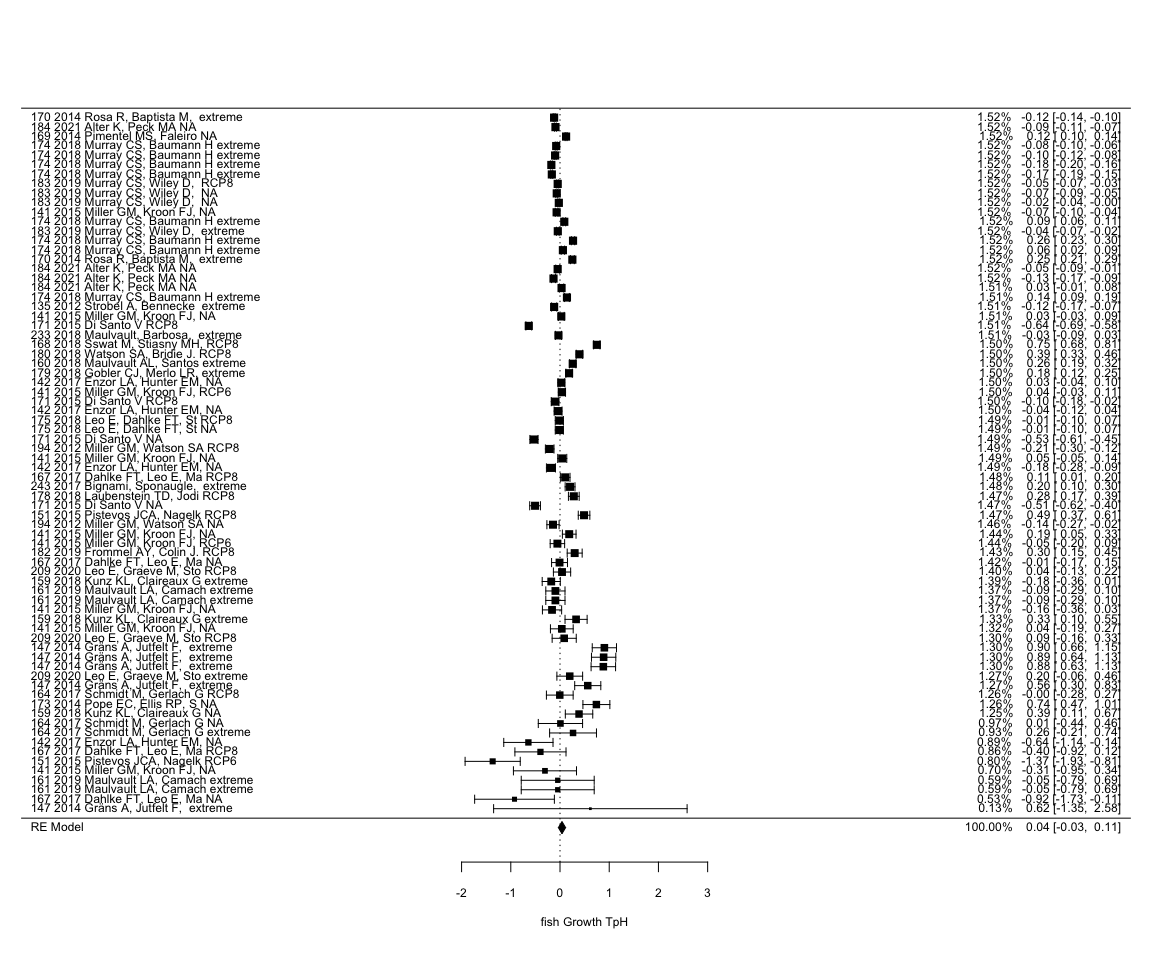


Abs_FishGrowth <- MA_TpH_abs("fish","Growth", Fish)

## Invertebrate, Physiology
InvertPhys <- MA_TpH("inverts","Physiology", Inverts,sensitivity)

## Random-Effects Model (k = 85; tau^2 estimator: REML)
##
## tau^2 (estimated amount of total heterogeneity): 0.2657 (SE = 0.0456)
## tau (square root of estimated tau^2 value): 0.5154
## I^2 (total heterogeneity / total variability): 99.69%
## H^2 (total variability / sampling variability): 317.79
##
## Test for Heterogeneity:
## Q(df = 84) = 2371.5509, p-val < .0001
##
## Model Results:
##
## estimate se zval pval ci.lb ci.ub ​
## -0.0862 0.0595 -1.4482 0.1476 -0.2028 0.0305
##
## ---
## Signif. codes: 0 '***' 0.001 '**' 0.01 '*' 0.05 '.' 0.1 ' ' 1


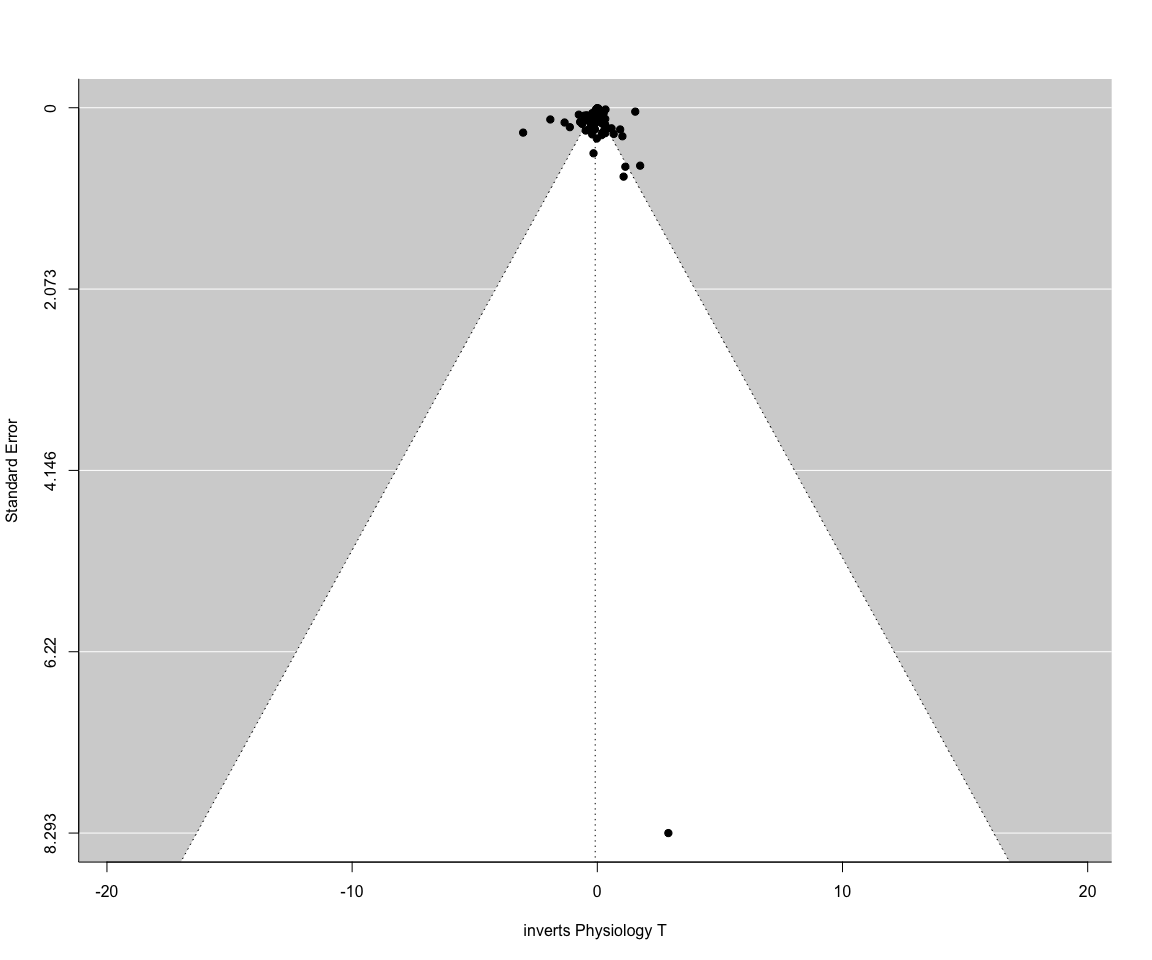

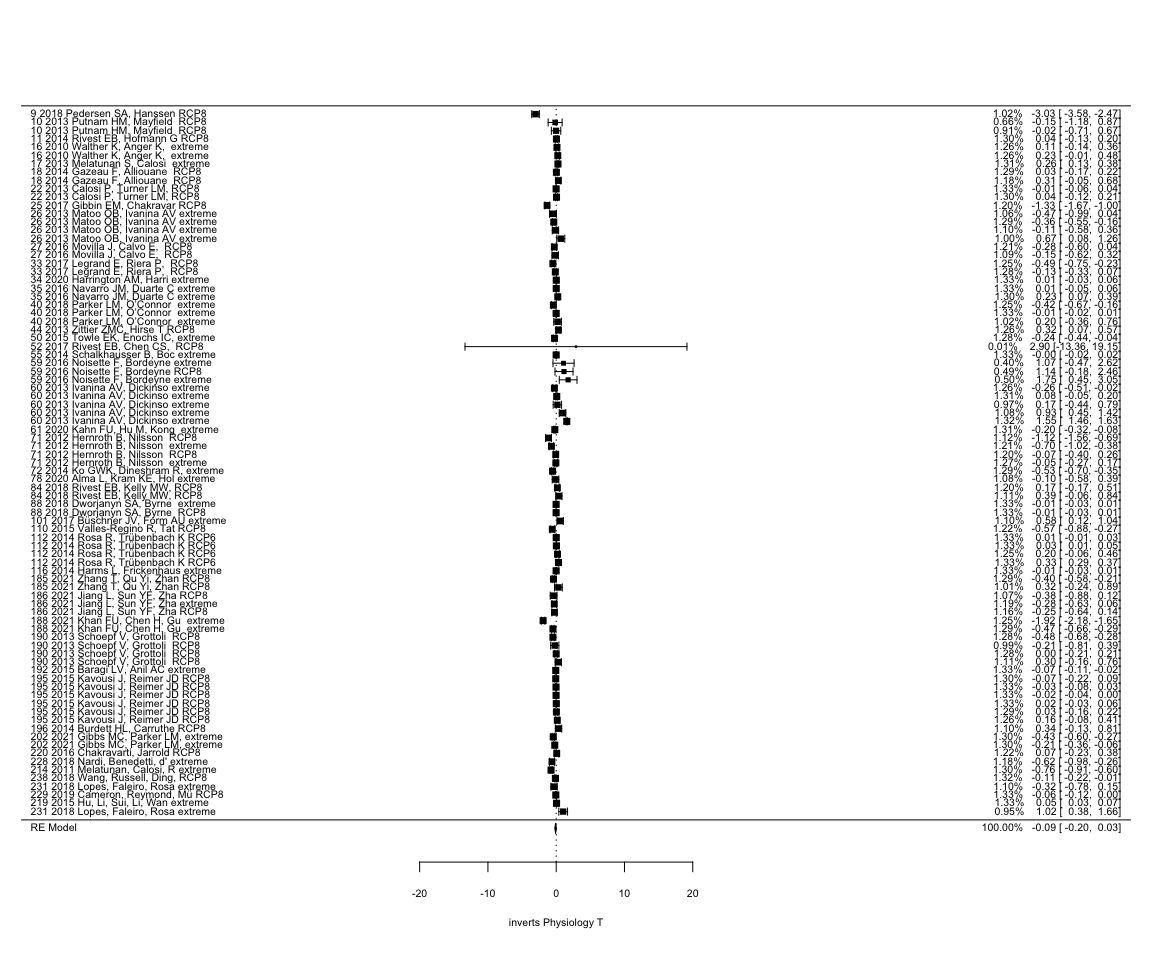


##
## Random-Effects Model (k = 92; tau^2 estimator: REML)
##
## tau^2 (estimated amount of total heterogeneity): 0.1622 (SE = 0.0276)
## tau (square root of estimated tau^2 value): 0.4027
## I^2 (total heterogeneity / total variability): 99.54%
## H^2 (total variability / sampling variability): 216.10
##
## Test for Heterogeneity:
## Q(df = 91) = 2016.3204, p-val < .0001
##
## Model Results:
##
## estimate se zval pval ci.lb ci.ub ​
## -0.0267 0.0457 -0.5846 0.5588 -0.1162 0.0628
##
## ---
## Signif. codes: 0 '***' 0.001 '**' 0.01 '*' 0.05 '.' 0.1 ' ' 1


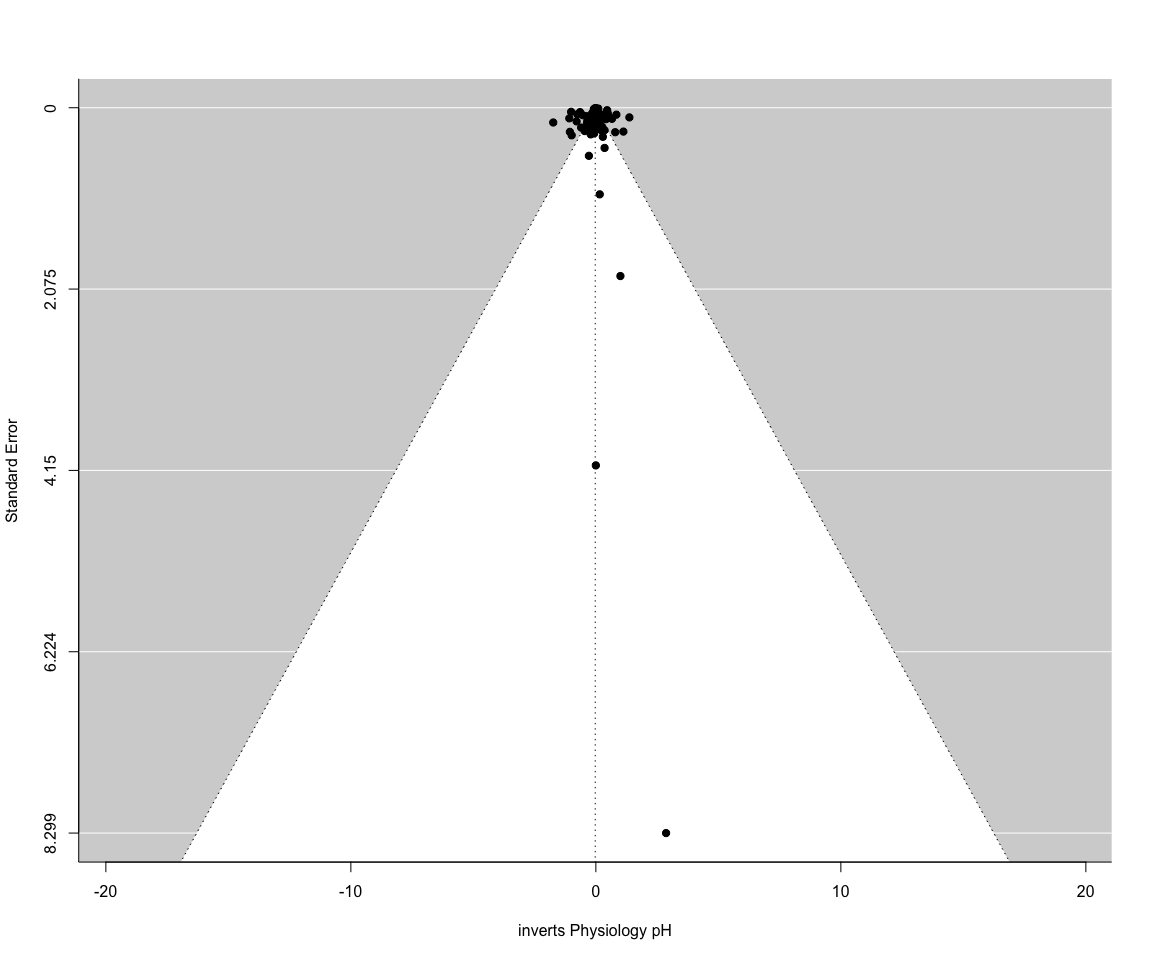

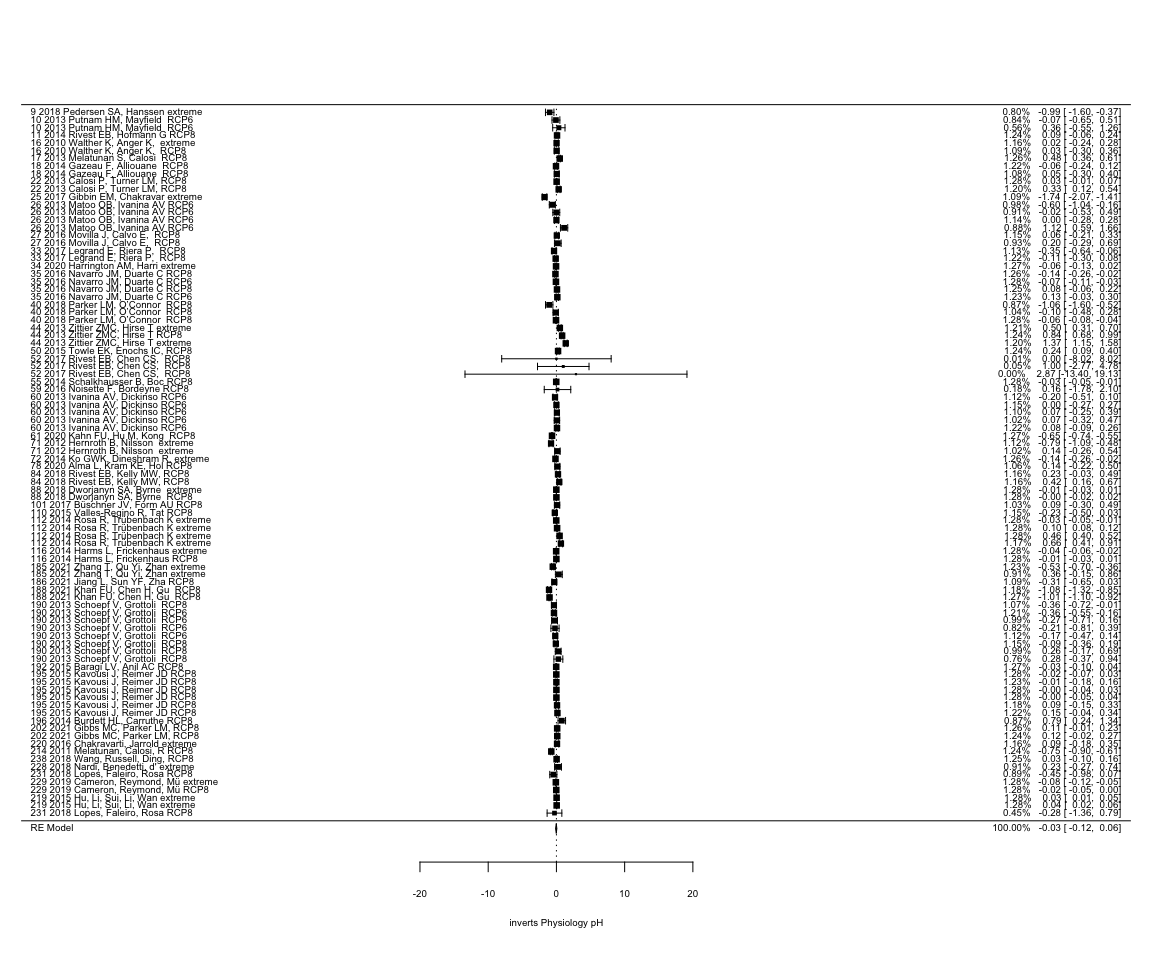


##
## Random-Effects Model (k = 104; tau^2 estimator: REML)
##
## tau^2 (estimated amount of total heterogeneity): 0.2533 (SE = 0.0401)
## tau (square root of estimated tau^2 value): 0.5033
## I^2 (total heterogeneity / total variability): 99.67%
## H^2 (total variability / sampling variability): 306.11
##
## Test for Heterogeneity:
## Q(df = 103) = 2937.6895, p-val < .0001
##
## Model Results:
##
## estimate se zval pval ci.lb ci.ub ​
## -0.0401 0.0533 -0.7524 0.4518 -0.1446 0.0644
##
## ---
## Signif. codes: 0 '***' 0.001 '**' 0.01 '*' 0.05 '.' 0.1 ' ' 1

Abs_InvertPhys <- MA_TpH_abs("inverts","Physiology", Inverts)
## Fish, Physiology
FishPhys <- MA_TpH("fish","Physiology", Fish,sensitivity)

## Warning: Fisher scoring algorithm may have gotten stuck at a local maximum.
## Setting tau^2 = 0. Check the profile likelihood plot with profile().

## Random-Effects Model (k = 72; tau^2 estimator: REML)
##
## tau^2 (estimated amount of total heterogeneity): 0.0754 (SE = 0.0158)
## tau (square root of estimated tau^2 value): 0.2746
## I^2 (total heterogeneity / total variability): 97.91%
## H^2 (total variability / sampling variability): 47.89
##
## Test for Heterogeneity:
## Q(df = 71) = 991.0182, p-val < .0001
##
## Model Results:
## ## estimate se zval pval ci.lb ci.ub ​
## 0.0467 0.0368 1.2701 0.2040 -0.0254 0.1189
## ---
## Signif. codes: 0 '***' 0.001 '**' 0.01 '*' 0.05 '.' 0.1 ' ' 1

##
## Random-Effects Model (k = 62; tau^2 estimator: REML)
##
## tau^2 (estimated amount of total heterogeneity): 0.0226 (SE = 0.0063)
## tau (square root of estimated tau^2 value): 0.1502
## I^2 (total heterogeneity / total variability): 93.21%
## H^2 (total variability / sampling variability): 14.73
##
## Test for Heterogeneity:
## Q(df = 61) = 530.0944, p-val < .0001
##
## Model Results:
##
## estimate se zval pval ci.lb ci.ub ​
## -0.0249 0.0246 -1.0122 0.3115 -0.0732 0.0233
##
## ---
## Signif. codes: 0 '***' 0.001 '**' 0.01 '*' 0.05 '.' 0.1 ' ' 1

##
## Random-Effects Model (k = 78; tau^2 estimator: REML)
##
## tau^2 (estimated amount of total heterogeneity): 0.1276 (SE = 0.0239)
## tau (square root of estimated tau^2 value): 0.3572
## I^2 (total heterogeneity / total variability): 98.69%
## H^2 (total variability / sampling variability): 76.19
##
## Test for Heterogeneity:
## Q(df = 77) = 964.5850, p-val < .0001
##
## Model Results:
##
## estimate se zval pval ci.lb ci.ub ​
## 0.0225 0.0440 0.5109 0.6094 -0.0638 0.1088
##
## ---
## Signif. codes: 0 '***' 0.001 '**' 0.01 '*' 0.05 '.' 0.1 ' ' 1

Abs_FishPhys <- MA_TpH_abs("fish","Physiology", Fish)
