# Supplementary material for: Hidden impacts of ocean warming and acidification on biological responses of marine animals revealed through meta-analysis
Source: Nat Commun. 2024 Apr 3;15:2885. doi: 10.1038/s41467-024-47064-3 (PMC10991405; doi:10.1038/s41467-024-47064-3)
Supplement: Supplementary file 10 — Reporting Summary [file 41467_2024_47064_MOESM10_ESM.pdf]

nature portfolio

Corresponding author(s): Katharina Alter

Last updated by author(s): Apr 3, 2024

Reporting Summary

Nature Portfolio wishes to improve the reproducibility of the work that we publish. This form provides structure for consistency and transparency in reporting. For further information on Nature Portfolio policies, see our Editorial Policies and the Editorial Policy Checklist.

Statistics

For all statistical analyses, confirm that the following items are present in the figure legend, table legend, main text, or Methods section.

n/a	Confirmed	
☐	☒	The exact sample size (n) for each experimental group/condition, given as a discrete number and unit of measurement
☒	☐	A statement on whether measurements were taken from distinct samples or whether the same sample was measured repeatedly
☐	☒	The statistical test(s) used AND whether they are one- or two-sided<br>*Only common tests should be described solely by name; describe more complex techniques in the Methods section.*
☐	☒	A description of all covariates tested
☐	☒	A description of any assumptions or corrections, such as tests of normality and adjustment for multiple comparisons
☐	☒	A full description of the statistical parameters including central tendency (e.g. means) or other basic estimates (e.g. regression coefficient) AND variation (e.g. standard deviation) or associated estimates of uncertainty (e.g. confidence intervals)
☐	☒	For null hypothesis testing, the test statistic (e.g. F, t, r) with confidence intervals, effect sizes, degrees of freedom and P value noted<br>*Give P values as exact values whenever suitable.*
☒	☐	For Bayesian analysis, information on the choice of priors and Markov chain Monte Carlo settings
☐	☒	For hierarchical and complex designs, identification of the appropriate level for tests and full reporting of outcomes
☒	☐	Estimates of effect sizes (e.g. Cohen's d, Pearson's r), indicating how they were calculated

Our web collection on statistics for biologists contains articles on many of the points above.

Software and code

Policy information about availability of computer code

Data collection	For the search of publications reporting on experiments, we used ISI Web of Science and Google Scholar. For data extraction from graphs presented in those publications, we used the software GetData and WebPlotDigitizer. We summarized the data collection procedure in our PRISMA flow chart (supplementary file) and made the raw data publicly available in the Zenodo database under accession code 10223034 [Hidden impacts of climate change on biological responses of marine life (zenodo.org)].
Data analysis	Meta-analysis models and statistical tests were run using the {metafor} package in Rstudio version 2022.12.0+353. Specific information on data analysis is listed in detail under the method section.

For manuscripts utilizing custom algorithms or software that are central to the research but not yet described in published literature, software must be made available to editors and reviewers. We strongly encourage code deposition in a community repository (e.g. GitHub). See the Nature Portfolio guidelines for submitting code & software for further information.

Data

Policy information about availability of data

All manuscripts must include a data availability statement. This statement should provide the following information, where applicable:

- Accession codes, unique identifiers, or web links for publicly available datasets
- A description of any restrictions on data availability
- For clinical datasets or third party data, please ensure that the statement adheres to our policy

The data generated in this study have been deposited in the Zenodo database under accession code 10223034 [Hidden impacts of climate change on biological

responses of marine life (zenodo.org)]. The data used in this study are available in the Zenodo database under accession code 10223034 [Hidden impacts of climate change on biological responses of marine life (zenodo.org)].

Research involving human participants, their data, or biological material

Policy information about studies with human participants or human data. See also policy information about sex, gender (identity/presentation), and sexual orientation and race, ethnicity and racism.

Reporting on sex and gender	not applicable
Reporting on race, ethnicity, or other socially relevant groupings	not applicable
Population characteristics	not applicable
Recruitment	not applicable
Ethics oversight	not applicable

Note that full information on the approval of the study protocol must also be provided in the manuscript.

Field-specific reporting

Please select the one below that is the best fit for your research. If you are not sure, read the appropriate sections before making your selection.

☐ Life sciences ☐ Behavioural & social sciences ☒ Ecological, evolutionary & environmental sciences

For a reference copy of the document with all sections, see nature.com/documents/nr-reporting-summary-flat.pdf

Ecological, evolutionary & environmental sciences study design

All studies must disclose on these points even when the disclosure is negative.

Study description	This study evaluates the effect of ocean acidification and ocean warming on ten biological response categories of fish and invertebrates. Because previous meta-analysis report conflicting results on the impacts of these factors on marine organisms, we developed a meta-analysis that accounts for species-specific responses and for the ambiguous relation of most metrics to fitness. This new approach is based on the deviation of responses from the reference value (absolute change) that we complement with the commonly used meta-analyses of directional (relative) change from the reference value.
Research sample	We searched for papers in ISI Web of Science and Google Scholar that were published between 2008 and 2022. We included 217 papers reporting on full factorial designs with at least two temperatures and two pCO2 levels in our final dataset. From these papers, we extracted 3162 control-treatment comparisons for biological responses that we classified into 10 categories (behaviour, biodiversity, biomechanics, calcification, development, growth, physiology, reproduction, metabolism, survival)
Sampling strategy	This study evaluates the effect of ocean acidification and ocean warming on ten biological response categories of fish and invertebrates. Because previous meta-analysis report conflicting results on the impacts of these factors on marine organisms, we developed a meta-analysis that accounts for species-specific responses and for the ambiguous relation of most metrics to fitness. This new approach is based on the deviation of responses from the reference value (absolute change) that we complement with the commonly used meta-analyses of directional (relative) change from the reference value.
Data collection	Data collection was performed through a systematic literature review on Google Scholar and ISI Web of Science. Screening of titles, abstracts and full research articles was performed by co-authors: KA, PHM, CPG, MEL, MEB, SM, MM, CC, PD
Timing and spatial scale	We considered papers from around the world that were published between 2008 and 2022
Data exclusions	We excluded studies from our systematic review based on the criteria described in our method section and PRISMA flow chart (e.g., only investigating warming or acidification; missing statistical information etc...)
Reproducibility	The procedure for the systematic literature search and a description of the meta-analyses performed are provided in detail in the methods section. Raw data, source data and supplementary data and files are publicly available. The doi for the data and code are stated in the data and code availability section of our manuscript
Randomization	To ensure that no paper was given a higher weight in the final meta-analysis results we limited the maximum values extracted from each paper to two. This is described in detail in the methods section and data is provided in the supplementary material.

Blinding	When paper titles and abstracts were screened for suitability for our meta-analysis, bibliographic data such as journal and authors were excluded. Blinding of descriptive and quantitative data was not applied because this data is necessary when performing the meta-analyses

Did the study involve field work? ☐ Yes ☒ No

Reporting for specific materials, systems and methods

We require information from authors about some types of materials, experimental systems and methods used in many studies. Here, indicate whether each material, system or method listed is relevant to your study. If you are not sure if a list item applies to your research, read the appropriate section before selecting a response.

Materials & experimental systems

n/a	Involved in the study
☒	☐ Antibodies
☒	☐ Eukaryotic cell lines
☒	☐ Palaeontology and archaeology
☒	☐ Animals and other organisms
☒	☐ Clinical data
☒	☐ Dual use research of concern
☒	☐ Plants

Methods

n/a	Involved in the study
☒	☐ ChIP-seq
☒	☐ Flow cytometry
☒	☐ MRI-based neuroimaging
